# Transcriptomic analysis of salt stress responsive genes in *Rhazya stricta*

**DOI:** 10.1371/journal.pone.0177589

**Published:** 2017-05-16

**Authors:** Nahid H. Hajrah, Abdullah Y. Obaid, Ahmed Atef, Ahmed M. Ramadan, Dhivya Arasappan, Charllotte A. Nelson, Sherif Edris, Mohammed Z. Mutwakil, Alawia Alhebshi, Nour O. Gadalla, Rania M. Makki, Madgy A. Al-Kordy, Fotouh M. El-Domyati, Jamal S. M. Sabir, Mohammad A. Khiyami, Neil Hall, Ahmed Bahieldin, Robert K. Jansen

**Affiliations:** 1 Biotechnology Research Group, Department of Biological Sciences, Faculty of Science, King Abdulaziz University (KAU), Jeddah, Saudi Arabia; 2 Department of Chemistry, Faculty of Science, King Abdulaziz University (KAU), Jeddah, Saudi Arabia; 3 Agricultural Genetic Engineering Research Institute (AGERI), Agriculture Research Center (ARC), Giza, Egypt; 4 Department of Integrative Biology, University of Texas at Austin, Austin, Texas, United States of America; 5 Centre of Genomic Research, Institute for Integrative Biology, Crown Street, Liverpool, United Kingdom; 6 Department of Genetics, Faculty of Agriculture, Ain Shams University, Cairo, Egypt; 7 Princess Al-Jawhara Al-Brahim Centre of Excellence in Research of Hereditary Disorders (PACER-HD), Faculty of Medicine, King Abdulaziz University (KAU), Jeddah, Saudi Arabia; 8 Department of Arid Land Agriculture, Faculty of Meteorology, Environment and Arid Land Agriculture, King Abdulaziz University, Jeddah, Saudi Arabia; 9 Genetics and Cytology Department, Genetic Engineering and Biotechnology Division, National Research Center, Dokki, Egypt; 10 King Abdulaziz City for Science and Technology (KACST), Riyadh, Saudi Arabia; 11 The Earlham Institute, Norwich Research Park, Norwich, United Kingdom; University of Western Sydney, AUSTRALIA

## Abstract

*Rhazya stricta* is an evergreen shrub that is widely distributed across Western and South Asia, and like many other members of the Apocynaceae produces monoterpene indole alkaloids that have anti-cancer properties. This species is adapted to very harsh desert conditions making it an excellent system for studying tolerance to high temperatures and salinity. RNA-Seq analysis was performed on *R*. *stricta* exposed to severe salt stress (500 mM NaCl) across four time intervals (0, 2, 12 and 24 h) to examine mechanisms of salt tolerance. A large number of transcripts including genes encoding tetrapyrroles and pentatricopeptide repeat (PPR) proteins were regulated only after 12 h of stress of seedlings grown in controlled greenhouse conditions. Mechanisms of salt tolerance in *R*. *stricta* may involve the upregulation of genes encoding chaperone protein Dnaj6, UDP-glucosyl transferase 85a2, protein transparent testa 12 and respiratory burst oxidase homolog protein b. Many of the highly-expressed genes act on protecting protein folding during salt stress and the production of flavonoids, key secondary metabolites in stress tolerance. Other regulated genes encode enzymes in the porphyrin and chlorophyll metabolic pathway with important roles during plant growth, photosynthesis, hormone signaling and abiotic responses. Heme biosynthesis in *R*. *stricta* leaves might add to the level of salt stress tolerance by maintaining appropriate levels of photosynthesis and normal plant growth as well as by the participation in reactive oxygen species (ROS) production under stress. We speculate that the high expression levels of PPR genes may be dependent on expression levels of their targeted editing genes. Although the results of PPR gene family indicated regulation of a large number of transcripts under salt stress, PPR actions were independent of the salt stress because their RNA editing patterns were unchanged.

## Introduction

Plant species have developed a series of mechanisms to adjust to environmental stresses [1, 2, 3]. These mechanisms act as regulatory networks of genes and pathways that crosstalk in order to cope with stress. An excellent example involves the genes encoding the plant hormone abscisic acid (ABA) [4, 5, 6]. Processes like plant growth and photosynthesis are greatly affected by salt stress due to the reduction of chlorophyll biosynthesis, which has a major effect on crop productivity [7]. Strategies of salt tolerance in plants include the maintenance of tetrapyrrole levels (e.g., siroheme, chlorophyll, heme and phytochromobilin) under stress [8]. Tetrapyrroles are mostly synthesized in the chloroplast, with the last two steps of heme synthesis occurring in mitochondria [9]. Several enzymes in the porphyrin and chlorophyll metabolism pathway are key players in the production of tetrapyrroles that maintain stay-green performance of wild plant species under adverse environmental conditions like drought and salinity. These enzymes involve 5-aminolevulinic acid (ALA) dehydratase, porphobilinogen deaminase, coproporphyrinogen III oxidase, protoporphyrinogen IX oxidase, Mg-protoporphyrin IX chelatase and protochlorophyllide oxidoreductase.

The gene superfamily encoding pentatricopeptide repeat (PPR) proteins is one of the largest gene families in plants [10], with about 450 members in *Arabidopsis thaliana* and over 600 in *Oryza sativa* (rice) [11, 12, 13]. These proteins are mostly targeted to mitochondria or chloroplasts and contribute in RNA processing, including RNA editing and stability [11, 14, 15]. Despite the paucity of information on mechanisms by which PPR proteins function in organelles, some are involved in plant developmental processes and respond positively to environmental stresses [16, 17, 18, 19, 20, 21, 22]. For example, the *Arabidopsis* chloroplast PPR protein SVR7 is involved in photosynthesis and oxidative stress tolerance [21], while six mitochondrial proteins (PPR40 [16]), ABO5 [17], Ahg11 [19], SLG1 [20], PGN [18] and SLO2 [22]) contribute to the regulation of ABA signaling, and consequently to salt or drought stress responses. Knockout mutants of some PPR genes were hypersensitive to salt or osmotic stress during germination and early growth stages [16, 19, 22]. These studies suggest a highly complicated mechanism by which mitochondrial/chloroplast PPRs are involved in regulating plant responses to abiotic stresses. It was also proposed that some PPRs may be involved in the regulation of homeostasis of reactive oxygen species (ROS) during stress responses or ABA signaling in the development of stress tolerance [23]. One nuclear PPR protein, SOAR1, has a positive role in plant response to abiotic stresses such as drought, high salinity and low temperature [23]. A previous report addressed the function of a nuclear and cytosolic PPR protein, PNM1, and indicated that it does not play a role in plant responses to abiotic stresses [24].

In the present study, high-throughput RNA-Seq was performed on *Rhazya*. *stricta* leaves exposed to severe salt stress (500 mM NaCl). This species is a member of the flowering plant family Apocynaceae and is included in a clade that is well-known for accumulating a wide diversity of monoterpene indole alkaloids (MIAs) that have important anticancer properties [25]. *Rhazya stricta* is an evergreen shrub that inhabits arid zones throughout Western and South Asia so it is well-adapted to harsh temperatures and salinity, making this species an excellent system for investigating the genes involved in tolerance to these conditions. The results of the present study indicated the possible roles of genes involved in protein folding and in porphyrin and chlorophyll metabolism pathway in conferring salt tolerance. Another phenomenon was also observed during the response of *R*. *stricta* leaves to salt stress, the upregulation of a large number of PPR- and tetrapyrrole-encoding genes only after 12 h of salt stress treatment. The explanation for this phenomenon requires further investigation.

## Materials and methods

### Salt stress experiment

Seeds of *Rhazya stricta* were collected from the Bahrah region, Jeddah, Saudi Arabia. Permission to collect *R*. *stricta* plant material and to perform fieldwork at the site was granted by the Governor of Makkah Province, Prince Khalid Al- Faisal. A voucher specimen has been deposited in the Department of Biological Sciences Herbarium at King Abdulaziz University (Number 1150/M/75 collected by N. Baeshen, M. Baeshen and J. Sabir). Twenty-four 30-day-old seedlings of *R*. *stricta* were grown in six pots (4 seedlings/pot) with a potting mix consisting of one part vermiculite and one part perlite. Plantlets were grown under a 16-h-light/8-h-dark cycle at 21±2°C (day/night), light intensity of ~175 uE m^–2^ sec^–1^ for the 16-h photoperiod and 80% humidity. At the time of treatment, leaf samples (control, unirrigated) were harvested from three arbitrarily chosen pots in which four leaves (one from each seedling) of each pot were gathered as one replicate. Seedlings of three pots were salt-stressed (500 mM NaCl) and leaf samples were harvested after 2, 12 and 24 h. The NaCl concentration and the harvest times were selected to be consistent with a previous study of salt stress in wild barley [26] so that the results of both studies could be compared. Concurrently, seedlings of the other three pots were water-irrigated and leaf samples were also harvested after 2, 12 and 24 h. For isolating total RNAs, flash-frozen similar-sized leaf samples from plants of each pot were crushed into a fine powder in a microcentrifuge tube using a sterilized metal rod. Total RNAs were extracted using Trizol (Invitrogen, Life Tech, Grand Island, NY, USA) and treated with RNase-free DNase (Promega Corporation, Madison, WI, USA) in the presence of 1 U/μl of RNasin® Plus RNase Inhibitor (Promega) for 2 h at 37^°^C. RNAs were quantified and 30 μg (400 ng/μl) was used for RNA-Seq. To test for the presence of DNA contamination in RNA samples, the *actin* gene of *R*. *stricta* was amplified by PCR from the original RNA samples. Purified RNA samples were shipped to Centre for Genomic Research, Institute of Integrative Biology, University of Liverpool, UK in three replicates of each treatment for deep sequencing and generation of datasets (an average of ~100 million reads per sample).

### Next-generation mRNA sequencing

Filtered reads were aligned with up to two mismatches to the *R*. *stricta* reference nuclear genome [25] available at NCBI (accession no. MEJB00000000.1). The remaining unmapped sequences were re-aligned against the contigs and collectively *de novo* assembled using the Trinity RNA-Seq Assembly package (r2013-02-25) following the methods described in Zhang *et al*. [27]. RSEM v1.1.6, an RNA-Seq quantification tool, was used to estimate the relative abundances and expected read counts for the transcripts. RSEM uses the Bowtie aligner (Bowtie v0.12.1) to map the reads against the transcripts. Transcript quantification of the reference-aligned as well as the *de novo* assembled reads was performed with RSEM, which allowed for the assessment of transcript abundances based on the mapping of RNA-Seq reads to the assembled transcriptome. Expected read counts were input into differential expression (DE) analysis using EdgeR (version 3.0.0, R version 2.1.5). The median value from biological replicates was used as the common dispersion factor for DE analyses. DE transcripts were annotated and KEGG pathway analyses were performed using blast-2-GO software (version 2.3.5, http://www.blast2go.com/) and GO terms were obtained with the default parameters. Blastx was performed against the NCBI non-redundant protein database with an E-value cut off of 1e^-5^. To identify clusters with functional enrichment, we determined a significant Pearson correlation through permutation analysis [28]. The resulting clusters were refined by visual inspection and analyzed for GO term enrichment using Blast2GO (http://www.blast2go.com/). We also clustered the RPKM data to provide a representation of absolute abundance of the transcripts.

### Validation experiments of RNA-seq data

Validation of the RNA-Seq data was performed via semi-quantitative RT-PCR for randomly selected transcripts of genes encoding PPR proteins that were upregulated at the 12 h time point. The *actin* gene of *R*. *stricta* was used as the unregulated housekeeping gene. Primers were designed with Netprimer software (http://www.premierbiosoft.com/netprimer/index.html) using five criteria: length ~26 basepairs, GC content ~50%, minimal secondary structure, comparable annealing temperatures (55–56^°^C) of the primer pairs and PCR products of ~300–400 bp.

## Results and discussion

Sequencing of cDNA samples of *R*. *stricta* leaves under control conditions and salt stress (500 mM NaCl) treatments across time (0–24 h) yielded 86–154 million reads corresponding to an average of ~10 billion nucleotides of cDNA per sample (Table 1). The raw sequencing reads were submitted to the Small Read Archive (SRA) at NCBI (Experiment no. PRJNA355223). Approximately, 45% of the reads mapped to the *R*. *stricta* reference nuclear genome and the remaining 55% matched no sequences (Table 1). The mapped sequences are in exonic regions. The unmapped reads were aligned to the *de novo*-assembled transcriptome and between 39.13 and 57.56% of the draft genome-unmapped reads were *de novo*-mapped to the *de novo*-assembled genome. Thus, ~72% of the total reads of each sample were either mapped to the *R*. *stricta* nuclear genome or to the *de novo* assembled transcriptome, and these sequences were subjected to further analyses.

**Table 1:** Statistics of *R*. *stricta* RNA-Seq numerical data analysis. 0h c1,2 (control), 2h-w1,2 (water treated for 2 h), 12h-w1,2,3 (water treated for 12 h), 24h-w1 (water treated for 24 h), 2h-s1,2,3 (salt stressed fo r 2 h), 12h-s1,2,3 (salt stressed for 12 h), 24h-s1,2,3 (salt stressed for 24 h).

Time-condition	Total no. reads^1^	Mapped reads^2^ (draft genome)	%^3^	Unmapped reads^4^	%^5^	Mapped reads^6^ (*de novo*)	%^7^	%^8^
0h-c1	87,151,878	39,445,080	45.26	47,706,798	54.74	23,643,555	27.13	72.39
0h-c2	153,943,752	69,191,898	44.95	84,751,854	55.05	33,159,843	21.54	66.49
2h-w1	87,639,149	39,195,497	44.72	48,443,652	55.28	24,308,107	27.74	72.46
2h-w2	89,863,836	40,022,306	44.54	49,841,530	55.46	24,077,557	26.79	71.33
12h-w1	89,383,819	38,176,106	42.71	51,207,713	57.29	24,485,660	27.40	70.11
12h-w2	85,958,876	38,998,763	45.37	46,960,113	54.63	24,094,502	28.03	73.40
12h-w3	92,526,665	42,125,366	45.53	50,401,299	54.47	26,189,855	28.31	73.84
24h-w1	86,673,531	39,179,668	45.20	47,493,863	54.80	24,865,085	28.69	73.89
2h-s1	89,056,769	40,170,783	45.11	48,885,986	54.89	25,519,598	28.66	73.77
2h-s2	91,575,385	41,804,669	45.65	49,770,716	54.35	25,323,495	27.65	73.30
2h-s3	95,132,204	42,838,000	45.03	52,294,204	54.97	26,498,735	27.86	72.89
12h-s1	86,260,164	39,645,187	45.96	46,614,977	54.04	22,838,921	26.48	72.44
12h-s2	97,705,696	44,756,425	45.81	52,949,271	54.19	27,177,669	27.82	73.63
24h-s1	89,710,811	40,750,202	45.42	48,960,609	54.58	28,179,718	31.41	76.83
24h-s2	98,243,664	44,727,450	45.53	53,516,214	54.47	24,044,493	24.47	70.00
24h-s3	91,434,299	41,529,282	45.42	49,905,017	54.58	26,987,468	29.52	74.94

^1^ Total number of reads recovered from RNA-Seq

^2^ Number of reads aligned with R. stricta draft genome

^3^ Percentage of reads aligned with draft genome over total reads

^4^ Number of reads unaligned with draft genome

^5^ Percentage of reads unaligned with draft genome over total reads

^6^ Number of reads aligned with de novo-assembled genome

^7^ Percentage of reads aligned with de novo-assembled genome over total de novo-assembled reads

^8^ Percentage of reads aligned with draft and de novo-assembled genomes

**Fig 1:**
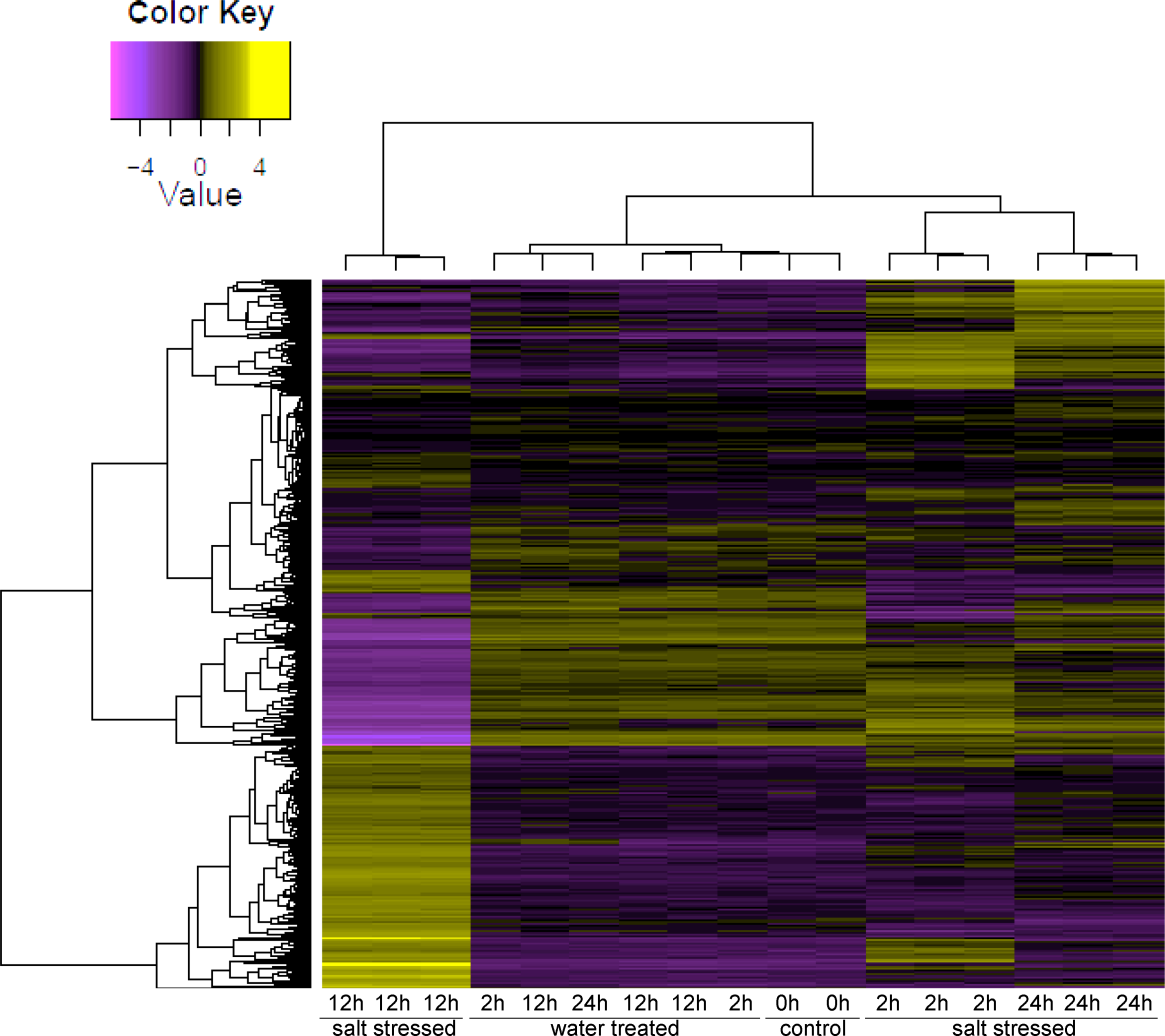
Hierarchical cluster analysis of gene expression based on log ratio RPKM data for leaf transcriptome of *R*. *stricta* under water and salt (500 mM NaCl) treatments for 0, 2, 12 and 24 h.

RNA-Seq analysis of *R*. *stricta* leaves was performed to detect changes in expression levels of genes across four time points (0, 2, 12 and 24 h) of salt stressed (500 mM NaCl) plants. Hierarchical cluster analysis of gene expression based on log ratio RPKM data for transcripts of *R*. *stricta* across different time points indicated the high quality of sampling and RNA-Seq analysis as evidenced by within time point clustering of replicates in different samples (Fig 1). Expression of genes in control samples (0 h) was similar to those treated with water across all four time points of the experiment (up to 24 h). Transcripts at 12 h post treatment were distinct from the genes regulated at other time points. This indicates that a large number of genes were either up- or downregulated at this time point. Therefore, we decided to restrict our characterization of genes to this time point because they represent the most responsive genes to salt stress in *R*. *stricta*. The number of clusters of DE transcripts was 236 (S1 Fig); 31 clusters were upregulated, while 53 clusters were downregulated only at the 12 h time point. The total number of upregulated and downregulated transcripts at this time point was ~5700 (S1 Table) and ~5100 (S2 Table), respectively. A similar pattern of transcript regulation only at the 12 h time point of salt stress was previously detected in wild barley [26]. Semi-quantitative RT-PCR (sqRT-PCR) of 10 randomly chosen upregulated transcripts of the PPR gene family was performed to validate the RNA-Seq data using specific primers (S3 Table). Analysis of sqRT-PCR indicated that the 10 PPR genes were upregulated only after 12 h post salt treatment supporting the results of RNA-Seq analyses (S2 Fig).

**Fig 2:**
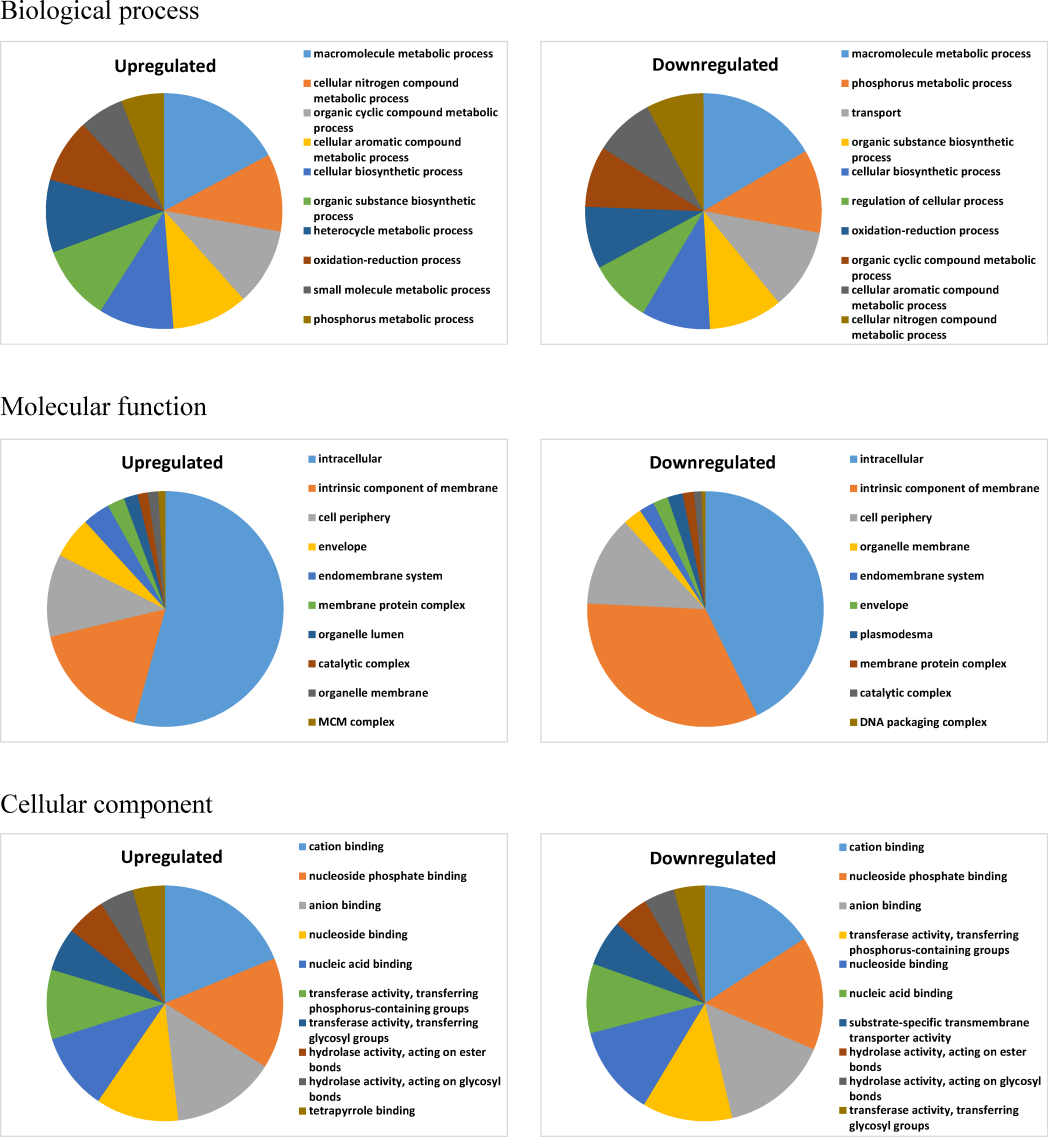
Gene ontology analysis describing the three main categories “biological process”, “molecular function” and “cellular component” for the 10 functional groups with the largest number of upregulated or downregulated transcripts under salt stress.

Differentially expressed genes were assigned to functional categories using blast2GO (http://www.blast2go.com/), which provided valuable information for detecting specific processes, functions and pathways during salt stress. We selected 10 functional groups with the largest number of transcripts, either upregulated or downregulated, for the three main categories (Fig 2). Subgroups of biological process with the largest number of transcripts are macromolecule metabolic process across both types of regulation. Cation binding was the function with the largest number of transcripts across the two types of regulation. The largest number of transcripts in the cellular component category encode intracellular compounds (Fig 2). Transcripts for tetrapyrrole binding were upregulated and no records for downregulated transcripts involved in this function were detected.

In *R*. *stricta* the overall results indicated limited involvement of genes and pathways previously known to respond to salt stress. Our recent work [29] indicated the involvement of several genes and pathways in regulating thermotolerance in this species. As many as 32 enzymes were affected in several pathways involved starch and sucrose metabolism, galactose metabolism, phenylpropanoid biosynthesis, flavonoid biosynthesis and cysteine and methionine metabolism. Earlier studies on salt stress-responsive pathways in other plant species indicated the involvement of these pathways in promoting salt stress tolerance [30, 31, 32, 33, 34]. We speculate that the unusual response to salt stress in *R*. *stricta* is because this desert-grown plant is not accustomed to such stress, hence, it may lack the salt-responsive regulatory machinery present in halophytes and glycophytes. We also speculate that extended exposure to salt stress might promote proper responses as the plant may adapt to this new type of stress over time. Exposure to gradual increases of salt concentrations might also allow the different genes to crosstalk and generate responses previously detected in other plant species.

### Regulated gene families with ≥ 5 fold change

Ninety-five transcripts were regulated under salt stress with fold change (FC) ≥ 5 (S4 Table), 27 of which were upregulated only at 12 h time point. They include transcripts encoding chaperone protein DnaJ6, UDP-glucosyltransferase 85a2 (UGT85A2), protein transparent testa 12 and respiratory burst oxidase homolog protein b (RBOH). Laufen *et al*. [35] indicated that the DnaJ gene family acts as a molecular chaperone that binds to adenosine triphosphate (ATP)-ligated form of DnaK to stimulate its hydrolysis to adenosine 5′-diphosphate [36]. The basic action of chaperones is to prevent proteins from misfolding/aggregating or denaturing during abiotic stresses including salinity [37]. The gene family encoding UDP-glucosyltransferase has an important role in quality control of newly synthesized glycoproteins in the endoplasmic reticulum (ER). It is well-known that the ER hosts the synthesis/folding of proteins secreted extracellularly or delivered to the endomembrane system [38]. Thus, the enzyme acts like a chaperone to assist the cell in maintaining proper protein folding during salt stress. The protein transparent testa 12 (tt12) gene family is known for its action in sequestering flavonoids in seed coat endothelium. Wahid and Ghazanfar [39] indicated that flavonoids are key secondary metabolites in environmental stress tolerance. The plant respiratory burst oxidase homolog gene family plays a crucial role in plant growth, biotic/abiotic responses and hormone signaling [40]. The family encodes the key enzymatic subunit of NADPH oxidase, which is the major source of ROS in plants. ROS plays a major role during plant response to abiotic stresses [41]. Our prior analysis of *R*. *stricta* transcriptomes under heat stress supports the data generated for genes encoding chaperones and those encoding protein transparent testa 12 (tt12) under salt stress, where these genes were also highly upregulated under heat stress [29]. This indicates that mechanisms of tolerance against heat and salt stresses in this wild plant species rely partially on the response of the genes preventing degradation or protecting protein folding under either stress.

### Tetrapyrrole regulation under salt stress

**Fig 3:**
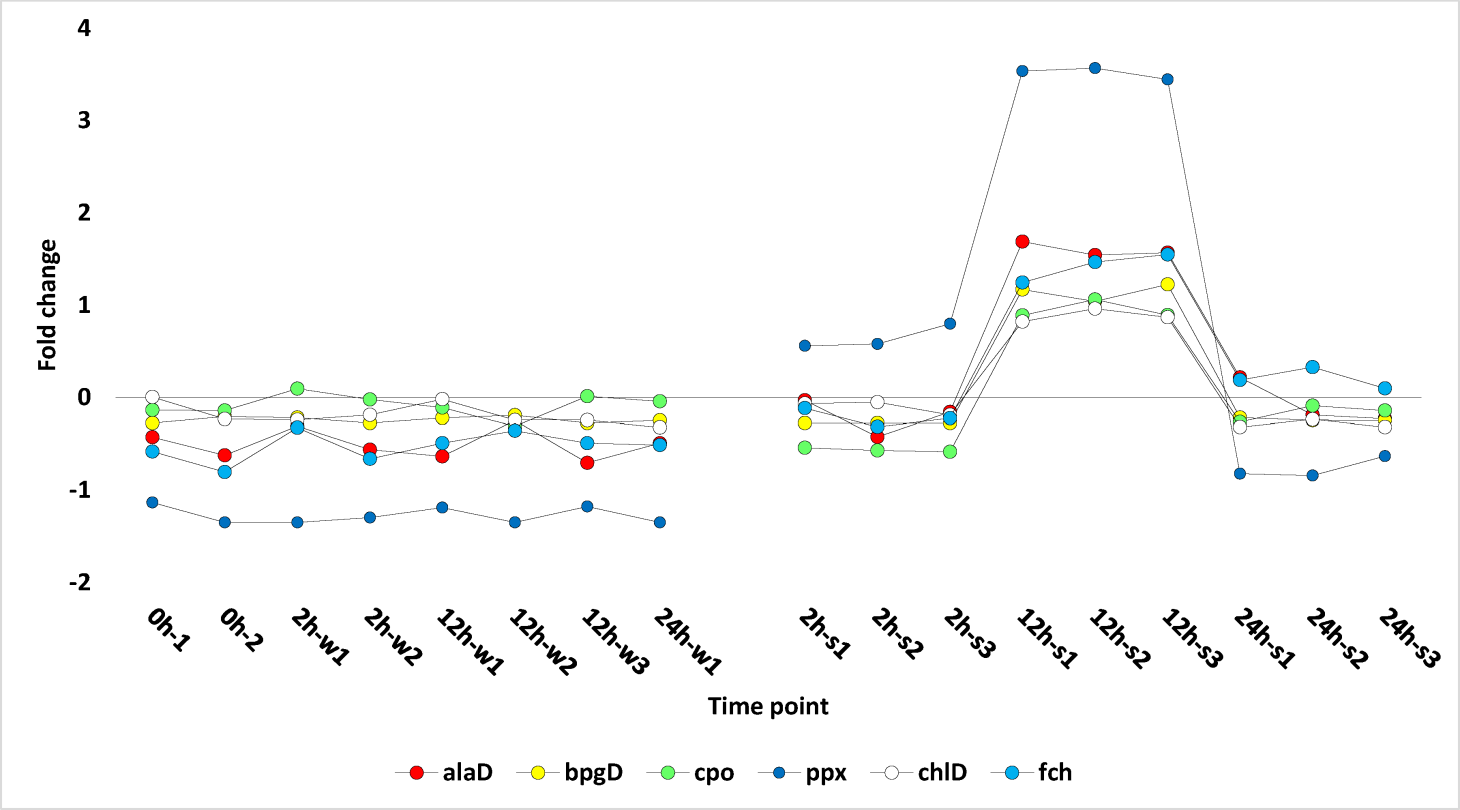
Profiles of fold change values for six transcripts (*alaD*, *pbgD*, *cpo*, *ppx*, *chlD* and *fch*) encoding enzymes in the porphyrin and chlorophyll metabolism pathway in leaves of *R*. *stricta* under water and salt stress treatments for 0, 2 12 and 24 h. 0h1,2 (control), 2h-w1,2 (water treated for 2 h), 12h-w1,2,3 (water treated for 12 h), 24h-w1 (water treated for 24 h), 2h-s1,2,3 (salt stressed for 2 h), 12h-s1,2,3 (salt stressed for 12 h), 24h-s1,2,3 (salt stressed for 24 h). Information about the different genes is shown in S1 and S5 Tables.

All DE transcripts were mapped to reference canonical pathways in the Kyoto Encyclopedia of Genes and Genomes (KEGG) (http://www.genome.ad.jp/kegg/) to identify the biological pathways that are activated or suppressed at the 12 h time point in *R*. *stricta*. Enzymes with roles in the pathways that showed regulation were examined. KEGG analysis detected no specific regulation of key enzymes of the different pathways except for the porphyrin and chlorophyll metabolism pathway (S3 Fig) that is basically light-inducible [42]. KEGG analysis of the porphyrin and chlorophyll metabolism pathway indicated that six enzymes with key roles in the pathway were activated. These results support the gene ontology analysis (Fig 2) that showed upregulation of transcripts encoding enzymes for tetrapyrrole binding. The regulated key enzymes in this pathway are 5-aminolevulinic acid (ALA) dehydratase (EC-2.4.1.24), porphobilinogen deaminase (EC-2.5.1.61), coproporphyrinogen III oxidase (EC-1.3.3.3), protoporphyrinogen IX oxidase (EC-1.3.3.4), Mg-protoporphyrin IX chelatase (EC-6.6.1.1) and ferrochelatase (EC-4.99.1.1). These enzymes are nuclear-encoded, but the pathway is located in chloroplasts and mitochondria, and they are important for biosynthesizing chlorophyll and heme in plant leaves [8]. Genes encoding these six enzymes are *alaD*, *pbgD*, *cpo*, *ppx*, *chlD* and *fch*, respectively. These genes were highly upregulated after 12 h of salt stress treatment (Fig 3). The first four genes encode enzymes involved in biosynthesis in chloroplasts, while last two enzymes act in directing the pathway towards heme biosynthesis in both chloroplasts and mitochondria. The two tetrapyrroles (chloroplast and heme) are important for plant growth and development, especially under abiotic stresses [8]. The Mg-chelatase enzyme was also reported to respond to changing environments [8]. This important pathway is usually severely affected by salt stress in plants. Our experiments indicate that *R*. *stricta* has the capacity to stay-green and produce important components of mitochondria, thus maintaining normal rates of photosynthesis, respiration and plant growth under harsh conditions. This could be a major mechanism of tolerance against environmental stresses for a wild plant species like *R*. *stricta*.

Previous reports have indicated that ALA synthesis is the initial rate-limiting step in the tetrapyrrole biosynthesis pathway [8]. This step results in the conversion of glycine to ALA due to the action of ALA synthase or ALAS (EC-2.3.1.37) (S3 Fig, enzyme indicated by the blue arrow). However, the present study indicates that the gene encoding ALAS is likely unregulated under salt stress in *R*. *stricta*. This resulted in no higher activation rate of the encoded enzyme (e.g., ALAS) (S3 Fig) under salt stress compared to normal conditions. Papenbrock and Grimm [43] indicated that heme can act as a feedback regulator of this step and other rate-limiting steps of the pathway. In the present study, heme is expected to result in a higher rate under salt stress due to the high activation rates of four enzymes (e.g., EC-4.2.1.24, EC-2.5.1.61, EC-1.3.3.3 and Ec-1.3.3.4) in the upstream steps of the pathway under salt stress (S3 Fig, enzymes indicated by red arrows). This might justify the recovery of the pathway despite the unchanged activation rate of the initial rate-limiting enzyme ALAS under salt stress. Although two chelating reactions result in the synthesis of heme, the responsible enzymes Mg-chelatase (EC-6.6.1.1) (S3 Fig, indicated by the green arrow) and Fe-chelatase (EC-4.99.1.1) (S3 Fig, indicated by the orange arrow) are functionally different. The first enzyme has greater influence on the pathway because it is subject to rigid control of gene expression and enzyme activity, hence, it is considered a reliable checkpoint monitor of metabolic flow of the pathway [43]. Heme seems to have roles other than those in the tetrapyrrole biosynthetic pathway, including participation in producing ROS required for normal plant growth under abiotic stresses [43]). In addition, heme biosynthesis can provide a mechanism by which leaves of *R*. *stricta* stay green and maintain appropriate levels of photosynthesis under salt stress. Intermediate tetrapyrroles also participate in several regulatory functions, including cross-talking between chloroplast and nucleus to synchronize their functions [44]. These tetrapyrroles can also block other pathways such as carotenoid biosynthesis in order to provoke the cell to produce ALA and activate the tetrapyrrole biosynthetic pathway [43]. In general, high rate of heme biosynthesis in leaves of *R*. *stricta* under salt stress may provide a mechanism of tolerance.

### PPR regulation under salt stress

The PPR gene superfamily plays an essential role in RNA editing in both chloroplasts [45] and mitochondria [46]. RNA editing is one of the major posttranscriptional RNA maturation processes that results in changing the information originally encoded by genomic DNA. Mitochondrial and plastid transcriptomes of plants are subject to hundreds of specific C-to-U changes by RNA editing [45, 46, 47]. These changes are functionally necessary either to generate a start codon, missense or nonsense amino acid changes [46]. It has been hypothesized that the plant “editosome” contains a enzyme, which interacts to edit single or several cytidine residue sites [48]. RNA-seq data for *R*. *stricta* indicated that as many as 304 PPR genes were upregulated (S1 Table), while 22 were downregulated after 12 h of salt stress treatment (S2 Table). Most of the regulated transcripts in *R*. *stricta* act on genes in mitochondria. A similar pattern was shown in our previous study of the transcriptomes of *R*. *stricta* under heat stress, where large numbers of PPR genes were consistently upregulated starting midday up to dusk both in the apical and mature leaves [29]. Examples of the regulation of five PPR genes under heat stress across the day are shown in S4 Fig The analysis of transcriptomes of the wild barley (*Hordeum spontaneum*) across time (0, 2, 12 and 24 h) of salt (500 mM NaCl) stress treatment indicated the regulation of 65 PPR genes [26]. Unlike PPR genes in *Rhazya*, these PPR genes were downregulated at the 12 h time point, and 63 of these were also downregulated at the 2 h time point. Twenty-four barley PPRs have analogues in *Arabidopsis* (S5 Fig). Our results indicate that the expression pattern of PPR genes under salt stress in *R*. *stricta* is unique to this wild plant species. The coordinated regulation of PPR genes under salt stress indicates the possible involvement of a single transcription factor that drives their expression. Otherwise, the regulation of PPR genes may be dependent on expression levels of their target edited genes.

**Fig 4:**
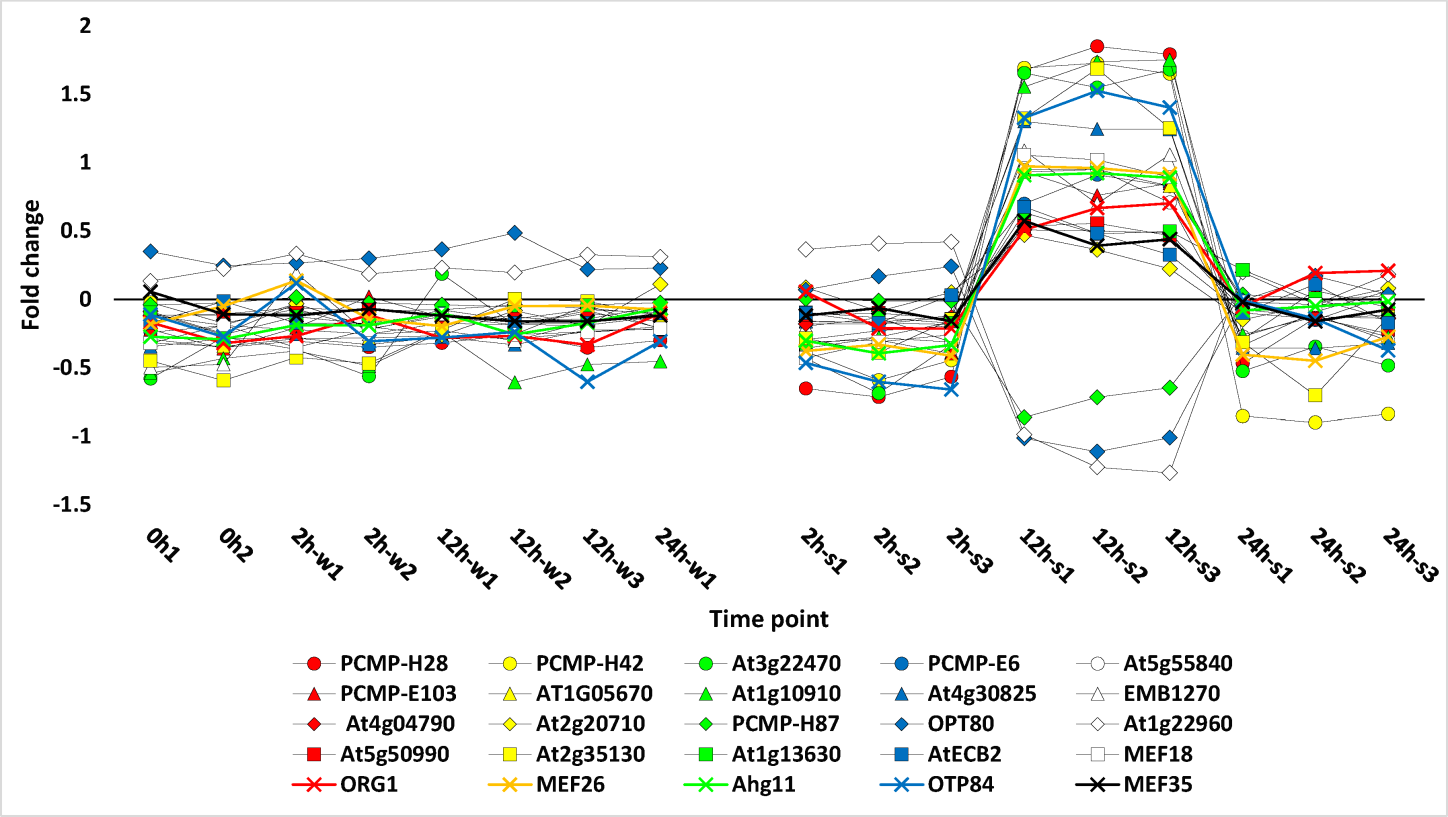
Profiles of fold change values for 25 selected *R*. *stricta* PPR leaf transcripts under water and salt stress treatments for 0, 2, 12 and 24 h. 0h1,2 (control), 2h-w1,2 (water treated for 2 h), 12h-w1,2,3 (water treated for 12 h), 24h-w1 (water treated for 24 h), 2h-s1,2,3 (salt stressed for 2 h), 12h-s1,2,3 (salt stressed for 12 h), 24h-s1,2,3 (salt stressed for 24 h). Information about the different genes is shown in S1, S2 and S5 Tables.

**Fig 5:**
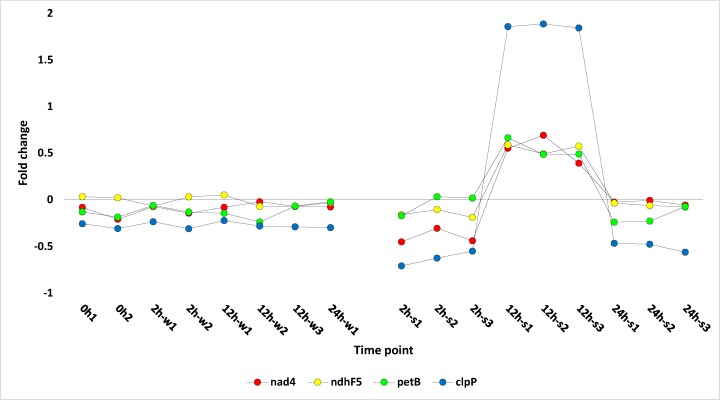
Profiles of fold change values for four selected *R*. *stricta* leaf transcripts, three of which are PPR-edited under water and salt stress treatments for 0, 2, 12 and 24 h. Three transcripts are chloroplast (*ndhF*, *petB* and *clpP*), while one is mitochondrial (*nad4*). 0h1,2 (control), 2h-w1,2 (water treated for 2 h), 12h-w1,2,3 (water treated for 12 h), 24h-w1 (water treated for 24 h), 2h-s1,2,3 (salt stressed for 2 h), 12h-s1,2,3 (salt stressed for 12 h), 24h-s1,2,3 (salt stressed for 24 h). Information about the different genes is shown in S1 and S5 Tables.

Expression patterns of 25 selected PPR transcripts and four PPR edited genes (*nad4*, *ndhF*, *petB* and *clpP*) are shown in Figs 4 and 5, respectively, and their expression levels are summarized in S5 Table. All these genes were upregulated after 12 h of salt stress except for three PPRs, (PCMP-H87, OPT80 and At1g22960), which showed downregulation at this time point. One of the four edited genes (*nad4*) is mitochondrial, while the other three are chloroplast (*ndhF*, *petB* and *clpP*). Among the selected PPR genes of *R*. *stricta* (Fig 4), seven were further studied in order to detect their function as affected by salt stress. Five PPR transcripts (*OGR1*, *Ahg11*, *MEF18*, *MEF26* and *MEF35*) are known for their action in editing different sites in the mitochondrial *nad4* transcript [19, 49, 50, 51], while two PPRs (*OTP84* and *ECB2*) are known for editing one site in the chloroplast *ndhF* transcript [49, 52, 53]. There are not enough records in the literature for the PPRs that edit *petB* gene. The PPR *CLB19* that edits the *clpP* gene of *R*. *stricta* was not regulated under the three different conditions, i.e., control, after 12 h of water treatment and after 12 h of salt stress. Transcript sequence alignment of the four genes *nad4*, *ndhF*, *petB* and *clpP* with edit sites indicated are shown in S6–S9 Figs.

The results of RNA editing pattern in mitochondrial *nad4* (NADH dehydrogenase subunit 4) transcript of *R*. *stricta* indicated the occurrence of 34 RNA edit sites to convert 33 codons (S6 Fig). Edits in different sites of *nad4* transcripts mostly involved the conversion of serine to either phenylalanine or leucine. One edit site (codon 473) involved the conversion of histidine into tyrosine. One incidence of double editing took place at site 146, where proline codon (CCC) was converted to phenylalanine codon (TTC). As indicated earlier, only five PPRs known to edit the *nad4* gene were regulated in *R*. *stricta*. These PPRs edit six sites in *R*. *stricta*, e.g., ORG1 edits two codons (no. 139 and 145), while PPRs MEF26, Agh11, MEF18 and MEF35 edit one codon each (no. 56, 126, 452 or 458, respectively). Many other PPRs are assigned for editing the transcript of this gene, but none of them were either identified or regulated after 12 h of salt stress in *R*. *stricta*.

The chloroplast *ndhF* transcript in *R*. *stricta* has only one edit site in codon 97 for the conversion of serine to leucine (S7 Fig). Previous reports indicated that PPRs *OTP84* [52] and *ECB2* [53] act on the same edit site in *Arabidopsis*. As the two PPRs were among the upregulated transcripts after 12 h of salt stress, we assume that they might compete for editing this site in *R*. *stricta*. Cao *et al*. [53] indicated that PPRs *ECB2* and *RARE* also edit the same site in the chloroplast *accD* transcript in *Arabidopsis*. This indicates that the binding sites of PPRs *OTP84* and *ECB2*, on one hand, or PPRs *ECB2* and *RARE*, on the other hand, might have similar affinities.

S8 Fig indicates the occurrence of two edit sites of *petB* transcript in *R*. *stricta* at codons 4 and 204. The second site of the transcript was reported in tobacco [54] and maize [55], while the first was not previously reported in the literature. RNA editing of the second site resulted in the conversion of proline to leucine, while the first site resulted in no change in the amino acid valine as the codon was changed in the third degenerate nucleotide (i.e., GTC into GTT). There is no explanation for the consistent change of this degenerate nucleotide (+12 of the transcript) across the three plant growth conditions as *R*. *stricta* showed no codon bias or preference in the third degenerate nucleotide of the different valine codons of this transcript (S8 Fig). There is no conclusive information with regard to the PPRs inducing RNA editing in these two sites except that Meierhoff *et al*. [14] indicated the involvement of the PPR HCF152 in processing *psbB-psbT*-*psbH-petB-petD* RNAs in *Arabidopsis*. Miyamoto *et al*. [56] only indicated that the PPR that edits *petB* transcript in tobacco and pea chloroplasts has a molecular mass of 70 kDa.

The gene encoding ATP-dependent Clp protease proteolytic subunit (*clpP*) was also upregulated after 12 h of salt stress (Fig 5) in *R*. *stricta*, while its PPR gene (*CLB19* [57]) was not regulated at this time point. This PPR likely edits the codon 187 of *clpP* transcript in *Arabidopsis* to convert histidine to tyrosine [57]. The *clpP* gene in *R*. *stricta* encodes a conserved tyrosine codon at this site (S9 Fig), thus the transcript requires no editing. This result explains the lack of regulation of its PPR gene under salt stress although its cognate target gene (*clpP*) is regulated.

There is no explanation for the high expression levels of the edited genes *nad4*, *ndhF petB* and *clpP* 12 h after salt stress as no previous reports indicated their direct involvement in salt responses. Available information indicates that NADH dehydrogenase or complex I (CI) is a main entry point for electrons into the mitochondrial respiratory chain and represents the most complicated portion of the respiratory system due to the large number of subunits in the enzyme. The *nad4* gene is among those governing the mitochondrial respiratory chain. Maize plants deficient in the *nad4* gene failed to assemble CI and have pale-green striping on the leaves and die during kernel development [58]. Also, the available information indicates that the thylakoid Ndh complex is analogous to the mitochondrial CI as it catalyzes the transfer of electrons from NADH to plastoquinone [59]. The chloroplast *ndh* genes encode polypeptides of the Ndh complex, and *ndhF* helps stimulate the expression of the other *ndh* genes [60]. As for the *petB*, it is within the *psbB-psbT-psbH-petB-petD* operon that encodes cytochrome *b*_6_ and subunit IV of the cytochrome b_6_*/*f complex (plastoquinol—plastocyanin reductase; EC:1.10.99.1). The latter enzyme catalyzes the transfer of electrons from plastoquinol to plastocyanin or mediates the transfer of electrons from PSII to PSI towards the production of ATP [61]. Previous reports indicated that the proteolytic enzymes generally play a fundamental role in chloroplast development and function [62]. Among these enzymes is the ATP-dependent Clp protease encoded by *clpP* gene. The enzyme facilitates the continuous rate of protein turnover during growth and differentiation and acts in removing inactive and misfolded polypeptides, especially during stress. The enzyme is also involved in amino acid recycling and the regulation of key enzymes and regulatory proteins [63]. Lack of editing of the gene encoding ATP-dependent Clp protease in the knockout mutant of *CLB19* gene of *Arabidopsis* prevented the change of the histidine at codon 187, thus impairing chloroplast development and early seedling lethality under greenhouse conditions [57]. As indicated earlier, the *clpP* transcript of *R*. *stricta* requires no editing at this site as the site encodes the conserved tyrosine.

## Conclusions

In general, we can conclude that the wild plant species *R*. *stricta* harbors several mechanisms for avoiding protein misfolding, aggregation or denaturation and the efficient production of several key secondary metabolites and ROS that helps the plant to withstand salt stress. Tetrapyrrole production in *R*. *stricta* adds to its ability to maintain normal rates of photosynthesis, respiration and plant growth under salt stress. There is no conclusive data available for the edited genes *nad4*, *ndhF*, *petB* and *clpP* regarding their involvement in salt stress responses in *R*. *stricta*. In conclusion, the results indicated the upregulation of a large number of transcripts belonging to PPR gene family at 12 h time point of salt stress, while their editing patterns were unchanged compared to the their editing patterns at 0 h time point or under water treatment at 12 h time point. We speculate that the high expression levels of PPRs after 12 h of salt stress may be dependent on expression levels of their target edited genes.

## Supporting information

S1 Fig Clusters (236) of assembled transcripts of *R*. *stricta* leaves under water (W) and salt (S) (500 mM NaCl) treatments for 0, 2, 12 and 24 h. Grey lines indicate expression patterns of individual transcripts in a given cluster. Blue lines indicate overall expression pattern across different transcripts of a given cluster. 0h1,2 (control), 2h-w1,2 (water treated for 2 h), 12h-w1,2,3 (water treated for 12 h), 24h-w1 (water treated for 24 h), 2h-s1,2,3 (salt stressed for 2 h), 12h-s1,2,3 (salt stressed for 12 h), 24h-s1,2,3 (salt stressed for 24 h).
(PDF)

S2 Fig Semi-quantitative RT-PCR validation results utilizing selected PPR genes of *R*. *stricta*. (a) sqRT-PCR profiles of 10 upregulated PPR genes after 12 h of salt stress, (b) fold change profiles resulting from RNA-Seq analysis of the same PPR genes after 12 h of salt stress, and (c) the “*actin*” gene used as the unregulated house keeping gene. 0h1,2 (control), 2h-s1,2 (salt stressed for 2 h), 12h-s1,2 (salt stressed for 12 h), 24h-s1,2 (salt stressed for 24 h). Further information about the different genes is shown in S1 Table.
(DOCX)

S3 Fig Enzymes in the porphyrin and chlorophyll metabolism pathway in leaves activated under salt (500 mM NaCl) treatment for 12 h in *R*. *stricta*. Colored rectangular boxes indicate the activated enzymes in the pathway. Highly activated enzymes at 12 h time point of salt (500 mM NaCl) stress are shown in colored boxes, while the enzymes with unchanged activation rates are shown in uncolored boxes. Different box colors in the pathway indicates different highly activated enzymes. Non-colored boxes indicate no change in enzyme activity at 12 h time point compared to 0 h time point. Red arrows indicate four enzymes that are expected to result in a higher rate under salt stress due to the high activation rates; orange arrow indicates the enzyme Fe-chelatase; green arrow indicates the enzyame Mg-chelatase.
(DOCX)

S4 Fig Profiles of fold change values for the five genes encoding PPRs from apical (A1-L4) and mature (A5-L8) *R*. *stricta* leaves under heat stress at five time points of the day, e.g., morning (A, 07:10), midday (F, 13:25; G, 14:05 & H, 14:30) and dusk (L, 18:27). Information about the different genes is shown in S1 and S2 Tables [29].
(DOCX)

S5 Fig Profiles of fold change values for 24 selected wild barley (*H*. *spontaneum*) PPR leaf transcripts under water and salt stress treatments for 0, 2, 12 and 24 h. 0h-1,2 (control), 2h-1,2,3 (salt stressed for 2 h), 12h-1,2,3 (salt stressed for 12 h), 24h-1,2,3 (salt stressed for 24 h). Information about the different genes is available at The *Arabidopsis* Information Resource (TAIR, https://www.arabidopsis.org/).
(DOCX)

S6 Fig Sequence alignment of the *nad4* transcripts (1488 nt) and their deduced amino acids generated under control condition, 12 h water treatment and 12 h salt stress in leaves of *R*. *stricta* showing 34 edit sites. Five PPRs are known to edit six sites in this transcript, e.g., ORG1 edits two codons (no. 139 and 145), while PPRs MEF26, Agh11, MEF18 and MEF35 edit one codon each (no. 56, 126, 452 or 458, respectively). The six edit sites of codons no. 56, 126, 139, 145, 452 and 458 resulted in the conversion of arginine (CGG) to tryptophan (TGG), arginine (CGT) to cysteine (TGT), proline (CCT) to leucine (CTT), leucine (CTT) to phenylalanine (TTT), proline (CCA) to leucine (CTA) and serine (TCC) to phenylalanine (TTC), respectively. Red rectangles indicate the different edit sites of the known five PPRs, while blue rectangles indicate the other edit sites in this transcript. The letters in the figure indicate the abbreviations of different amino acids.
(DOCX)

S7 Fig Sequence alignment of the *ndhF* transcripts (2226 nt) and their deduced amino acids generated under control condition, 12 h water treatment and 12 h salt stress in leaves of *R*. *stricta* showing only one edit site (inside the red rectangle) in codon no. 97. Editing resulted in the conversion of serine (S) codon (TCA) to leucine (L) codon (TTA). Two PPRs, OTP84 and ECB2, compete to edit this site in the transcript. The letters in the figure indicate the abbreviations of different amino acids.
(DOCX)

S8 Fig Sequence alignment of the *petB* transcripts (648 nt) and their deduced amino acids generated under control condition, 12 h water treatment and 12 h salt stress in leaves of *R*. *stricta* showing two edit sites (inside the red squares) in codons no. 4 and 204. Editing of the first site (GTC to GTT) resulted in no change in the amino acid valine (V), while the second edit (CCA to CTA) resulted in the conversion of proline (P) to leucine (L). No PPRs are known for editing either site. The letters in the figure indicate the abbreviations of different amino acids.
(DOCX)

S9 Fig Sequence alignment of the *clpP* transcripts (591 nt) and their deduced amino acids generated under control condition, 12 h water treatment and 12 h salt stress in leaves of *R*. *stricta* showing no edit sites. One PPR, CLB19, is known to edit one site in this transcript to convert histidine to tyrosine in codon number 187 (shown inside the red square). This codon (UAU) in *R*. *stricta* normally encodes a conserved tyrosine (Y), hence, requires no editing. The letters in the figure indicate the abbreviations of different amino acids.
(DOCX)

S1 Table Cluster analysis indicating fold change values of assembled transcripts upregulated at 12 h time point in *R*. *stricta* under salt (500 mM NaCl) stress. 0h1,2 (control), 2h-w1,2 (water treated for 2 h), 12h-w1,2,3 (water treated for 12 h), 24h-w1 (water treated for 24 h), 2h-s1,2,3 (salt stressed for 2 h), 12h-s1,2,3 (salt stressed for 12 h), 24h-s1,2,3 (salt stressed for 24 h).
(XLSX)

S2 Table Cluster analysis indicating fold change values of assembled transcripts downregulated at 12 h time point in *R*. *stricta* under salt (500 mM NaCl) stress. 0h1,2 (control), 2h-w1,2 (water treated for 2 h), 12h-w1,2,3 (water treated for 12 h), 24h-w1 (water treated for 24 h), 2h-s1,2,3 (salt stressed for 2 h), 12h-s1,2,3 (salt stressed for 12 h), 24h-s1,2,3 (salt stressed for 24 h).
(XLSX)

S3 Table Primer sequences along with the annealing temperature and expected amplicon sizes (bp) to be utilized in in validating RNA-Seq dataset of *R*. *stricta* via semi-quantitative RT-PCR. (XLSX)

S4 Table Comparative differential expression of genes of *R*. *stricta* transcriptomes under salt (500 mM NaCl) treatments for 0, 2, 12 and 24 h. Blue box = upregulation, orange box = downregulation.
(DOCX)

S5 Table **Description of 25 selected PPR genes (A) and four PPR-edited (B) genes under water (w) treatment and salt (s) stress for 0, 2, 12 and 24 h in *R*. *stricta*.** All PPR genes were upregulated except for *PCMP-H87*, *OPT80* and *At1g22960* that were downregulated. This data was extracted from S1 and S2 Tables. 0h1,2 (control), 2h-w1,2 (water treated for 2 h), 12h-w1,2,3 (water treated for 12 h), 24h-w1 (water treated for 24 h), 2h-s1,2,3 (salt stressed for 2 h), 12h-s1,2,3 (salt stressed for 12 h), 24h-s1,2,3 (salt stressed for 24 h).
(XLSX)

## References

[1] Xiong L, Zhu JK. Abiotic stress signal transduction in plants: molecular and genetic perspectives. Physiol Plant. 2001; 112:152–166. 11454221 10.1034/j.1399-3054.2001.1120202.x

[2] Zhu JK Salt and drought stress signal transduction in plants. Ann Rev Plant Biol. 2002; 53:247–273. 12221975 10.1146/annurev.arplant.53.091401.143329 PMC3128348

[3] Zhu JK Regulation of ion homeostasis under salt stress. Curr Opin Plant Biol. 2003; 6:441–445. 12972044 10.1016/s1369-5266(03)00085-2

[4] Adie BAT, Pérez-Pérez J, Pérez-Pérez MM, Godoy M, Sunchez-Serrano JJ, Schmelz EA, et al ABA is an essential signal for plant resistance to pathogens affecting JA biosynthesis and the activation of defenses in *Arabidopsis*. Plant Cell 2007; 19:1665–1681. doi: 10.1105/tpc.106.048041 17513501 10.1105/tpc.106.048041 PMC1913739

[5] Cutler SR, Rodriguez PL, Finkelstein RR, Abrams SR. Abscisic acid: emergence of a core signaling network. Ann Rev Plant Biol. 2010; 61:651–679. 20192755 10.1146/annurev-arplant-042809-112122

[6] Fujita Y, Fujita M, Shinozaki K, Yamaguchi-Shinozaki K. ABA-mediated transcriptional regulation in response to osmotic stress in plants. J Plant Res 2011; 124:509–525. doi: 10.1007/s10265-011-0412-3 21416314 10.1007/s10265-011-0412-3

[7] Chaves MM, Flexas J, Pinheiro C. Photosynthesis under drought and salt stress: regulation mechanisms from whole plant to cell. Ann Bot. 2009; 103:551–560. doi: 10.1093/aob/mcn125 18662937 10.1093/aob/mcn125 PMC2707345

[8] Turan S, Tripathy BC. Salt-stress induced modulation of chlorophyll biosynthesis during de-etiolation of rice seedlings. Physiol Plant. 2015; 153:477–491. doi: 10.1111/ppl.12250 25132047 10.1111/ppl.12250

[9] Cornah JE, Terry MJ, Smith AG. Green or red: what stops the traffic in the tetrapyrrole pathway? Trends in Plant Sci. 2003; 8:224–230. 12758040 10.1016/S1360-1385(03)00064-5

[10] Small ID, Peeters N. The PPR motif—A TPR-related motif prevalent in plant organellar proteins. Trends Biochem Sci. 2000; 25:46–47. 10664580 10.1016/s0968-0004(99)01520-0

[11] Lurin C, Andres C, Aubourg S, Bellaoui M, Bitton F, Bruyère C, et al Genome-wide analysis of *Arabidopsis* pentatricopeptide repeat proteins reveals their essential role in organelle biogenesis. Plant Cell. 2004; 16:2089–2103. doi: 10.1105/tpc.104.022236 15269332 10.1105/tpc.104.022236 PMC519200

[12] Rivals E, Bruyere C, Toffano-Nioche C, Lecharny A. Formation of the *Arabidopsis* pentatricopeptide repeat family. Plant Physiol. 2006; 141:825–839. doi: 10.1104/pp.106.077826 16825340 10.1104/pp.106.077826 PMC1489915

[13] Schmitz-Linneweber C, Small I. Pentatricopeptide repeat proteins: a socket set for organelle gene expression. Trends Plant Sci. 2008; 13:663–670. doi: 10.1016/j.tplants.2008.10.001 19004664 10.1016/j.tplants.2008.10.001

[14] Meierhoff K, Felder S, Nakamura T, Bechtold N, Schuster G. HCF152, an *Arabidopsis* RNA binding pentatricopeptide repeat protein involved in the processing of chloroplast *psbB*-*psbT-psbH*-*petB*-*petD* RNAs. Plant Cell. 2003; 15:1480–1495. doi: 10.1105/tpc.010397 12782738 10.1105/tpc.010397 PMC156381

[15] Williams PM, Barkan A. A chloroplast-localized PPR protein required for plastid ribosome accumulation. Plant J. 2003; 36:675–686. 14617068 10.1046/j.1365-313x.2003.01915.x

[16] Zsigmond L, Rigo G, Szarka A, Székely G, Ötvos K, Darula Z, et al *Arabidopsis* PPR40 connects abiotic stress responses to mitochondrial electron transport. Plant Physiol. 2008; 146, 1721–1737. 18305213 10.1104/pp.107.111260 PMC2287346

[17] Liu Y, He J, Chen Z, Ren X, Hong X, Gong Z. *ABA overlysensitive 5* (*ABO5*), encoding a pentatricopeptide repeat protein required for *cis*-splicing of mitochondrial *nad2* intron 3, is involved in the abscisic acid response in *Arabidopsis*. Plant J. 2010; 63:749–765. doi: 10.1111/j.1365-313X.2010.04280.x 20561255 10.1111/j.1365-313X.2010.04280.x

[18] Laluk K, Abuqamar S, Mengiste T. The *Arabidopsis* mitochondria-localized pentatricopeptide repeat protein PGN functions in defense against necrotrophic fungi and abiotic stress tolerance. Plant Physiol. 2011; 156:2053–2068. doi: 10.1104/pp.111.177501 21653783 10.1104/pp.111.177501 PMC3149943

[19] Murayama M, Hayashi S, Nishimura N, Ishide M, Kobayashi K, Yagi Y et al Isolation of *Arabidopsis ahg11*, a weak ABA hypersensitive mutant defective in *nad4* RNA editing. J Experim Bot. 2012; 63:5301–5310. 10.1093/jxb/ers188 PMC3430999 22821940

[20] Yuan H, Liu D. Functional disruption of the pentatricopeptide protein SLG1 affects mitochondrial RNA editing, plant development, and responses to abiotic stresses in *Arabidopsis*. Plant J. 2012; 70:432–444. 22248025 10.1111/j.1365-313X.2011.04883.x

[21] Lv HX, Huang C, Guo GQ, Yang ZN. Roles of the nuclear encoded chloroplast SMR domain-containing PPR protein SVR7 in photosynthesis and oxidative stress tolerance in *Arabidopsis*. J Plant Biol. 2014; 57:291–301.

[22] Zhu Q, Dugardeyn J, Zhang C, Mühlenbock P, Eastmond PJ, Valcke R, et al The *Arabidopsis thaliana* RNA editing factor SLO2, which affects the mitochondrial electron transport chain, participates in multiple stress and hormone responses. Mol Plant. 2014; 7:290–310. 23990142 10.1093/mp/sst102

[23] Jiang S-C,·Mei C, Liang S, Yu YT, Lu K, Wu Z, Wang XF, Zhang DP. Crucial roles of the pentatricopeptide repeat protein SOAR1 in *Arabidopsis* response to drought, salt and cold stresses. Plant Mol Biol. 2015; 88:369–385. doi: 10.1007/s11103-015-0327-9 26093896 10.1007/s11103-015-0327-9 PMC4486114

[24] Hammani K, des Francs-Small CC, Takenaka M, Tanz SK, Okuda K, Shikanai T, Brennicke A, Small I. The pentatricopeptide repeat protein OTP87 is essential for RNA editing of *nad7* and *atp1* transcripts in *Arabidopsis* mitochondria. J Biol Chem. 2011; 286:21361–21371. doi: 10.1074/jbc.M111.230516 21504904 10.1074/jbc.M111.230516 PMC3122196

[25] Sabir JSM, Jansen RK, Arasappan D, Calderon V, Noutahi E, Zheng C, et al The nuclear genome of *Rhazya stricta* and the evolution of alkaloid diversity in a medically relevant clade of Apocynaceae. Sci Rep. 2016; 6:33782 doi: 10.1038/srep33782 27653669 10.1038/srep33782 PMC5031960

[26] Bahieldin A, Atef A, Sabir JSM, Gadalla NO, Edris S, Alzohairy AM, et al RNA-Seq analysis of the wild barley (*H*. *spontaneum*) leaf transcriptome under salt stress. C R Biologies. 2015; 338:285–297. doi: 10.1016/j.crvi.2015.03.010 25882349 10.1016/j.crvi.2015.03.010

[27] Zhang J, Ruhlman TA, Mower JP, Jansen RK. Comparative analyses of two Geraniaceae transcriptomes using next-generation sequencing. BMC Plant Biol. 2013; 13:228 doi: 10.1186/1471-2229-13-228 24373163 10.1186/1471-2229-13-228 PMC3880972

[28] Brown JA, Sherlock G, Myers CL, Burrows NM, Deng C, Wu HI, et al Global analysis of gene function in yeast by quantitative phenotypic profiling. Mol Syst Biol. 2006; 2:2006.0001 10.1038/msb4100043 PMC1681475 16738548

[29] Obaid AY, Sabir JSM, Atef A, Liu X, Edris S, El-Domyati FM, et al Analysis of transcriptional response to heat stress in *Rhazya stricta*. BMC Plant Biol. 2016; 16:252 doi: 10.1186/s12870-016-0938-6 27842501 10.1186/s12870-016-0938-6 PMC5109689

[30] Rosa M, Prado C, Podazza G, Interdonato R, González JA, Hilal M et al Soluble sugars—Metabolism, sensing and abiotic stress. A complex network in the life of plants. Plant Signal Behav. 2009; 4:388–393. doi: 10.4161/psb.4.5.8294 19816104 10.4161/psb.4.5.8294 PMC2676748

[31] Fini A, Brunetti C, Ferdinando MD, Ferrini F, Tattini M. Stress-induced flavonoid biosynthesis and the antioxidant machinery of plants. Plant Signal Behav. 2011; 6:709–711. doi: 10.4161/psb.6.5.15069 21448007 10.4161/psb.6.5.15069 PMC3172844

[32] Wu D, Cai S, Chen M, Ye L, Chen Z, Zhang H, et al Tissue metabolic responses to salt stress in wild and cultivated barley. PLoS One. 2013; 8:e55431 doi: 10.1371/journal.pone.0055431 23383190 10.1371/journal.pone.0055431 PMC3561194

[33] Khan NA, Khan MIR, Asgher M, Fatma M, Masood A, Syeed S. Salinity tolerance in plants: Revisiting the role of sulfur metabolites. J Plant Biochem Physiol. 2014; 2:120.

[34] Goyal E, Amit SK, Singh RS, Mahato AK, Chand S, Kanika K. Transcriptome profiling of the salt-stress response in Triticum aestivum cv. Kharchia Local. Sci Rep. 2016; 6:27752 doi: 10.1038/srep27752 27293111 10.1038/srep27752 PMC4904219

[35] Laufen T, Mayer MP, Beisel C, Klostermeier D, Moor A, Reinstein J, et al Mechanism of regulation of hsp70 chaperones by DnaJ cochaperones. Proc Natl Acad Sci USA. 1999; 96:5452–5457. 10318904 10.1073/pnas.96.10.5452 PMC21880

[36] Liberek K, Marszalek J, Ang D, Georgopoulos C, Żylicz M. *Escherichia coli* DnaJ and GrpE heat shock proteins jointly stimulate ATPase activity of DnaK. Proc Natl Acad Sci USA. 1991; 88:2874–2878. 1826368 10.1073/pnas.88.7.2874 PMC51342

[37] Vierling E. The roles of heat shock proteins in plants. Ann Rev Plant Physiol Plant Mol Biol. 1991; 42:579–620.

[38] Blanco-Herrera F, Moreno AA, Tapia R, Reyes F, Araya M, D'Alessio C, et al The UDP-glucose: glycoprotein glucosyltransferase (UGGT), a key enzyme in ER quality control, plays a significant role in plant growth as well as biotic and abiotic stress in *Arabidopsis thaliana*. BMC Plant Biol. 2015; 15:127 doi: 10.1186/s12870-015-0525-2 26017403 10.1186/s12870-015-0525-2 PMC4465474

[39] Wahid A, Ghazanfar A. Possible involvement of some secondary metabolites in salt tolerance of sugarcane. J Plant Physiol. 2006; 163:723–730. doi: 10.1016/j.jplph.2005.07.007 16616583 10.1016/j.jplph.2005.07.007

[40] Cheng C, Xu X, Gao M, Li J, Guo C, Song J, et al Genome-wide analysis of respiratory burst oxidase homologs in grape (*Vitis vinifera* L.). Int J Mol Sci. 2013; 14:24169–24186. doi: 10.3390/ijms141224169 24351809 10.3390/ijms141224169 PMC3876103

[41] Apel K, Hirt H. Reactive oxygen species: Metabolism, oxidative stress, and signal transduction. Ann Rev Plant Biol. 2004; 55:373–399. 15377225 10.1146/annurev.arplant.55.031903.141701

[42] Grimm B. Novel insights in the control of tetrapyrrole metabolism of higher plants. Curr Opin Plant Biol. 1998; 1:245–250. 10066589 10.1016/s1369-5266(98)80112-x

[43] Papenbrock J, Grimm B. Regulatory network of tetrapyrrole biosynthesis–studies of intracellular signalling involved in metabolic and developmental control of plastids. Planta. 2001; 213:667–681. 11678270 10.1007/s004250100593

[44] Juhanningmeier U, Howell SH. Regulation of the light harvesting chlorophyll-binding protein in *Chlamydomonas reinhardtii*. J Biol Chem. 1984; 259:13541–13549. 6386816

[45] Kotera E, Tasaka M, Shikanai T. A pentatricopeptide repeat protein is essential for RNA editing in chloroplasts. Nature. 2005; 433:326–330. 15662426 10.1038/nature03229

[46] Takenaka M, Verbitskiy D, van der Merwe JA, Zehrmann A, Brennicke A. The process of RNA editing in plant mitochondria. Mitochondrion. 2008; 8:35–46. 18326075 10.1016/j.mito.2007.09.004

[47] Takenaka M, Zehrmann A, Verbitskiy D, Hartel B, Brennicke A. RNA editing in plants and its evolution. Ann Revi Genet. 2013; 47:335–352. 10.1146/annurev-genet-111212-133519 24274753

[48] Hammani K, Gobert A, Hleibieh K, Choulier L, Small I, Geige P. An *Arabidopsis* dual-localized pentatricopeptide repeat protein interacts with nuclear proteins involved in gene expression regulation. Plant Cell. 2011; 23:730–740. doi: 10.1105/tpc.110.081638 21297037 10.1105/tpc.110.081638 PMC3077779

[49] Fujii S, Small I. The evolution of RNA editing and pentatricopeptide repeat genes. New Phytol. 2011; 191:37–47. doi: 10.1111/j.1469-8137.2011.03746.x 21557747 10.1111/j.1469-8137.2011.03746.x

[50] Arenas-M A, Zehrmann A, Moreno S, Takenaka M, Jordana X. The pentatricopeptide repeat protein MEF26 participates in RNA editing in mitochondrial cox3 and nad4 transcripts. Mitochondrion. 2014; 19:126–134. 25173472 10.1016/j.mito.2014.08.006

[51] Brehme N, Bayer-Császár E, Glass F, Takenaka M. The DYW subgroup PPR protein MEF35 targets RNA editing sites in the mitochondrial *rpl16*, *nad4* and *cob* mRNAs in *Arabidopsis thaliana*. PLoS ONE. 2015; 10:e0140680 doi: 10.1371/journal.pone.0140680 26470017 10.1371/journal.pone.0140680 PMC4607164

[52] Hammani K, Okuda K, Tanz SK, Chateigner-Boutin A-L, Shikanai T, Small I. A study of new *Arabidopsis* chloroplast RNA editing mutants reveals general features of editing factors and their target sites. Plant Cell. 2009; 21:3686–3699. doi: 10.1105/tpc.109.071472 19934379 10.1105/tpc.109.071472 PMC2798323

[53] Cao Z-L, Yu Q-B, Sun Y1, Lu Y, Cui Y-L, Yang ZN. A point mutation in the pentatricopeptide repeat motif of the AtECB2 protein causes delayed chloroplast development. J Integr Plant Biol. 2011; 53:258–269. doi: 10.1111/j.1744-7909.2011.01030.x 21294841 10.1111/j.1744-7909.2011.01030.x

[54] Hirose T, Wakasugi T, Sugiura M, Kössel H. RNA editing of tobacco *petB* mRNAs occurs both in chloroplasts and non-photosynthetic proplastids. Plant Mol Biol. 1994; 26:509–513. 7948899 10.1007/BF00039562

[55] Freyer R, Hoch B, Neckermann K, Maier RM, Kössel H. RNA editing in maize chloroplast is a processing step independent of splicing and cleavage to monocistronic mRNAs. Plant J. 1993; 4:621–629. 8252066 10.1046/j.1365-313x.1993.04040621.x

[56] Miyamoto T, Obokata J, Sugiura M. Recognition of RNA editing sites is directed by unique proteins in chloroplasts: biochemical identification of *cis*-acting elements and *trans*-acting factors involved in RNA editing in tobacco and pea chloroplasts. Mol Cell Biol. 2002; 22:6726–6734. doi: 10.1128/MCB.22.19.6726-6734.2002 12215530 10.1128/MCB.22.19.6726-6734.2002 PMC134032

[57] Chateigner-Boutin A-L, Ramos-Vega M, Guevara-Garcí A, Andrés C, de la Luz Gutiérrez-Nava M, Cantero A, et al CLB19, a pentatricopeptide repeat protein required for editing of *rpoA* and *clpP* chloroplast transcripts. Plant J. 2008; 56:590–602. doi: 10.1111/j.1365-313X.2008.03634.x 18657233 10.1111/j.1365-313X.2008.03634.x

[58] Karpova OV, Newton KJ. A partially assembled complex I in NAD4-deficient mitochondria of maize. Plant J. 1999; 17:511–521.

[59] Rumeau D, Becuwe-Linka N, Beyly A, Louwagie M, Garin J, Peltier G. New subunits NDH-M, -N, and -O, encoded by nuclear genes, are essential for plastid Ndh complex functioning in higher plants. Plant Cell. 2005; 17:219–232. doi: 10.1105/tpc.104.028282 15608332 10.1105/tpc.104.028282 PMC544500

[60] Martín M, Funk HT, Serrot PH, Poltnigg P, Sabater B. Functional characterization of the thylakoid Ndh complex phosphorylation by site-directed mutations in the *ndhF* gene. Biochim Biophys Acta. 2009; 1787:920–928. 19272354 10.1016/j.bbabio.2009.03.001

[61] Hasan SS, Yamashita E, Baniulis D, Cramer WA. Quinone-dependent proton transfer pathways in the photosynthetic cytochrome b6f complex. Proc Natl Acad Sci USA. 2013; 110:4297–4302 doi: 10.1073/pnas.1222248110 23440205 10.1073/pnas.1222248110 PMC3600468

[62] Sjögren LLE, Stanne TM, Zheng B, Sutinen S, Clarke AK. Structural and functional insights into the chloroplast ATP-dependent Clp protease in *Arabidopsis*. Plant Cell. 2006; 18:2635–2649. doi: 10.1105/tpc.106.044594 16980539 10.1105/tpc.106.044594 PMC1626633

[63] Vierstra RD. Proteolysis in plants: Mechanisms and functions. Plant Mol Biol. 1993; 32:275–302. 10.1007/BF00039386 8980483

