# Supplementary material for: Transcriptomic analysis of salt stress responsive genes in Rhazya stricta
Source: PLoS One. 2017 May 16;12(5):e0177589. doi: 10.1371/journal.pone.0177589 (PMC5433744; doi:10.1371/journal.pone.0177589)
Supplement: S1 Fig — Grey lines indicate expression patterns of individual transcripts in a given cluster. Blue lines indicate overall expression pattern across different transcripts of a given cluster. 0h1,2 (control), 2h-w1,2 (water treated for 2 h), 12h-w1,2,3 (water treated for 12 h), 24h-w1 (water treated for 24 h), 2h-s1,2,3 (salt stressed for 2 h), 12h-s1,2,3 (salt stressed for 12 h), 24h-s1,2,3 (salt stressed for 24 h). (PDF) [file pone.0177589.s001.pdf]

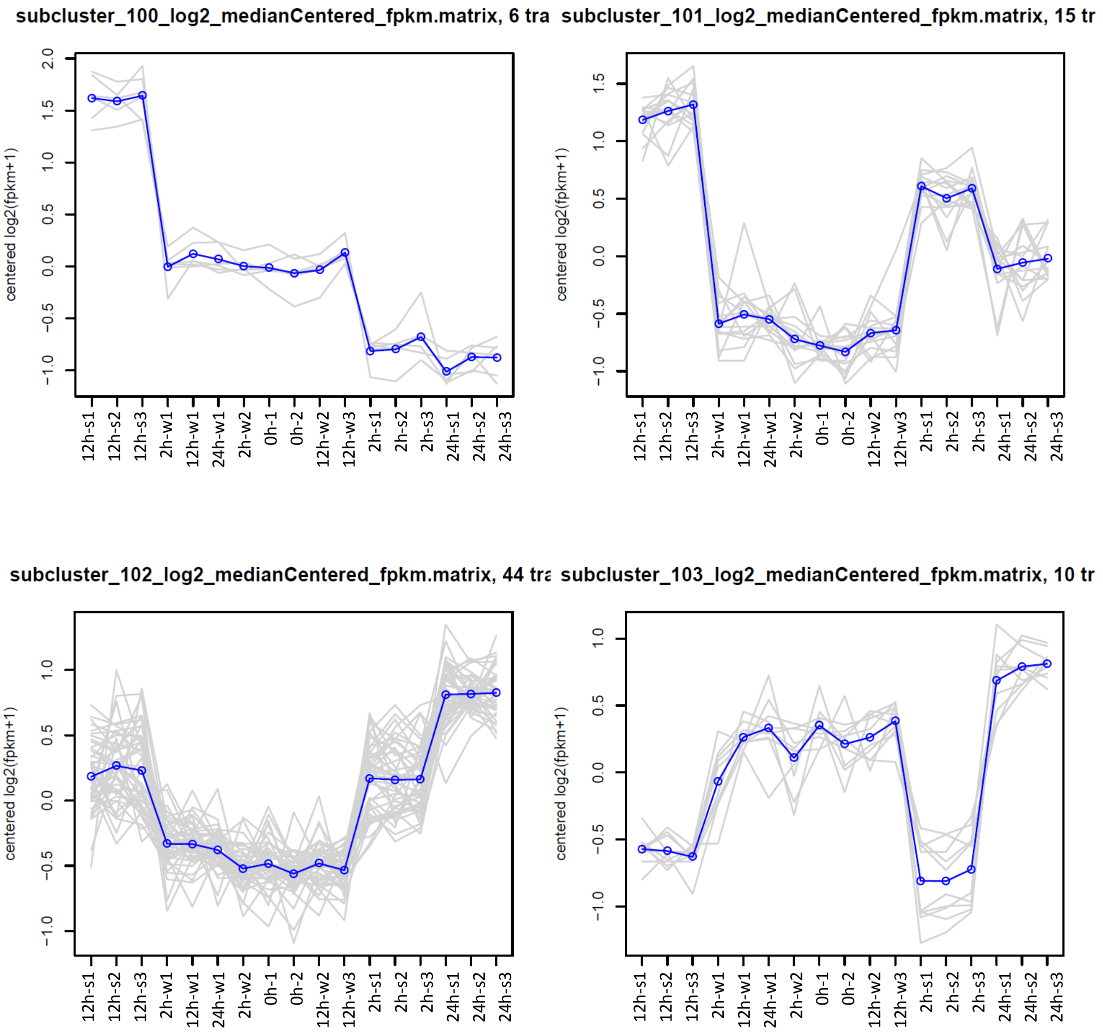
subcluster_100_log2_medianCentered_fpkm.matrix, 6 tra
subcluster_101_log2_medianCentered_fpkm.matrix, 15 tr
subcluster_102_log2_medianCentered_fpkm.matrix, 44 tra
subcluster_103_log2_medianCentered_fpkm.matrix, 10 tr
centered log2(fpkm+1)
12h-s1
12h-s2
12h-s3
2h-w1
12h-w1
24h-w1
2h-w2
0h-1
0h-2
12h-w2
12h-w3
2h-s1
2h-s2
2h-s3
24h-s1
24h-s2
24h-s3

Fig. S1.

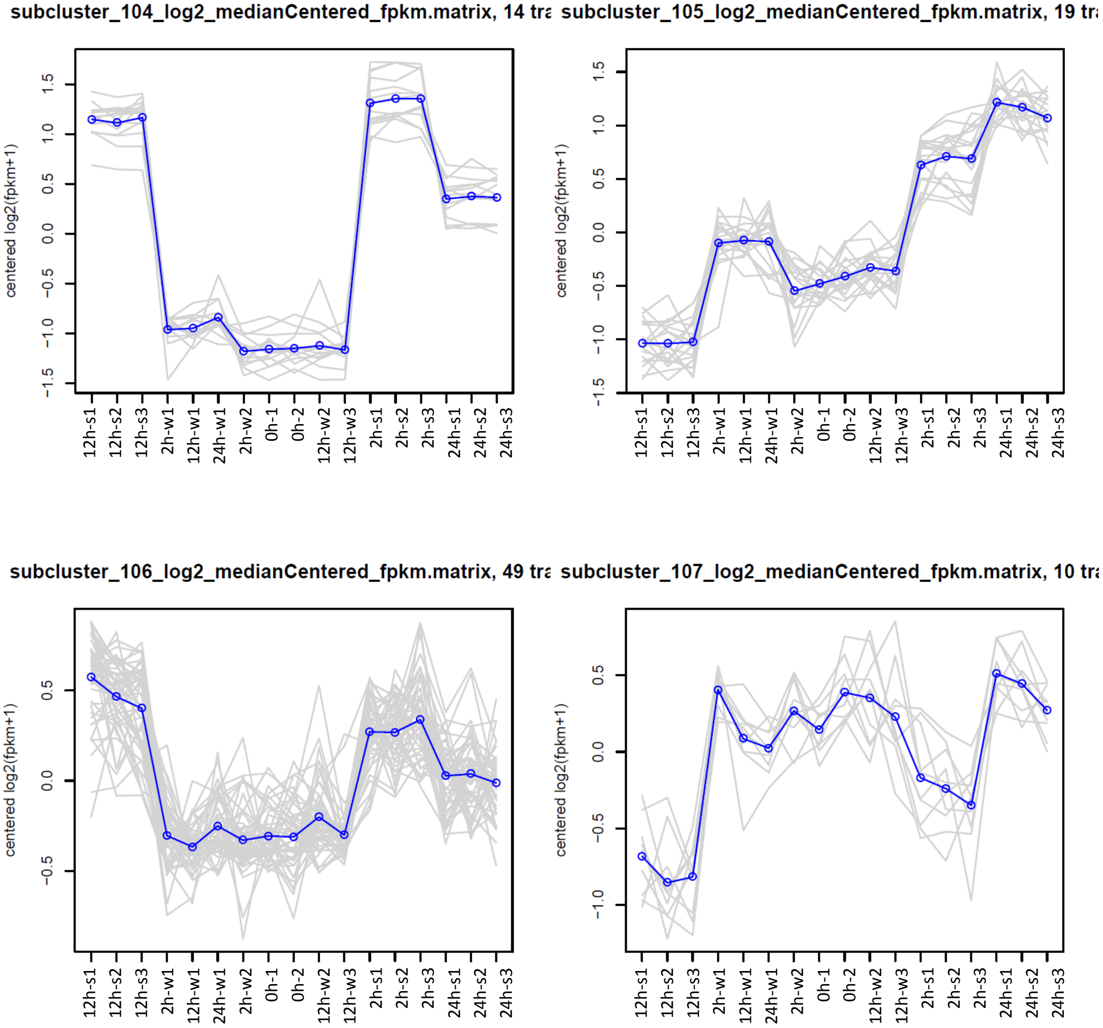
subcluster_104_log2_medianCentered_fpkm.matrix, 14 tra
subcluster_105_log2_medianCentered_fpkm.matrix, 19 tra
subcluster_106_log2_medianCentered_fpkm.matrix, 49 tra
subcluster_107_log2_medianCentered_fpkm.matrix, 10 tra
centered log2(fpkm+1)
12h-s1
12h-s2
12h-s3
2h-w1
12h-w1
24h-w1
2h-w2
0h-1
0h-2
12h-w2
12h-w3
2h-s1
2h-s2
2h-s3
24h-s1
24h-s2
24h-s3

Fig. S1.

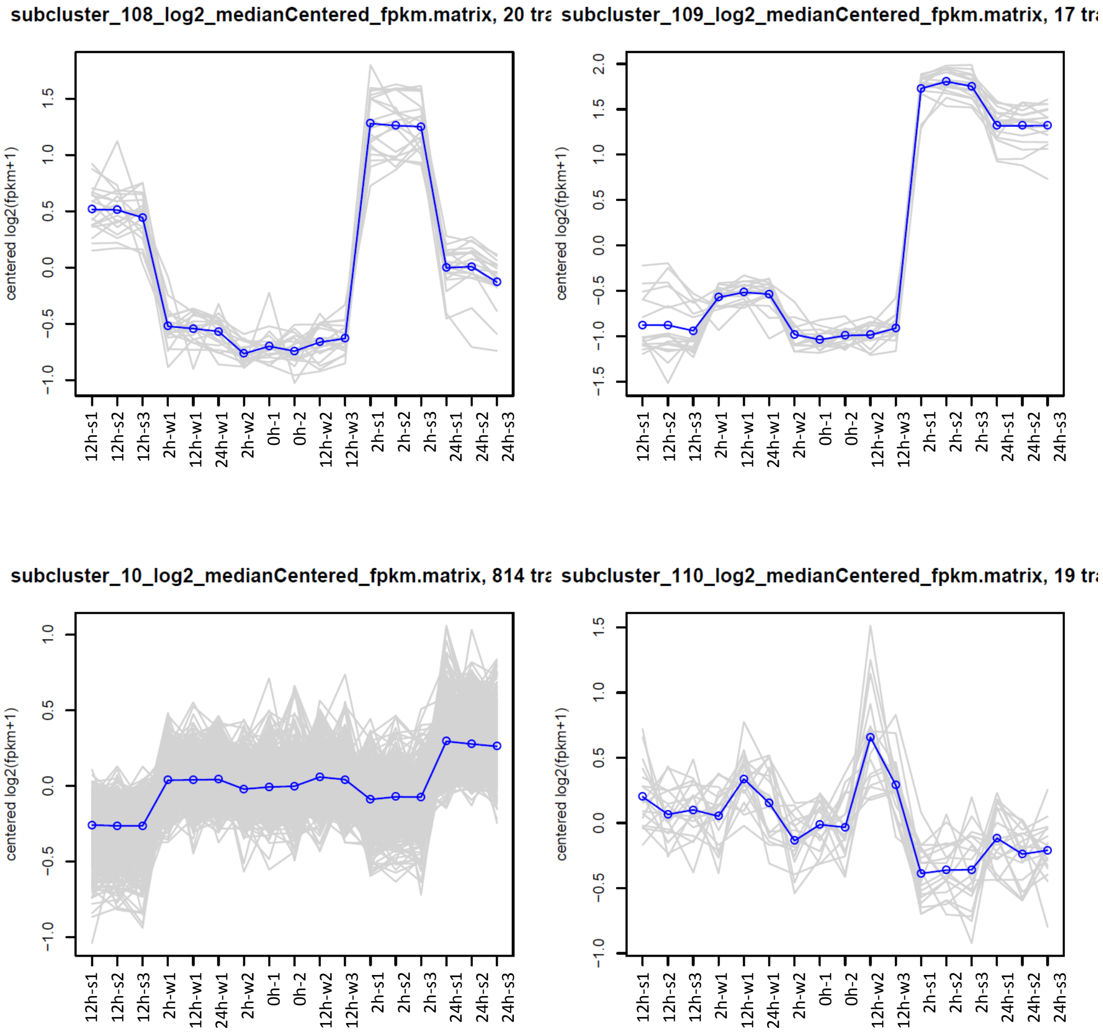
subcluster_108_log2_medianCentered_fpkm.matrix, 20 tra
subcluster_109_log2_medianCentered_fpkm.matrix, 17 tra
subcluster_10_log2_medianCentered_fpkm.matrix, 814 tra
subcluster_110_log2_medianCentered_fpkm.matrix, 19 tra
centered log2(fpkm+1)
12h-s1
12h-s2
12h-s3
2h-w1
12h-w1
24h-w1
2h-w2
0h-1
0h-2
12h-w2
12h-w3
2h-s1
2h-s2
2h-s3
24h-s1
24h-s2
24h-s3

Fig. S1.

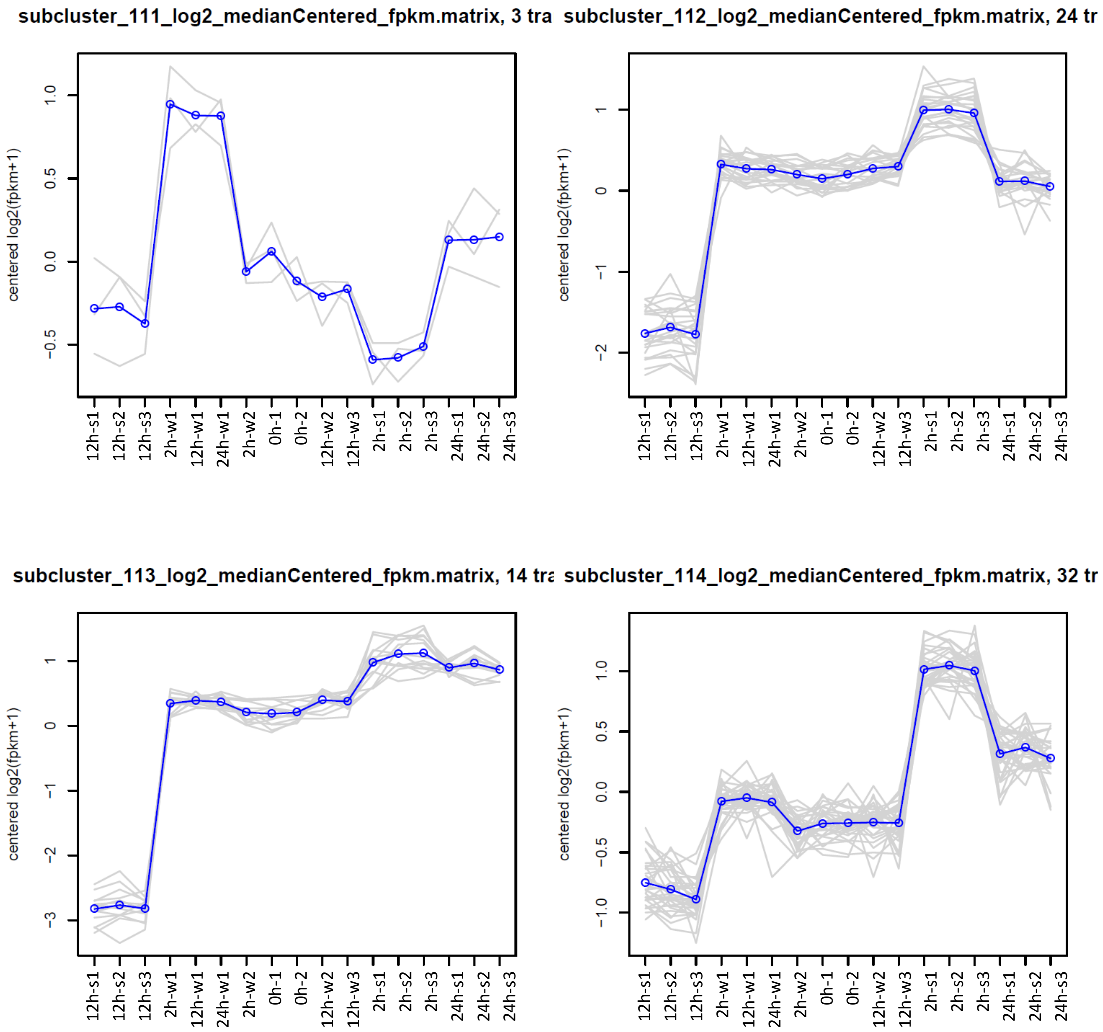

subcluster_111_log2_medianCentered_fpkm.matrix, 3 tra
subcluster_112_log2_medianCentered_fpkm.matrix, 24 tr
subcluster_113_log2_medianCentered_fpkm.matrix, 14 tra
subcluster_114_log2_medianCentered_fpkm.matrix, 32 tr
centered log2(fpkm+1)
12h-s1
12h-s2
12h-s3
2h-w1
12h-w1
24h-w1
2h-w2
0h-1
0h-2
12h-w2
12h-w3
2h-s1
2h-s2
2h-s3
24h-s1
24h-s2
24h-s3

Fig. S1.

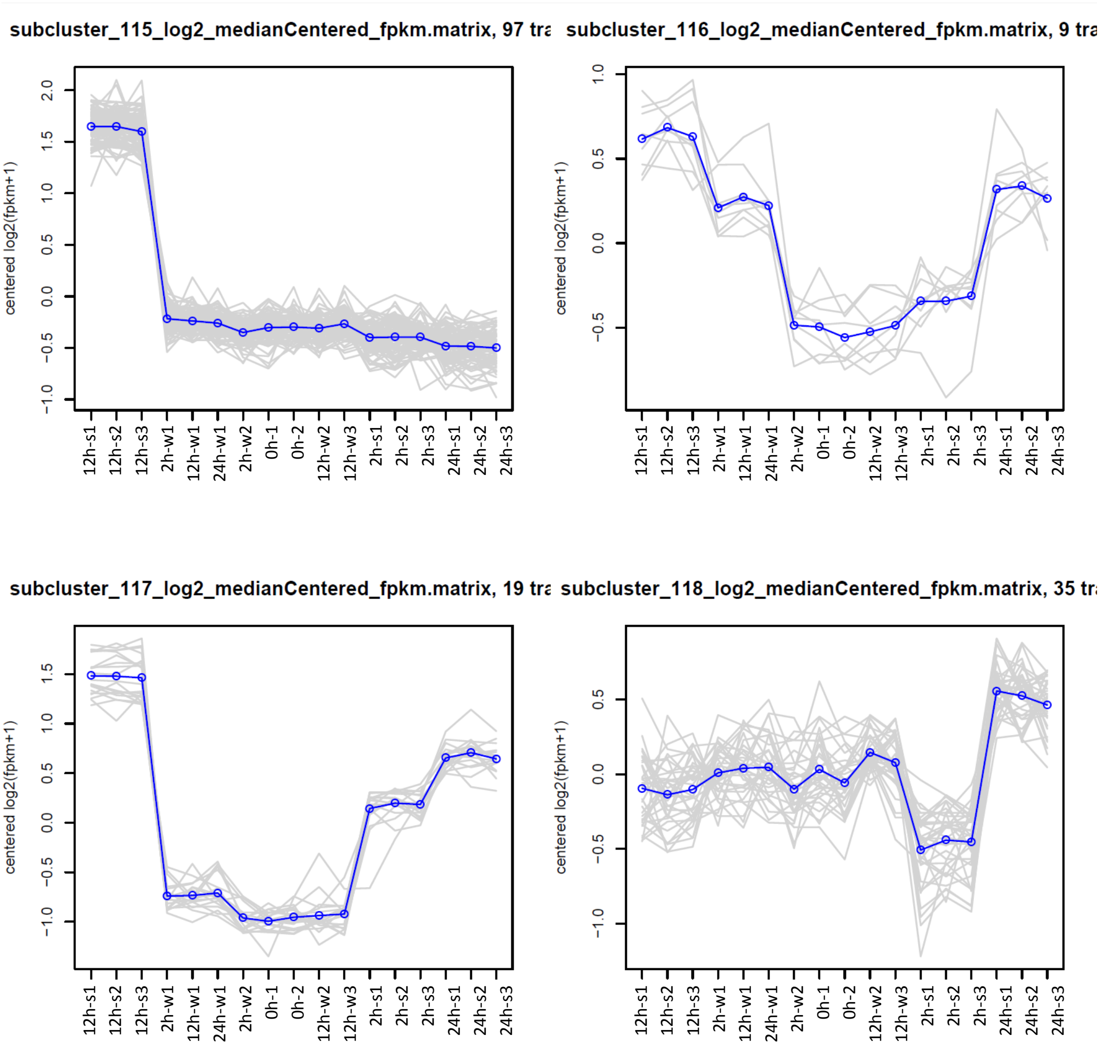
subcluster_115_log2_medianCentered_fpkm.matrix, 97 tra
subcluster_116_log2_medianCentered_fpkm.matrix, 9 tra
subcluster_117_log2_medianCentered_fpkm.matrix, 19 tra
subcluster_118_log2_medianCentered_fpkm.matrix, 35 tra
centered log2(fpkm+1)
12h-s1
12h-s2
12h-s3
2h-w1
12h-w1
24h-w1
2h-w2
0h-1
0h-2
12h-w2
12h-w3
2h-s1
2h-s2
2h-s3
24h-s1
24h-s2
24h-s3

Fig. S1.

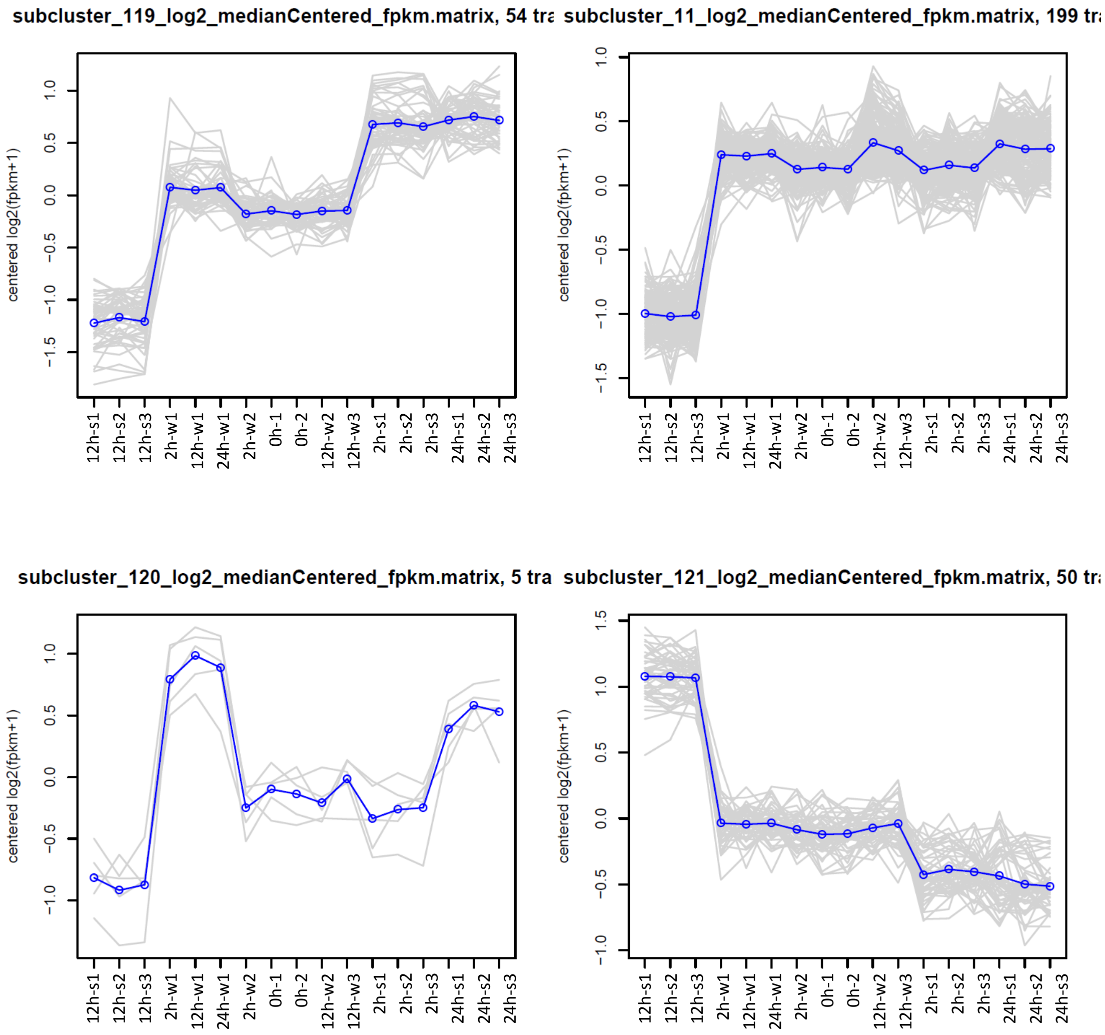

subcluster_119_log2_medianCentered_fpkm.matrix, 54 tra
subcluster_11_log2_medianCentered_fpkm.matrix, 199 tr
subcluster_120_log2_medianCentered_fpkm.matrix, 5 tra
subcluster_121_log2_medianCentered_fpkm.matrix, 50 tr
centered log2(fpkm+1)
−1.5
−1.0
−0.5
0.0
0.5
1.0
1.5
12h-s1
12h-s2
12h-s3
2h-w1
12h-w1
24h-w1
2h-w2
0h-1
0h-2
12h-w2
12h-w3
2h-s1
2h-s2
2h-s3
24h-s1
24h-s2
24h-s3

Fig. S1.

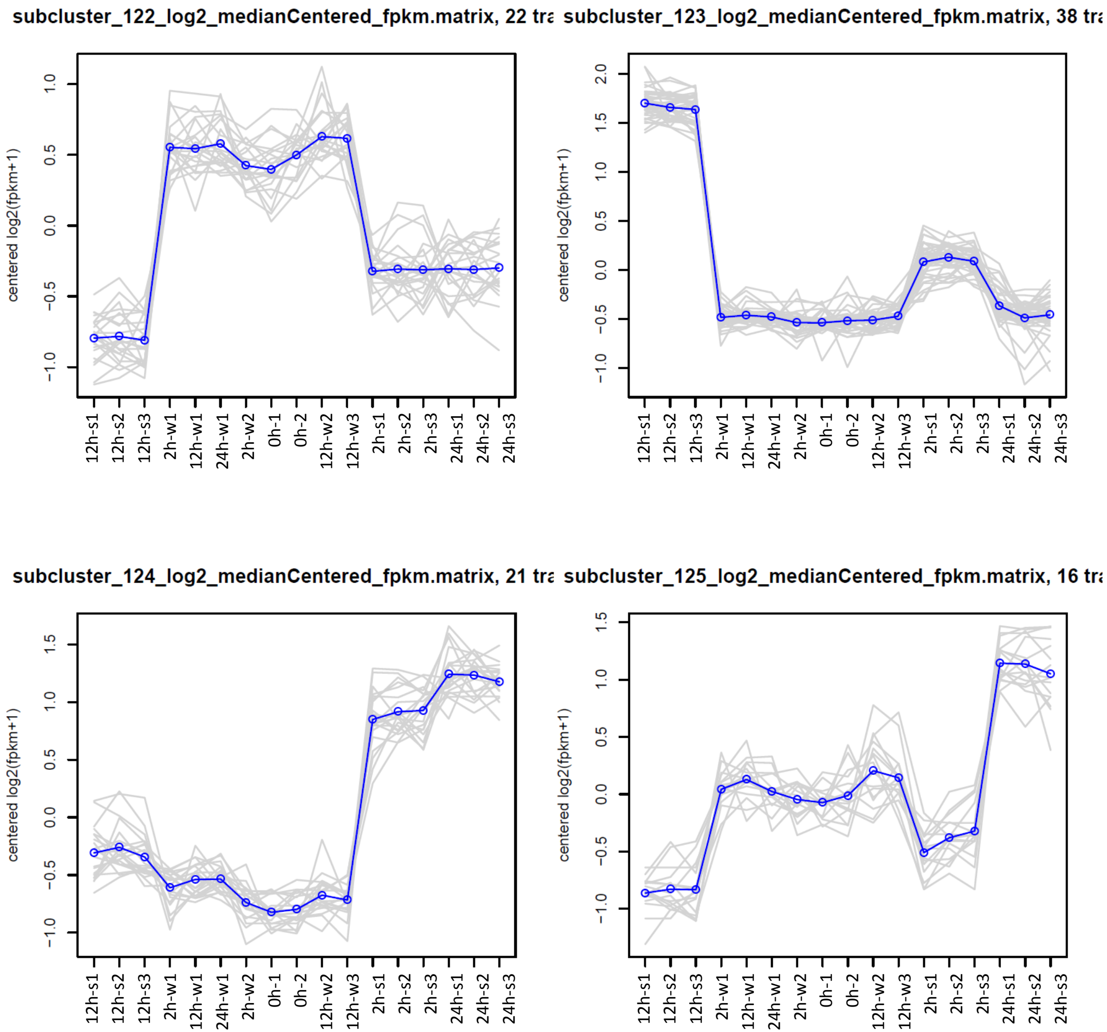
subcluster_122_log2_medianCentered_fpkm.matrix, 22 tra
subcluster_123_log2_medianCentered_fpkm.matrix, 38 tra
subcluster_124_log2_medianCentered_fpkm.matrix, 21 tra
subcluster_125_log2_medianCentered_fpkm.matrix, 16 tra
centered log2(fpkm+1)
12h-s1
12h-s2
12h-s3
2h-w1
12h-w1
24h-w1
2h-w2
0h-1
0h-2
12h-w2
12h-w3
2h-s1
2h-s2
2h-s3
24h-s1
24h-s2
24h-s3

Fig. S1.

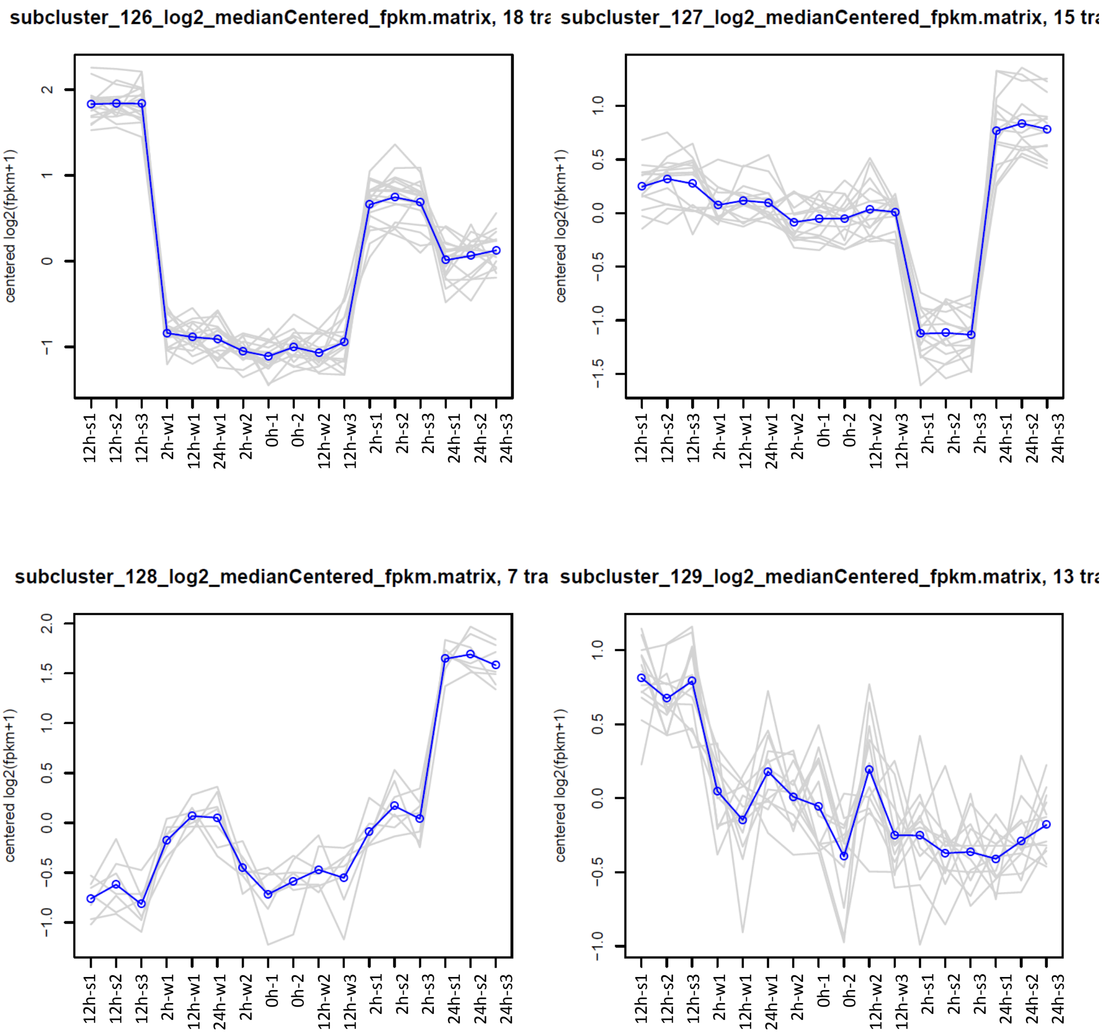
subcluster_126_log2_medianCentered_fpkm.matrix, 18 tra
subcluster_127_log2_medianCentered_fpkm.matrix, 15 tra
subcluster_128_log2_medianCentered_fpkm.matrix, 7 tra
subcluster_129_log2_medianCentered_fpkm.matrix, 13 tra
centered log2(fpkm+1)
12h-s1
12h-s2
12h-s3
2h-w1
12h-w1
24h-w1
2h-w2
0h-1
0h-2
12h-w2
12h-w3
2h-s1
2h-s2
2h-s3
24h-s1
24h-s2
24h-s3

Fig. S1.

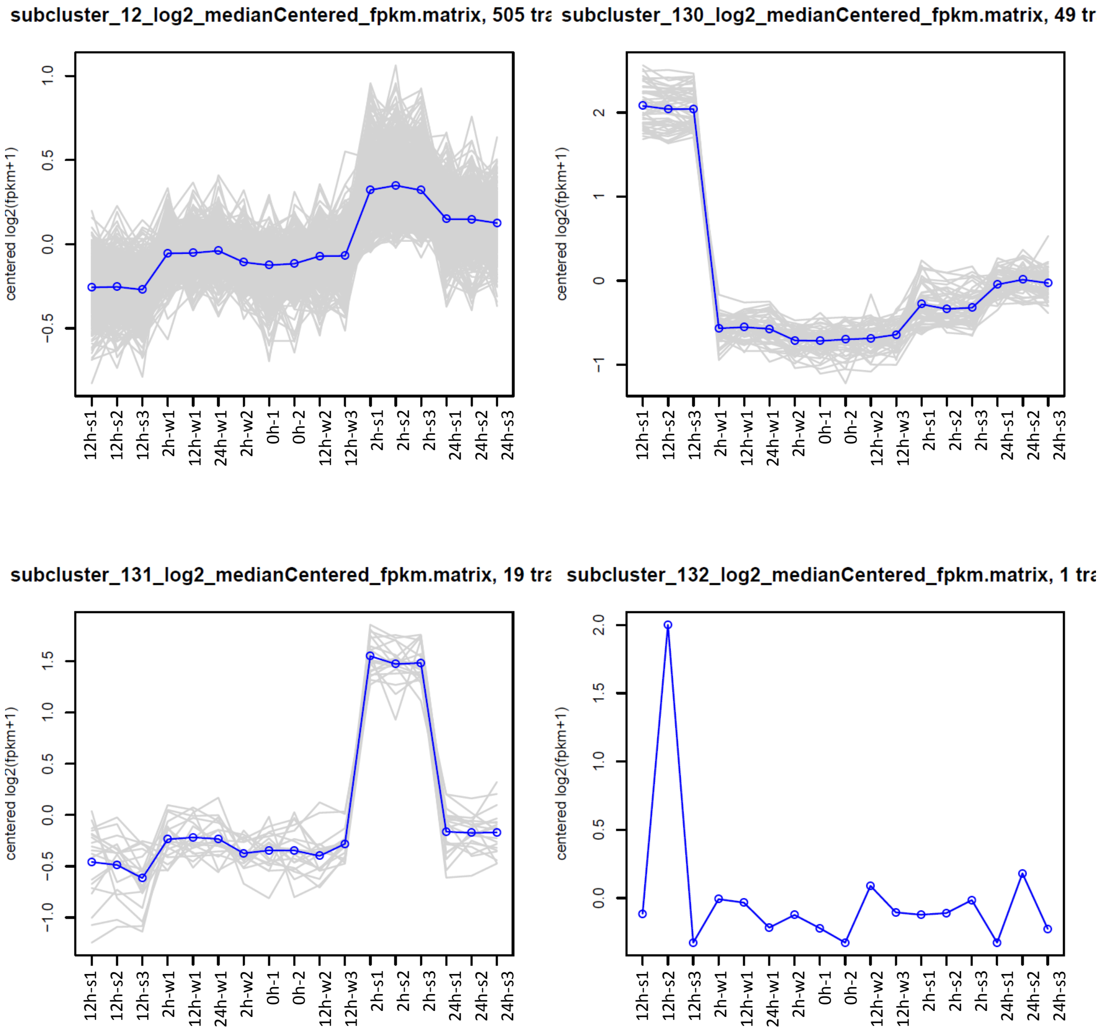
subcluster_12_log2_medianCentered_fpkm.matrix, 505 tra
subcluster_130_log2_medianCentered_fpkm.matrix, 49 tr
subcluster_131_log2_medianCentered_fpkm.matrix, 19 tra
subcluster_132_log2_medianCentered_fpkm.matrix, 1 tra
centered log2(fpkm+1)
12h-s1
12h-s2
12h-s3
2h-w1
12h-w1
24h-w1
2h-w2
0h-1
0h-2
12h-w2
12h-w3
2h-s1
2h-s2
2h-s3
24h-s1
24h-s2
24h-s3

Fig. S1.

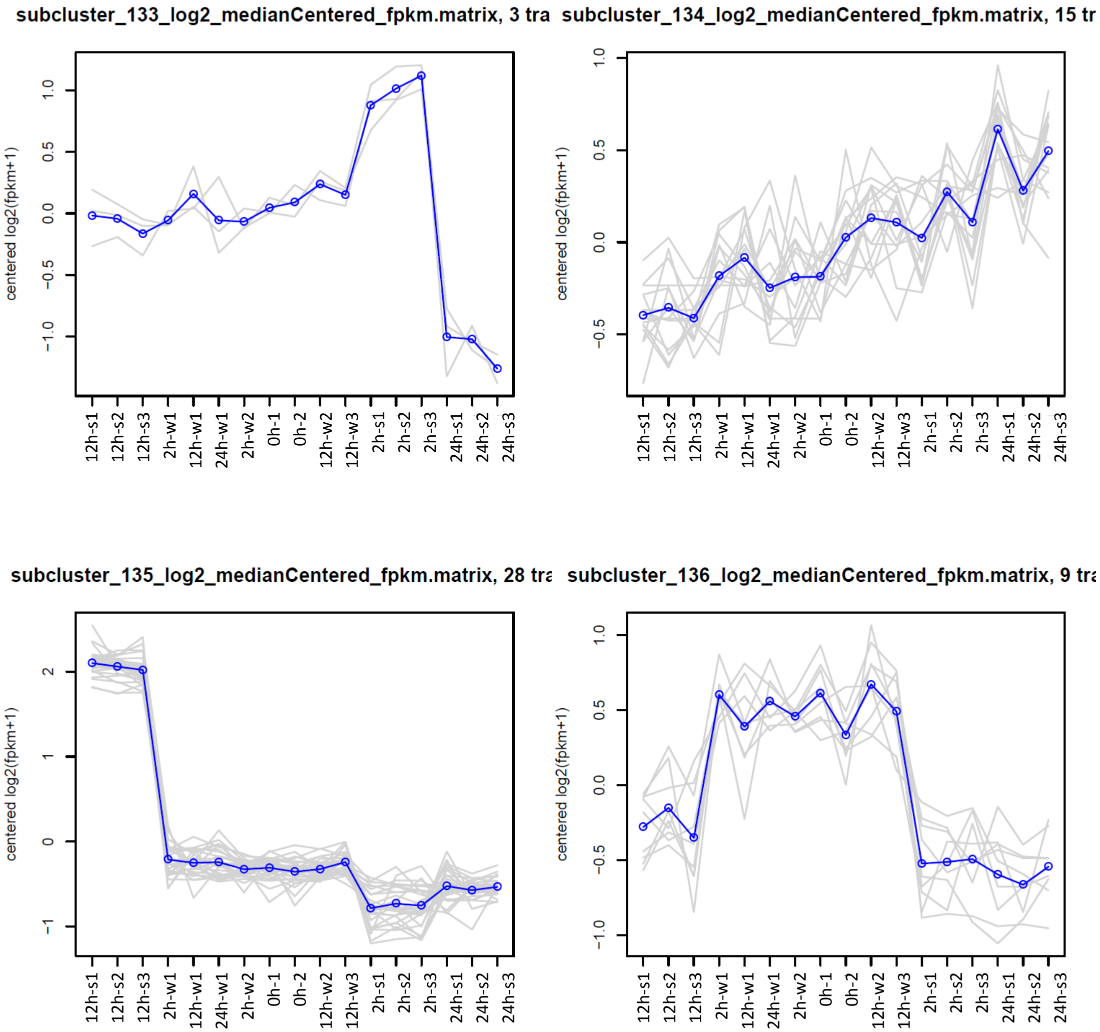
subcluster_133_log2_medianCentered_fpkm.matrix, 3 tra
subcluster_134_log2_medianCentered_fpkm.matrix, 15 tr
subcluster_135_log2_medianCentered_fpkm.matrix, 28 tra
subcluster_136_log2_medianCentered_fpkm.matrix, 9 tra
centered log2(fpkm+1)
12h-s1 12h-s2 12h-s3 2h-w1 12h-w1 24h-w1 2h-w2 0h-1 0h-2 12h-w2 12h-w3 2h-s1 2h-s2 2h-s3 24h-s1 24h-s2 24h-s3

Fig. S1.

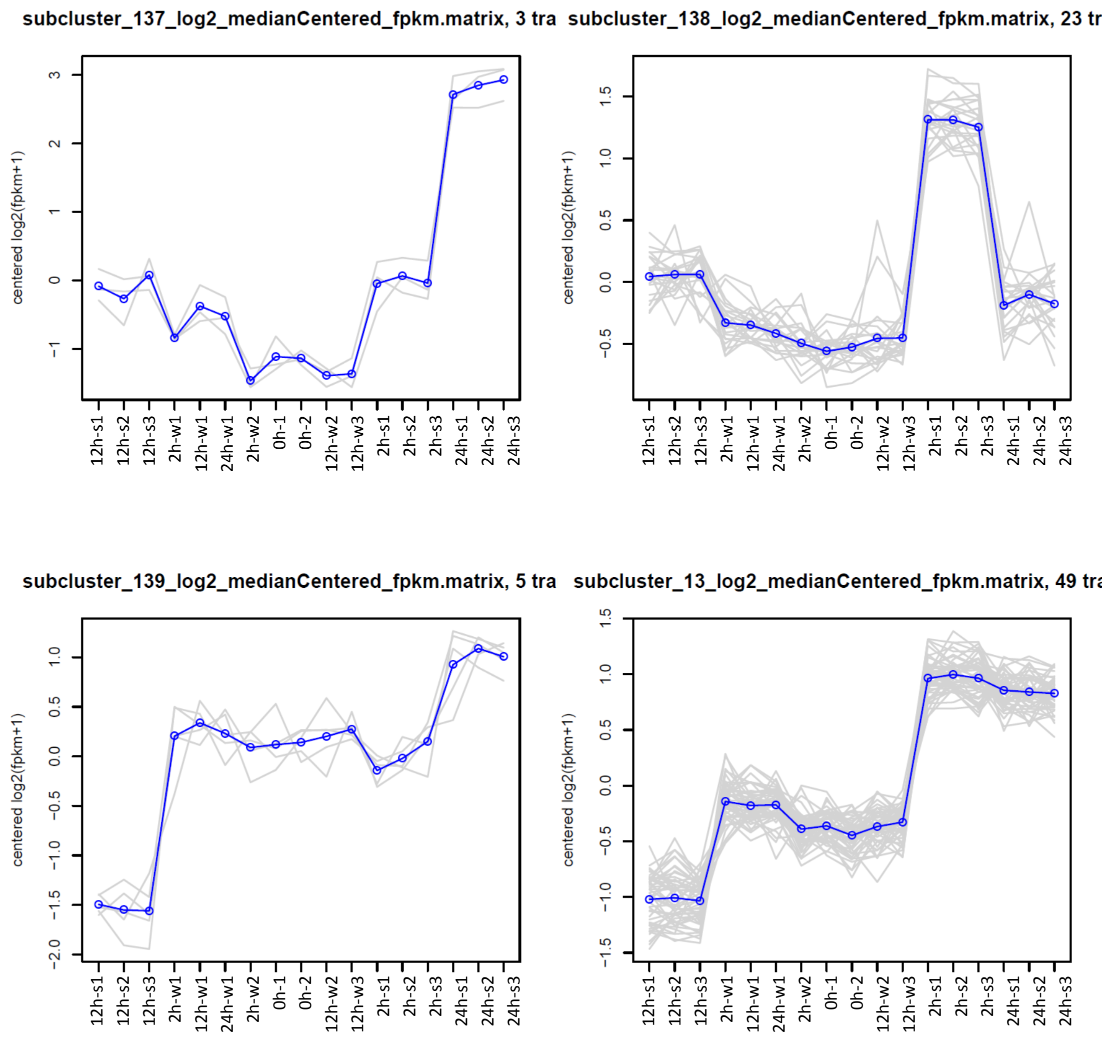
subcluster_137_log2_medianCentered_fpkm.matrix, 3 tra
subcluster_138_log2_medianCentered_fpkm.matrix, 23 tr
subcluster_139_log2_medianCentered_fpkm.matrix, 5 tra
subcluster_13_log2_medianCentered_fpkm.matrix, 49 tra
centered log2(fpkm+1)
12h-s1
12h-s2
12h-s3
2h-w1
12h-w1
24h-w1
2h-w2
0h-1
0h-2
12h-w2
12h-w3
2h-s1
2h-s2
2h-s3
24h-s1
24h-s2
24h-s3

Fig. S1.

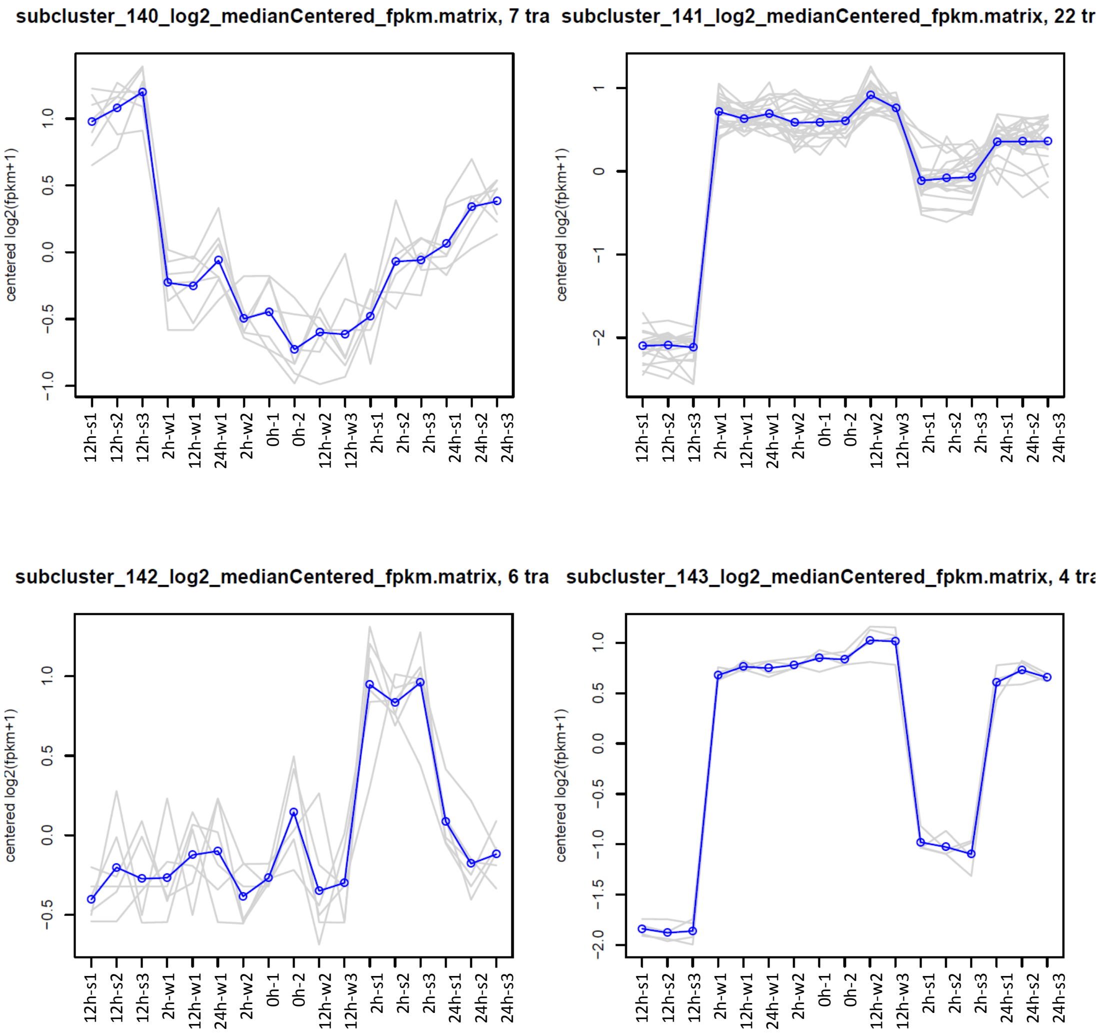
subcluster_140_log2_medianCentered_fpkm.matrix, 7 tra
subcluster_141_log2_medianCentered_fpkm.matrix, 22 tr
subcluster_142_log2_medianCentered_fpkm.matrix, 6 tra
subcluster_143_log2_medianCentered_fpkm.matrix, 4 tra
centered log2(fpkm+1)
12h-s1
12h-s2
12h-s3
2h-w1
12h-w1
24h-w1
2h-w2
0h-1
0h-2
12h-w2
12h-w3
2h-s1
2h-s2
2h-s3
24h-s1
24h-s2
24h-s3

Fig. S1.

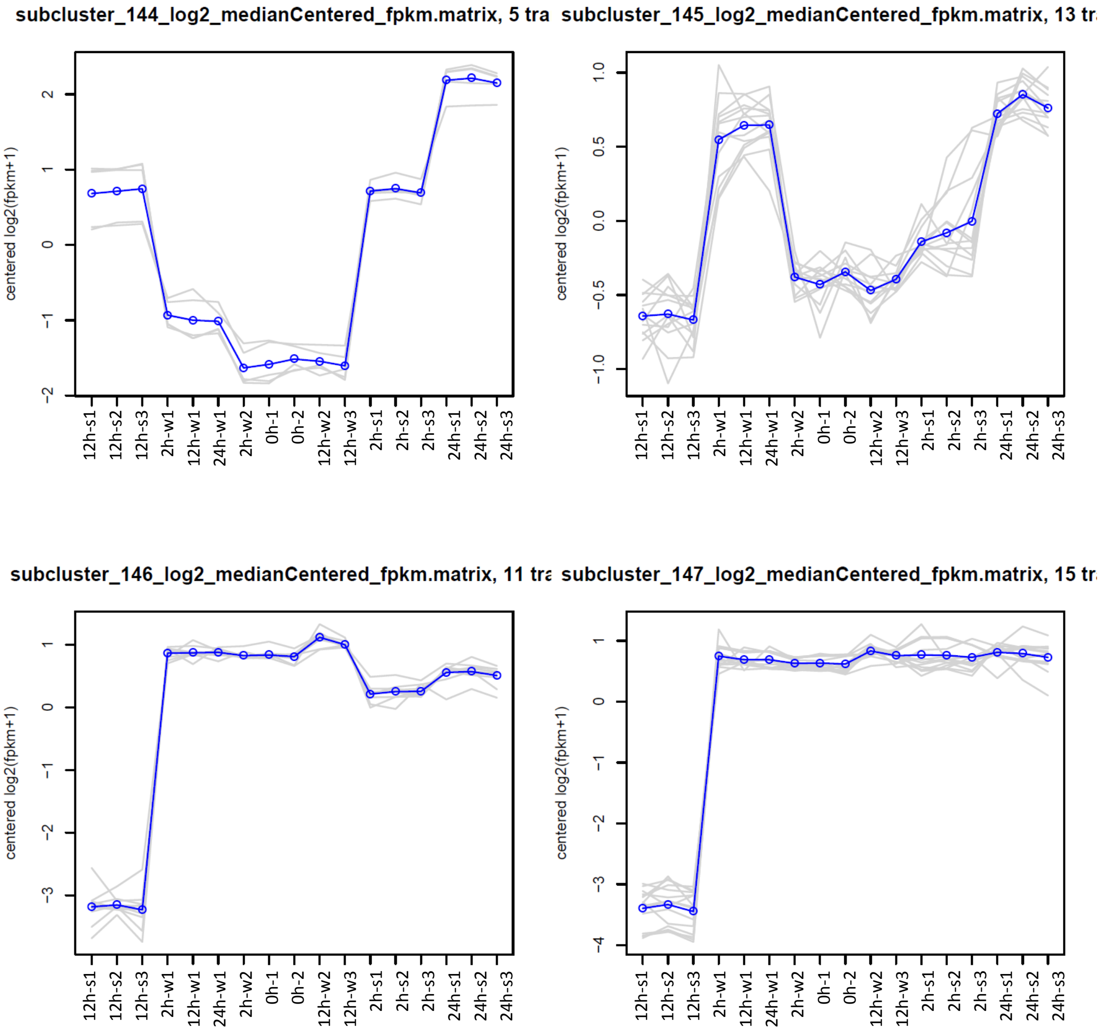

subcluster_144_log2_medianCentered_fpkm.matrix, 5 tra
subcluster_145_log2_medianCentered_fpkm.matrix, 13 tr
subcluster_146_log2_medianCentered_fpkm.matrix, 11 tra
subcluster_147_log2_medianCentered_fpkm.matrix, 15 tr
centered log2(fpkm+1)
12h-s1
12h-s2
12h-s3
2h-w1
12h-w1
24h-w1
2h-w2
0h-1
0h-2
12h-w2
12h-w3
2h-s1
2h-s2
2h-s3
24h-s1
24h-s2
24h-s3

Fig. S1.

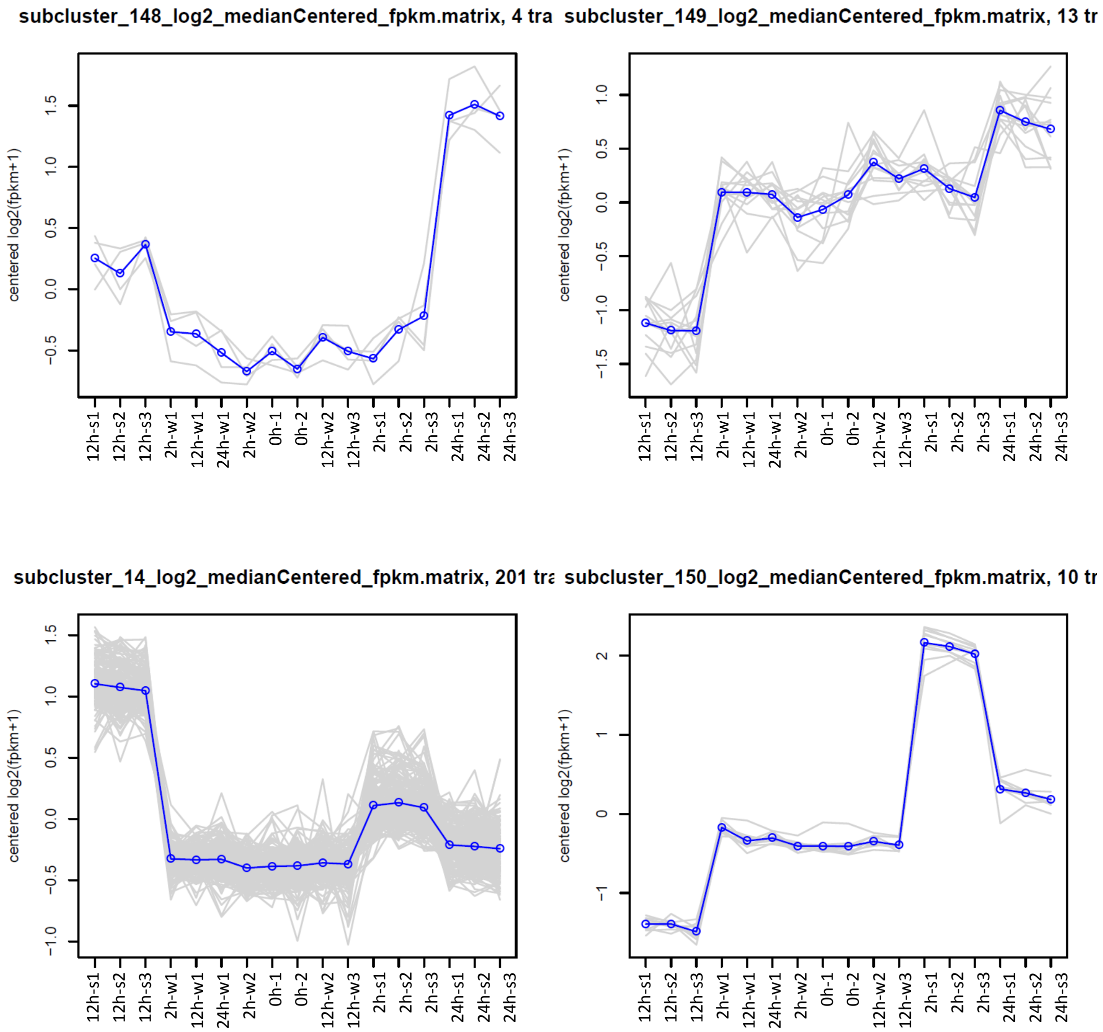
subcluster_148_log2_medianCentered_fpkm.matrix, 4 tra
subcluster_149_log2_medianCentered_fpkm.matrix, 13 tr
subcluster_14_log2_medianCentered_fpkm.matrix, 201 tra
subcluster_150_log2_medianCentered_fpkm.matrix, 10 tr
centered log2(fpkm+1)
12h-s1
12h-s2
12h-s3
2h-w1
12h-w1
24h-w1
2h-w2
0h-1
0h-2
12h-w2
12h-w3
2h-s1
2h-s2
2h-s3
24h-s1
24h-s2
24h-s3

Fig. S1.

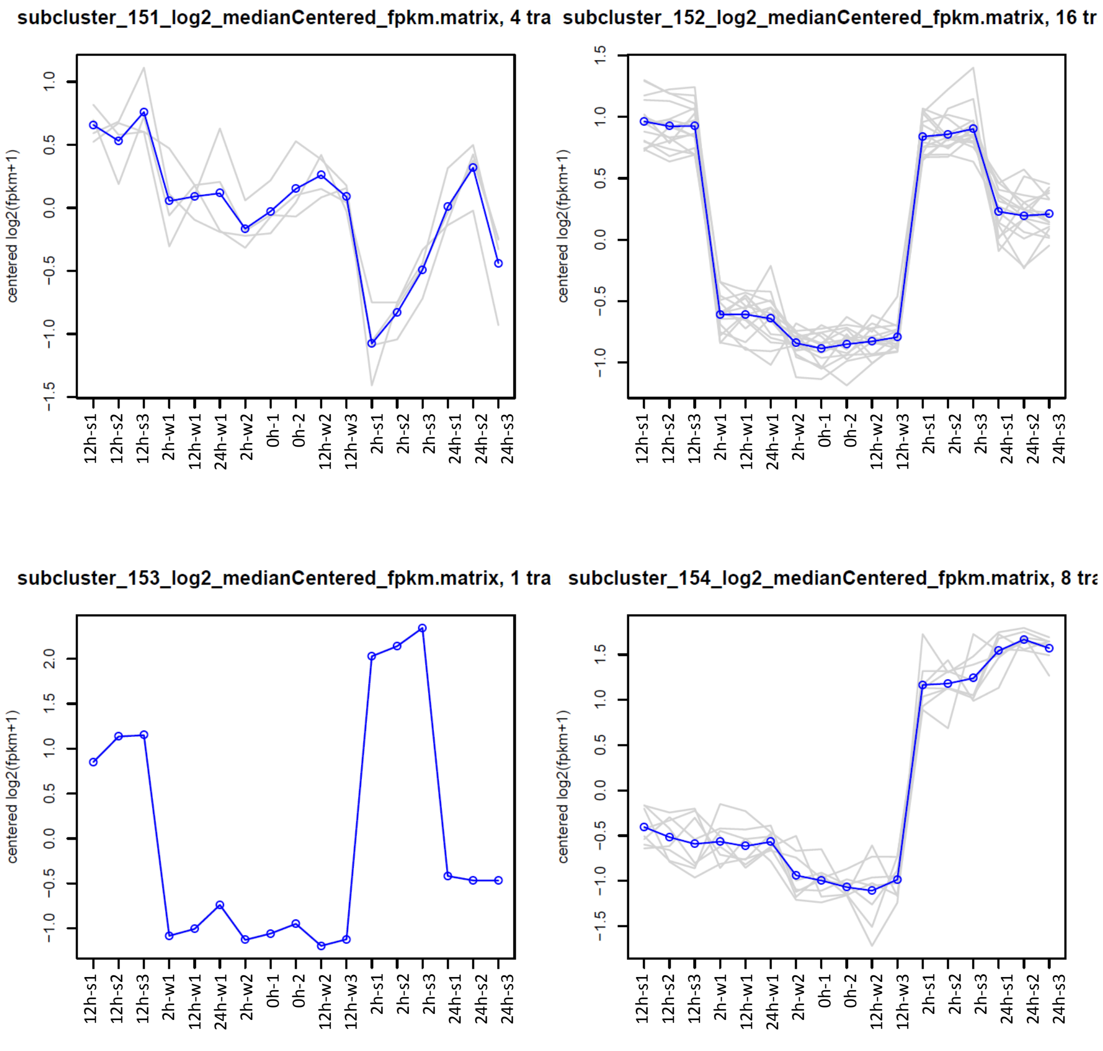
subcluster_151_log2_medianCentered_fpkm.matrix, 4 tra
subcluster_152_log2_medianCentered_fpkm.matrix, 16 tr
subcluster_153_log2_medianCentered_fpkm.matrix, 1 tra
subcluster_154_log2_medianCentered_fpkm.matrix, 8 tra
centered log2(fpkm+1)
12h-s1
12h-s2
12h-s3
2h-w1
12h-w1
24h-w1
2h-w2
0h-1
0h-2
12h-w2
12h-w3
2h-s1
2h-s2
2h-s3
24h-s1
24h-s2
24h-s3

Fig. S1.

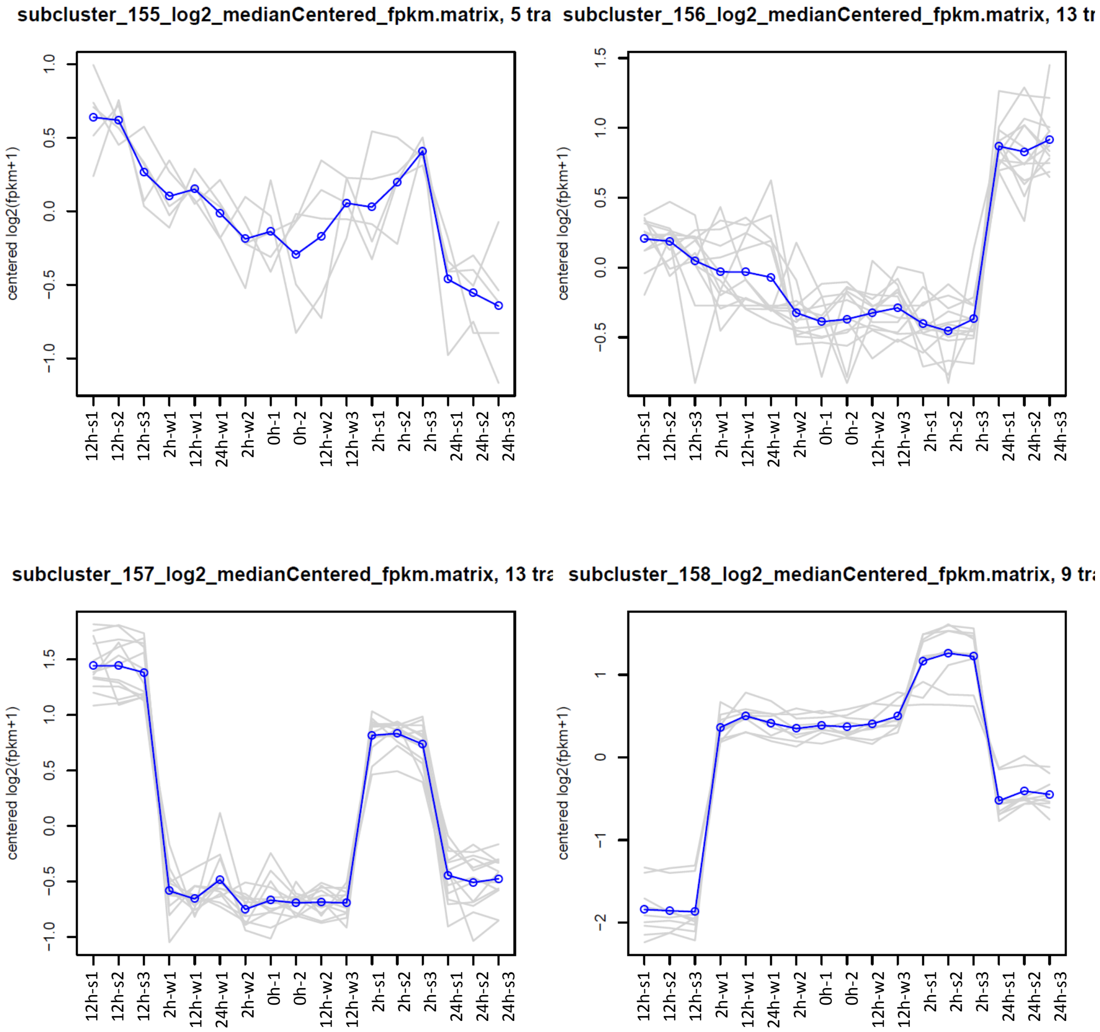

subcluster_155_log2_medianCentered_fpkm.matrix, 5 tra
subcluster_156_log2_medianCentered_fpkm.matrix, 13 tr
subcluster_157_log2_medianCentered_fpkm.matrix, 13 tra
subcluster_158_log2_medianCentered_fpkm.matrix, 9 tra
centered log2(fpkm+1)
12h-s1
12h-s2
12h-s3
2h-w1
12h-w1
24h-w1
2h-w2
0h-1
0h-2
12h-w2
12h-w3
2h-s1
2h-s2
2h-s3
24h-s1
24h-s2
24h-s3

Fig. S1.

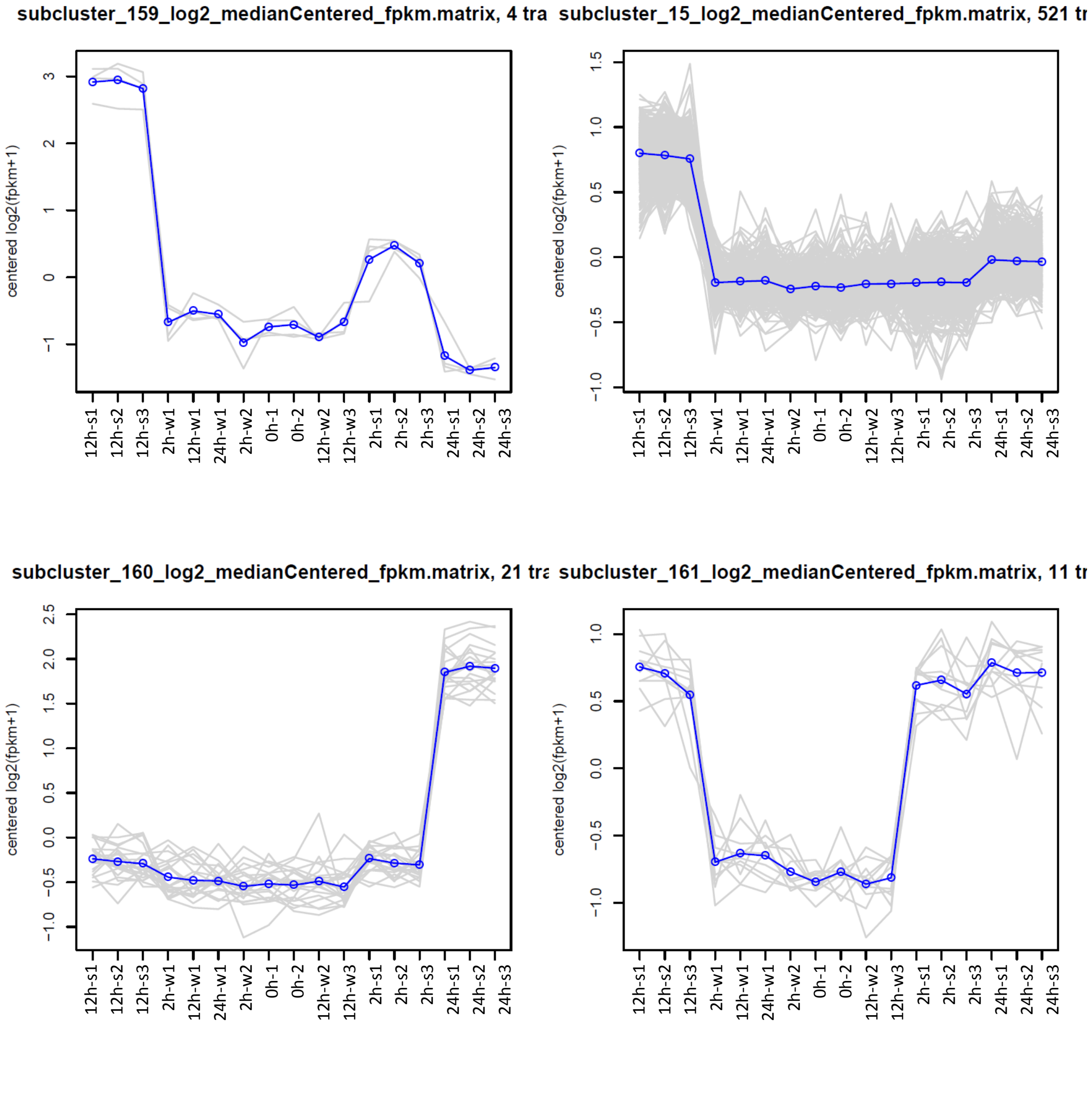

subcluster_159_log2_medianCentered_fpkm.matrix, 4 tra
subcluster_15_log2_medianCentered_fpkm.matrix, 521 tr
subcluster_160_log2_medianCentered_fpkm.matrix, 21 tra
subcluster_161_log2_medianCentered_fpkm.matrix, 11 tr
centered log2(fpkm+1)
12h-s1
12h-s2
12h-s3
2h-w1
12h-w1
24h-w1
2h-w2
0h-1
0h-2
12h-w2
12h-w3
2h-s1
2h-s2
2h-s3
24h-s1
24h-s2
24h-s3

Fig. S1.

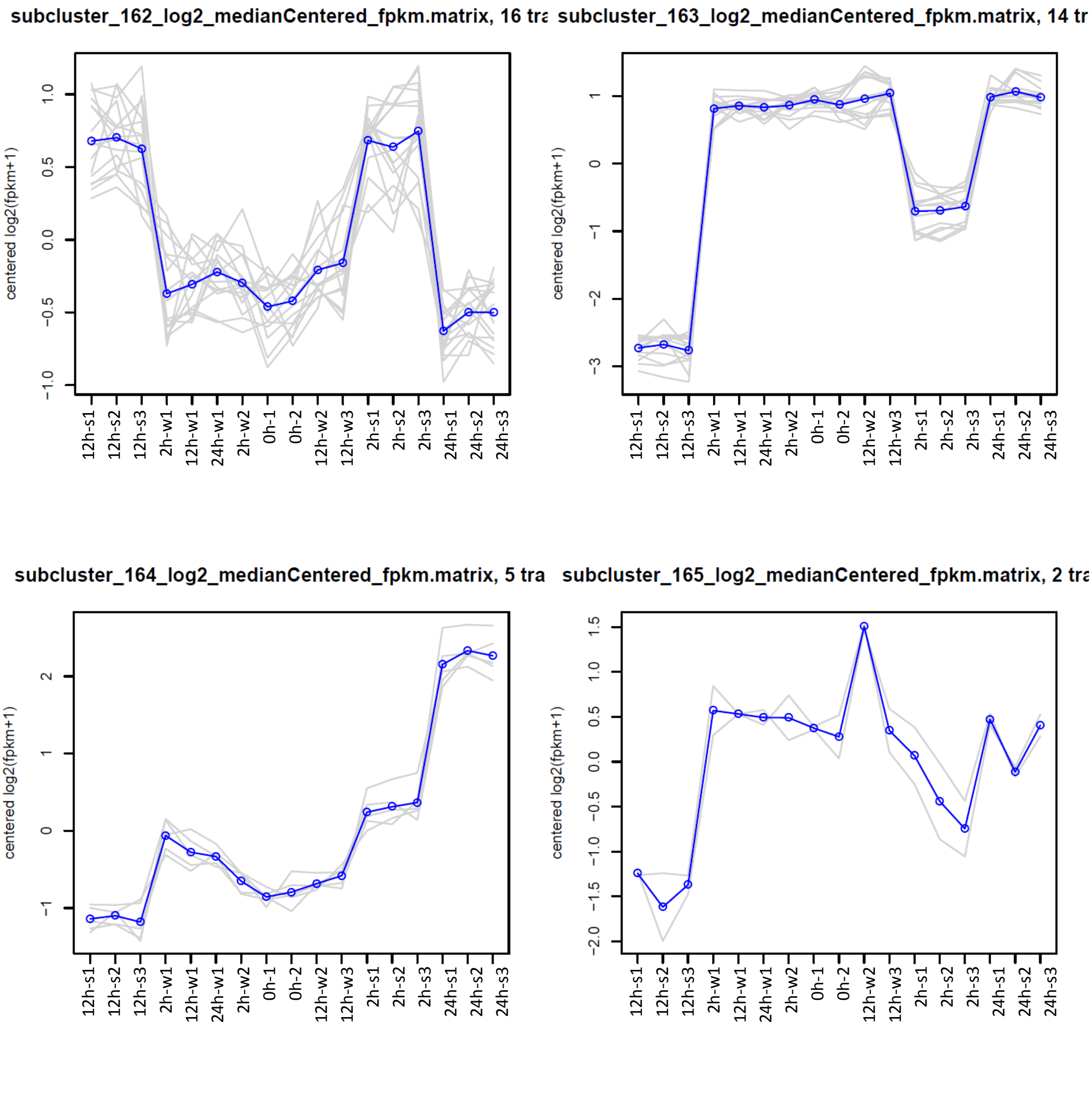

subcluster_162_log2_medianCentered_fpkm.matrix, 16 tra
subcluster_163_log2_medianCentered_fpkm.matrix, 14 tr
subcluster_164_log2_medianCentered_fpkm.matrix, 5 tra
subcluster_165_log2_medianCentered_fpkm.matrix, 2 tra
centered log2(fpkm+1)
12h-s1
12h-s2
12h-s3
2h-w1
12h-w1
24h-w1
2h-w2
0h-1
0h-2
12h-w2
12h-w3
2h-s1
2h-s2
2h-s3
24h-s1
24h-s2
24h-s3

Fig. S1.

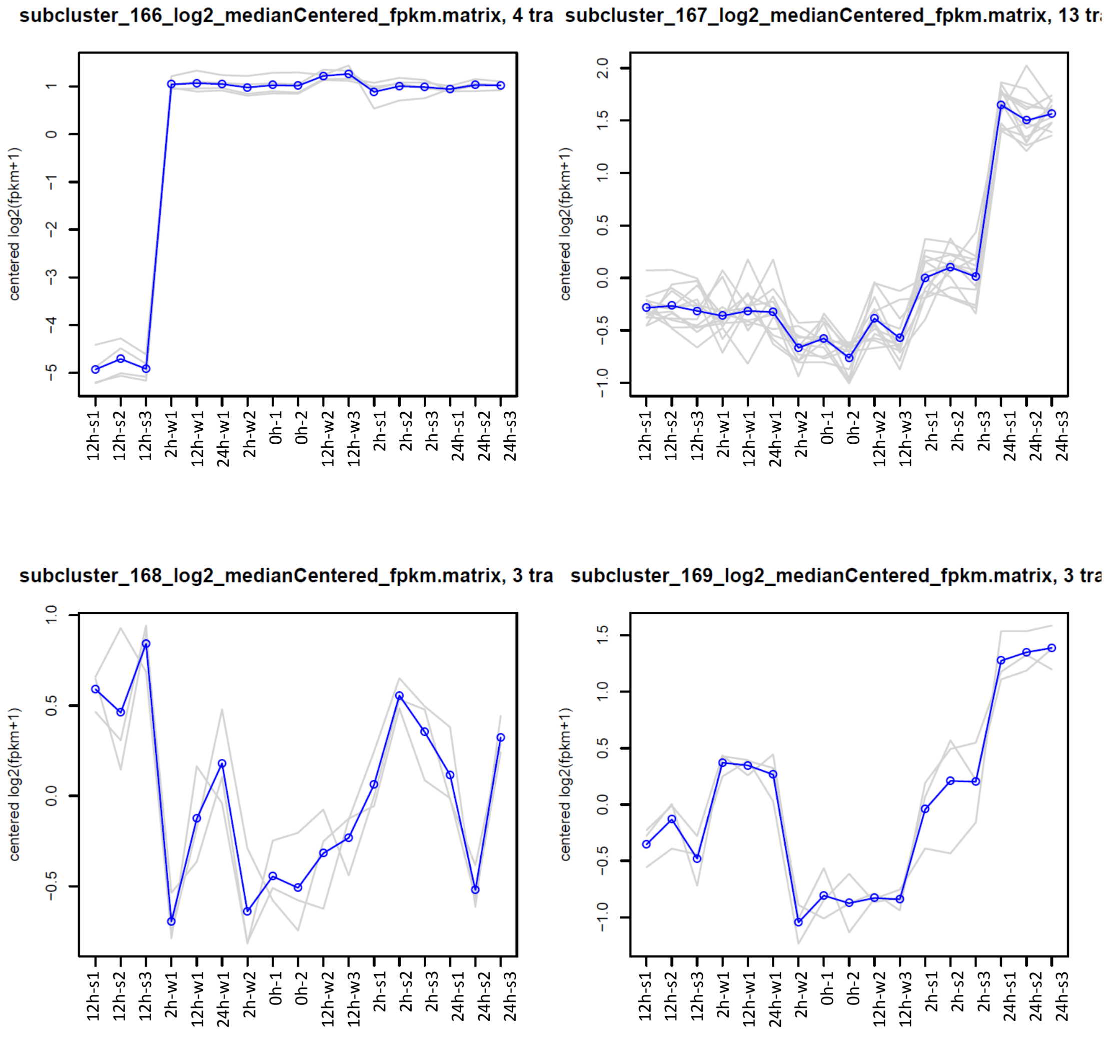
subcluster_166_log2_medianCentered_fpkm.matrix, 4 tra
subcluster_167_log2_medianCentered_fpkm.matrix, 13 tr
subcluster_168_log2_medianCentered_fpkm.matrix, 3 tra
subcluster_169_log2_medianCentered_fpkm.matrix, 3 tra
centered log2(fpkm+1)
12h-s1
12h-s2
12h-s3
2h-w1
12h-w1
24h-w1
2h-w2
0h-1
0h-2
12h-w2
12h-w3
2h-s1
2h-s2
2h-s3
24h-s1
24h-s2
24h-s3

Fig. S1.

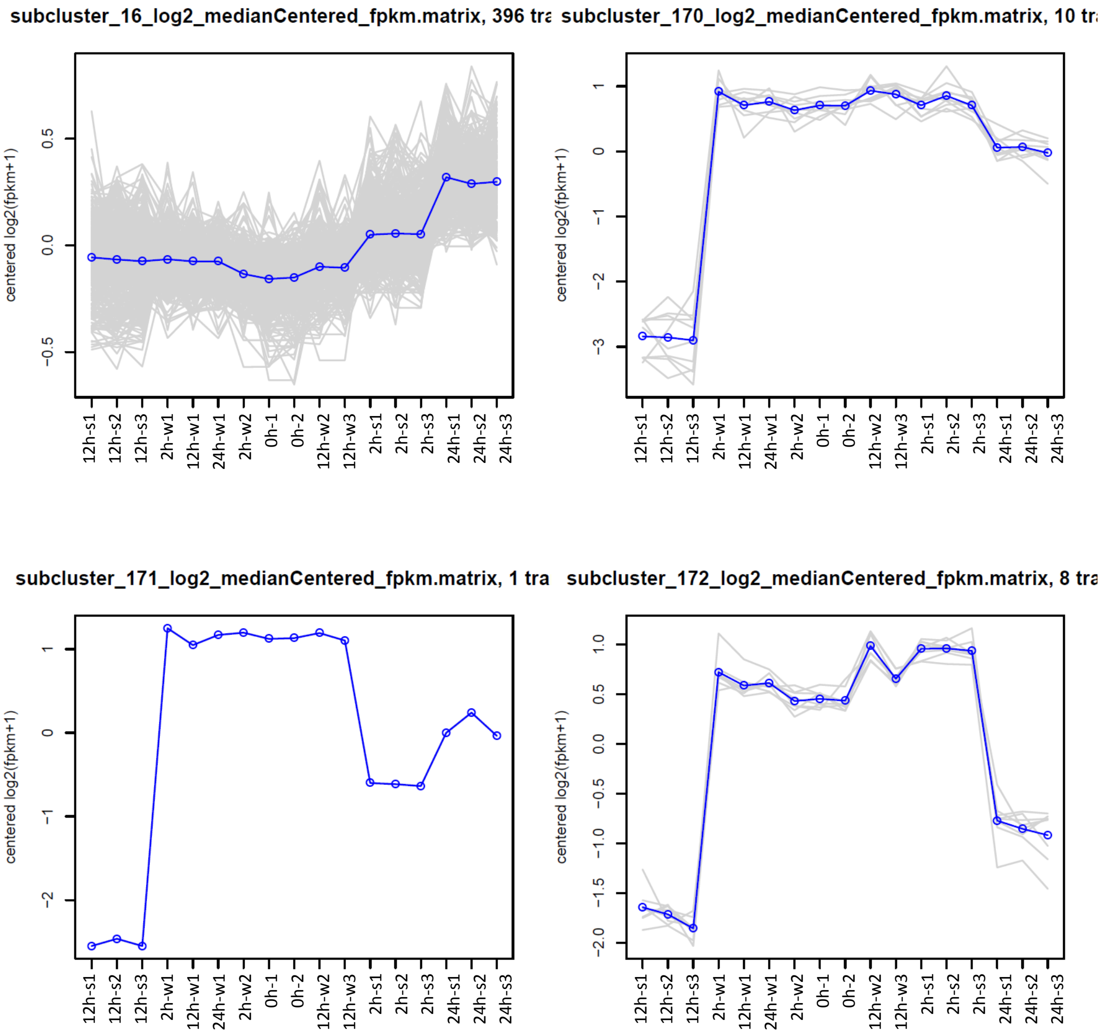

subcluster_16_log2_medianCentered_fpkm.matrix, 396 tra
subcluster_170_log2_medianCentered_fpkm.matrix, 10 tra
subcluster_171_log2_medianCentered_fpkm.matrix, 1 tra
subcluster_172_log2_medianCentered_fpkm.matrix, 8 tra
centered log2(fpkm+1)
12h-s1
12h-s2
12h-s3
2h-w1
12h-w1
24h-w1
2h-w2
0h-1
0h-2
12h-w2
12h-w3
2h-s1
2h-s2
2h-s3
24h-s1
24h-s2
24h-s3

Fig. S1.

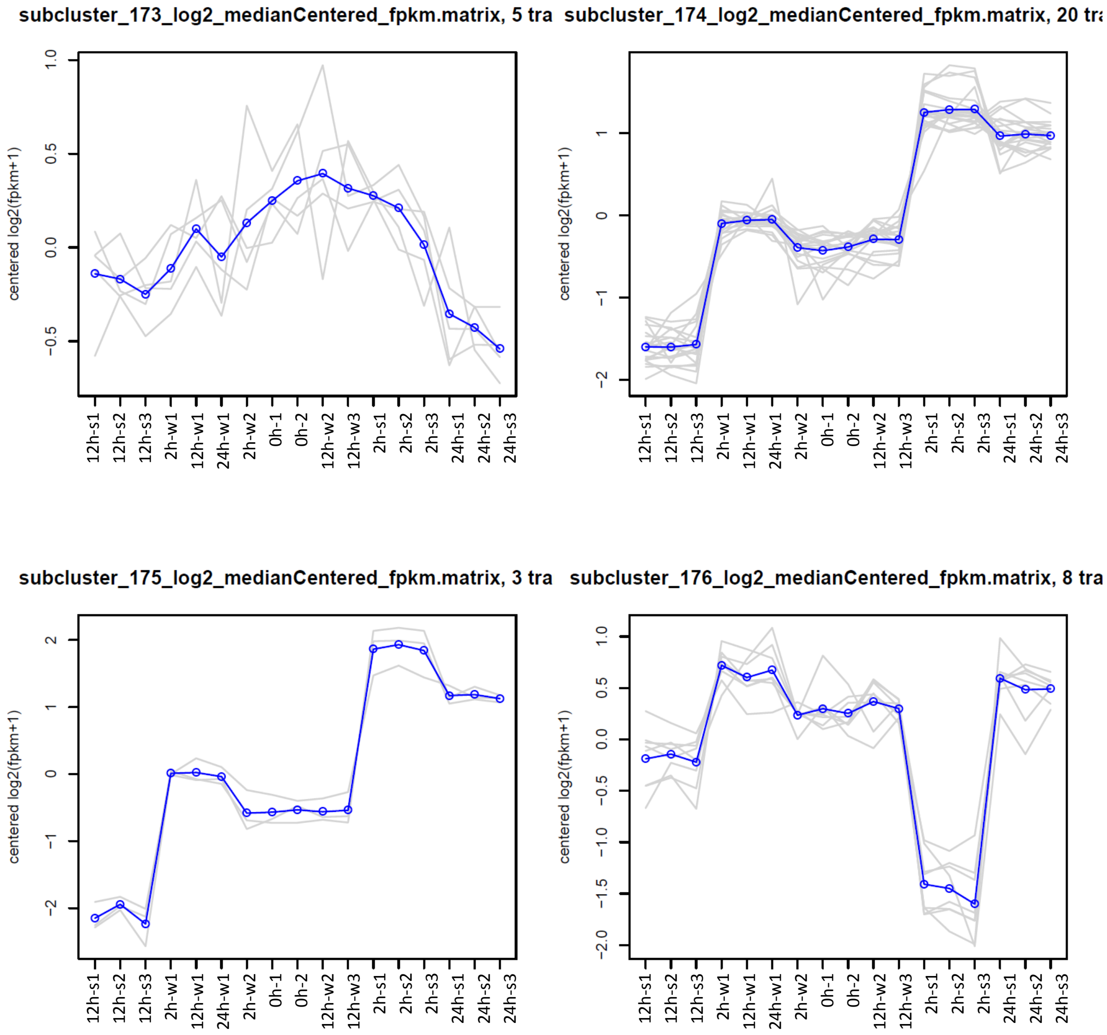
subcluster_173_log2_medianCentered_fpkm.matrix, 5 tra
subcluster_174_log2_medianCentered_fpkm.matrix, 20 tr
subcluster_175_log2_medianCentered_fpkm.matrix, 3 tra
subcluster_176_log2_medianCentered_fpkm.matrix, 8 tra
centered log2(fpkm+1)
12h-s1
12h-s2
12h-s3
2h-w1
12h-w1
24h-w1
2h-w2
0h-1
0h-2
12h-w2
12h-w3
2h-s1
2h-s2
2h-s3
24h-s1
24h-s2
24h-s3

Fig. S1.

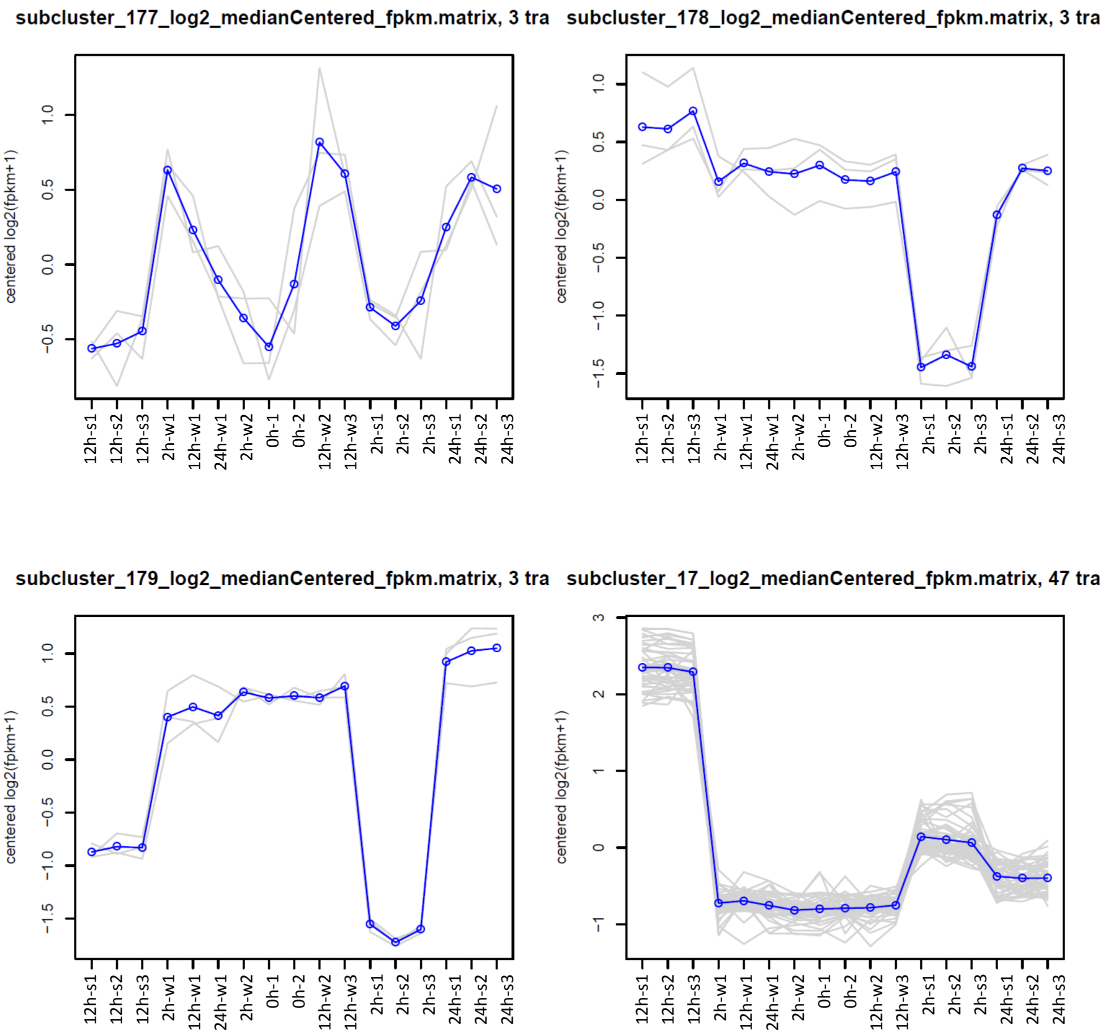
subcluster_177_log2_medianCentered_fpkm.matrix, 3 tra
subcluster_178_log2_medianCentered_fpkm.matrix, 3 tra
subcluster_179_log2_medianCentered_fpkm.matrix, 3 tra
subcluster_17_log2_medianCentered_fpkm.matrix, 47 tra
centered log2(fpkm+1)
12h-s1
12h-s2
12h-s3
2h-w1
12h-w1
24h-w1
2h-w2
0h-1
0h-2
12h-w2
12h-w3
2h-s1
2h-s2
2h-s3
24h-s1
24h-s2
24h-s3

Fig. S1.

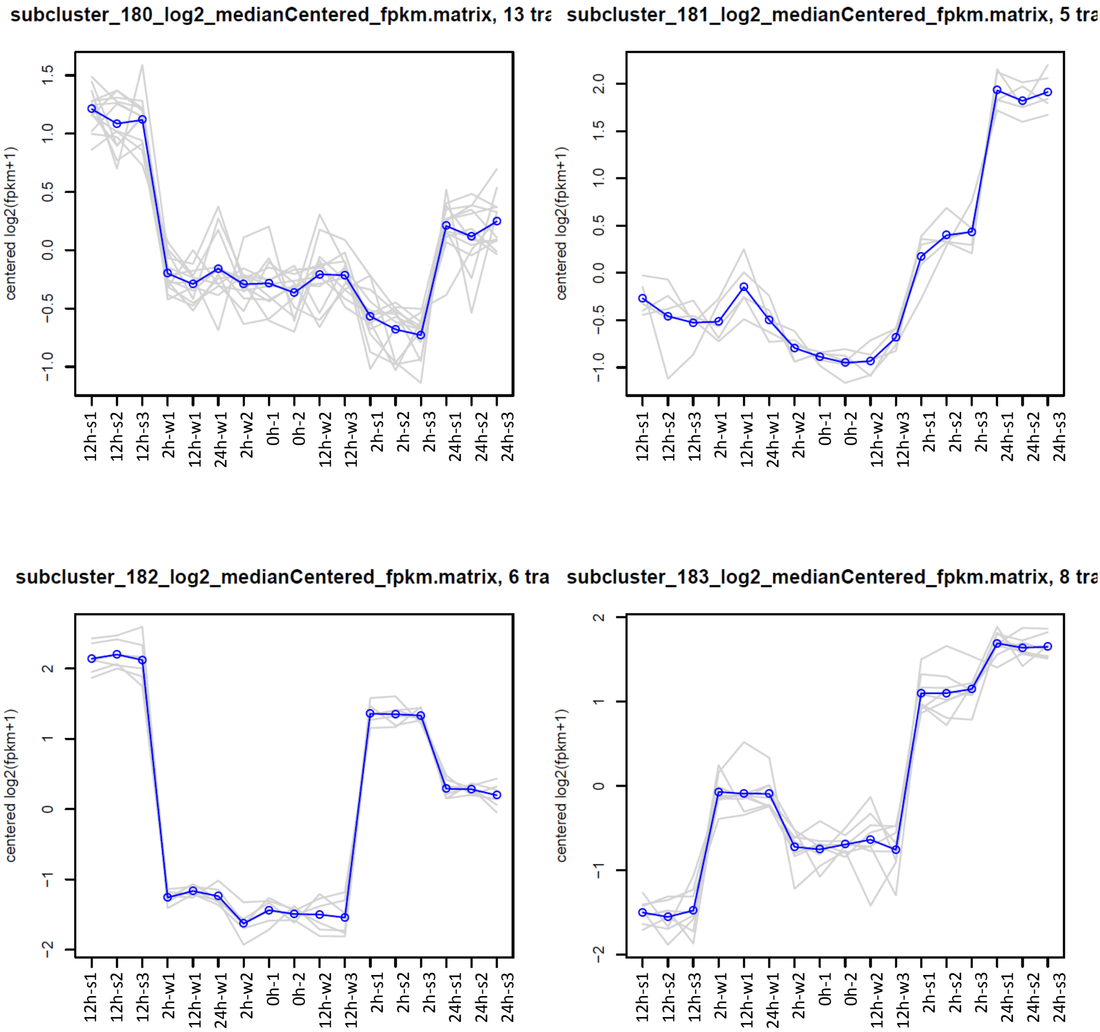
subcluster_180_log2_medianCentered_fpkm.matrix, 13 tra
subcluster_181_log2_medianCentered_fpkm.matrix, 5 tra
subcluster_182_log2_medianCentered_fpkm.matrix, 6 tra
subcluster_183_log2_medianCentered_fpkm.matrix, 8 tra
centered log2(fpkm+1)
12h-s1
12h-s2
12h-s3
2h-w1
12h-w1
24h-w1
2h-w2
0h-1
0h-2
12h-w2
12h-w3
2h-s1
2h-s2
2h-s3
24h-s1
24h-s2
24h-s3

Fig. S1.

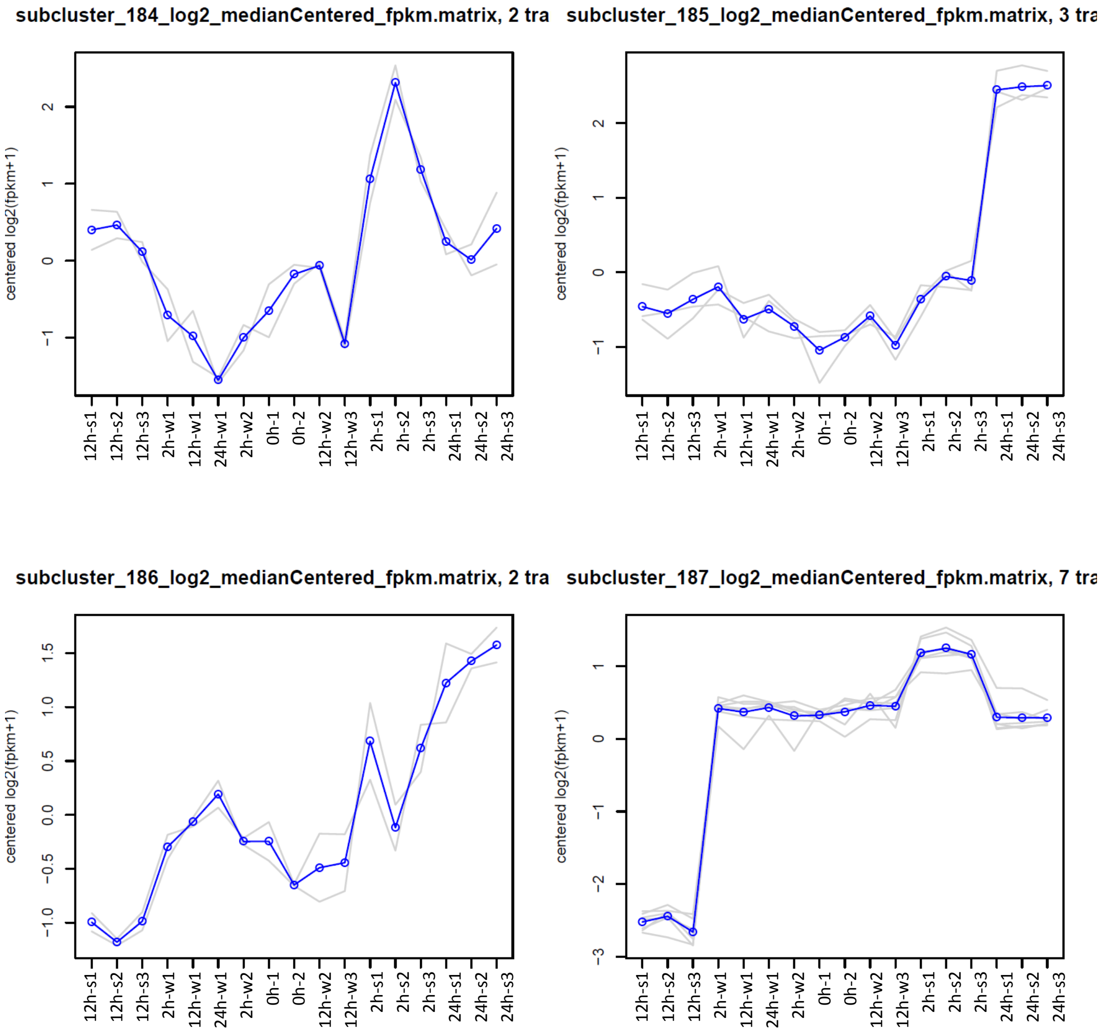
subcluster_184_log2_medianCentered_fpkm.matrix, 2 tra
subcluster_185_log2_medianCentered_fpkm.matrix, 3 tra
subcluster_186_log2_medianCentered_fpkm.matrix, 2 tra
subcluster_187_log2_medianCentered_fpkm.matrix, 7 tra
centered log2(fpkm+1)
12h-s1 12h-s2 12h-s3 2h-w1 12h-w1 24h-w1 2h-w2 0h-1 0h-2 12h-w2 12h-w3 2h-s1 2h-s2 2h-s3 24h-s1 24h-s2 24h-s3

Fig. S1.

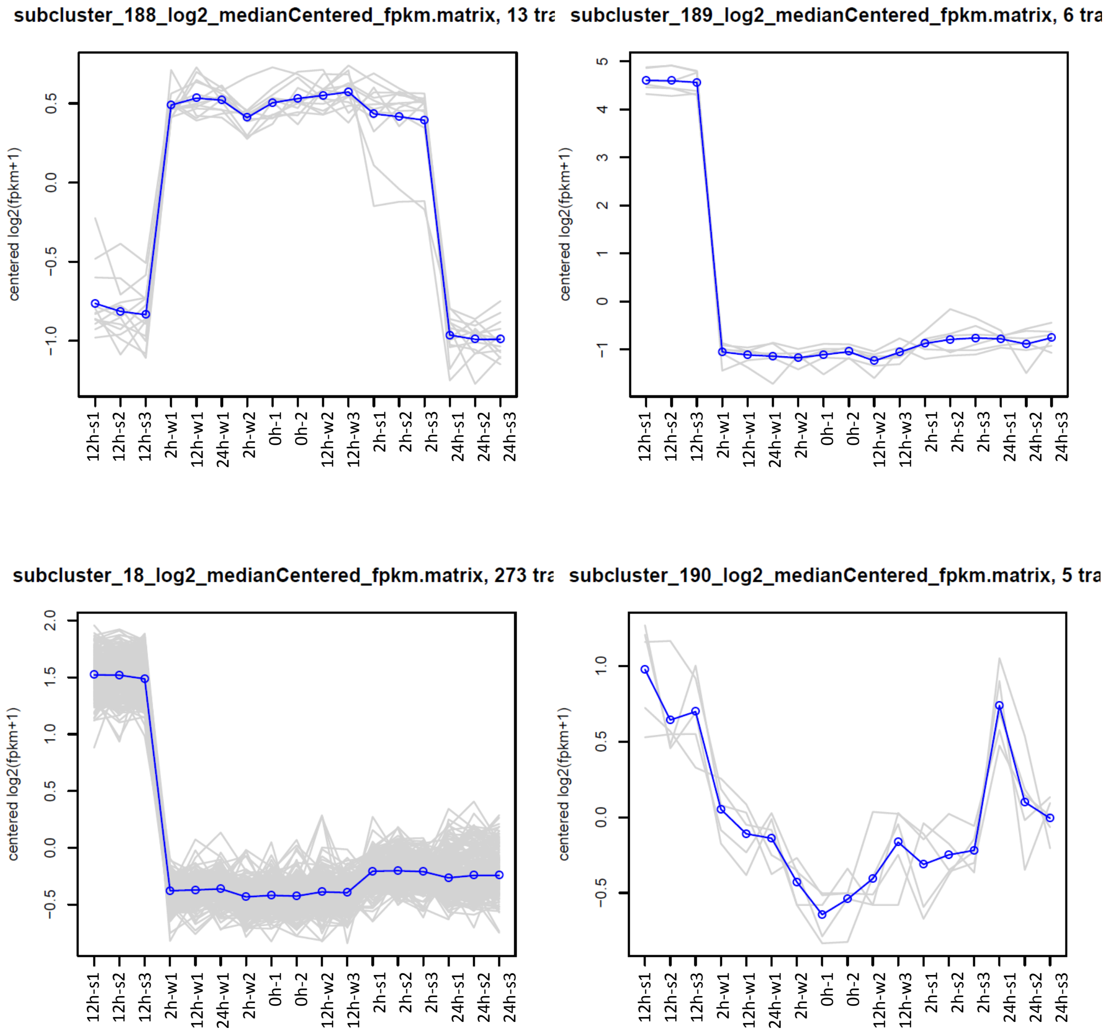
subcluster_188_log2_medianCentered_fpkm.matrix, 13 tra
centered log2(fpkm+1)
subcluster_189_log2_medianCentered_fpkm.matrix, 6 tra
centered log2(fpkm+1)
subcluster_18_log2_medianCentered_fpkm.matrix, 273 tra
centered log2(fpkm+1)
subcluster_190_log2_medianCentered_fpkm.matrix, 5 tra
centered log2(fpkm+1)
12h-s1 12h-s2 12h-s3 2h-w1 12h-w1 24h-w1 2h-w2 0h-1 0h-2 12h-w2 12h-w3 2h-s1 2h-s2 2h-s3 24h-s1 24h-s2 24h-s3

Fig. S1.

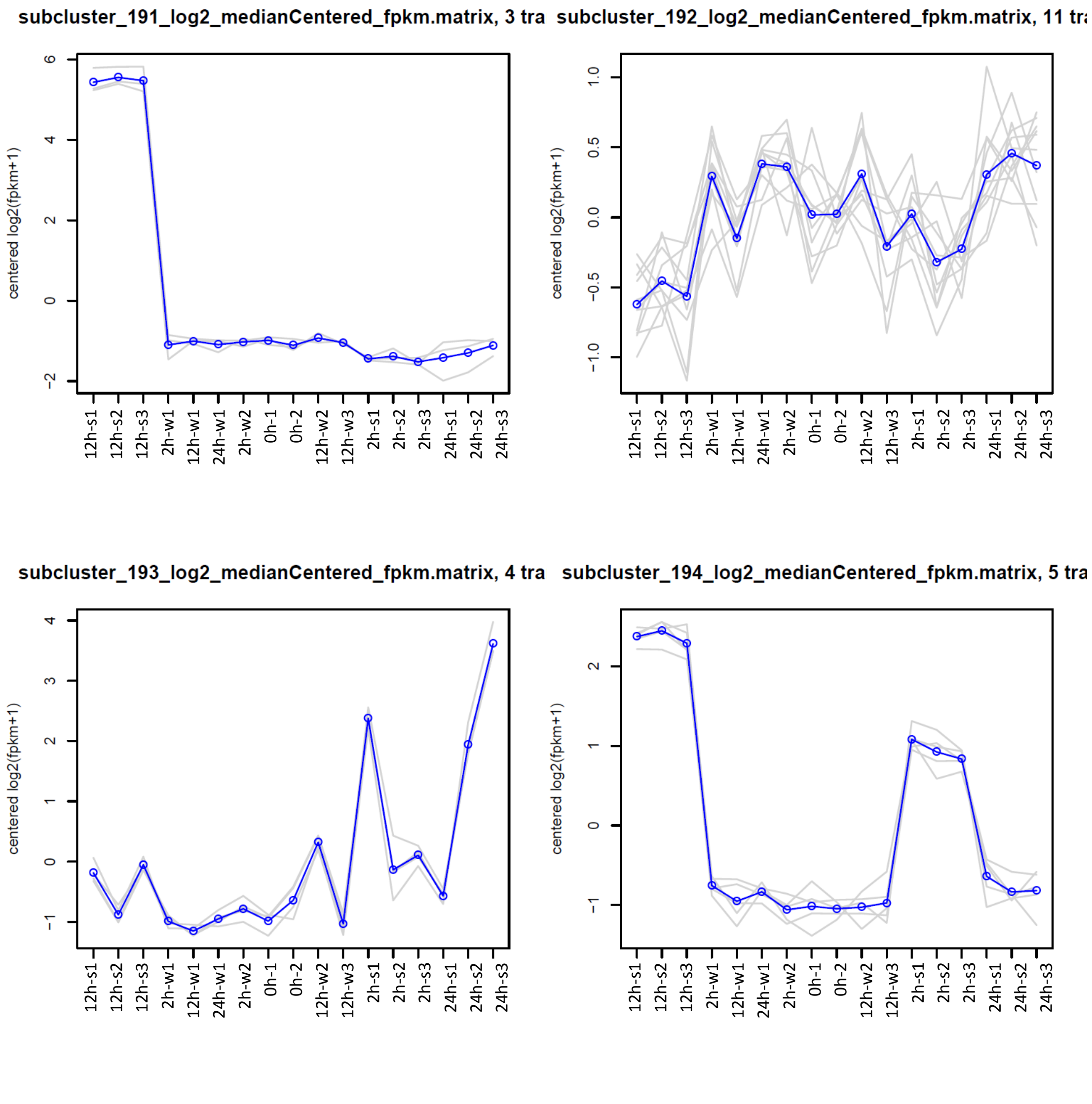
subcluster_191_log2_medianCentered_fpkm.matrix, 3 tra
subcluster_192_log2_medianCentered_fpkm.matrix, 11 tra
subcluster_193_log2_medianCentered_fpkm.matrix, 4 tra
subcluster_194_log2_medianCentered_fpkm.matrix, 5 tra
centered log2(fpkm+1)
12h-s1
12h-s2
12h-s3
2h-w1
12h-w1
24h-w1
2h-w2
0h-1
0h-2
12h-w2
12h-w3
2h-s1
2h-s2
2h-s3
24h-s1
24h-s2
24h-s3

Fig. S1.

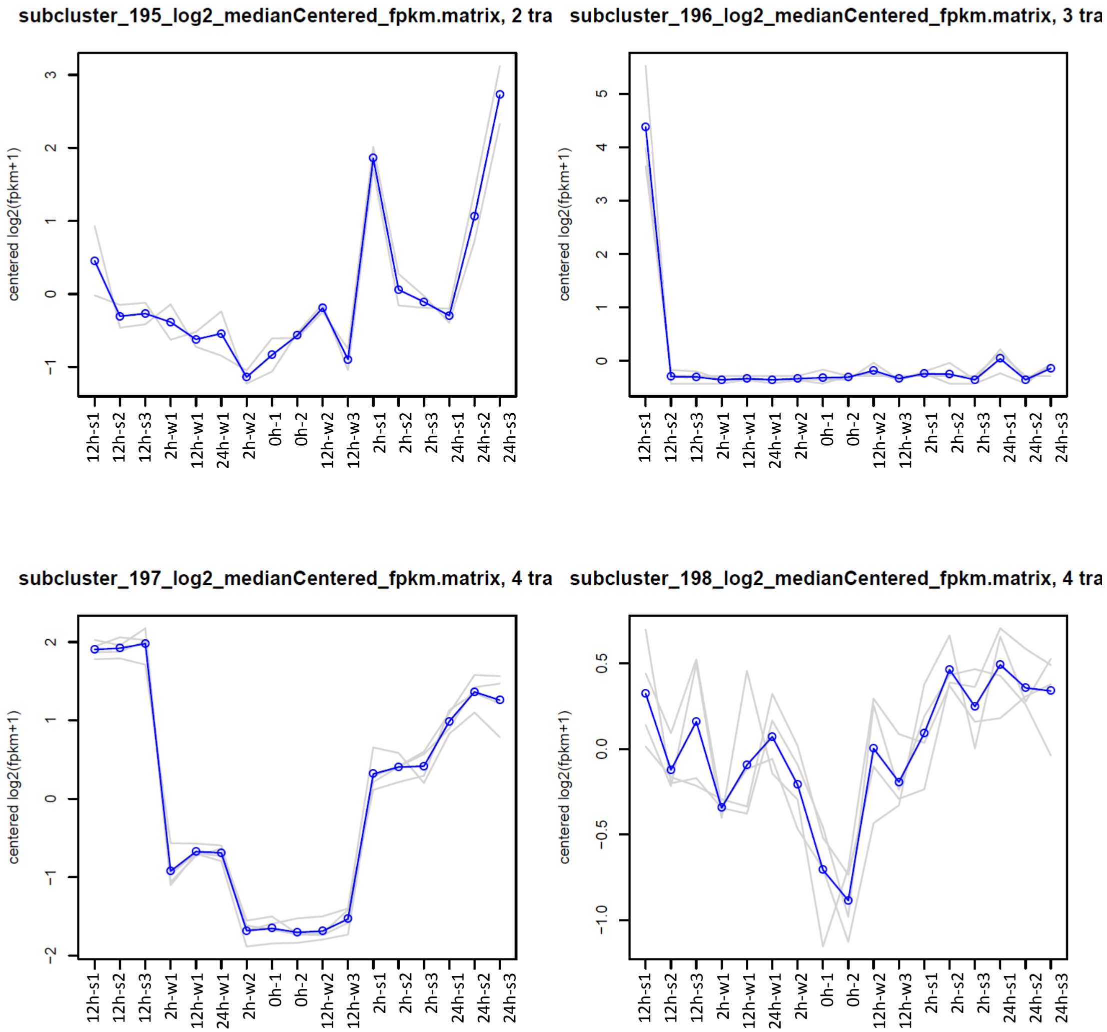

subcluster_195_log2_medianCentered_fpkm.matrix, 2 tra
subcluster_196_log2_medianCentered_fpkm.matrix, 3 tra
subcluster_197_log2_medianCentered_fpkm.matrix, 4 tra
subcluster_198_log2_medianCentered_fpkm.matrix, 4 tra
centered log2(fpkm+1)
12h-s1
12h-s2
12h-s3
2h-w1
12h-w1
24h-w1
2h-w2
0h-1
0h-2
12h-w2
12h-w3
2h-s1
2h-s2
2h-s3
24h-s1
24h-s2
24h-s3

Fig. S1.

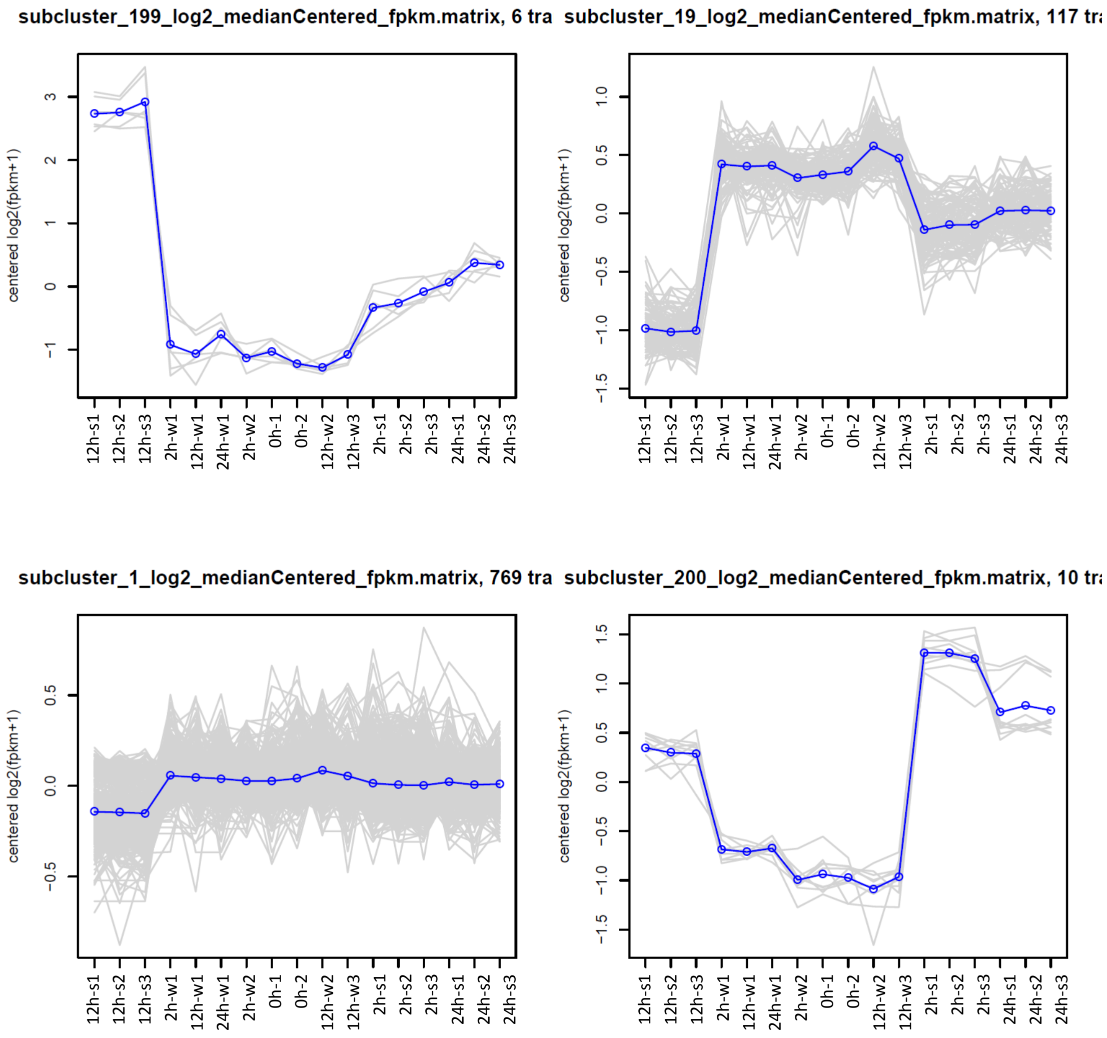
subcluster_199_log2_medianCentered_fpkm.matrix, 6 tra
subcluster_19_log2_medianCentered_fpkm.matrix, 117 tr
subcluster_1_log2_medianCentered_fpkm.matrix, 769 tra
subcluster_200_log2_medianCentered_fpkm.matrix, 10 tr
centered log2(fpkm+1)
12h-s1
12h-s2
12h-s3
2h-w1
12h-w1
24h-w1
2h-w2
0h-1
0h-2
12h-w2
12h-w3
2h-s1
2h-s2
2h-s3
24h-s1
24h-s2
24h-s3

Fig. S1.

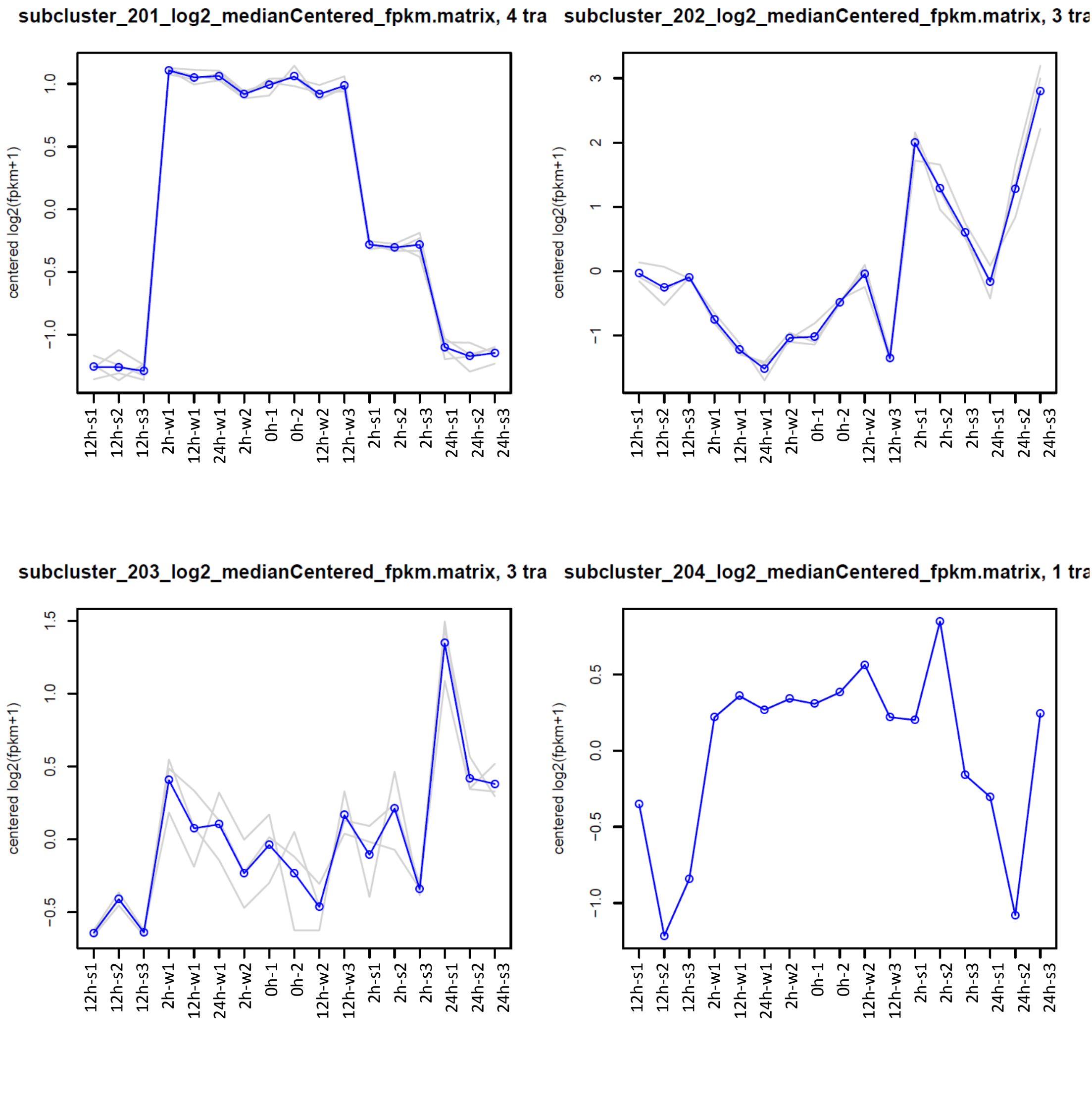
subcluster_201_log2_medianCentered_fpkm.matrix, 4 tra
subcluster_202_log2_medianCentered_fpkm.matrix, 3 tra
subcluster_203_log2_medianCentered_fpkm.matrix, 3 tra
subcluster_204_log2_medianCentered_fpkm.matrix, 1 tra
centered log2(fpkm+1)
12h-s1
12h-s2
12h-s3
2h-w1
12h-w1
24h-w1
2h-w2
0h-1
0h-2
12h-w2
12h-w3
2h-s1
2h-s2
2h-s3
24h-s1
24h-s2
24h-s3

Fig. S1.

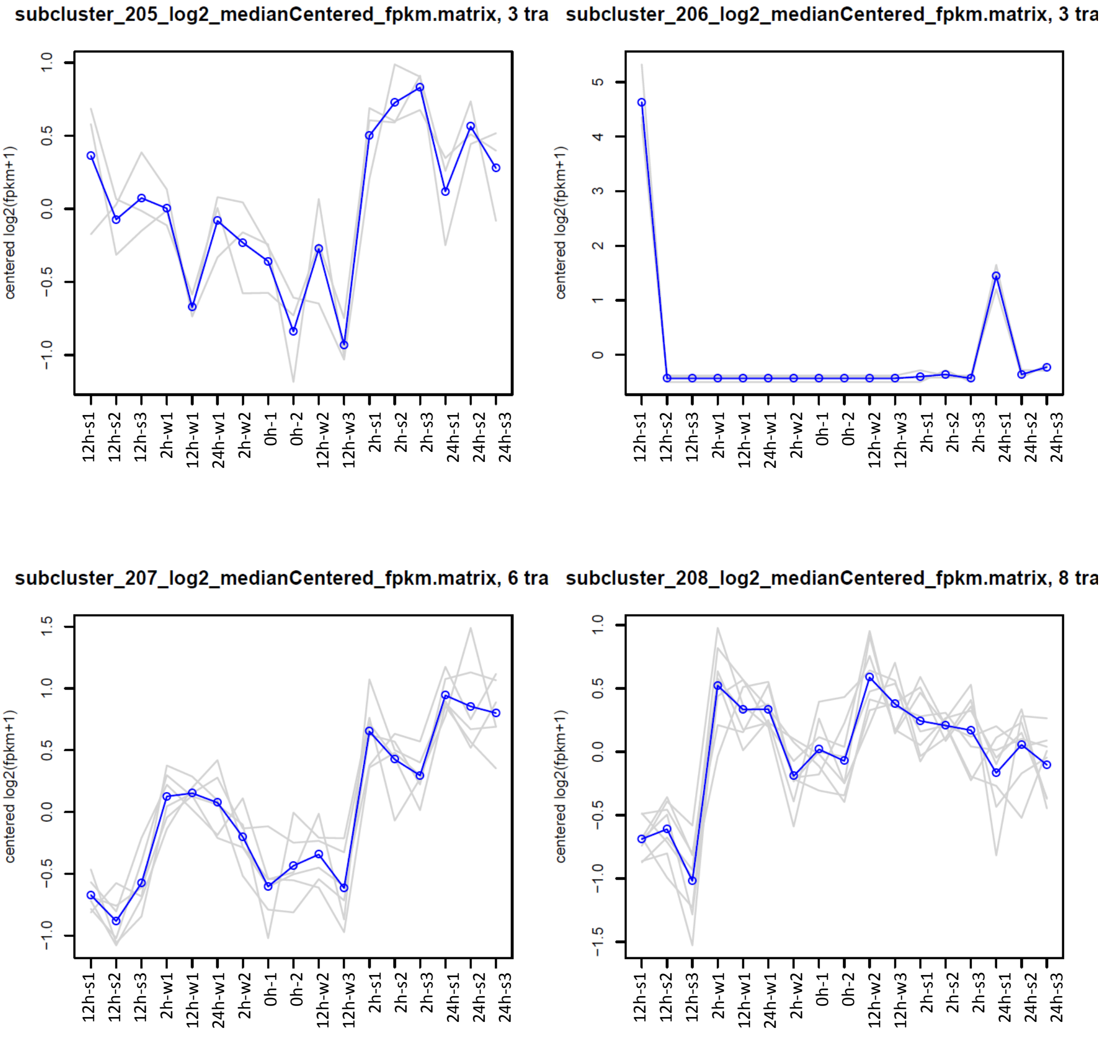
subcluster_205_log2_medianCentered_fpkm.matrix, 3 tra
subcluster_206_log2_medianCentered_fpkm.matrix, 3 tra
subcluster_207_log2_medianCentered_fpkm.matrix, 6 tra
subcluster_208_log2_medianCentered_fpkm.matrix, 8 tra
centered log2(fpkm+1)
12h-s1
12h-s2
12h-s3
2h-w1
12h-w1
24h-w1
2h-w2
0h-1
0h-2
12h-w2
12h-w3
2h-s1
2h-s2
2h-s3
24h-s1
24h-s2
24h-s3

Fig. S1.

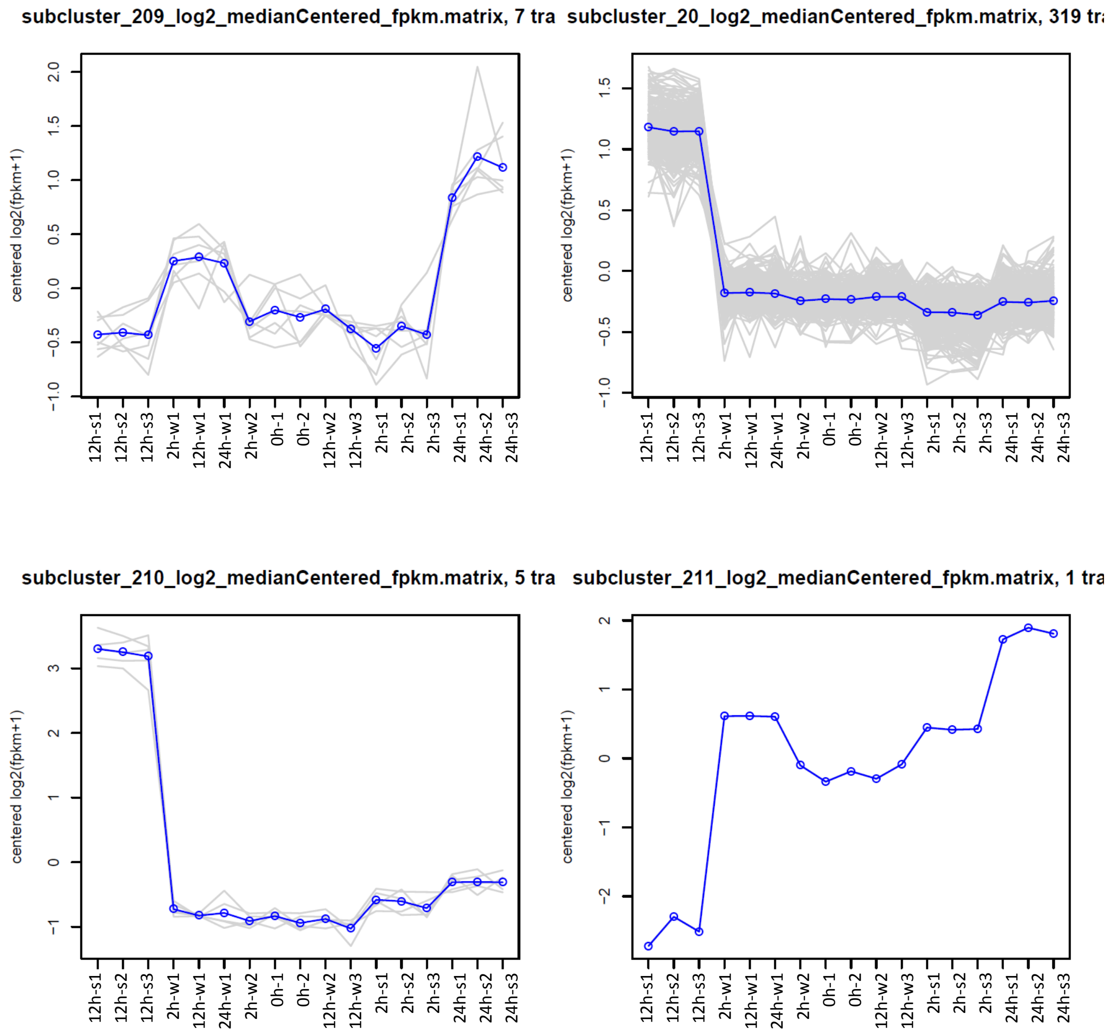
subcluster_209_log2_medianCentered_fpkm.matrix, 7 tra
subcluster_20_log2_medianCentered_fpkm.matrix, 319 tr
subcluster_210_log2_medianCentered_fpkm.matrix, 5 tra
subcluster_211_log2_medianCentered_fpkm.matrix, 1 tra
centered log2(fpkm+1)
12h-s1
12h-s2
12h-s3
2h-w1
12h-w1
24h-w1
2h-w2
0h-1
0h-2
12h-w2
12h-w3
2h-s1
2h-s2
2h-s3
24h-s1
24h-s2
24h-s3

Fig. S1.

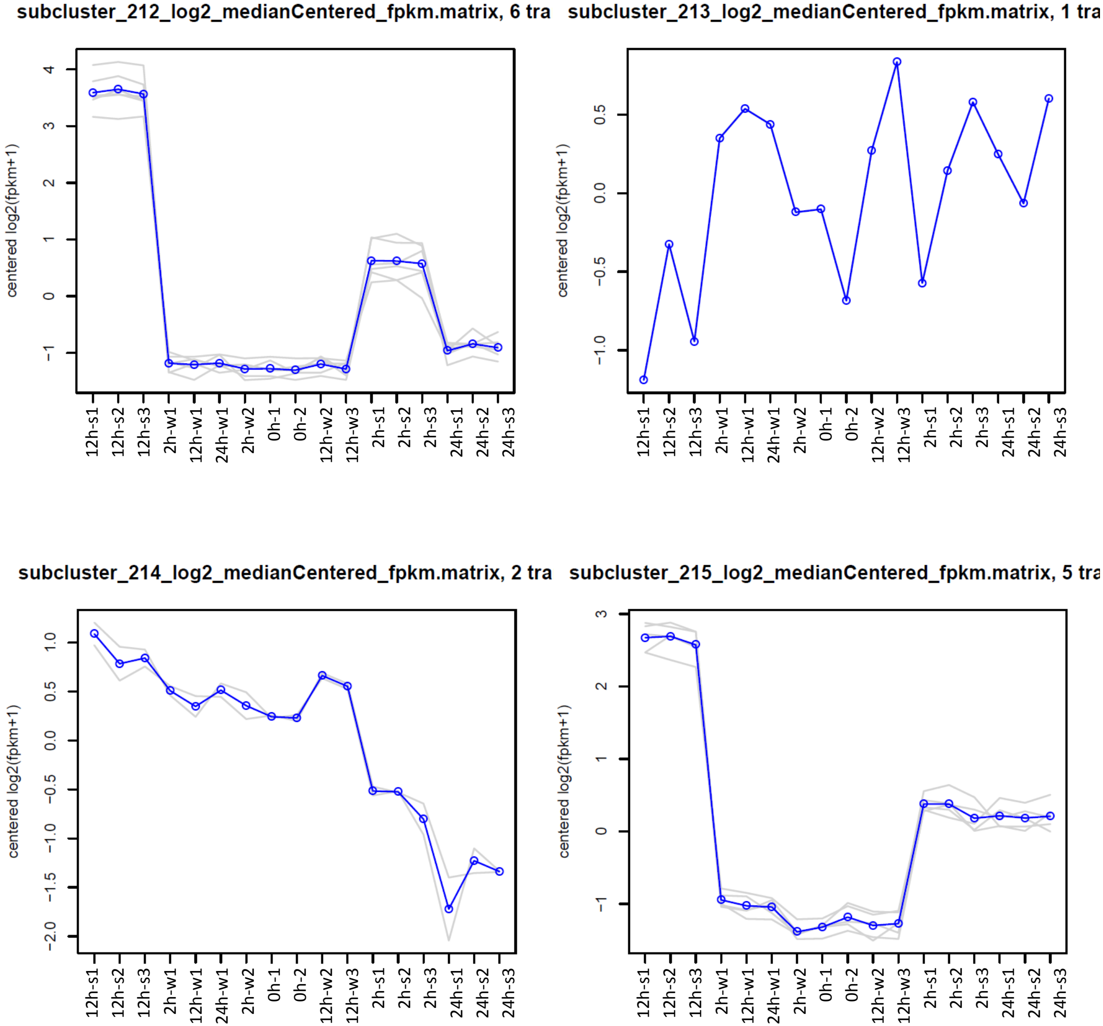
subcluster_212_log2_medianCentered_fpkm.matrix, 6 tra
subcluster_213_log2_medianCentered_fpkm.matrix, 1 tra
subcluster_214_log2_medianCentered_fpkm.matrix, 2 tra
subcluster_215_log2_medianCentered_fpkm.matrix, 5 tra
centered log2(fpkm+1)
12h-s1
12h-s2
12h-s3
2h-w1
12h-w1
24h-w1
2h-w2
0h-1
0h-2
12h-w2
12h-w3
2h-s1
2h-s2
2h-s3
24h-s1
24h-s2
24h-s3

Fig. S1.

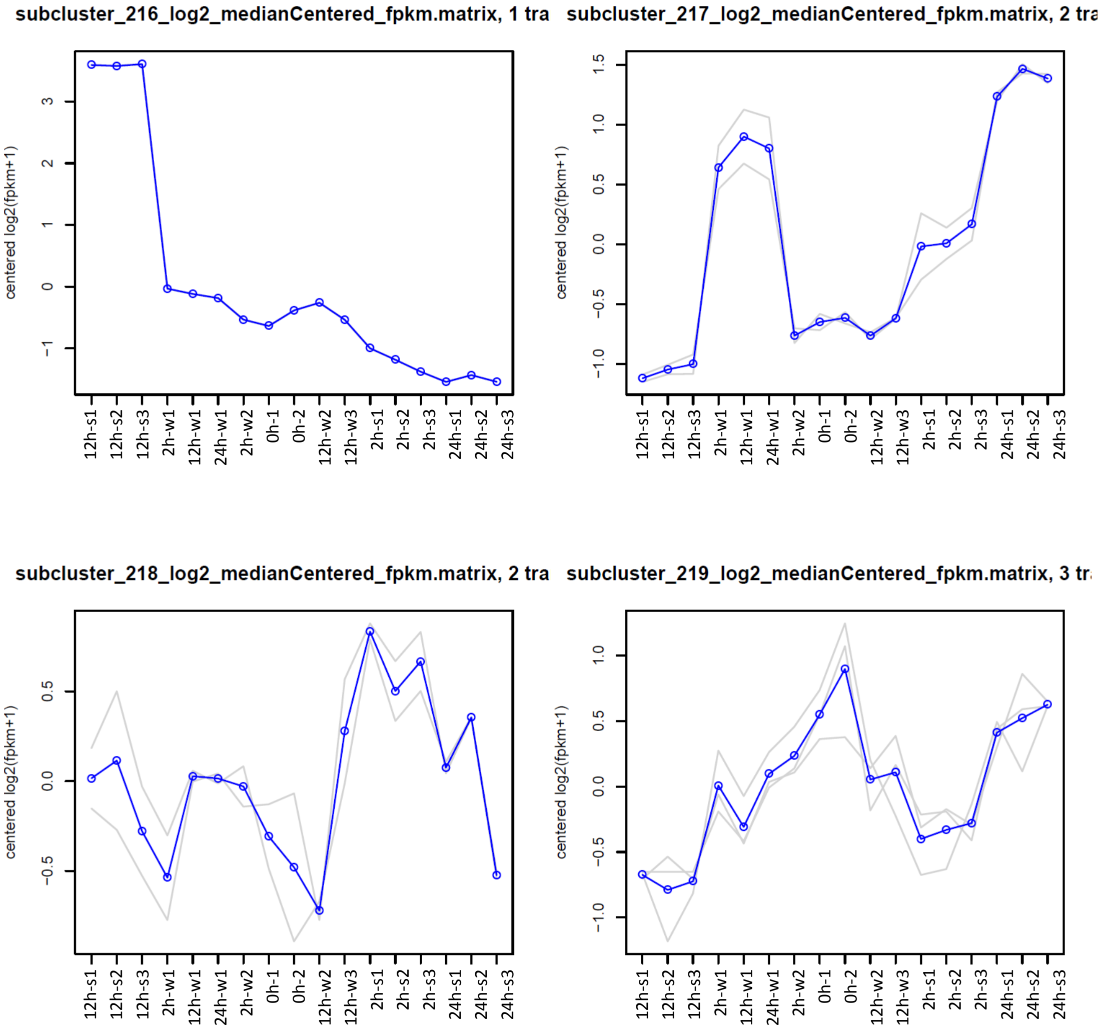
subcluster_216_log2_medianCentered_fpkm.matrix, 1 tra
subcluster_217_log2_medianCentered_fpkm.matrix, 2 tra
subcluster_218_log2_medianCentered_fpkm.matrix, 2 tra
subcluster_219_log2_medianCentered_fpkm.matrix, 3 tr
centered log2(fpkm+1)
12h-s1
12h-s2
12h-s3
2h-w1
12h-w1
24h-w1
2h-w2
0h-1
0h-2
12h-w2
12h-w3
2h-s1
2h-s2
2h-s3
24h-s1
24h-s2
24h-s3

Fig. S1.

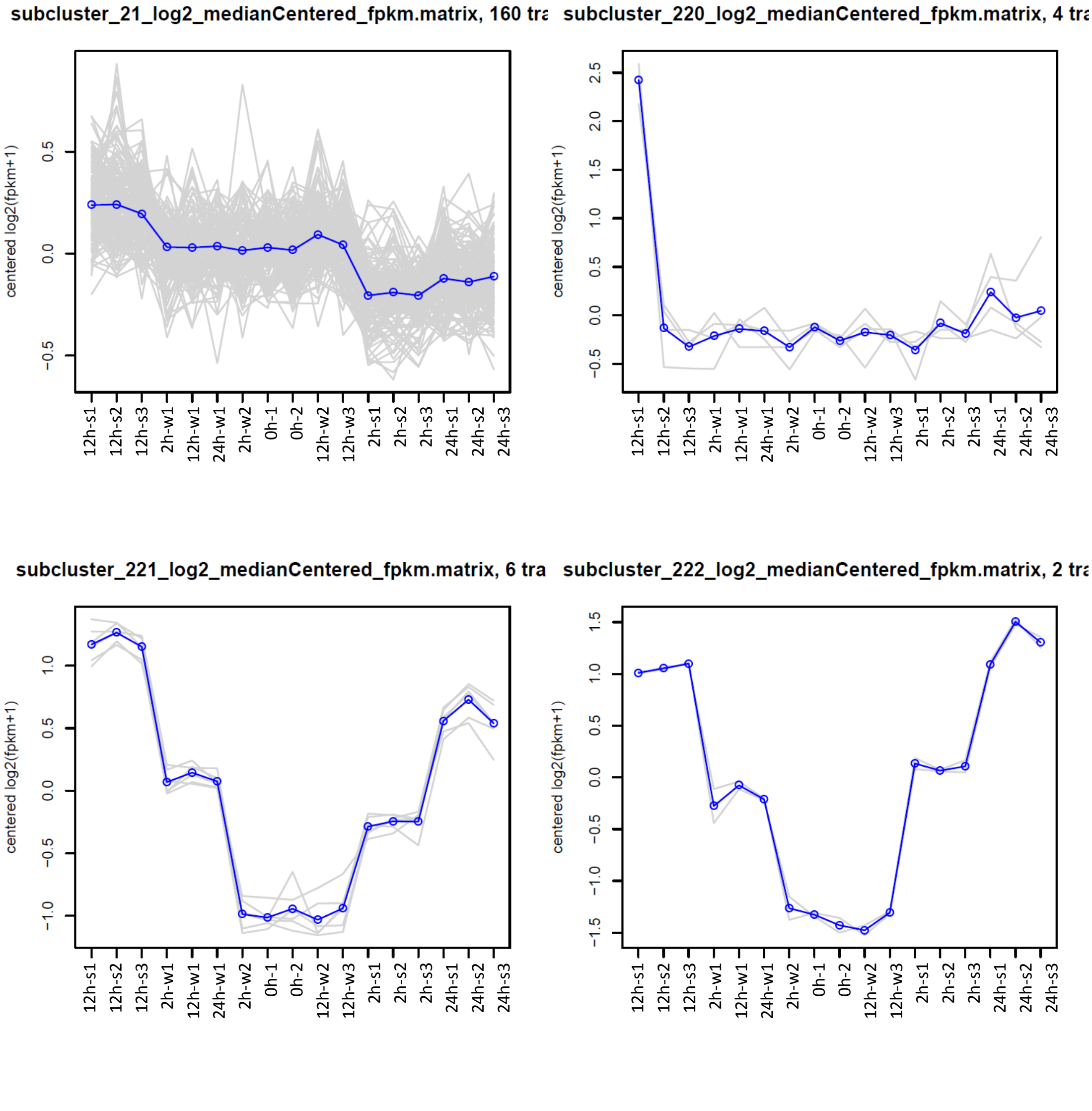
subcluster_21_log2_medianCentered_fpkm.matrix, 160 tra
subcluster_220_log2_medianCentered_fpkm.matrix, 4 tra
subcluster_221_log2_medianCentered_fpkm.matrix, 6 tra
subcluster_222_log2_medianCentered_fpkm.matrix, 2 tra
centered log2(fpkm+1)
12h-s1
12h-s2
12h-s3
2h-w1
12h-w1
24h-w1
2h-w2
0h-1
0h-2
12h-w2
12h-w3
2h-s1
2h-s2
2h-s3
24h-s1
24h-s2
24h-s3

Fig. S1.

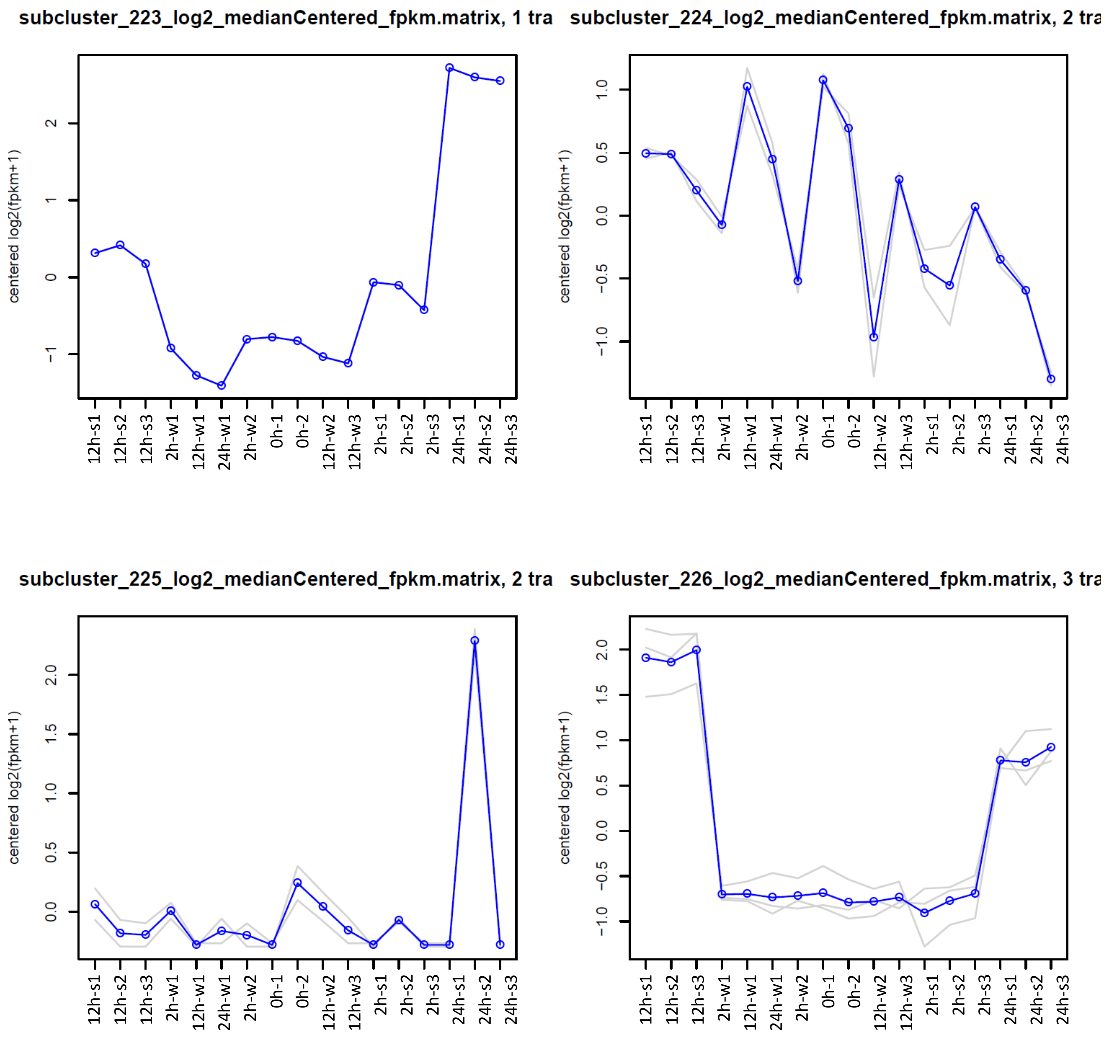
subcluster_223_log2_medianCentered_fpkm.matrix, 1 tra
subcluster_224_log2_medianCentered_fpkm.matrix, 2 tra
subcluster_225_log2_medianCentered_fpkm.matrix, 2 tra
subcluster_226_log2_medianCentered_fpkm.matrix, 3 tra
centered log2(fpkm+1)
12h-s1
12h-s2
12h-s3
2h-w1
12h-w1
24h-w1
2h-w2
0h-1
0h-2
12h-w2
12h-w3
2h-s1
2h-s2
2h-s3
24h-s1
24h-s2
24h-s3

Fig. S1.

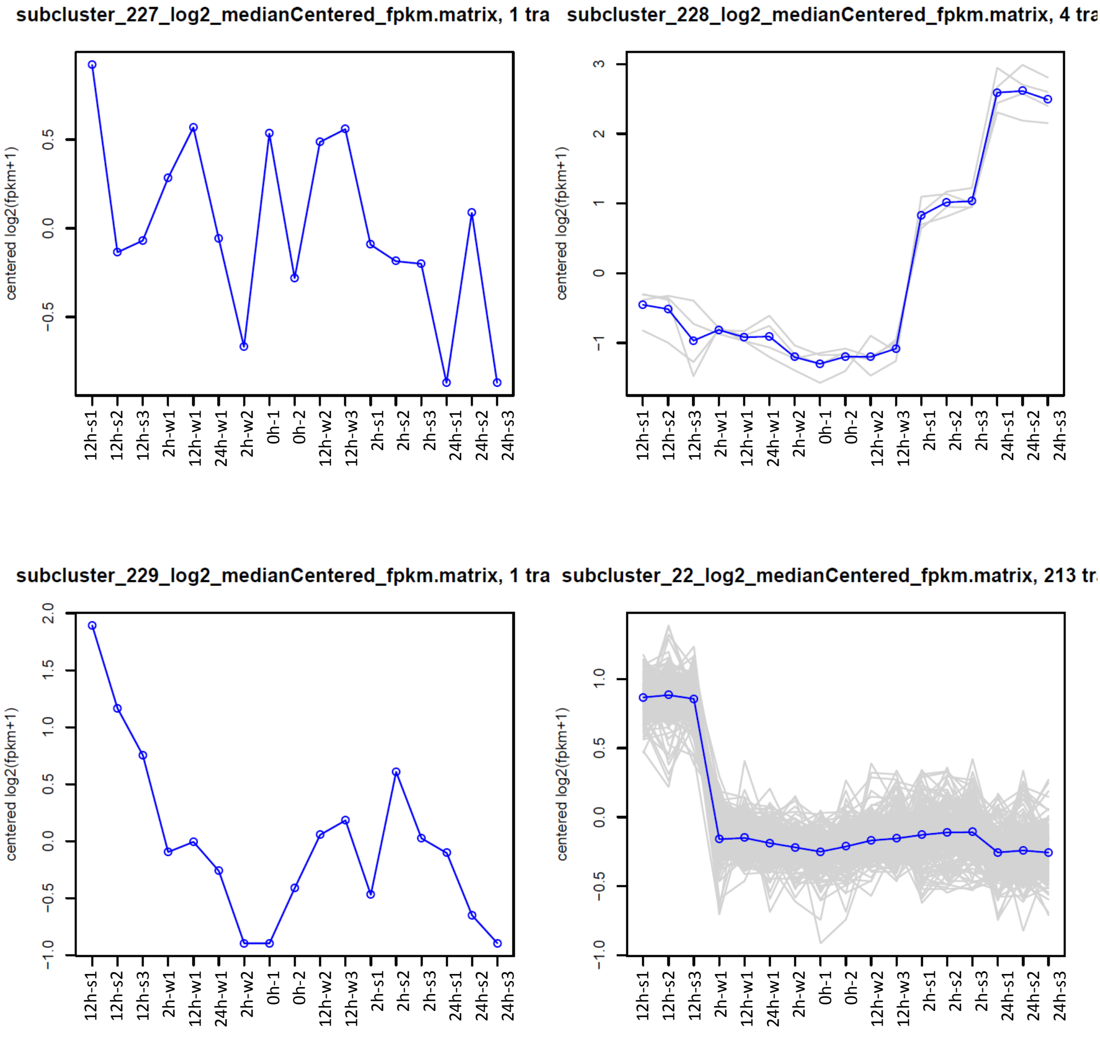
subcluster_227_log2_medianCentered_fpkm.matrix, 1 tra
subcluster_228_log2_medianCentered_fpkm.matrix, 4 tra
subcluster_229_log2_medianCentered_fpkm.matrix, 1 tra
subcluster_22_log2_medianCentered_fpkm.matrix, 213 tr
centered log2(fpkm+1)
12h-s1
12h-s2
12h-s3
2h-w1
12h-w1
24h-w1
2h-w2
0h-1
0h-2
12h-w2
12h-w3
2h-s1
2h-s2
2h-s3
24h-s1
24h-s2
24h-s3

Fig. S1.

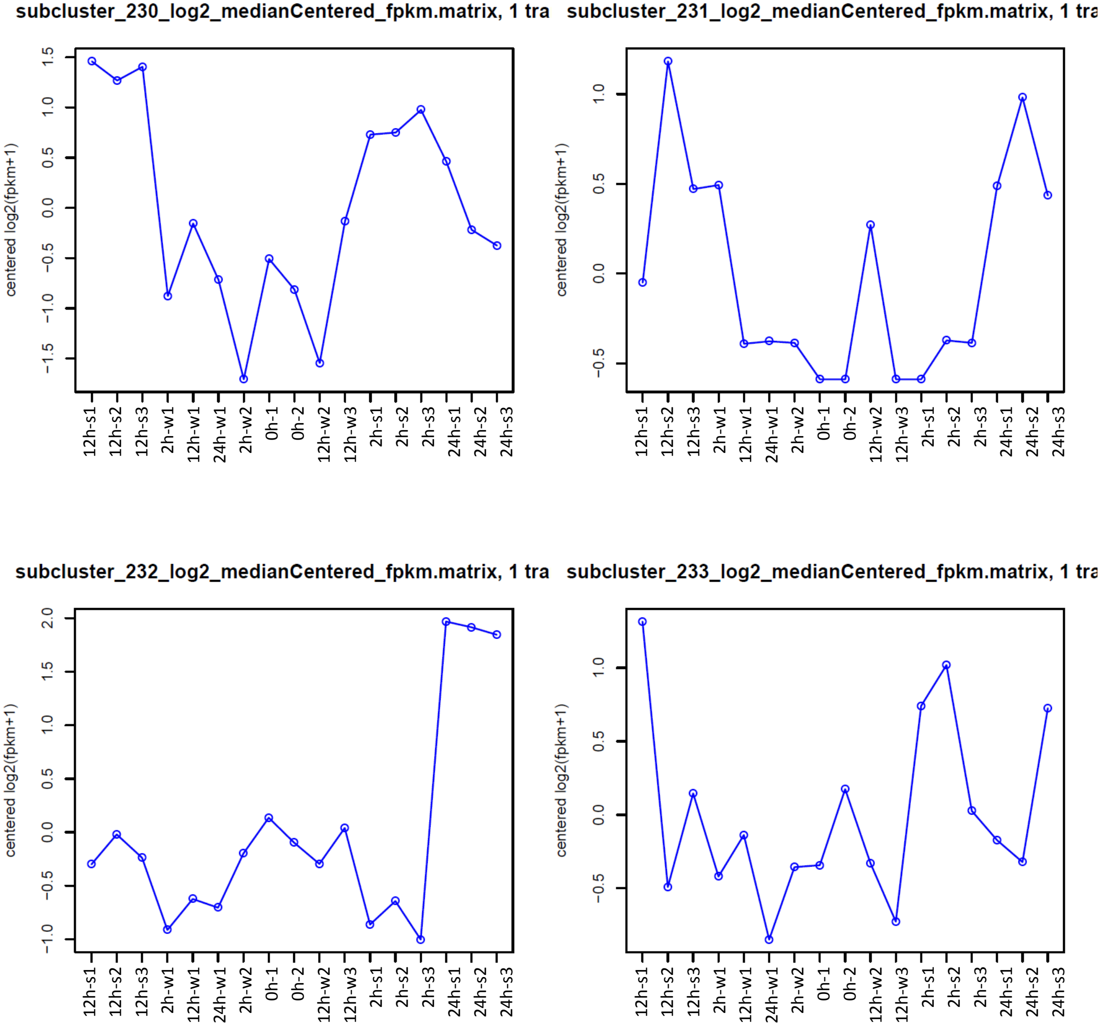
subcluster_230_log2_medianCentered_fpkm.matrix, 1 tra
subcluster_231_log2_medianCentered_fpkm.matrix, 1 tra
subcluster_232_log2_medianCentered_fpkm.matrix, 1 tra
subcluster_233_log2_medianCentered_fpkm.matrix, 1 tra
centered log2(fpkm+1)
12h-s1 12h-s2 12h-s3 2h-w1 12h-w1 24h-w1 2h-w2 0h-1 0h-2 12h-w2 12h-w3 2h-s1 2h-s2 2h-s3 24h-s1 24h-s2 24h-s3

Fig. S1.

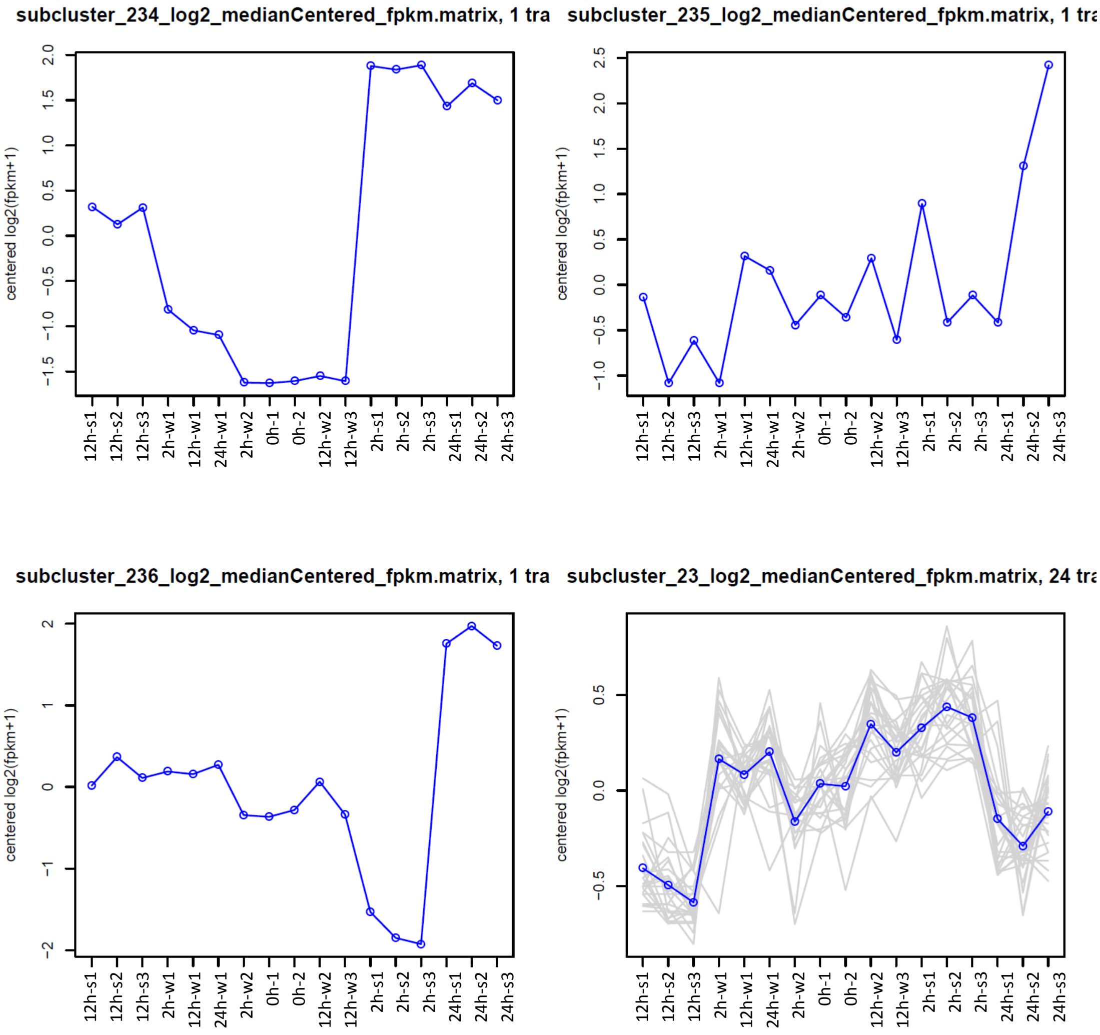
subcluster_234_log2_medianCentered_fpkm.matrix, 1 tra
subcluster_235_log2_medianCentered_fpkm.matrix, 1 tra
subcluster_236_log2_medianCentered_fpkm.matrix, 1 tra
subcluster_23_log2_medianCentered_fpkm.matrix, 24 tra
centered log2(fpkm+1)
12h-s1
12h-s2
12h-s3
2h-w1
12h-w1
24h-w1
2h-w2
0h-1
0h-2
12h-w2
12h-w3
2h-s1
2h-s2
2h-s3
24h-s1
24h-s2
24h-s3

Fig. S1.

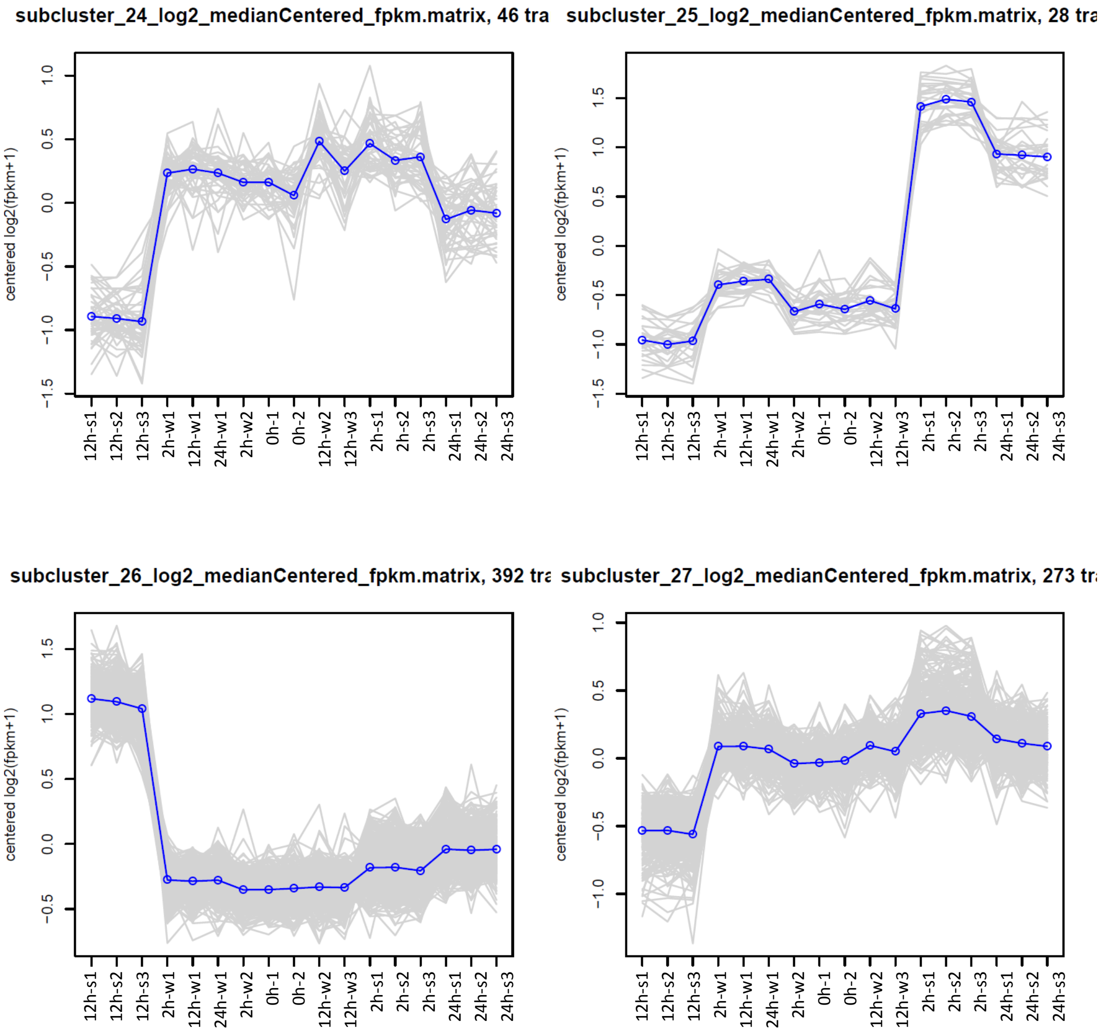
subcluster_24_log2_medianCentered_fpkm.matrix, 46 tra
subcluster_25_log2_medianCentered_fpkm.matrix, 28 tra
subcluster_26_log2_medianCentered_fpkm.matrix, 392 tra
subcluster_27_log2_medianCentered_fpkm.matrix, 273 tr
centered log2(fpkm+1)
12h-s1
12h-s2
12h-s3
2h-w1
12h-w1
24h-w1
2h-w2
0h-1
0h-2
12h-w2
12h-w3
2h-s1
2h-s2
2h-s3
24h-s1
24h-s2
24h-s3

Fig. S1.

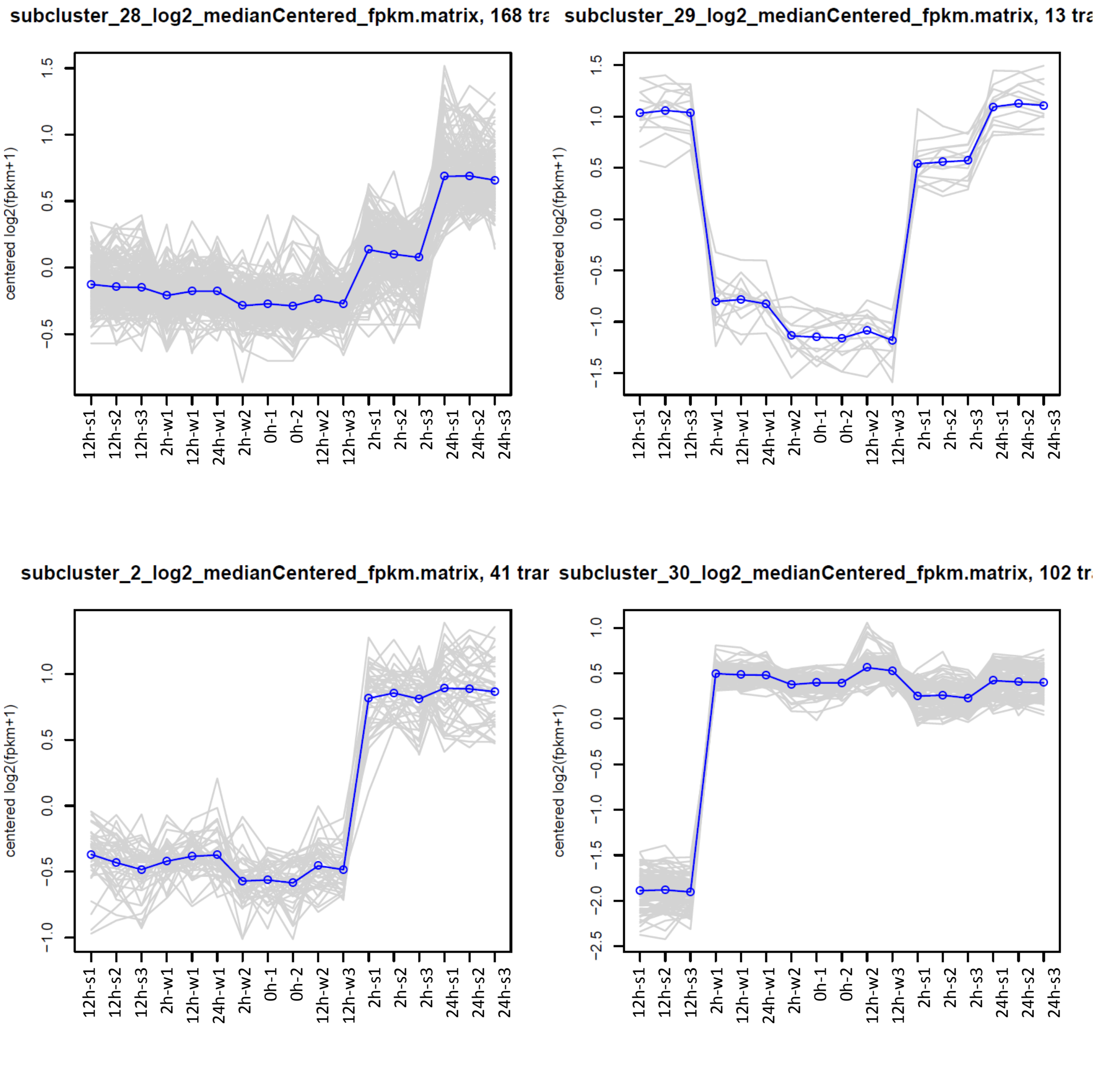
subcluster_28_log2_medianCentered_fpkm.matrix, 168 tra
subcluster_29_log2_medianCentered_fpkm.matrix, 13 tra
subcluster_2_log2_medianCentered_fpkm.matrix, 41 trar
subcluster_30_log2_medianCentered_fpkm.matrix, 102 tr
centered log2(fpkm+1)
12h-s1
12h-s2
12h-s3
2h-w1
12h-w1
24h-w1
2h-w2
0h-1
0h-2
12h-w2
12h-w3
2h-s1
2h-s2
2h-s3
24h-s1
24h-s2
24h-s3

Fig. S1.

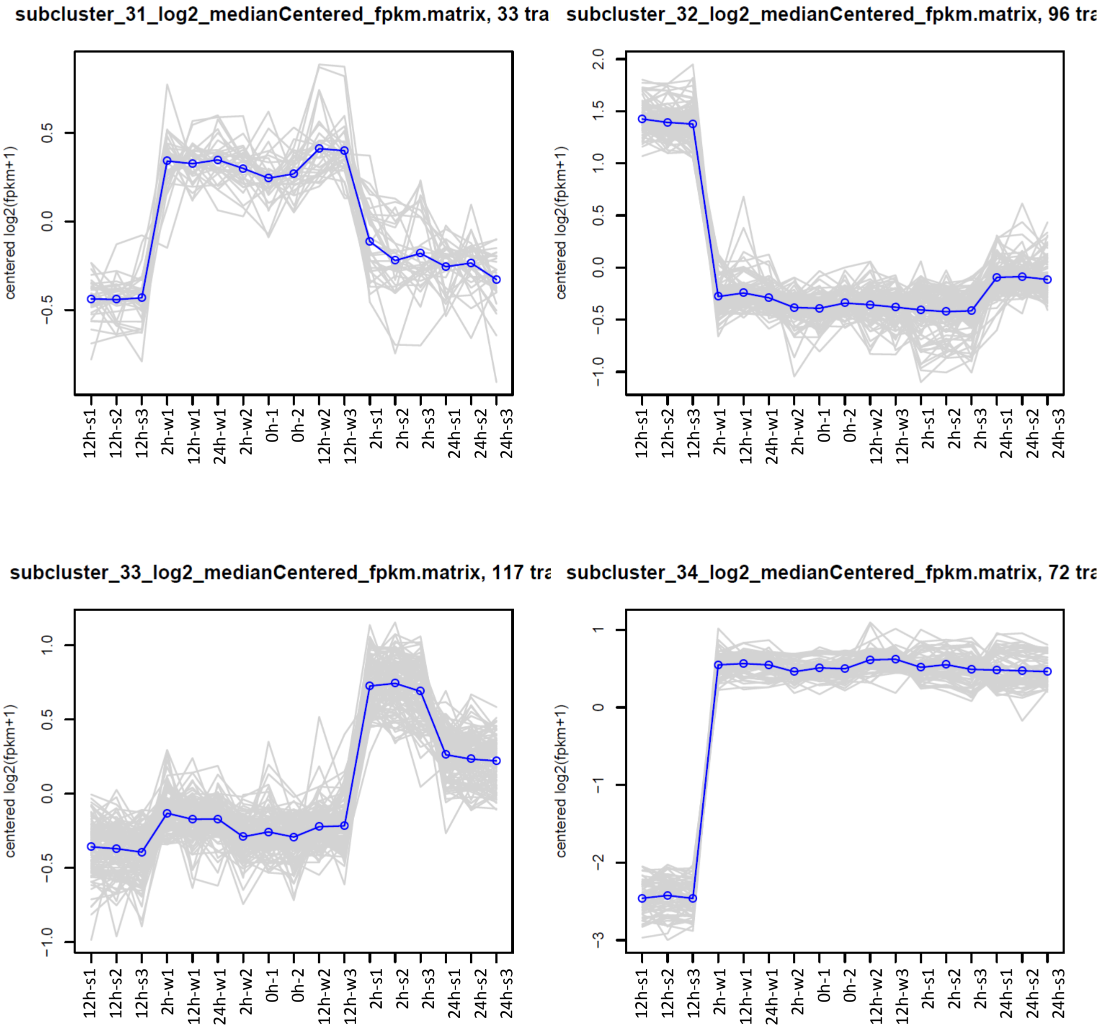

subcluster_31_log2_medianCentered_fpkm.matrix, 33 tra
subcluster_32_log2_medianCentered_fpkm.matrix, 96 tra
subcluster_33_log2_medianCentered_fpkm.matrix, 117 tra
subcluster_34_log2_medianCentered_fpkm.matrix, 72 tra
centered log2(fpkm+1)
12h-s1
12h-s2
12h-s3
2h-w1
12h-w1
24h-w1
2h-w2
0h-1
0h-2
12h-w2
12h-w3
2h-s1
2h-s2
2h-s3
24h-s1
24h-s2
24h-s3

Fig. S1.

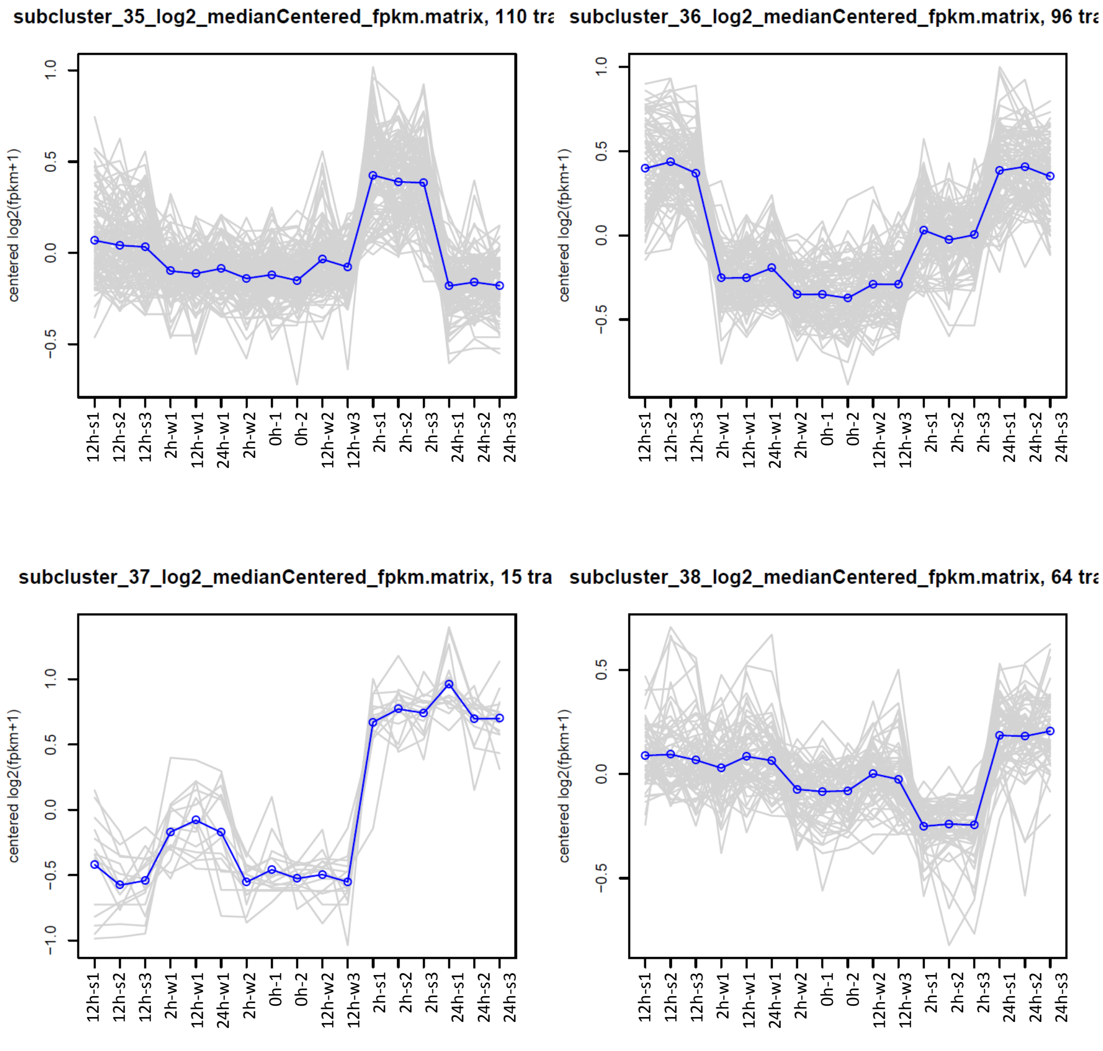
subcluster_35_log2_medianCentered_fpkm.matrix, 110 tra
subcluster_36_log2_medianCentered_fpkm.matrix, 96 tra
subcluster_37_log2_medianCentered_fpkm.matrix, 15 tra
subcluster_38_log2_medianCentered_fpkm.matrix, 64 tra
centered log2(fpkm+1)
12h-s1
12h-s2
12h-s3
2h-w1
12h-w1
24h-w1
2h-w2
0h-1
0h-2
12h-w2
12h-w3
2h-s1
2h-s2
2h-s3
24h-s1
24h-s2
24h-s3

Fig. S1.

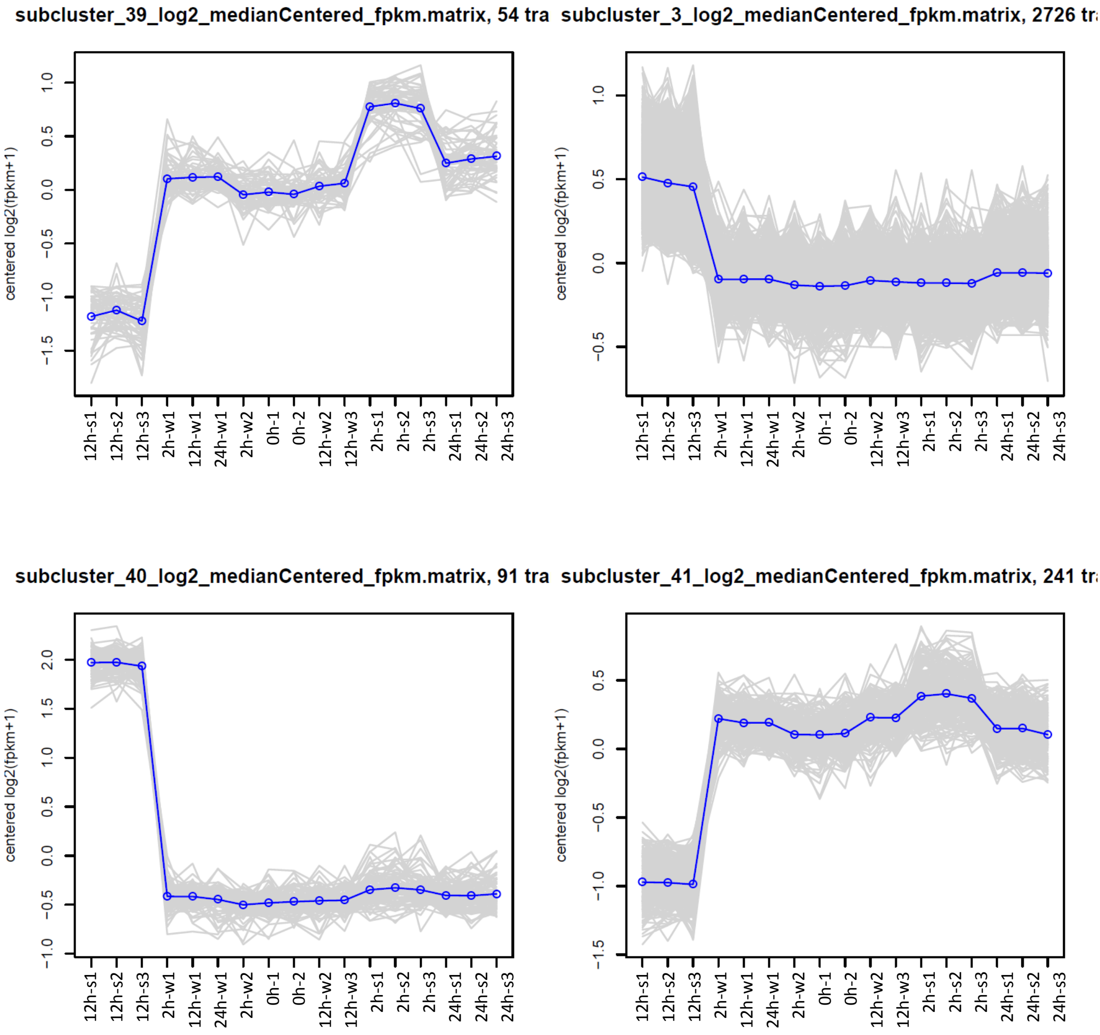
subcluster_39_log2_medianCentered_fpkm.matrix, 54 tra
subcluster_3_log2_medianCentered_fpkm.matrix, 2726 tr
subcluster_40_log2_medianCentered_fpkm.matrix, 91 tra
subcluster_41_log2_medianCentered_fpkm.matrix, 241 tr
centered log2(fpkm+1)
12h-s1
12h-s2
12h-s3
2h-w1
12h-w1
24h-w1
2h-w2
0h-1
0h-2
12h-w2
12h-w3
2h-s1
2h-s2
2h-s3
24h-s1
24h-s2
24h-s3

Fig. S1.

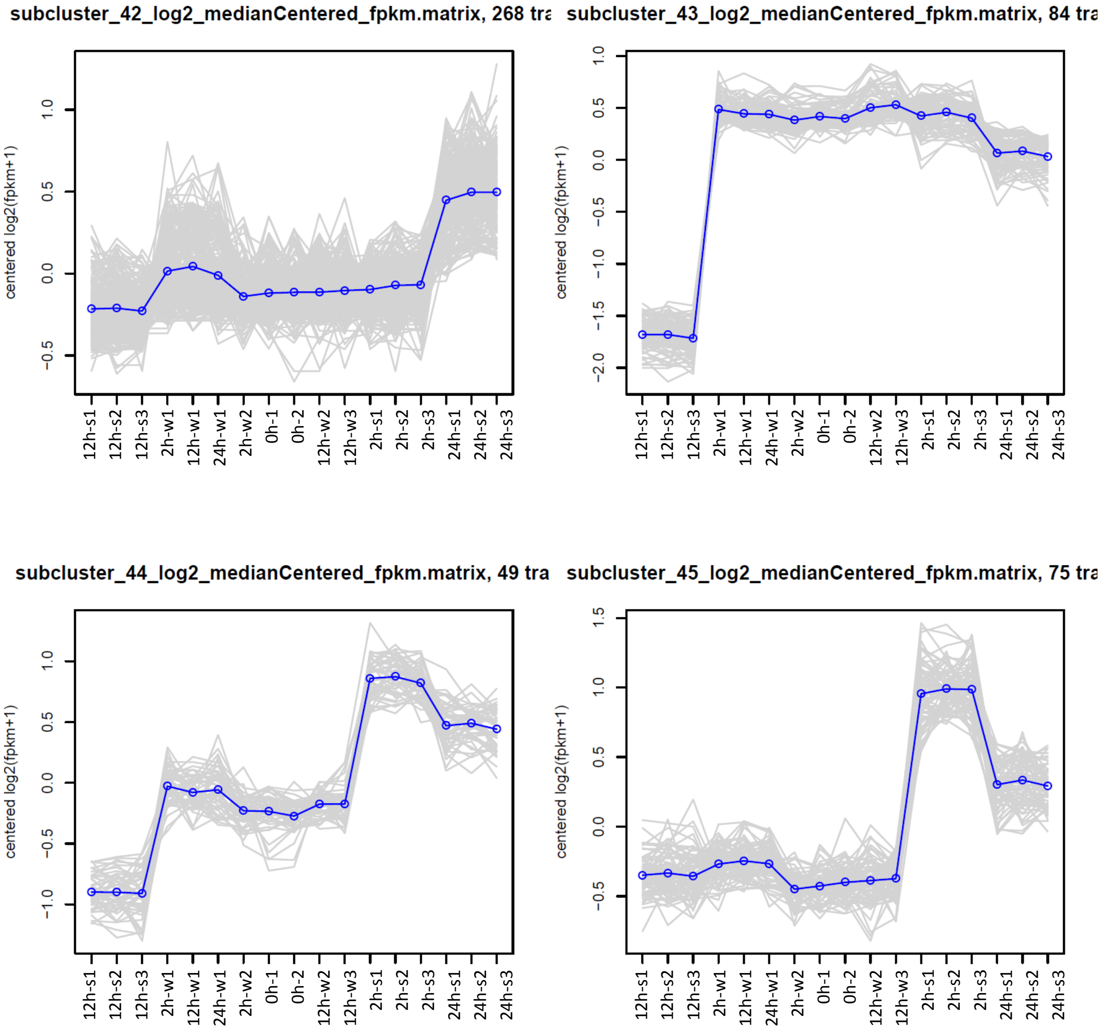
subcluster_42_log2_medianCentered_fpkm.matrix, 268 tra
subcluster_43_log2_medianCentered_fpkm.matrix, 84 tra
subcluster_44_log2_medianCentered_fpkm.matrix, 49 tra
subcluster_45_log2_medianCentered_fpkm.matrix, 75 tra
centered log2(fpkm+1)
12h-s1
12h-s2
12h-s3
2h-w1
12h-w1
24h-w1
2h-w2
0h-1
0h-2
12h-w2
12h-w3
2h-s1
2h-s2
2h-s3
24h-s1
24h-s2
24h-s3

Fig. S1.

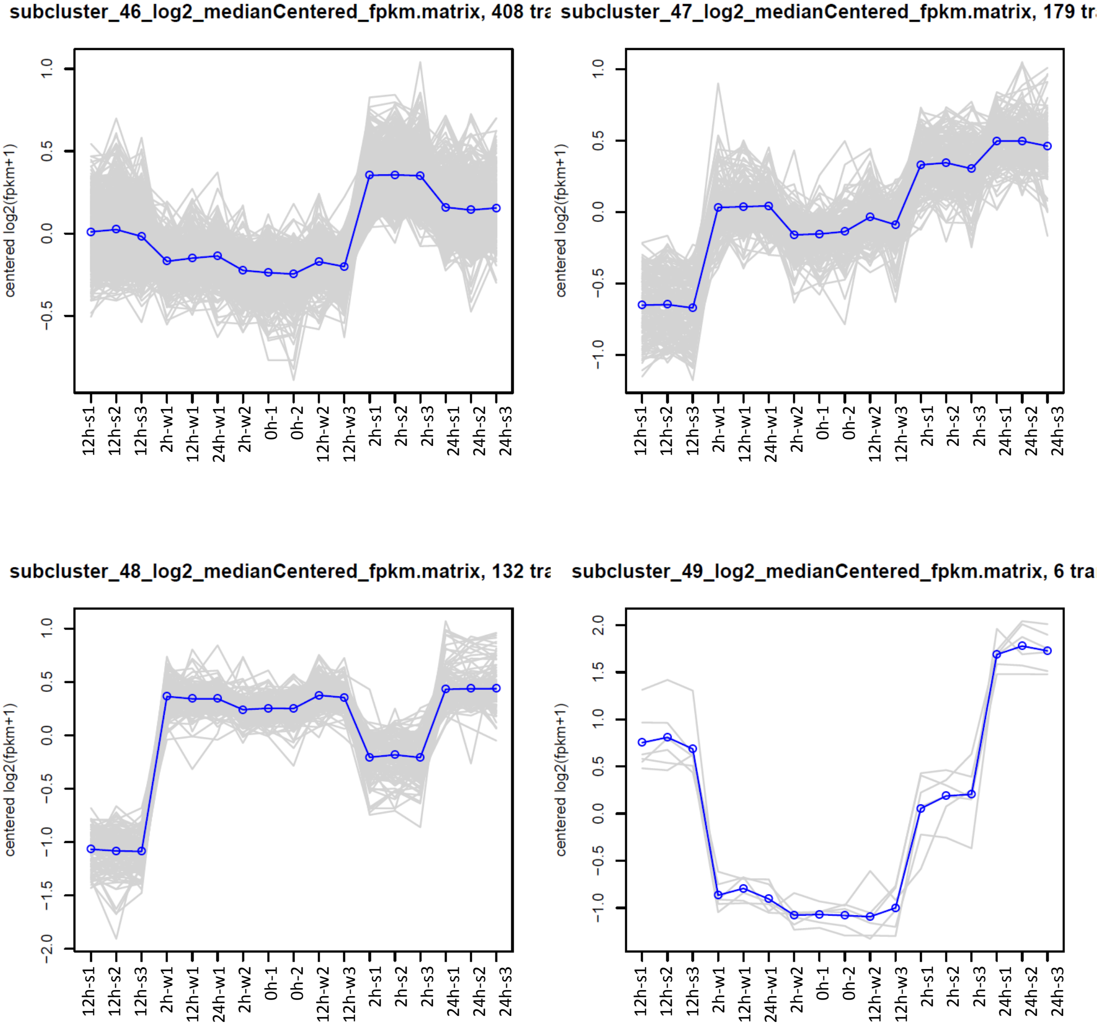

subcluster_46_log2_medianCentered_fpkm.matrix, 408 tra
subcluster_47_log2_medianCentered_fpkm.matrix, 179 tr
subcluster_48_log2_medianCentered_fpkm.matrix, 132 tra
subcluster_49_log2_medianCentered_fpkm.matrix, 6 tra
centered log2(fpkm+1)
12h-s1
12h-s2
12h-s3
2h-w1
12h-w1
24h-w1
2h-w2
0h-1
0h-2
12h-w2
12h-w3
2h-s1
2h-s2
2h-s3
24h-s1
24h-s2
24h-s3

Fig. S1.

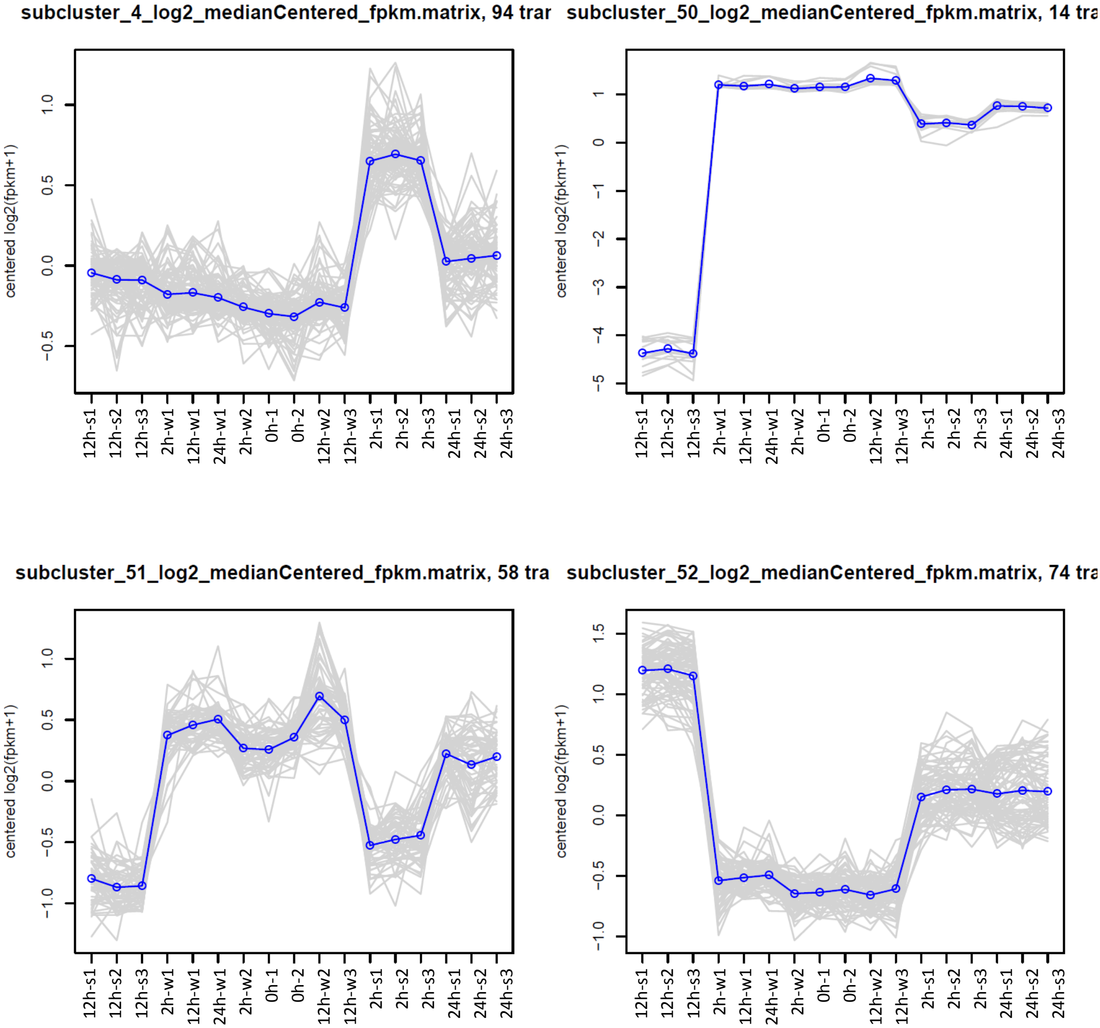

subcluster_4_log2_medianCentered_fpkm.matrix, 94 trar
subcluster_50_log2_medianCentered_fpkm.matrix, 14 tra
subcluster_51_log2_medianCentered_fpkm.matrix, 58 tra
subcluster_52_log2_medianCentered_fpkm.matrix, 74 tra
centered log2(fpkm+1)
12h-s1
12h-s2
12h-s3
2h-w1
12h-w1
24h-w1
2h-w2
0h-1
0h-2
12h-w2
12h-w3
2h-s1
2h-s2
2h-s3
24h-s1
24h-s2
24h-s3

Fig. S1.

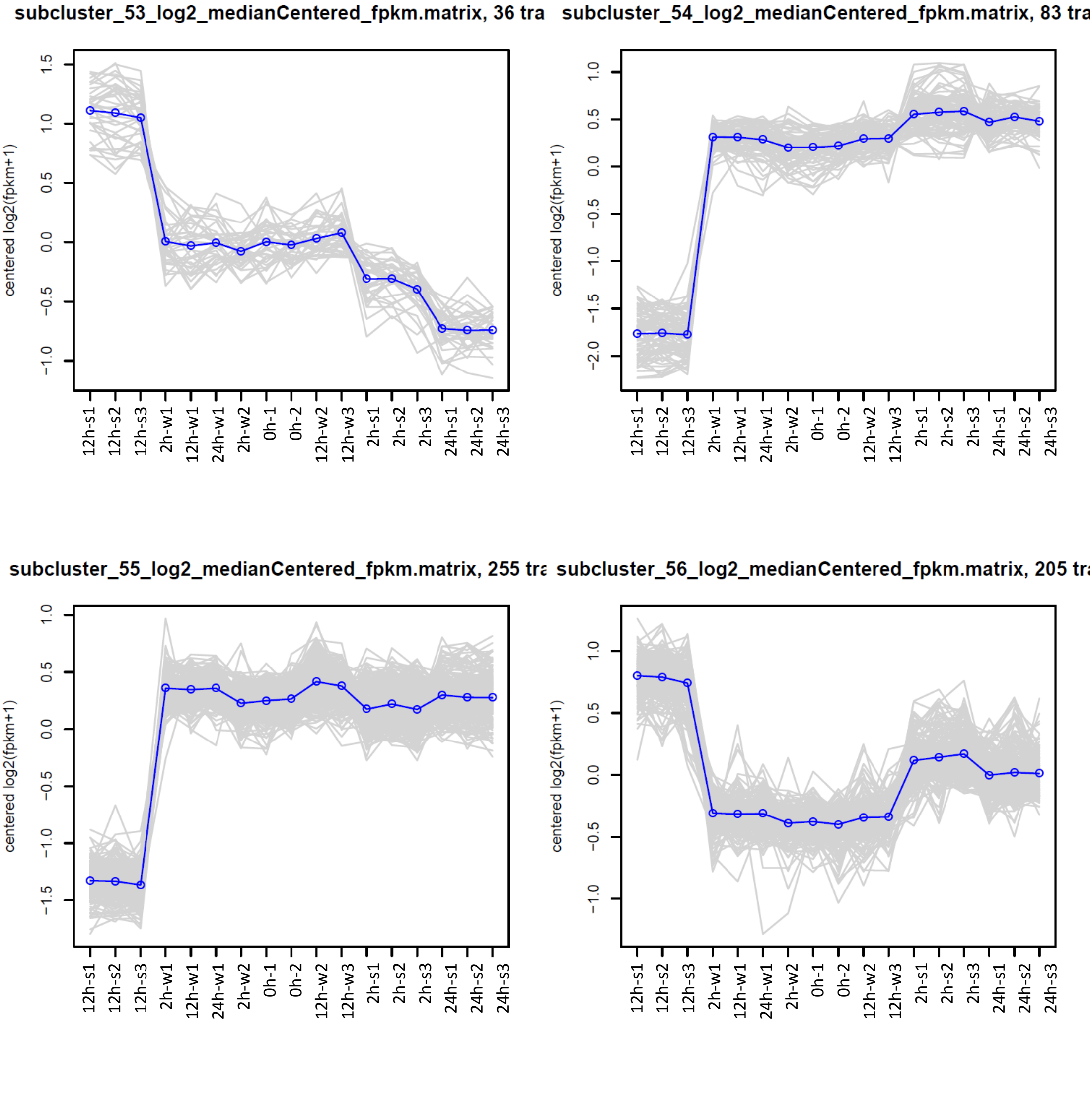
subcluster_53_log2_medianCentered_fpkm.matrix, 36 tra
subcluster_54_log2_medianCentered_fpkm.matrix, 83 tra
subcluster_55_log2_medianCentered_fpkm.matrix, 255 tra
subcluster_56_log2_medianCentered_fpkm.matrix, 205 tra
centered log2(fpkm+1)
12h-s1
12h-s2
12h-s3
2h-w1
12h-w1
24h-w1
2h-w2
0h-1
0h-2
12h-w2
12h-w3
2h-s1
2h-s2
2h-s3
24h-s1
24h-s2
24h-s3

Fig. S1.

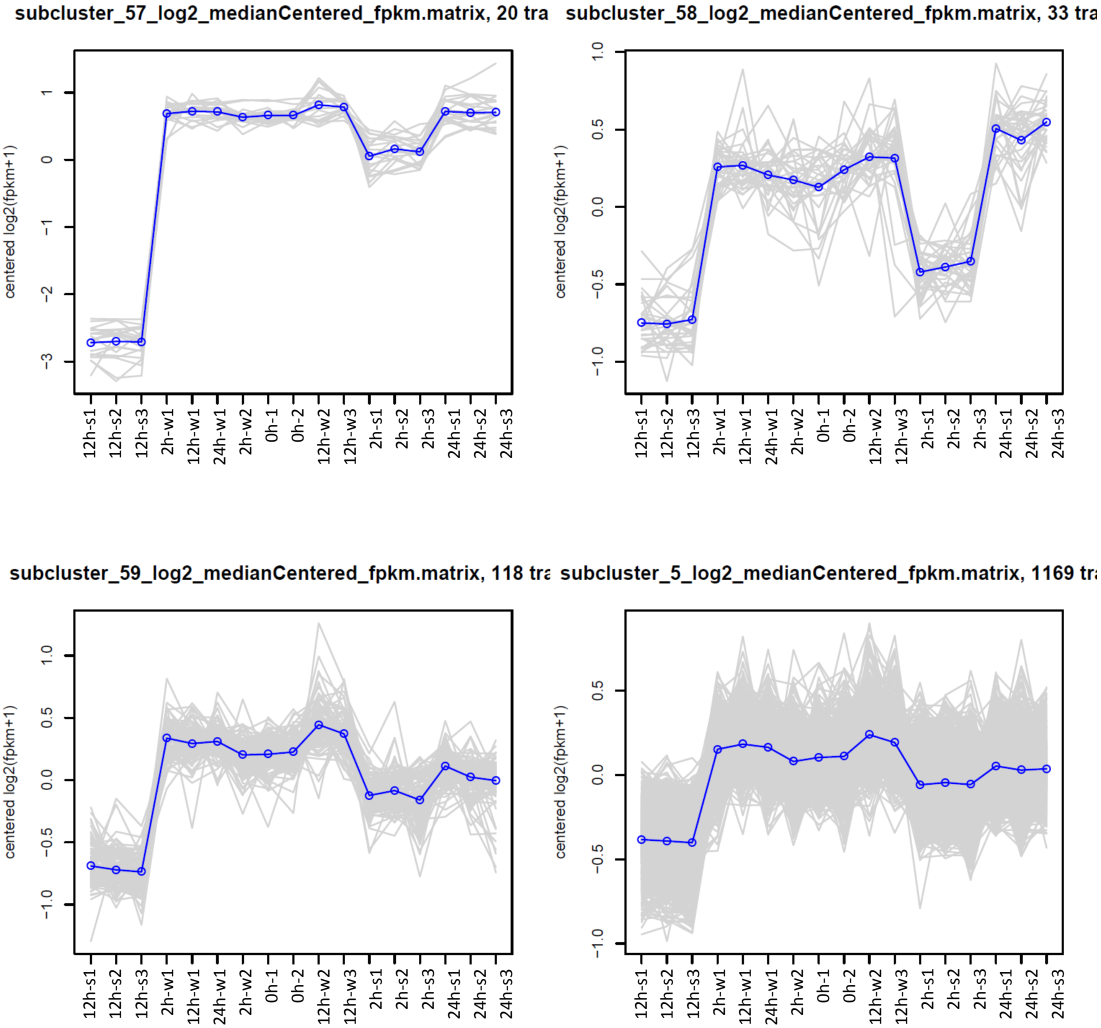
subcluster_57_log2_medianCentered_fpkm.matrix, 20 tra
subcluster_58_log2_medianCentered_fpkm.matrix, 33 tra
subcluster_59_log2_medianCentered_fpkm.matrix, 118 tra
subcluster_5_log2_medianCentered_fpkm.matrix, 1169 tr
centered log2(fpkm+1)
12h-s1
12h-s2
12h-s3
2h-w1
12h-w1
24h-w1
2h-w2
0h-1
0h-2
12h-w2
12h-w3
2h-s1
2h-s2
2h-s3
24h-s1
24h-s2
24h-s3

Fig. S1.

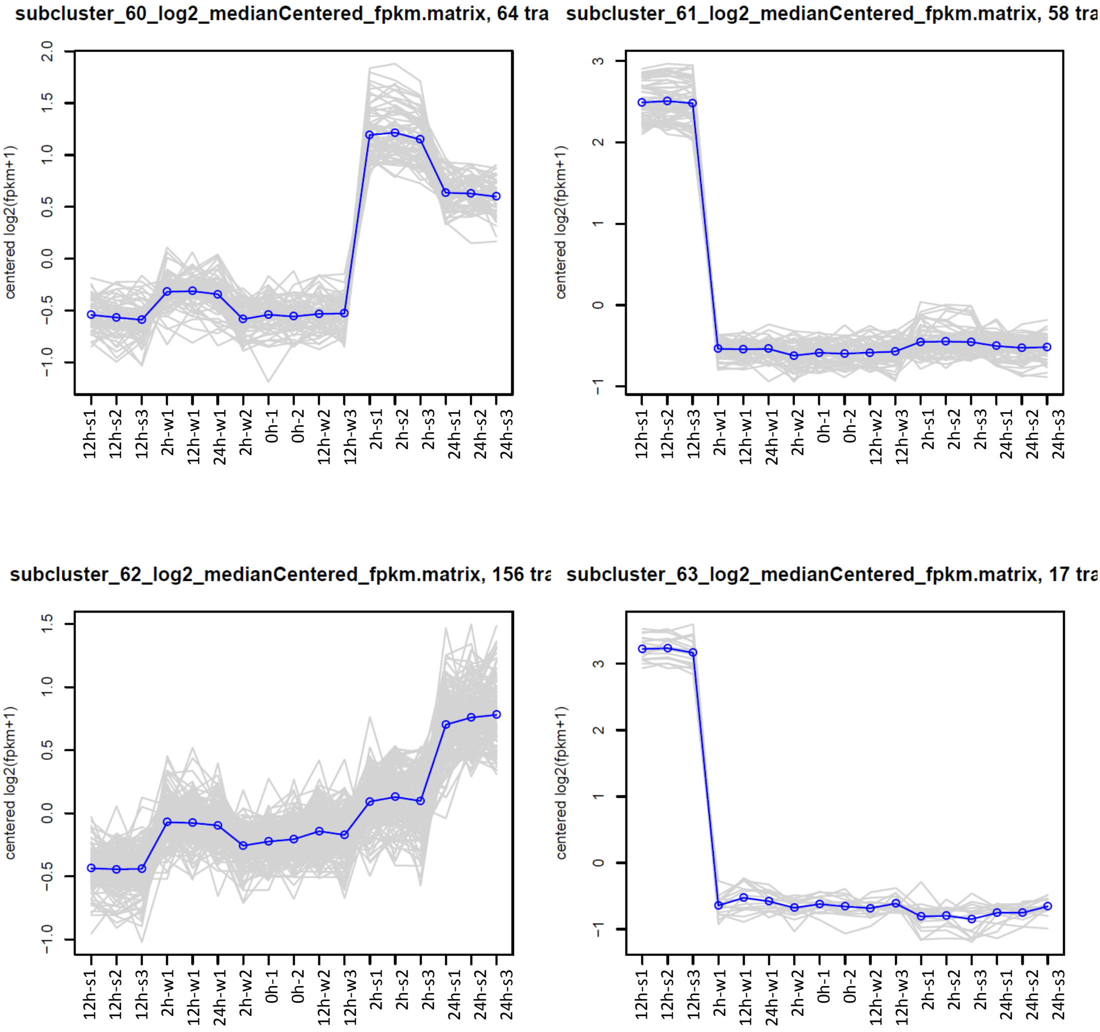

subcluster_60_log2_medianCentered_fpkm.matrix, 64 tra
subcluster_61_log2_medianCentered_fpkm.matrix, 58 tra
subcluster_62_log2_medianCentered_fpkm.matrix, 156 tra
subcluster_63_log2_medianCentered_fpkm.matrix, 17 tra
centered log2(fpkm+1)
12h-s1
12h-s2
12h-s3
2h-w1
12h-w1
24h-w1
2h-w2
0h-1
0h-2
12h-w2
12h-w3
2h-s1
2h-s2
2h-s3
24h-s1
24h-s2
24h-s3

Fig. S1.

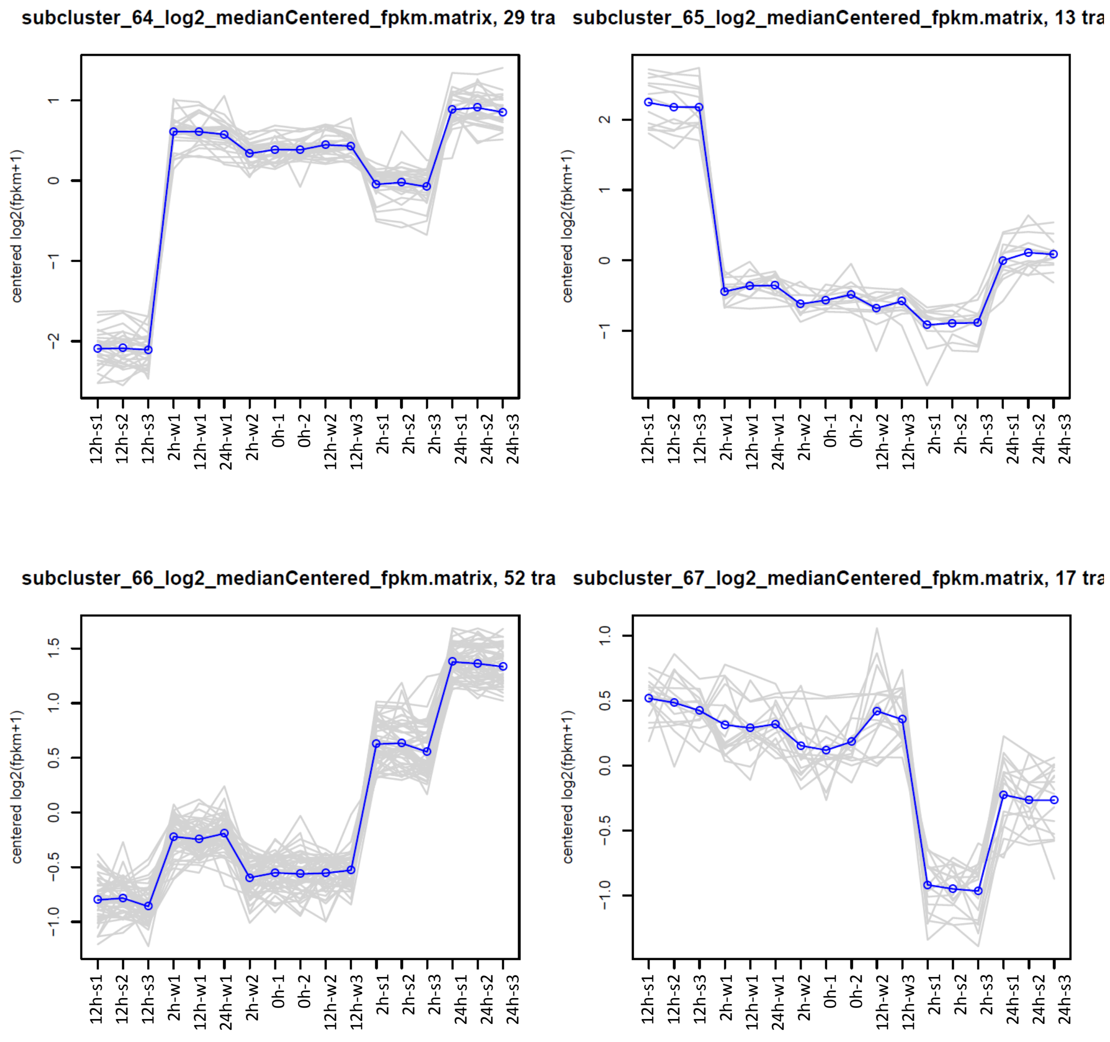
subcluster_64_log2_medianCentered_fpkm.matrix, 29 tra
subcluster_65_log2_medianCentered_fpkm.matrix, 13 tra
subcluster_66_log2_medianCentered_fpkm.matrix, 52 tra
subcluster_67_log2_medianCentered_fpkm.matrix, 17 tra
centered log2(fpkm+1)
12h-s1
12h-s2
12h-s3
2h-w1
12h-w1
24h-w1
2h-w2
0h-1
0h-2
12h-w2
12h-w3
2h-s1
2h-s2
2h-s3
24h-s1
24h-s2
24h-s3

Fig. S1.

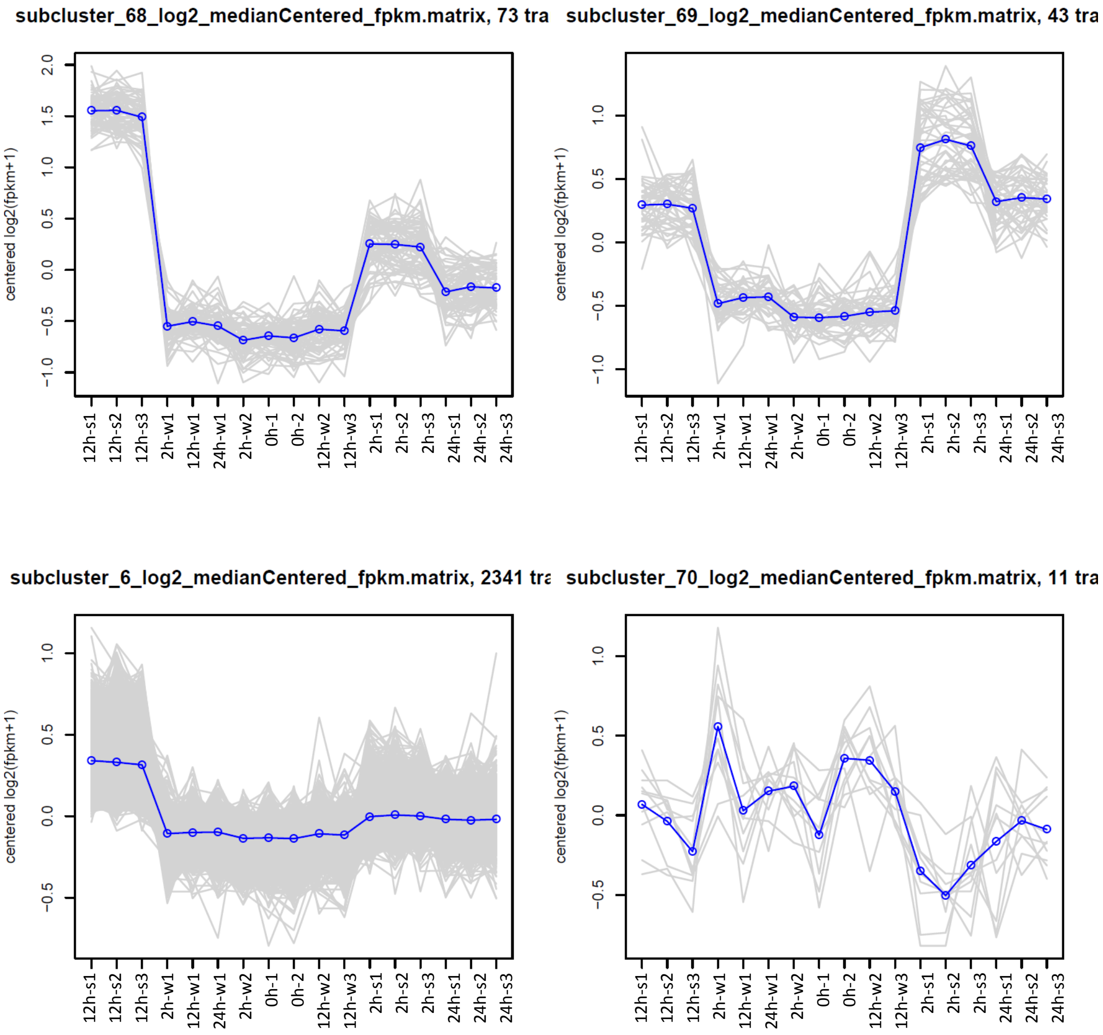

subcluster_68_log2_medianCentered_fpkm.matrix, 73 tra
subcluster_69_log2_medianCentered_fpkm.matrix, 43 tra
subcluster_6_log2_medianCentered_fpkm.matrix, 2341 tra
subcluster_70_log2_medianCentered_fpkm.matrix, 11 tra
centered log2(fpkm+1)
12h-s1
12h-s2
12h-s3
2h-w1
12h-w1
24h-w1
2h-w2
0h-1
0h-2
12h-w2
12h-w3
2h-s1
2h-s2
2h-s3
24h-s1
24h-s2
24h-s3

Fig. S1.

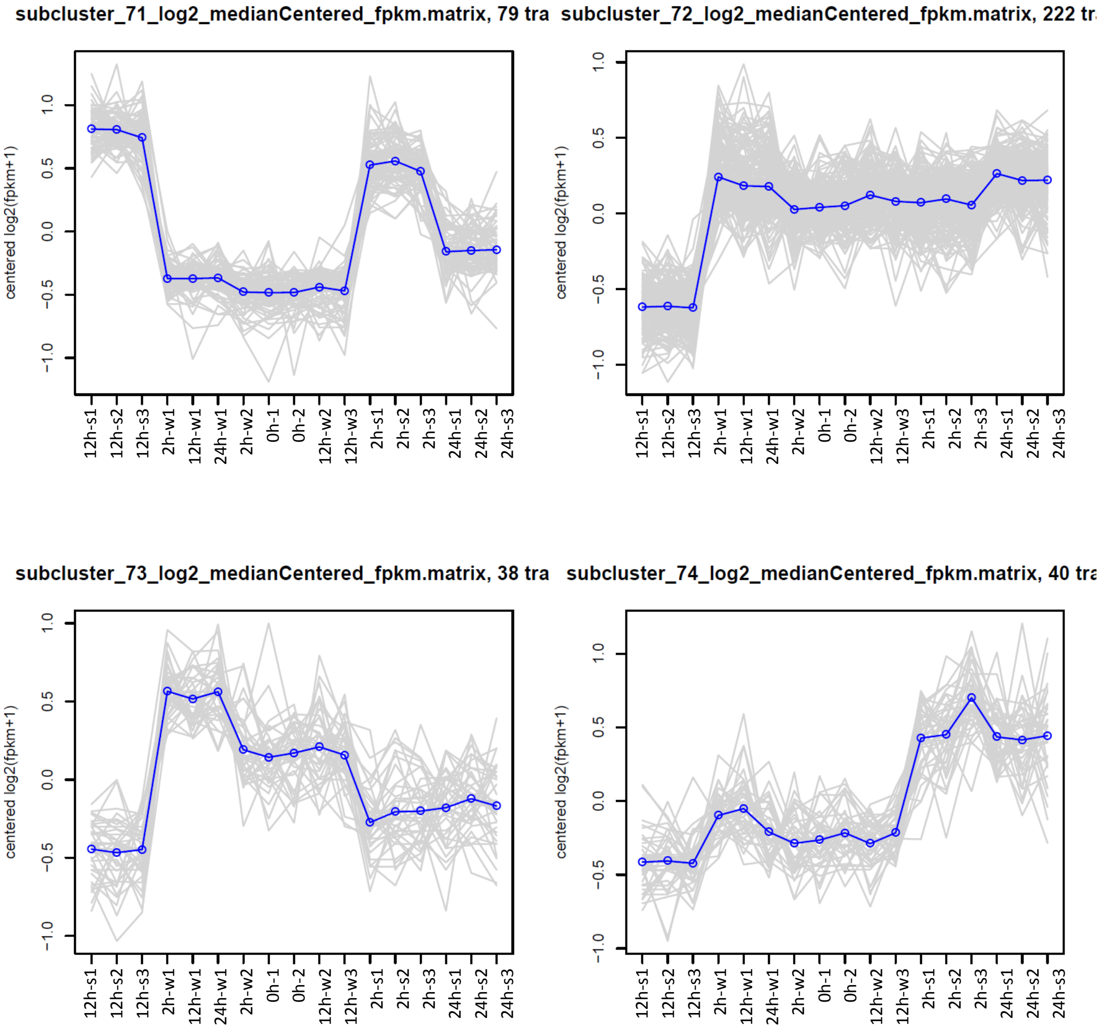
subcluster_71_log2_medianCentered_fpkm.matrix, 79 tra
subcluster_72_log2_medianCentered_fpkm.matrix, 222 tr
subcluster_73_log2_medianCentered_fpkm.matrix, 38 tra
subcluster_74_log2_medianCentered_fpkm.matrix, 40 tra
centered log2(fpkm+1)
−1.0
−0.5
0.0
0.5
1.0
12h-s1
12h-s2
12h-s3
2h-w1
12h-w1
24h-w1
2h-w2
0h-1
0h-2
12h-w2
12h-w3
2h-s1
2h-s2
2h-s3
24h-s1
24h-s2
24h-s3

Fig. S1.

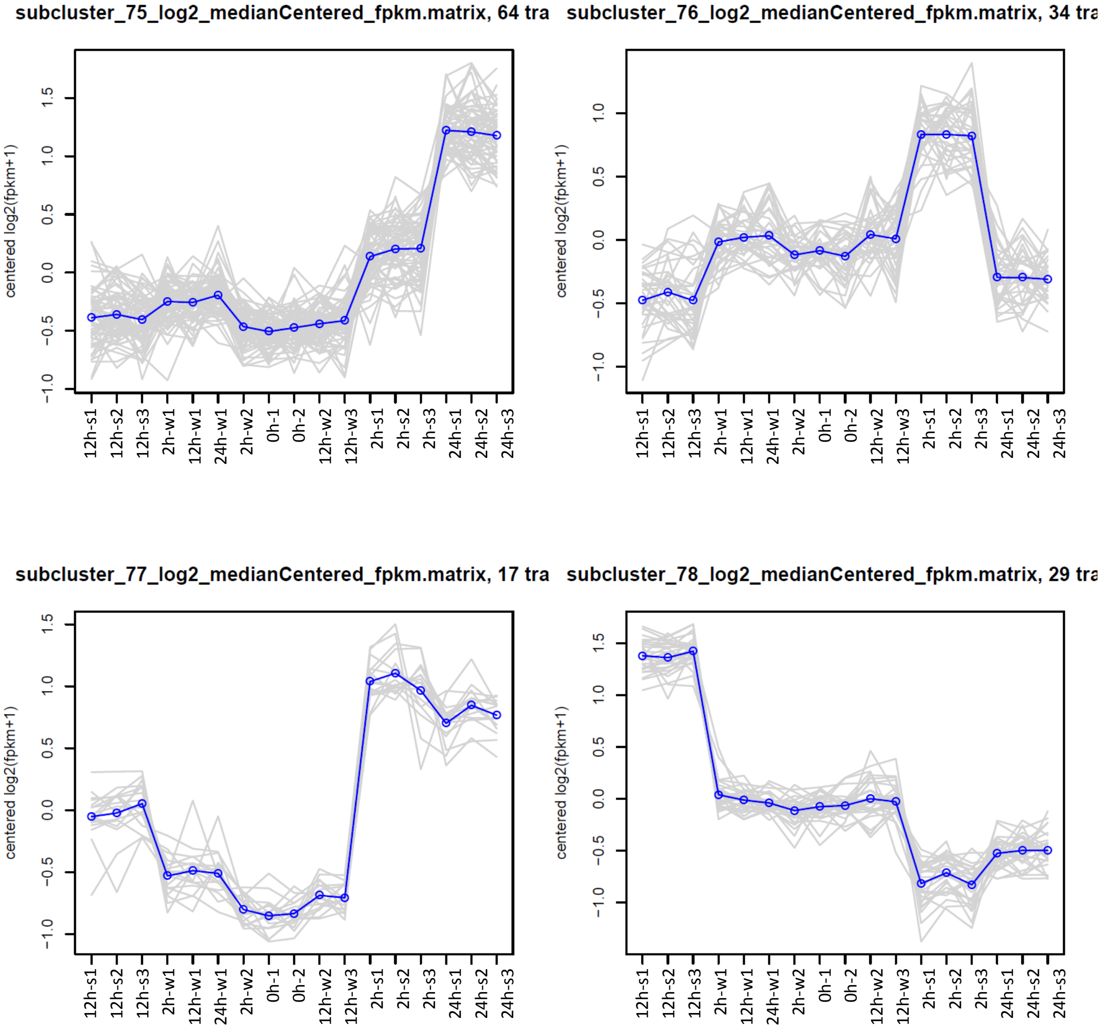
subcluster_75_log2_medianCentered_fpkm.matrix, 64 tra
subcluster_76_log2_medianCentered_fpkm.matrix, 34 tra
subcluster_77_log2_medianCentered_fpkm.matrix, 17 tra
subcluster_78_log2_medianCentered_fpkm.matrix, 29 tra
centered log2(fpkm+1)
12h-s1
12h-s2
12h-s3
2h-w1
12h-w1
24h-w1
2h-w2
0h-1
0h-2
12h-w2
12h-w3
2h-s1
2h-s2
2h-s3
24h-s1
24h-s2
24h-s3

Fig. S1.

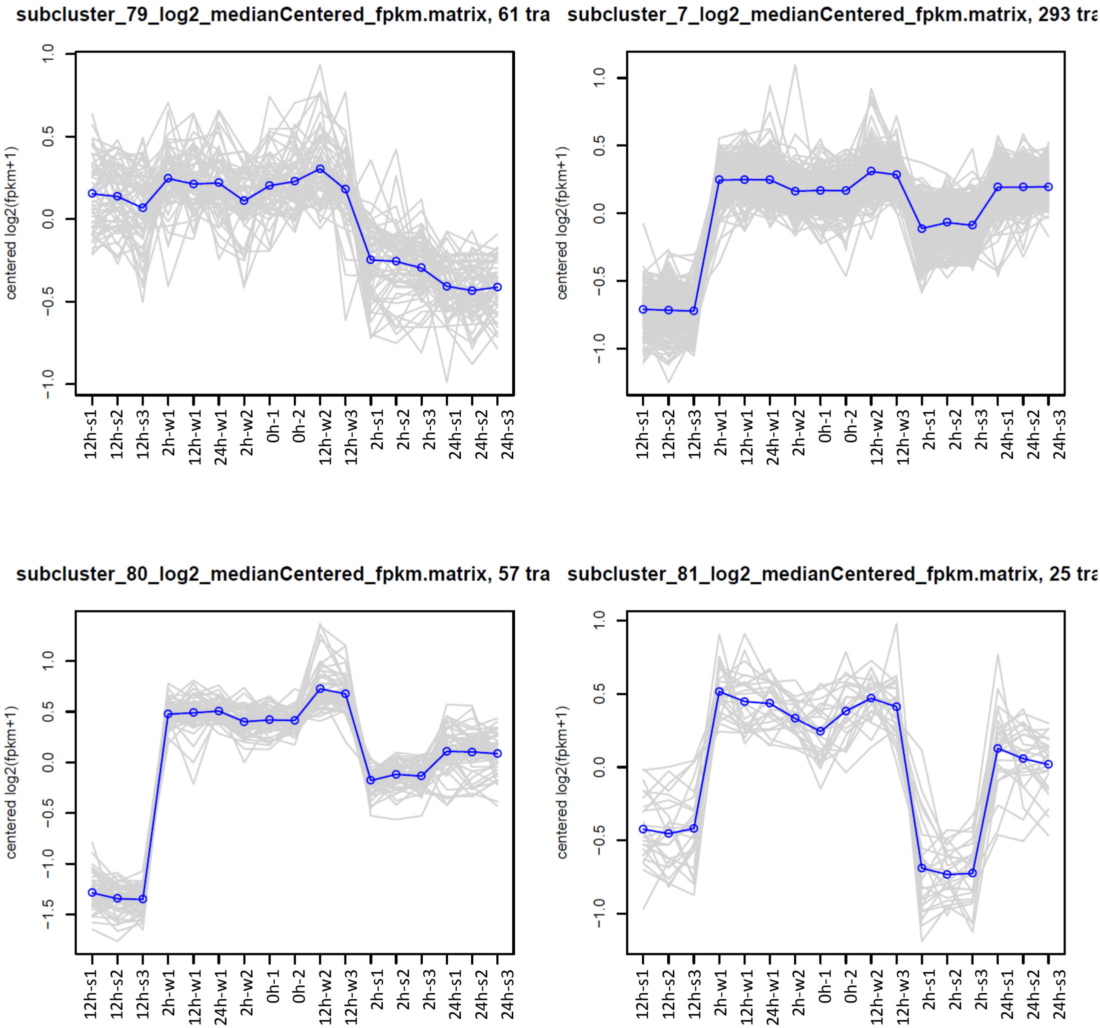
subcluster_79_log2_medianCentered_fpkm.matrix, 61 tra
subcluster_7_log2_medianCentered_fpkm.matrix, 293 tra
subcluster_80_log2_medianCentered_fpkm.matrix, 57 tra
subcluster_81_log2_medianCentered_fpkm.matrix, 25 tra
centered log2(fpkm+1)
12h-s1
12h-s2
12h-s3
2h-w1
12h-w1
24h-w1
2h-w2
0h-1
0h-2
12h-w2
12h-w3
2h-s1
2h-s2
2h-s3
24h-s1
24h-s2
24h-s3

Fig. S1.

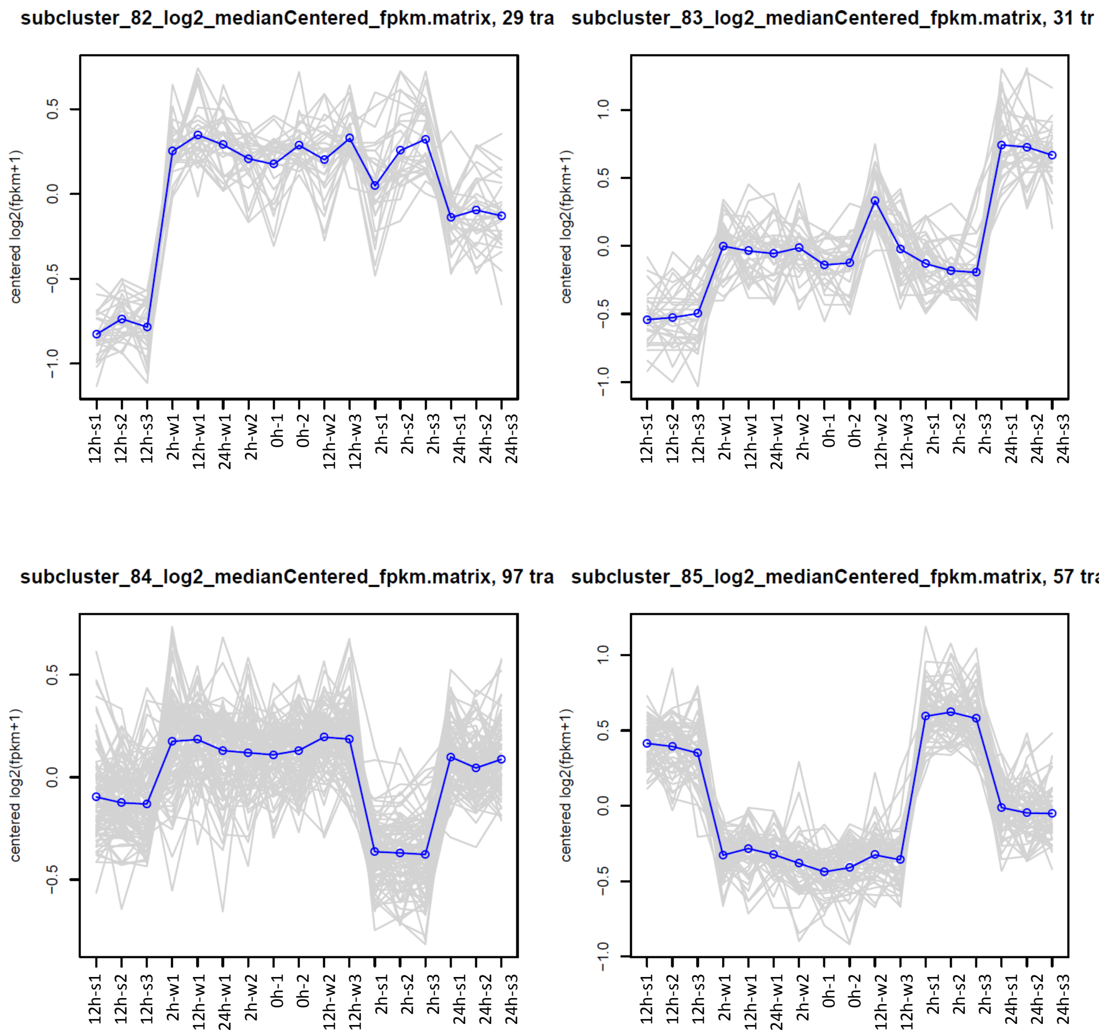
subcluster_82_log2_medianCentered_fpkm.matrix, 29 tra
subcluster_83_log2_medianCentered_fpkm.matrix, 31 tr
subcluster_84_log2_medianCentered_fpkm.matrix, 97 tra
subcluster_85_log2_medianCentered_fpkm.matrix, 57 tra
centered log2(fpkm+1)
-1.0
-0.5
0.0
0.5
1.0
12h-s1
12h-s2
12h-s3
2h-w1
12h-w1
24h-w1
2h-w2
0h-1
0h-2
12h-w2
12h-w3
2h-s1
2h-s2
2h-s3
24h-s1
24h-s2
24h-s3

Fig. S1.

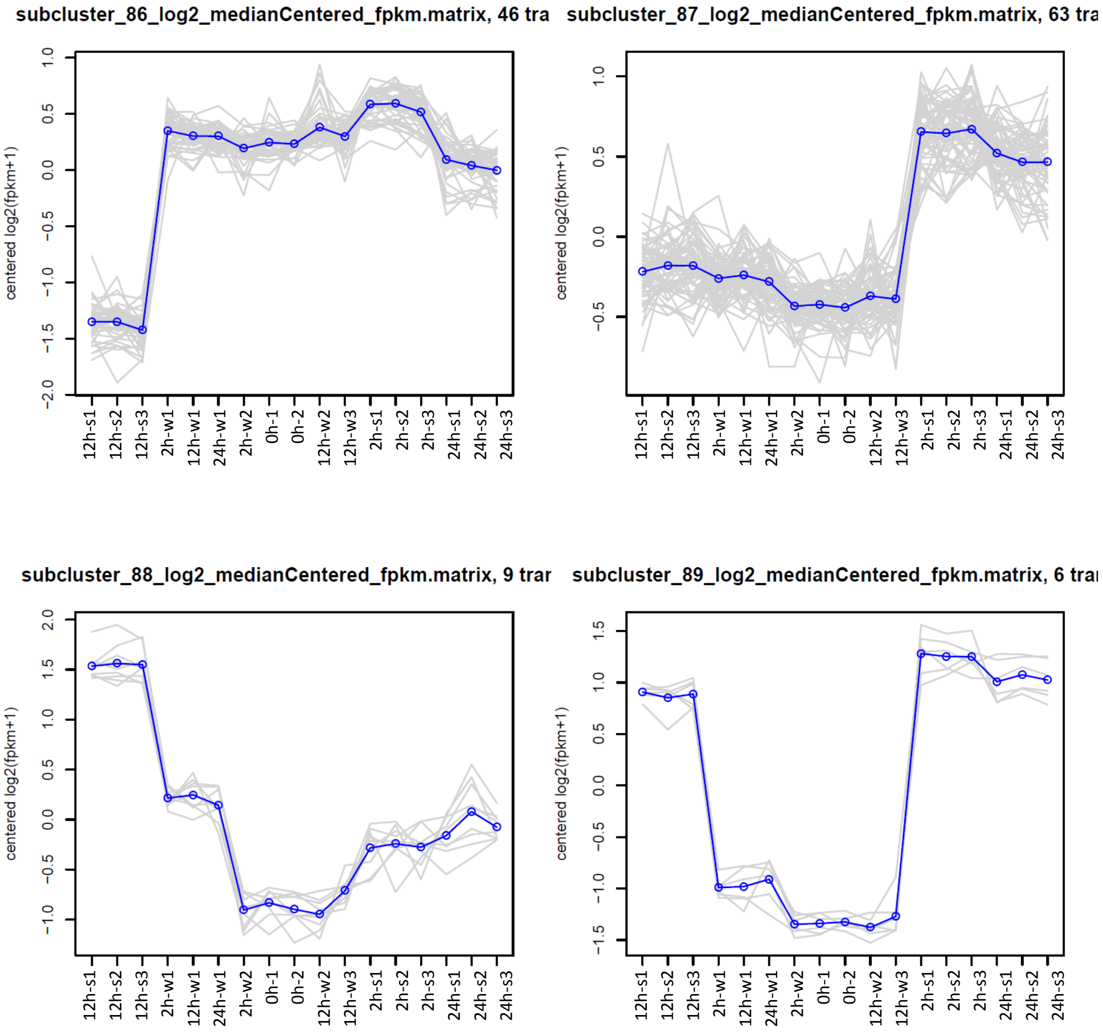
subcluster_86_log2_medianCentered_fpkm.matrix, 46 tra
subcluster_87_log2_medianCentered_fpkm.matrix, 63 tra
subcluster_88_log2_medianCentered_fpkm.matrix, 9 trar
subcluster_89_log2_medianCentered_fpkm.matrix, 6 trai
centered log2(fpkm+1)
12h-s1
12h-s2
12h-s3
2h-w1
12h-w1
24h-w1
2h-w2
0h-1
0h-2
12h-w2
12h-w3
2h-s1
2h-s2
2h-s3
24h-s1
24h-s2
24h-s3

Fig. S1.

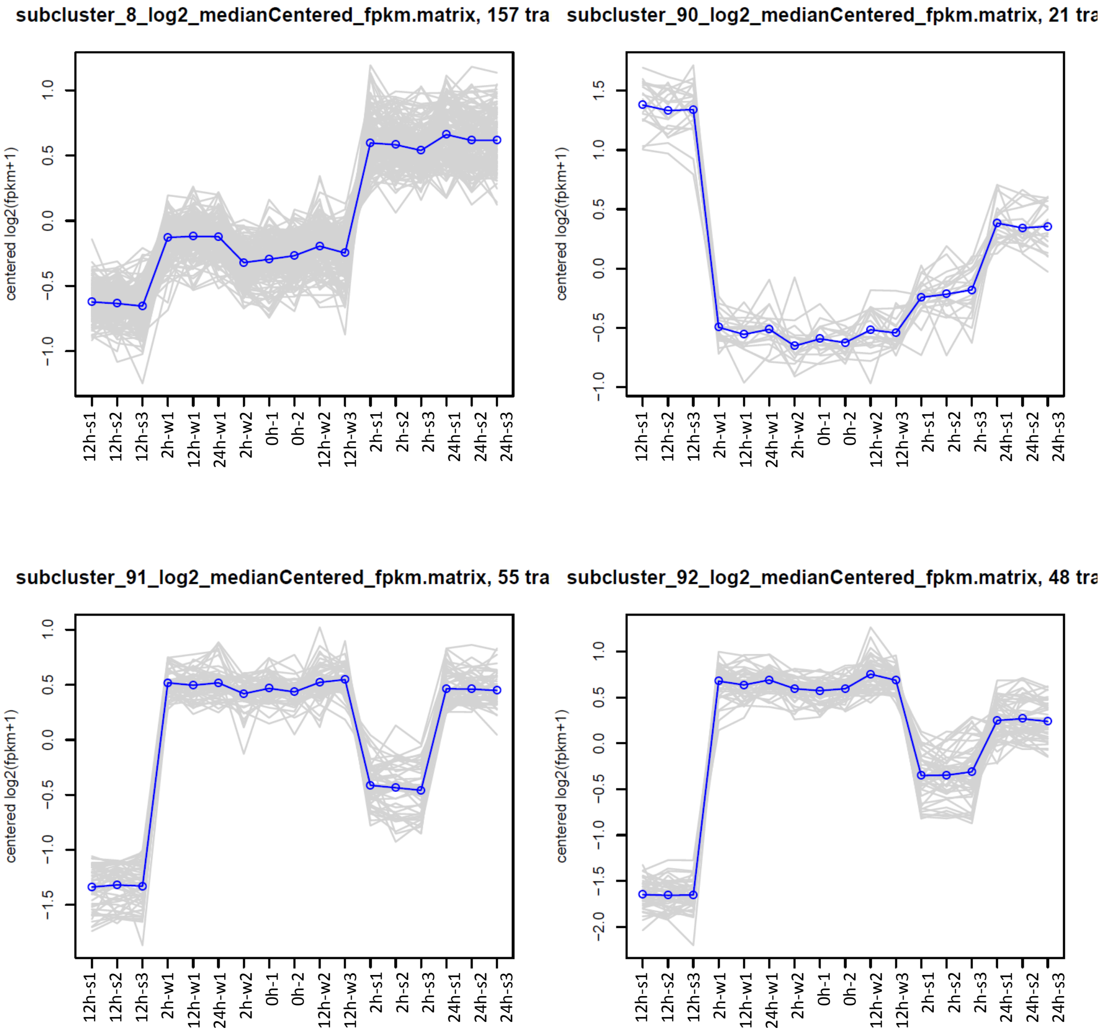
subcluster_8_log2_medianCentered_fpkm.matrix, 157 tra
centered log2(fpkm+1)
subcluster_90_log2_medianCentered_fpkm.matrix, 21 tra
subcluster_91_log2_medianCentered_fpkm.matrix, 55 tra
subcluster_92_log2_medianCentered_fpkm.matrix, 48 tra
12h-s1 12h-s2 12h-s3 2h-w1 12h-w1 24h-w1 2h-w2 0h-1 0h-2 12h-w2 12h-w3 2h-s1 2h-s2 2h-s3 24h-s1 24h-s2 24h-s3

Fig. S1.

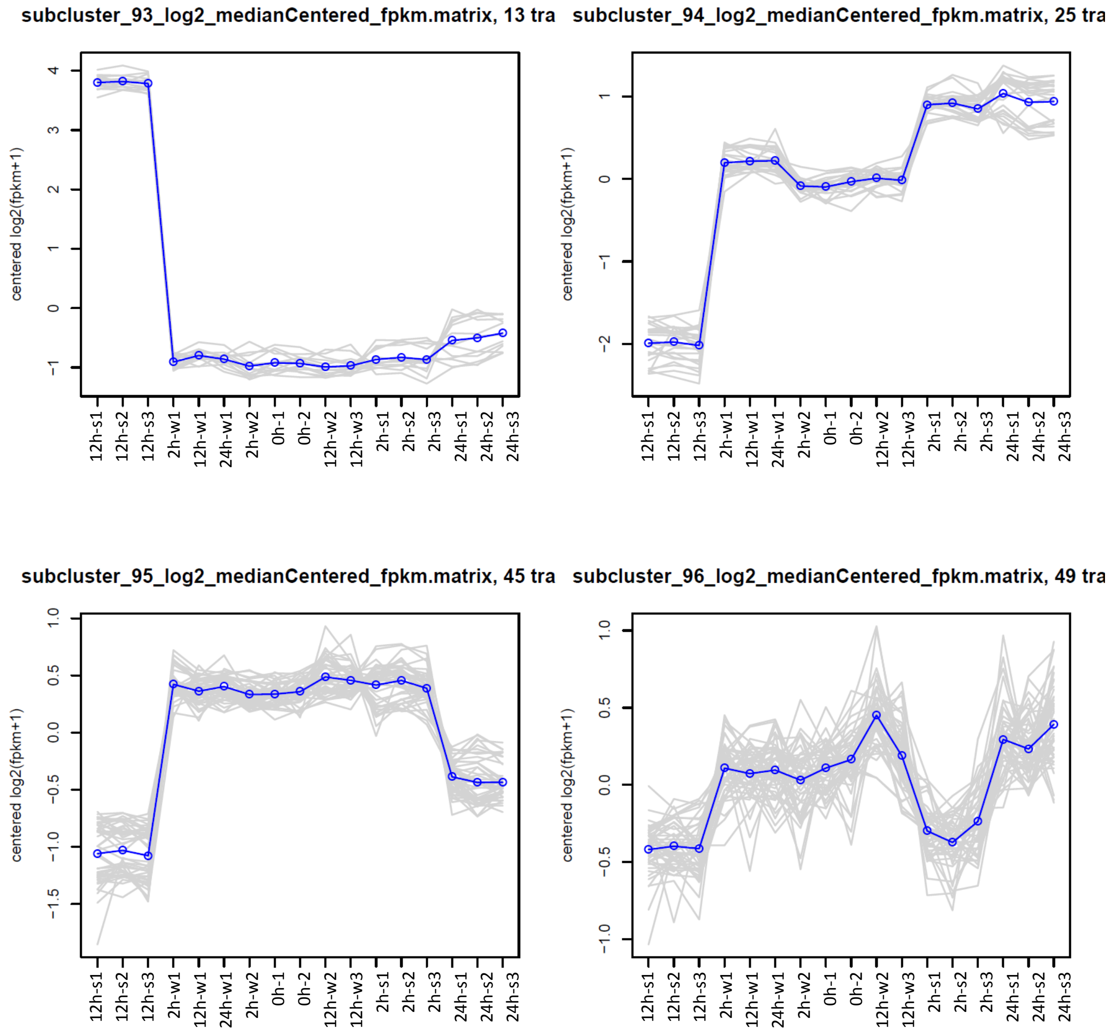
subcluster_93_log2_medianCentered_fpkm.matrix, 13 tra
subcluster_94_log2_medianCentered_fpkm.matrix, 25 tra
subcluster_95_log2_medianCentered_fpkm.matrix, 45 tra
subcluster_96_log2_medianCentered_fpkm.matrix, 49 tra
centered log2(fpkm+1)
12h-s1
12h-s2
12h-s3
2h-w1
12h-w1
24h-w1
2h-w2
0h-1
0h-2
12h-w2
12h-w3
2h-s1
2h-s2
2h-s3
24h-s1
24h-s2
24h-s3

Fig. S1.

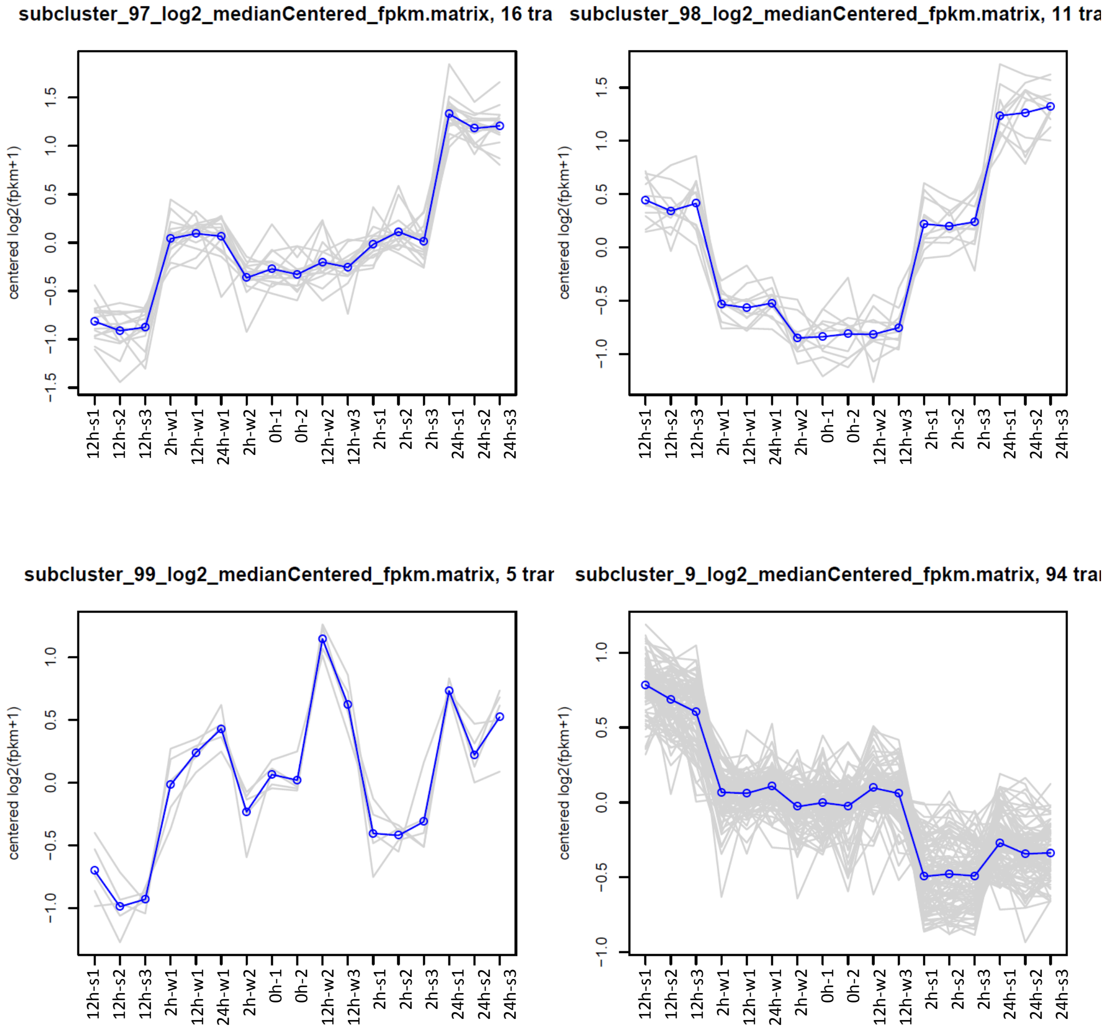
subcluster_97_log2_medianCentered_fpkm.matrix, 16 tra
centered log2(fpkm+1)
−1.5
−1.0
−0.5
0.0
0.5
1.0
1.5
12h-s1
12h-s2
12h-s3
2h-w1
12h-w1
24h-w1
2h-w2
0h-1
0h-2
12h-w2
12h-w3
2h-s1
2h-s2
2h-s3
24h-s1
24h-s2
24h-s3
subcluster_98_log2_medianCentered_fpkm.matrix, 11 tra
centered log2(fpkm+1)
−1.0
−0.5
0.0
0.5
1.0
1.5
12h-s1
12h-s2
12h-s3
2h-w1
12h-w1
24h-w1
2h-w2
0h-1
0h-2
12h-w2
12h-w3
2h-s1
2h-s2
2h-s3
24h-s1
24h-s2
24h-s3
subcluster_99_log2_medianCentered_fpkm.matrix, 5 trar
centered log2(fpkm+1)
−1.0
−0.5
0.0
0.5
1.0
12h-s1
12h-s2
12h-s3
2h-w1
12h-w1
24h-w1
2h-w2
0h-1
0h-2
12h-w2
12h-w3
2h-s1
2h-s2
2h-s3
24h-s1
24h-s2
24h-s3
subcluster_9_log2_medianCentered_fpkm.matrix, 94 trar
centered log2(fpkm+1)
−1.0
−0.5
0.0
0.5
1.0
12h-s1
12h-s2
12h-s3
2h-w1
12h-w1
24h-w1
2h-w2
0h-1
0h-2
12h-w2
12h-w3
2h-s1
2h-s2
2h-s3
24h-s1
24h-s2
24h-s3

Fig. S1.
