# Supplementary figures and images for: Transcriptomic analysis of salt stress responsive genes in Rhazya stricta
Source: PLoS One. 2017 May 16;12(5):e0177589. doi: 10.1371/journal.pone.0177589 (PMC5433744; doi:10.1371/journal.pone.0177589)

(a)

Gene

*At4g21065-1*

*At1g05670*

*At3g22470-1*

*At4g21065-2*

*At4g21065-3*

*At4g21065-4*

*At3g22470-2*

*At4g21065-5*

*At1g10910*

*At4g21065-6*


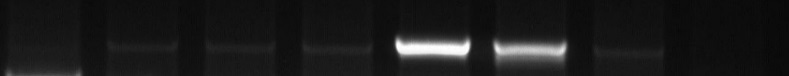

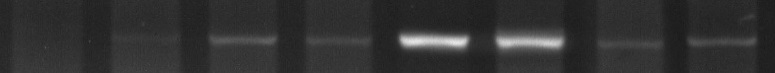

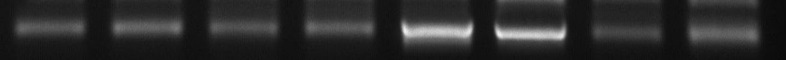

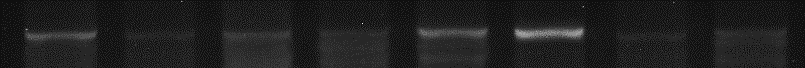

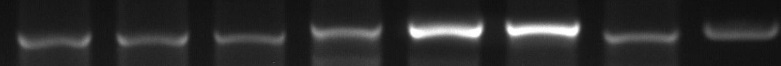

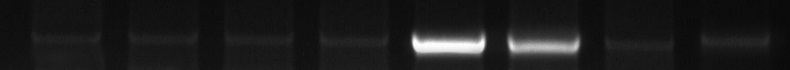

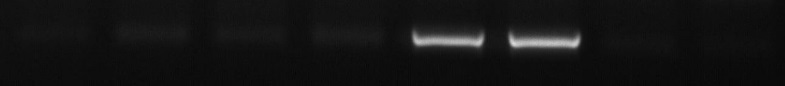

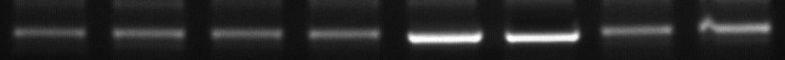

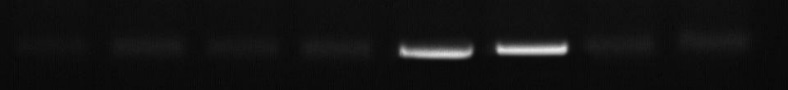

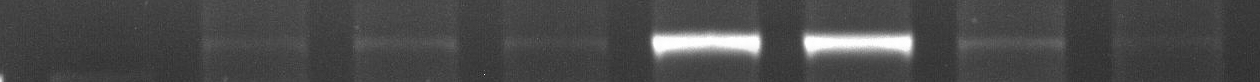


(b)


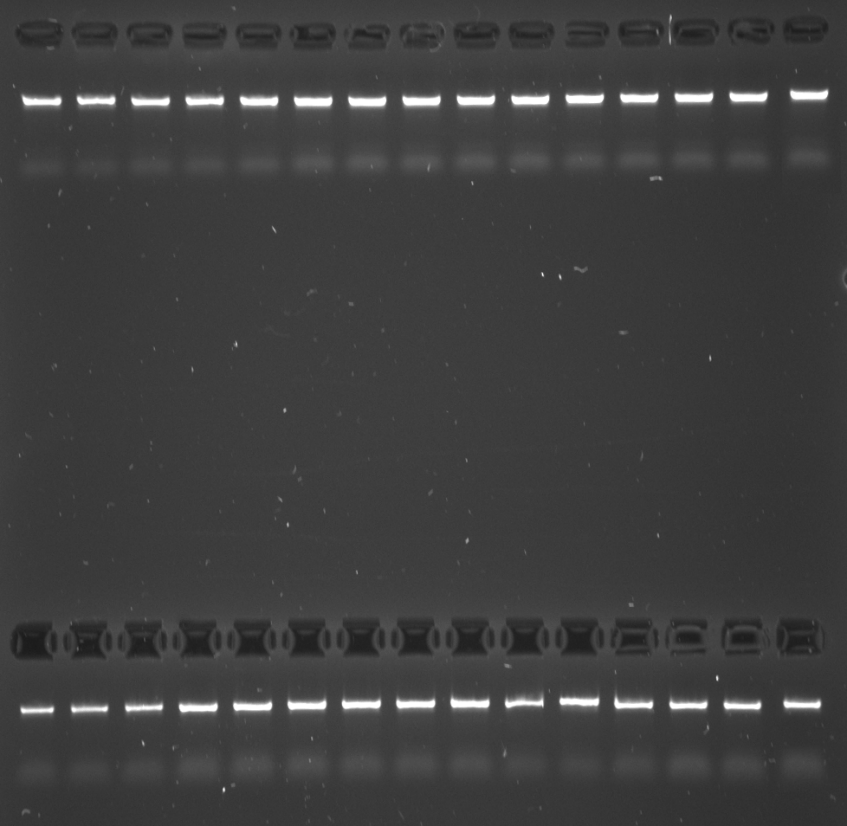


(c)

Figure S2.

Supplement: S2 Fig — (a) sqRT-PCR profiles of 10 upregulated PPR genes after 12 h of salt stress, (b) fold change profiles resulting from RNA-Seq analysis of the same PPR genes after 12 h of salt stress, and (c) the “actin” gene used as the unregulated house keeping gene. 0h1,2 (control), 2h-s1,2 (salt stressed for 2 h), 12h-s1,2 (salt stressed for 12 h), 24h-s1,2 (salt stressed for 24 h). Further information about the different genes is shown in S1 Table. (DOCX) [file pone.0177589.s002.docx]

Figure S3.


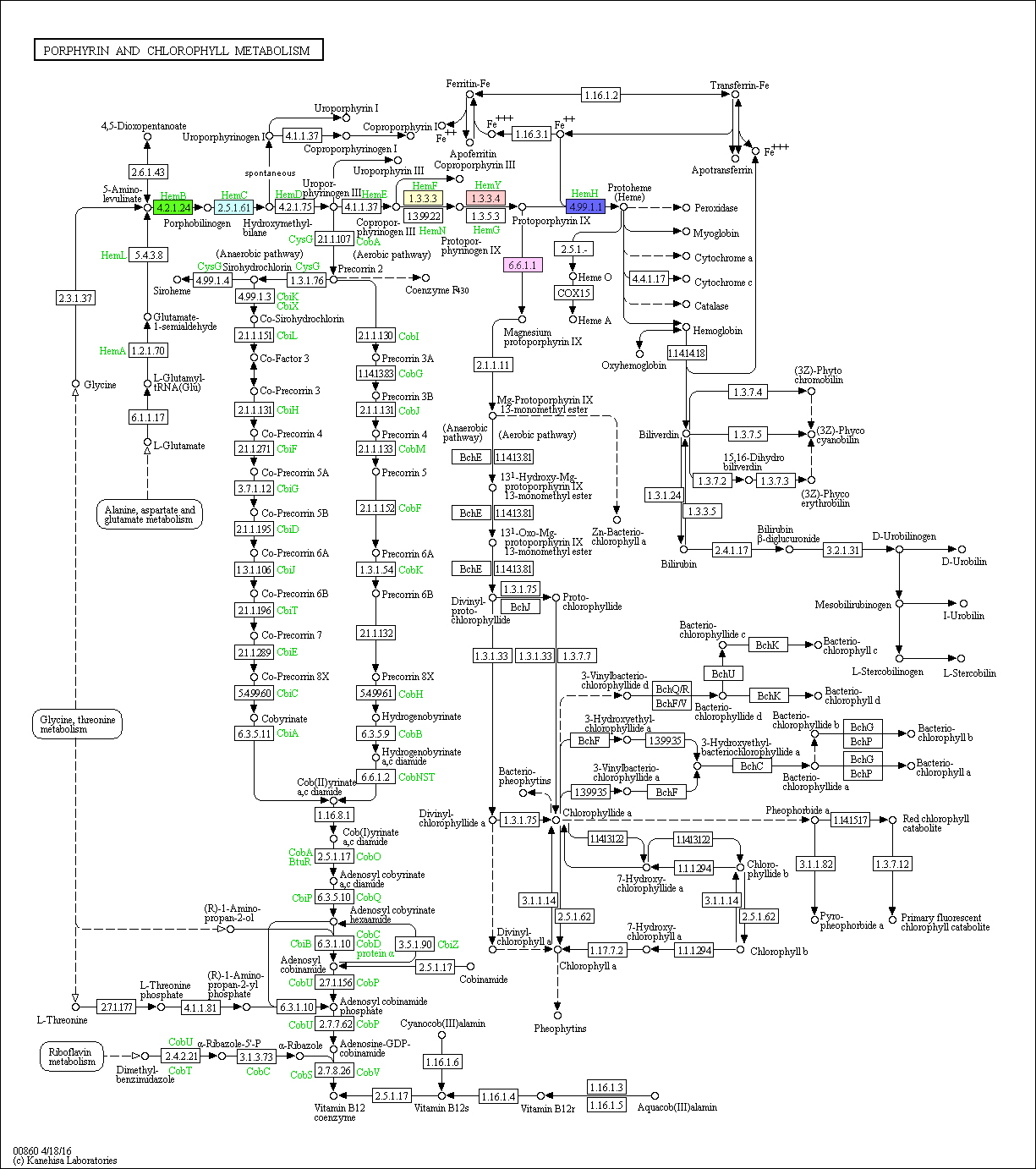

Supplement: S3 Fig — Colored rectangular boxes indicate the activated enzymes in the pathway. Highly activated enzymes at 12 h time point of salt (500 mM NaCl) stress are shown in colored boxes, while the enzymes with unchanged activation rates are shown in uncolored boxes. Different box colors in the pathway indicates different highly activated enzymes. Non-colored boxes indicate no change in enzyme activity at 12 h time point compared to 0 h time point. Red arrows indicate four enzymes that are expected to result in a higher rate under salt stress due to the high activation rates; orange arrow indicates the enzyme Fe-chelatase; green arrow indicates the enzyame Mg-chelatase. (DOCX) [file pone.0177589.s003.docx]

Figure S6.


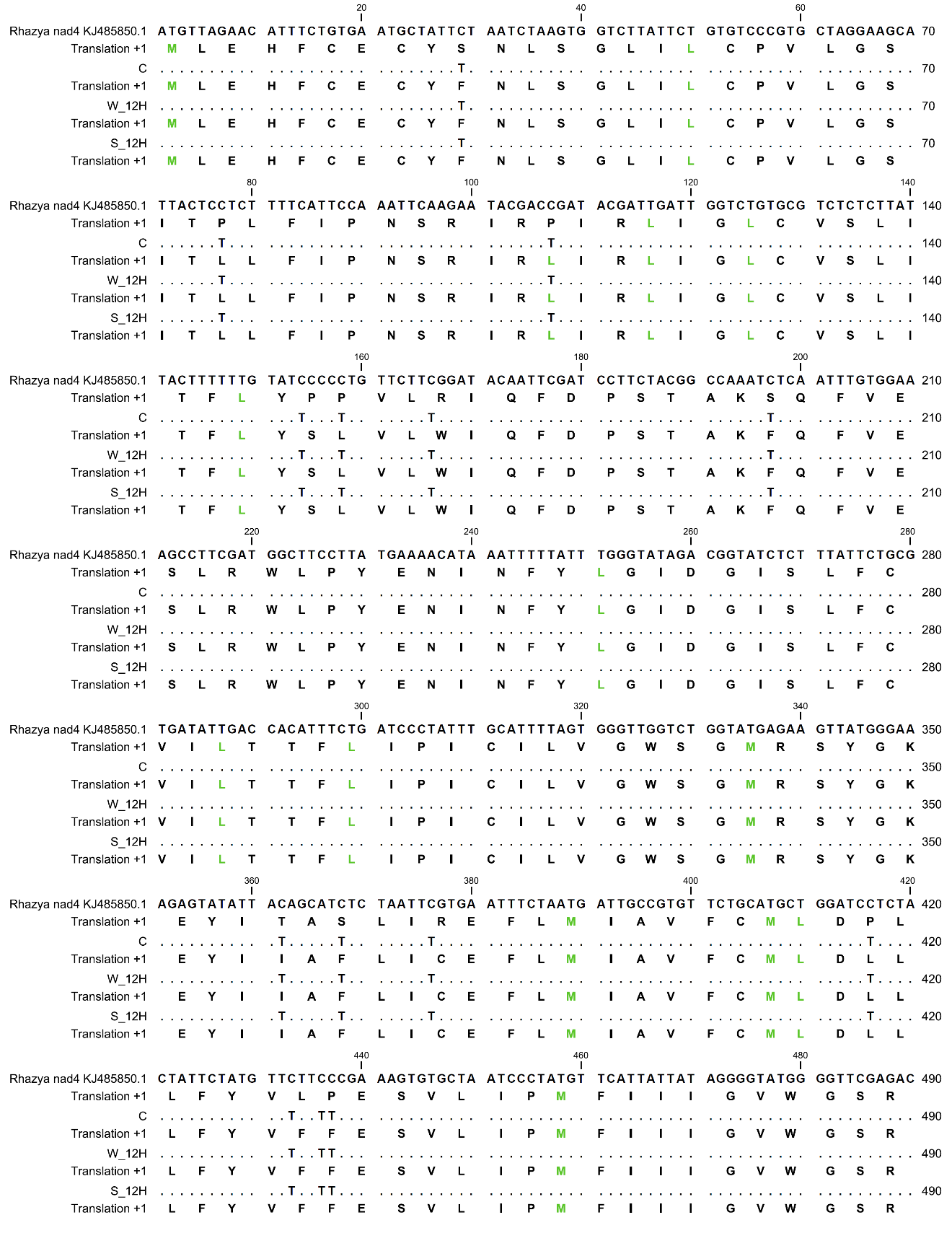

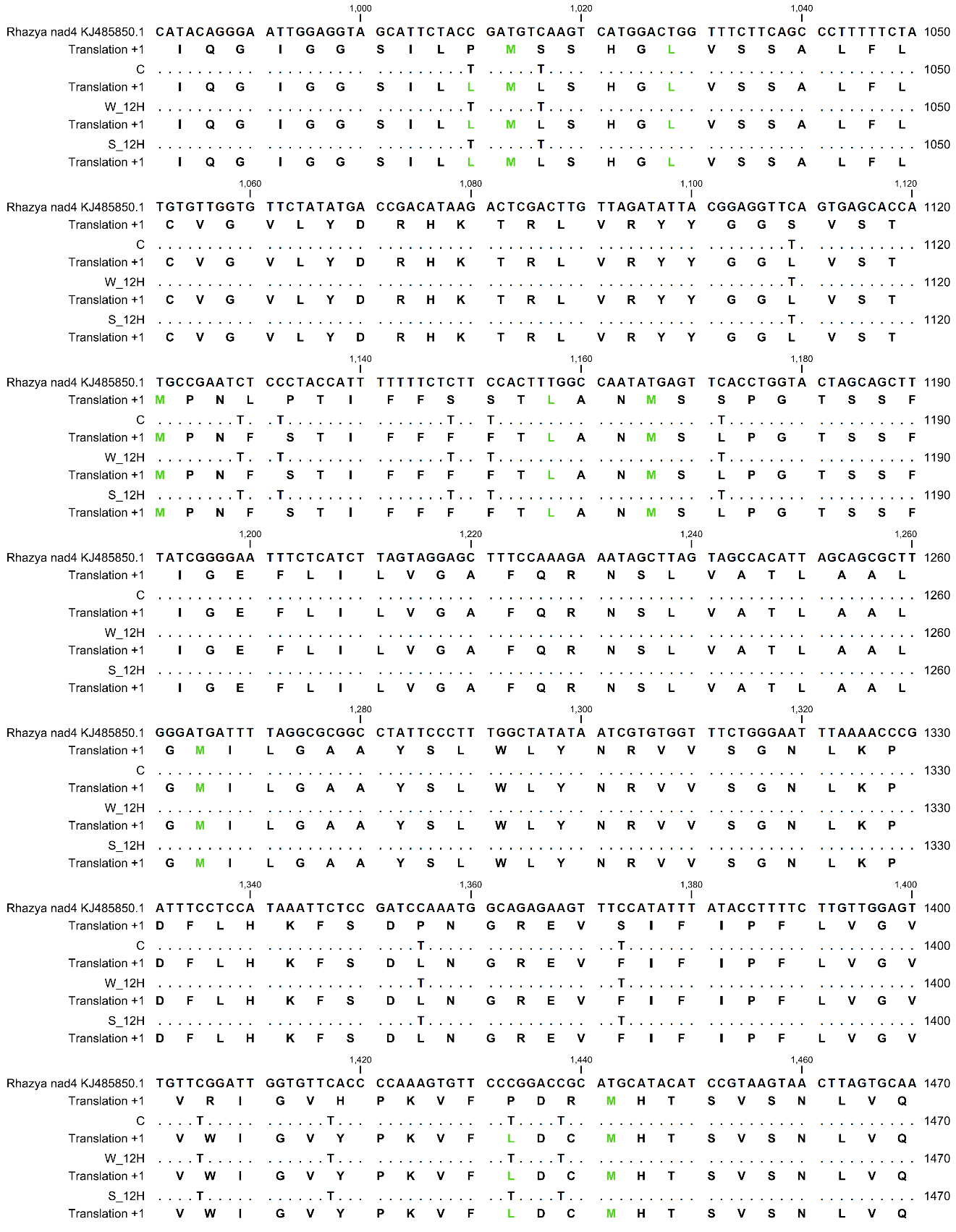

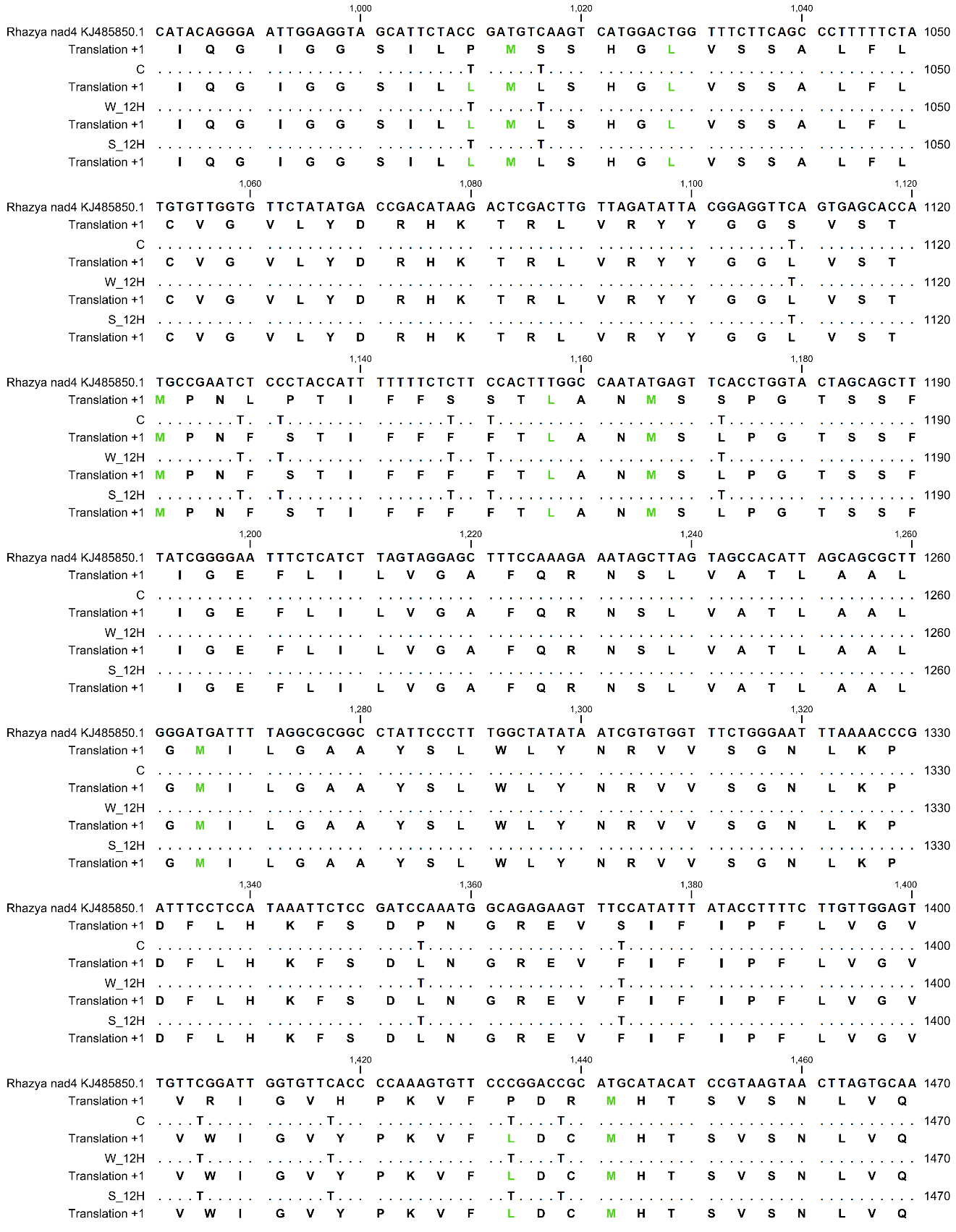

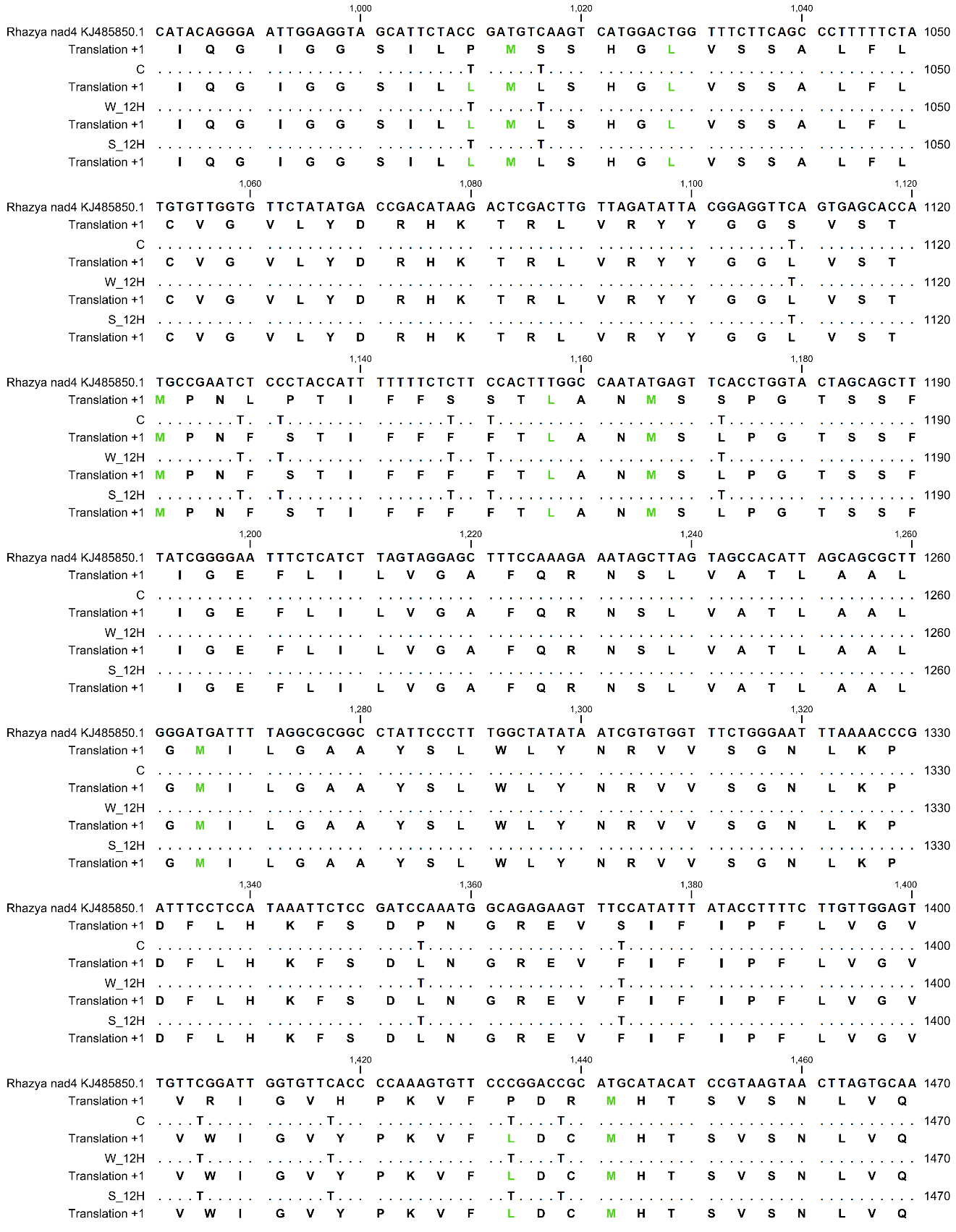

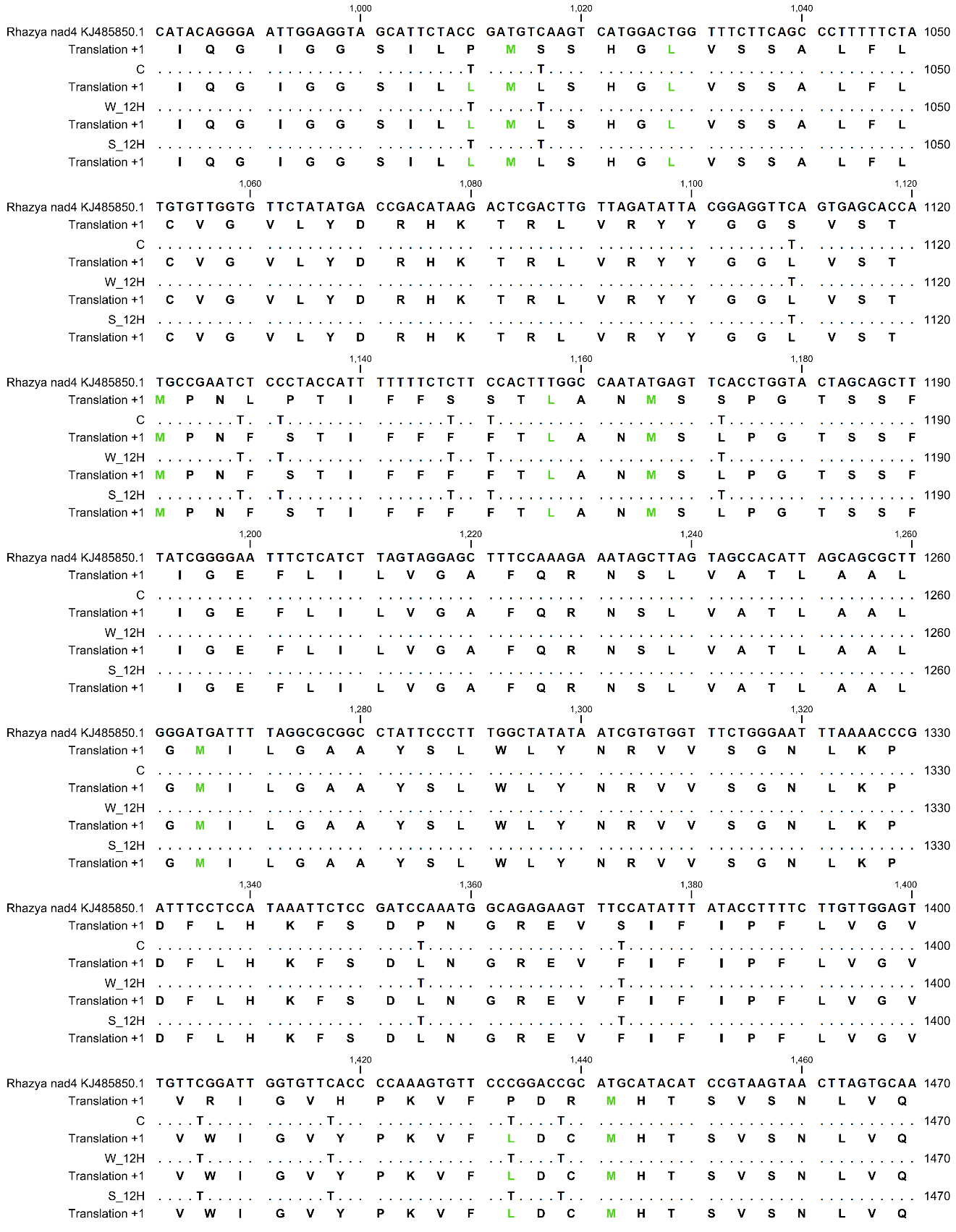

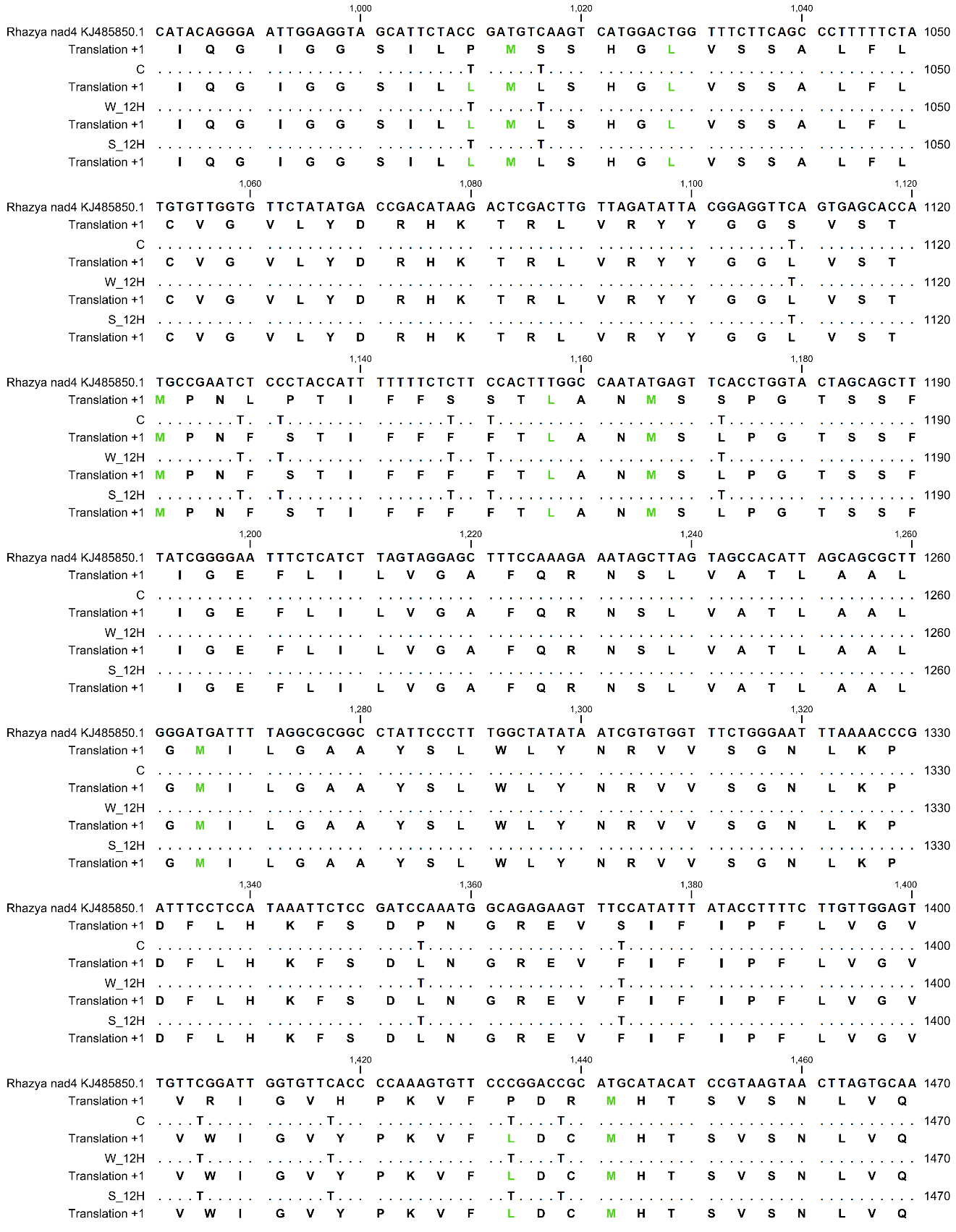

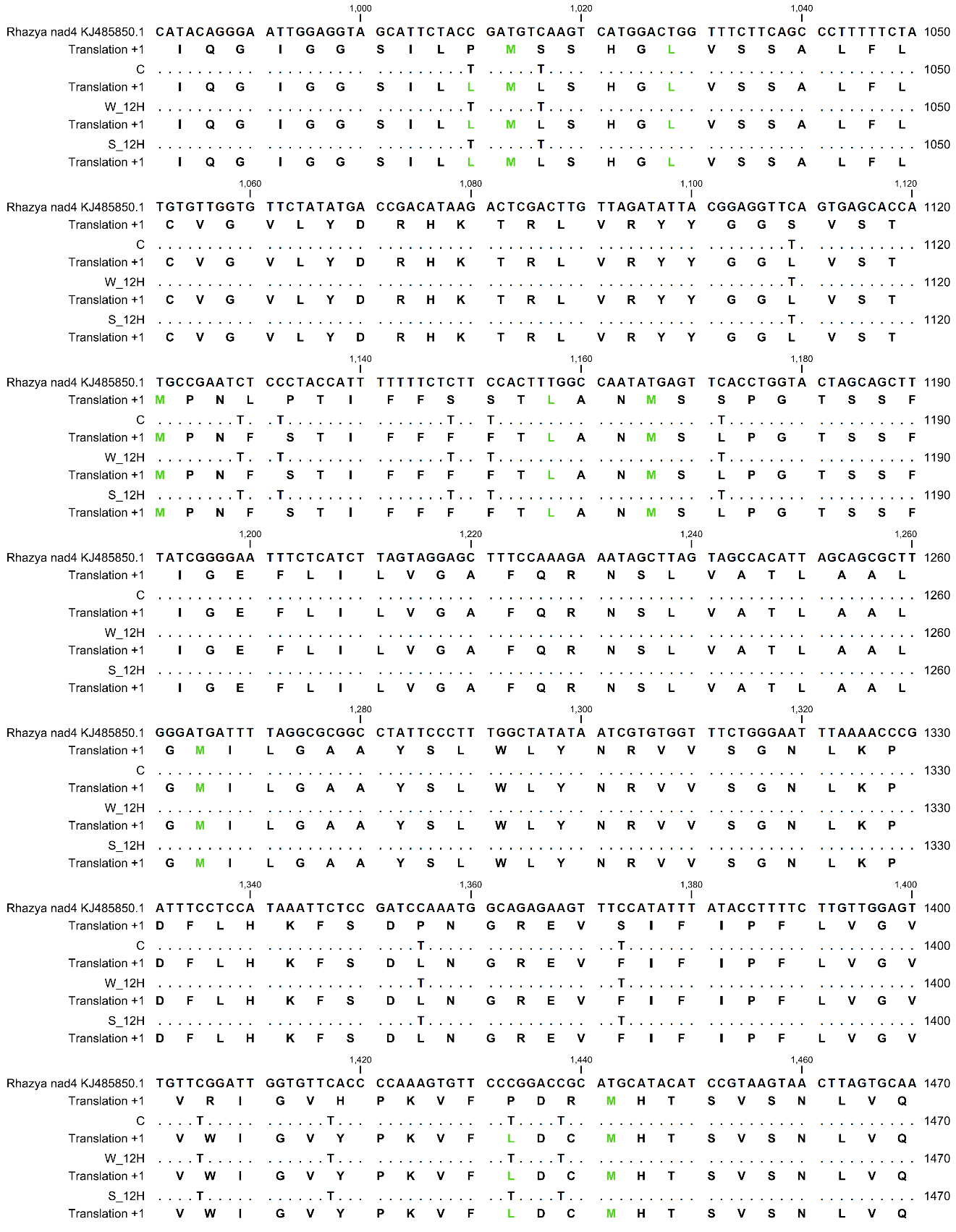

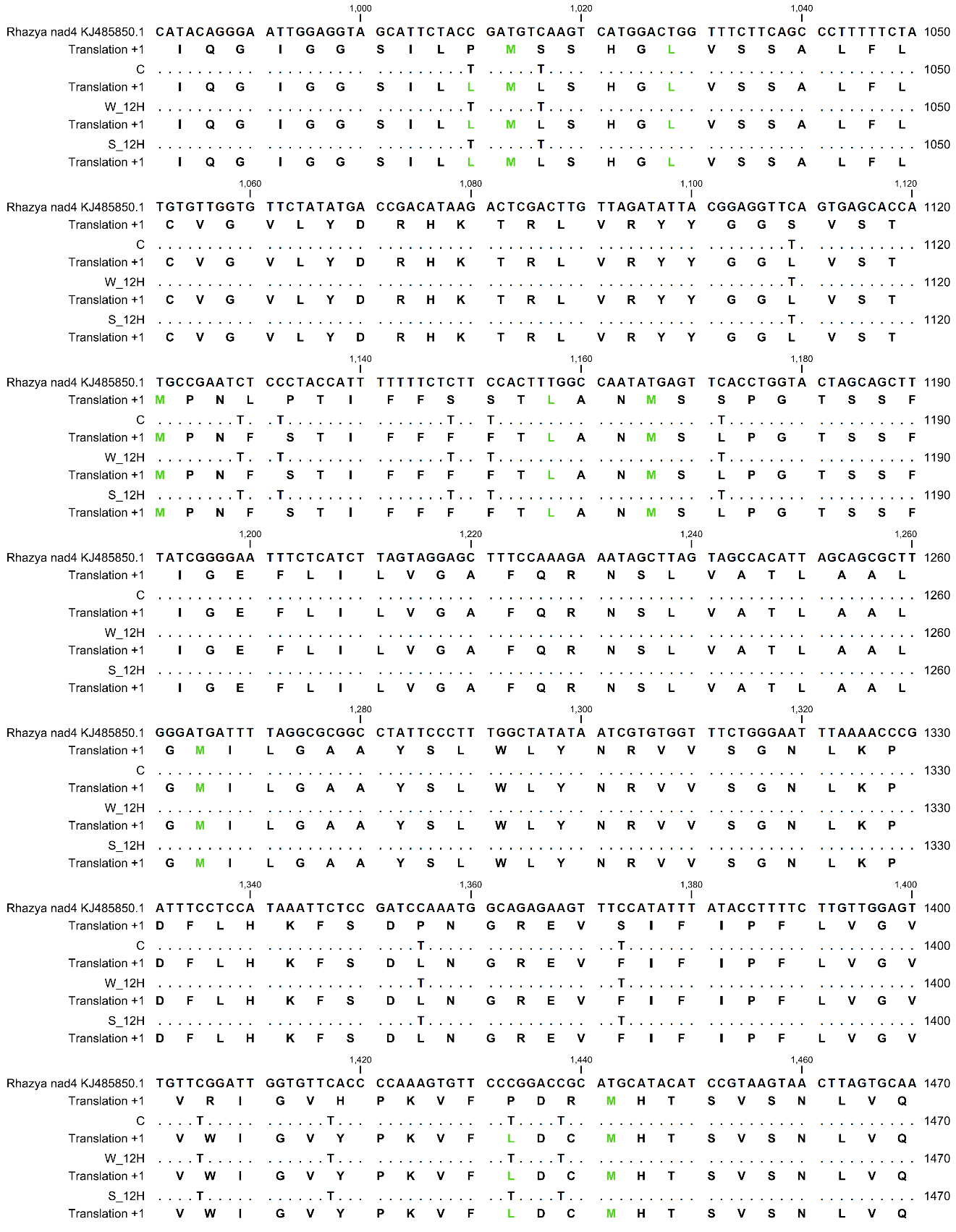

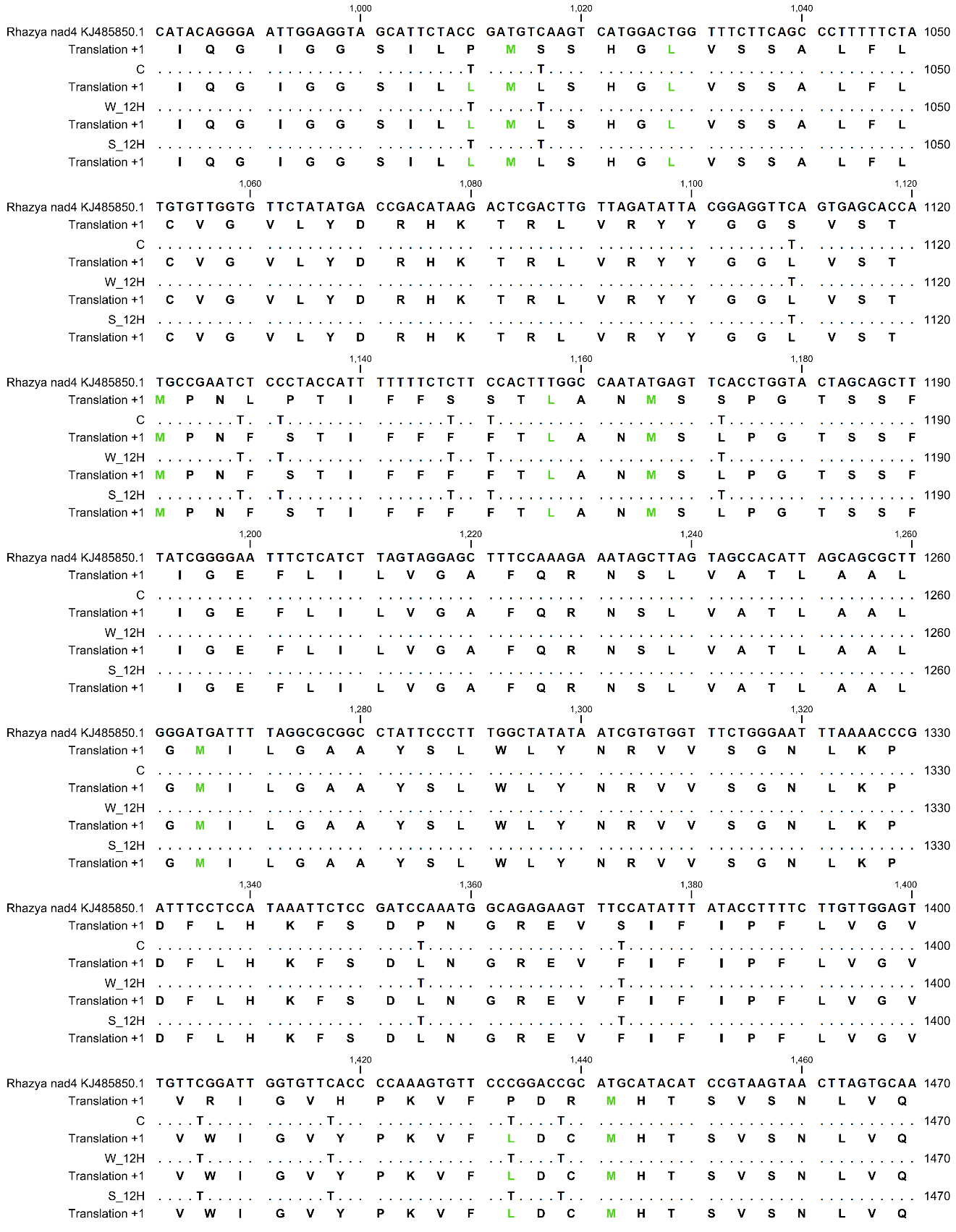

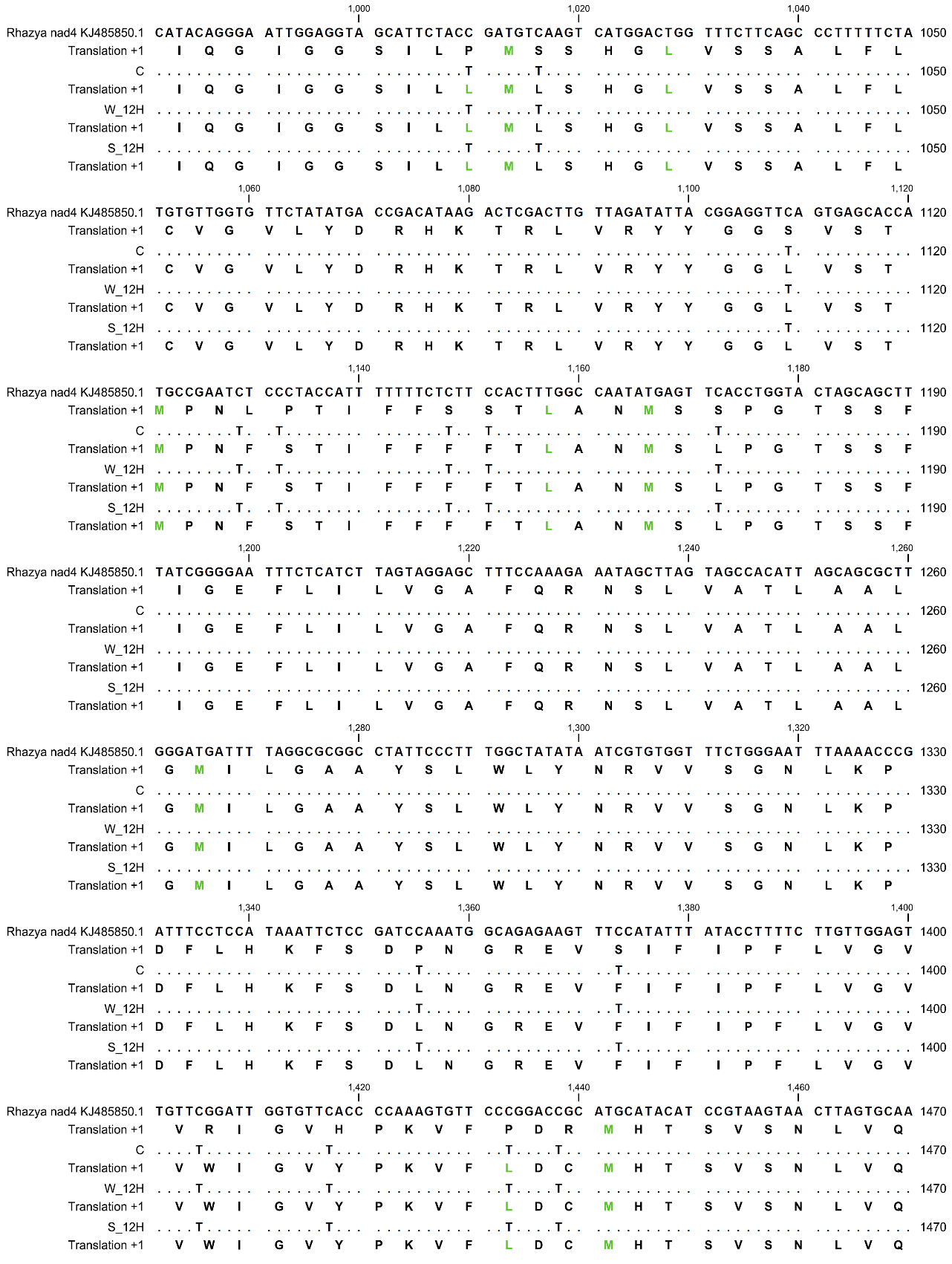


Figure S6. Continued


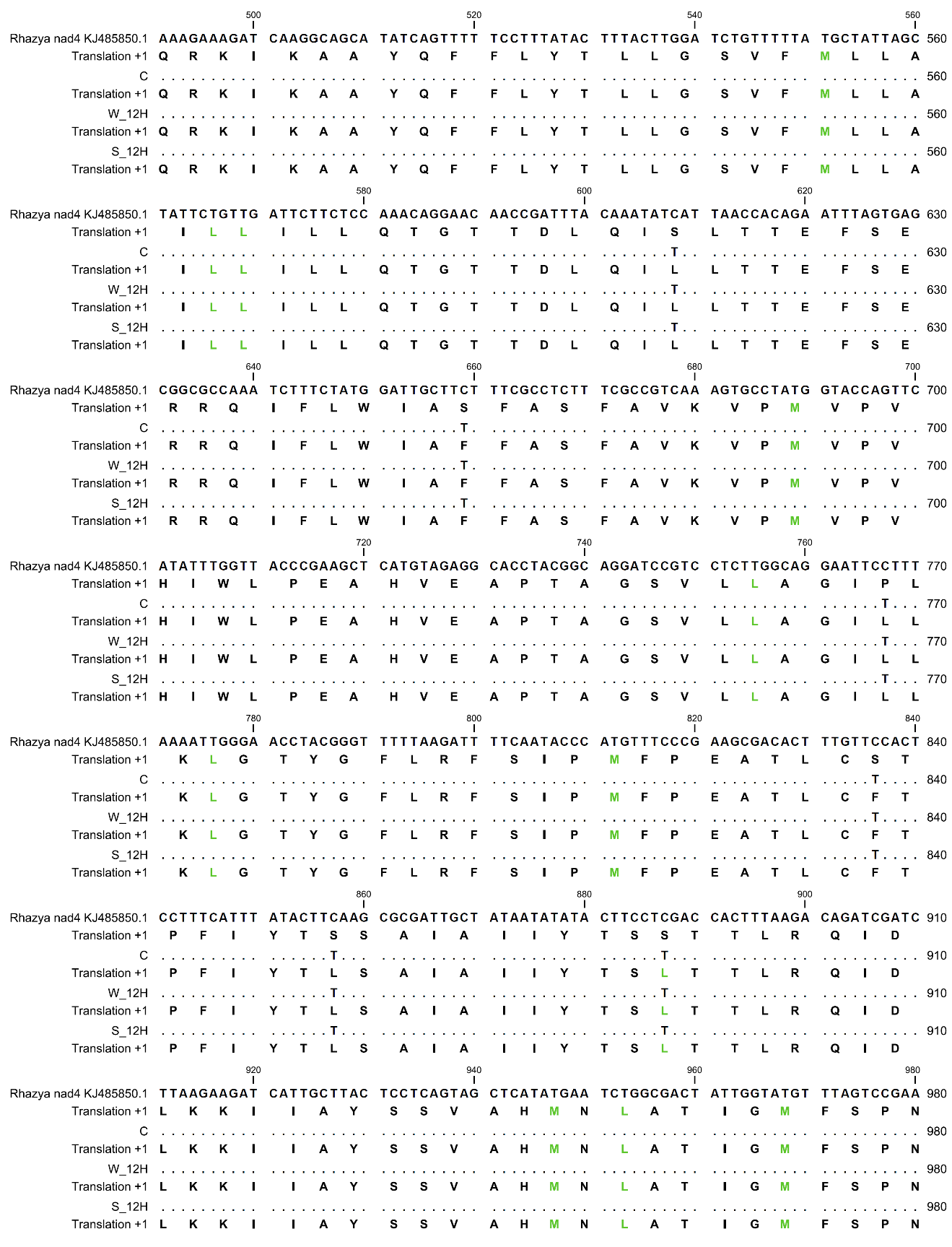

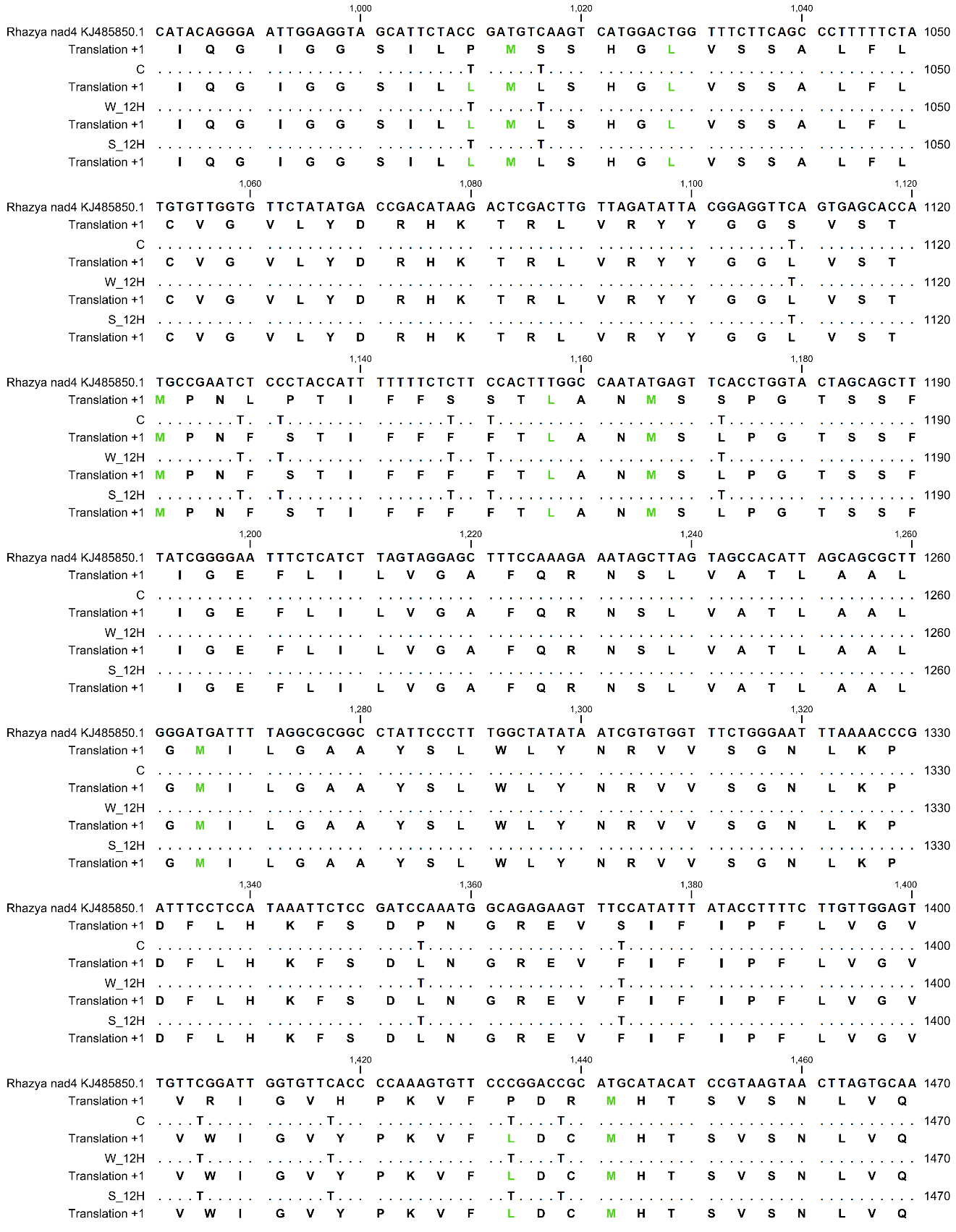

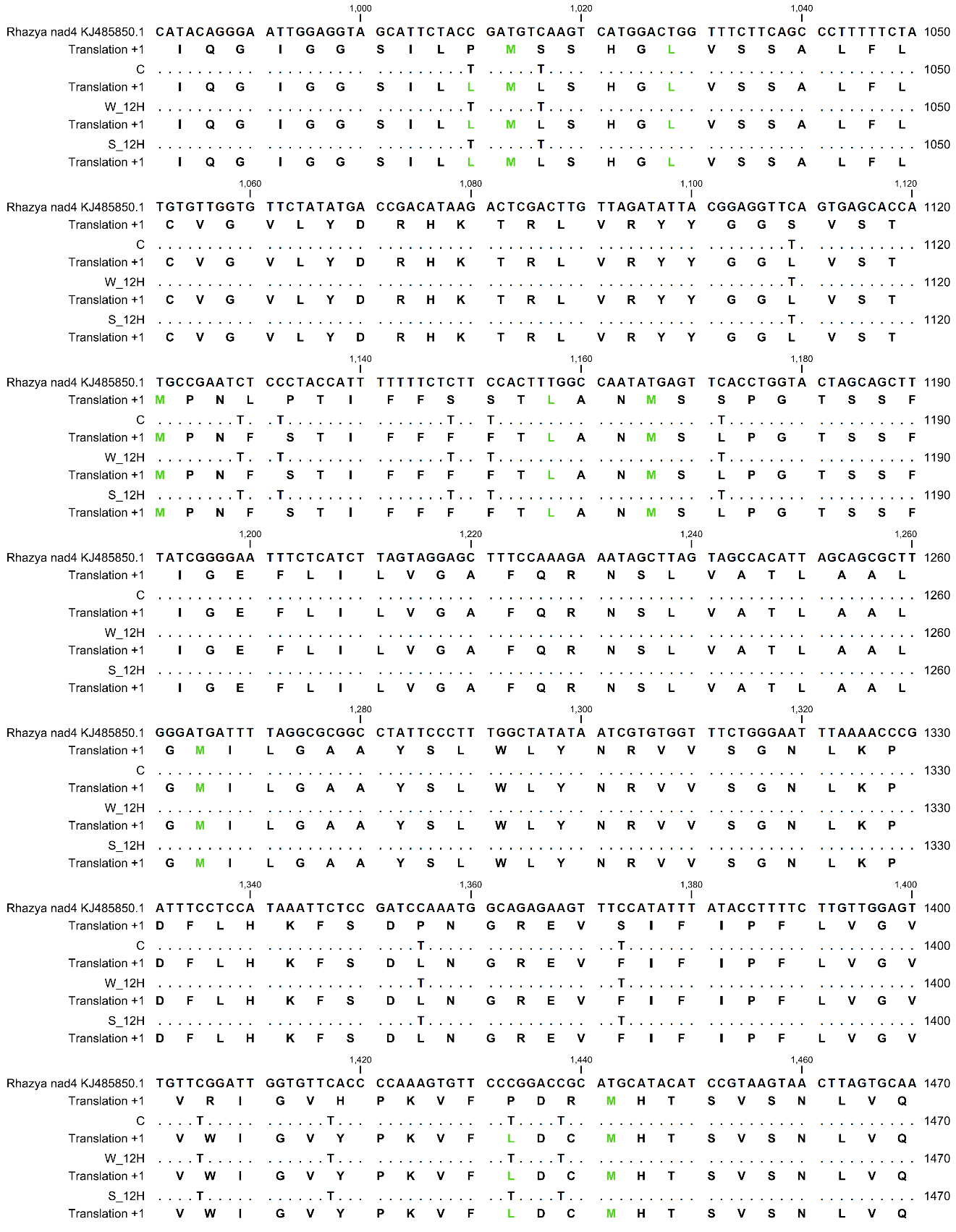

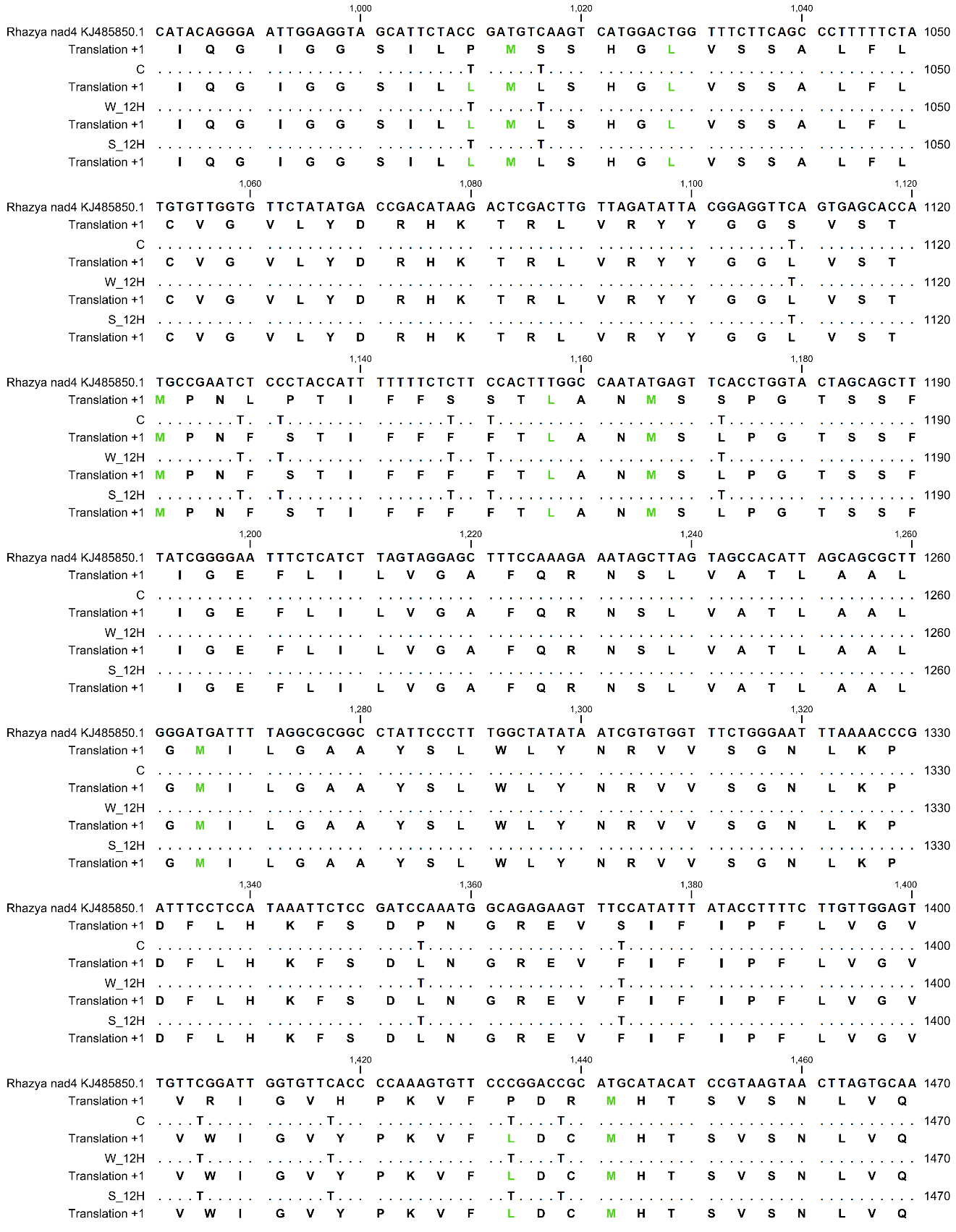

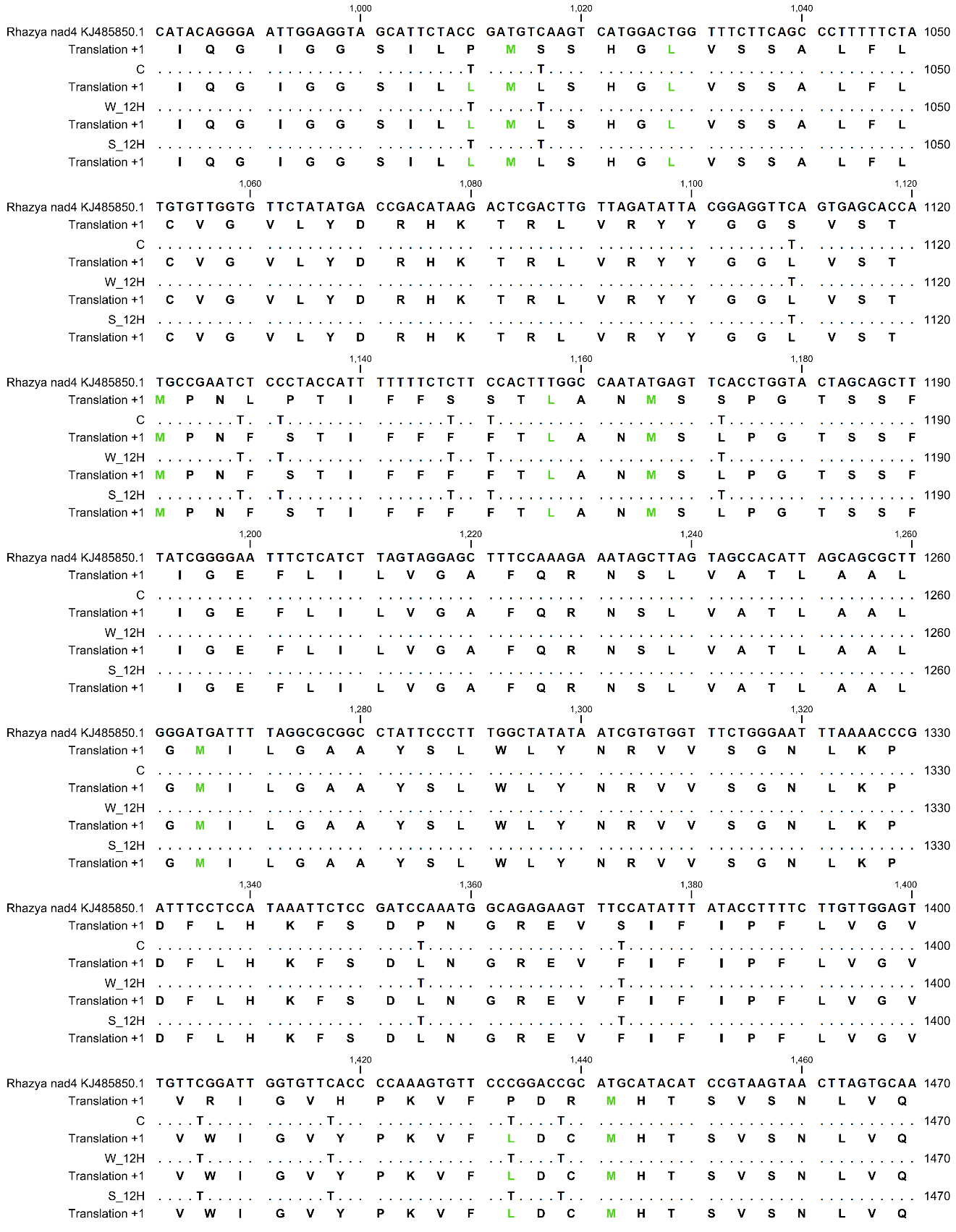

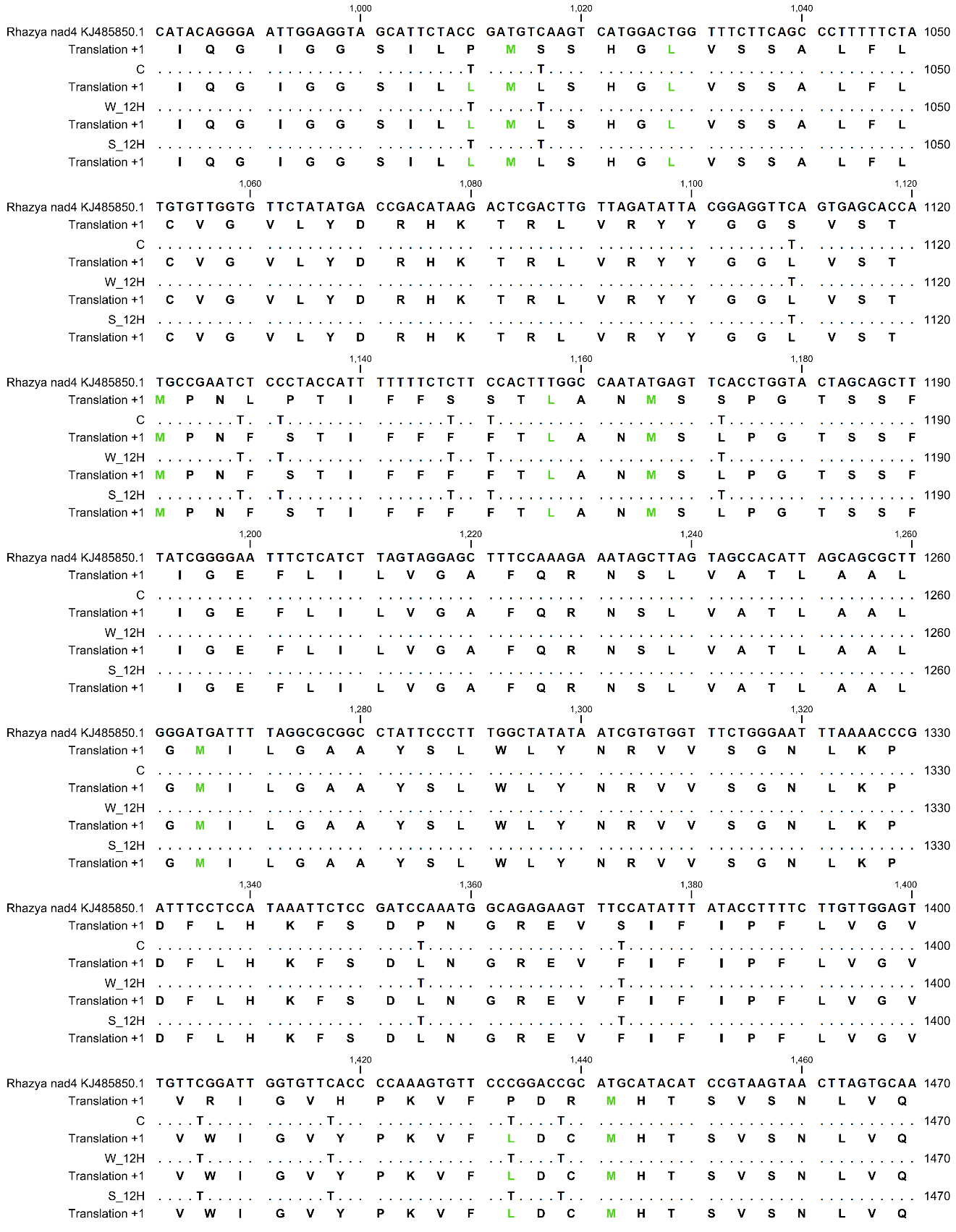

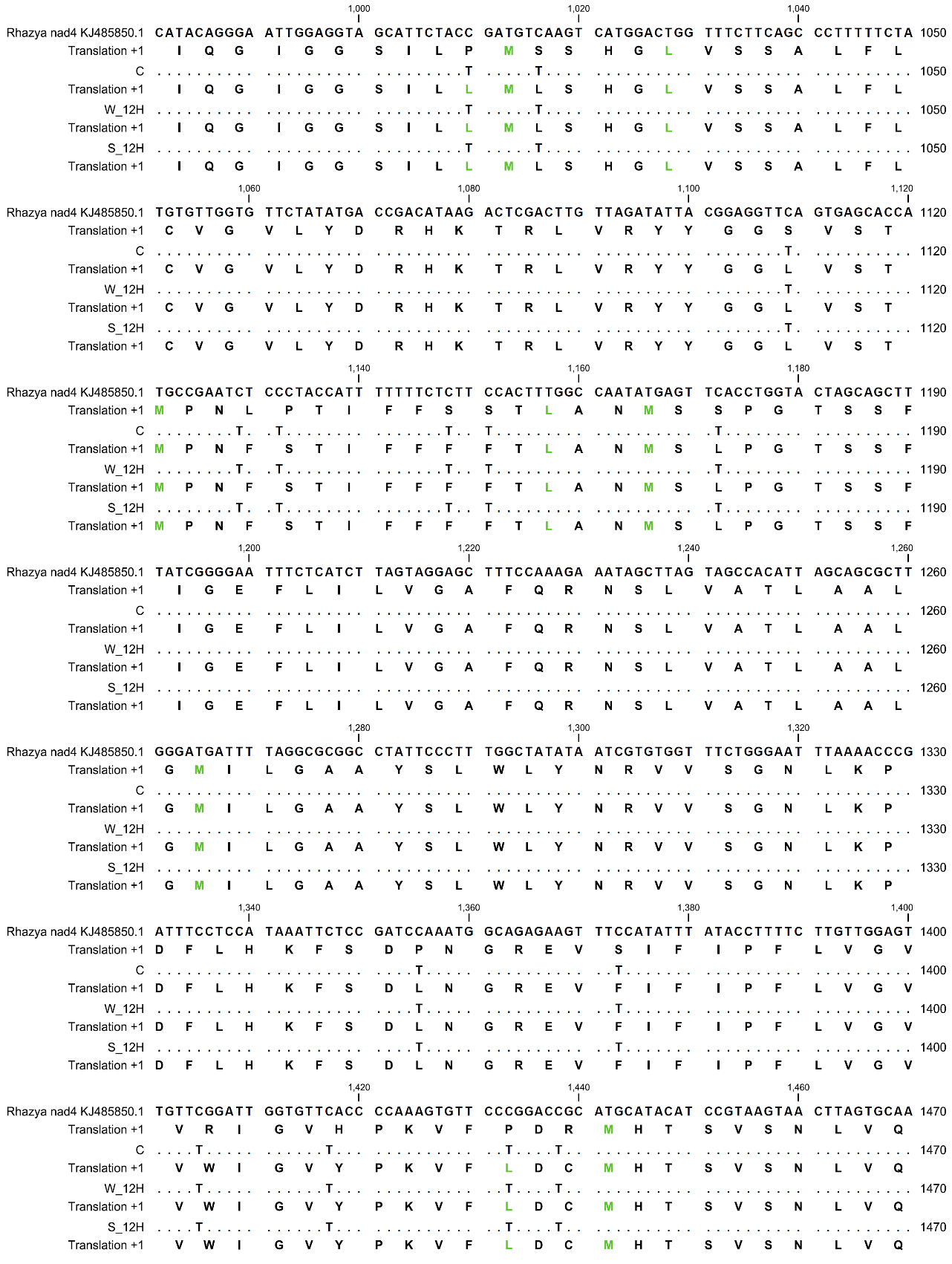


Figure S6. Continued


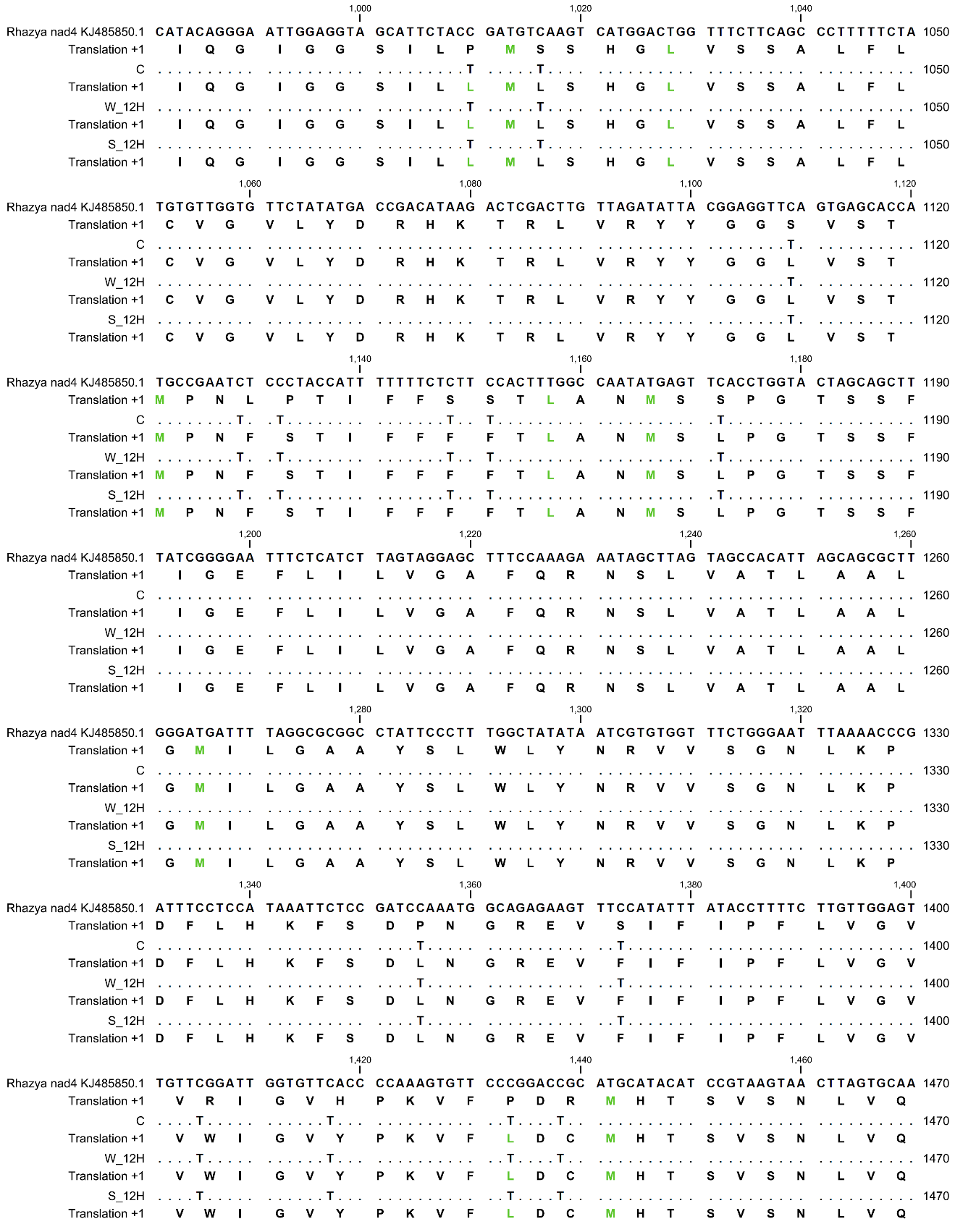

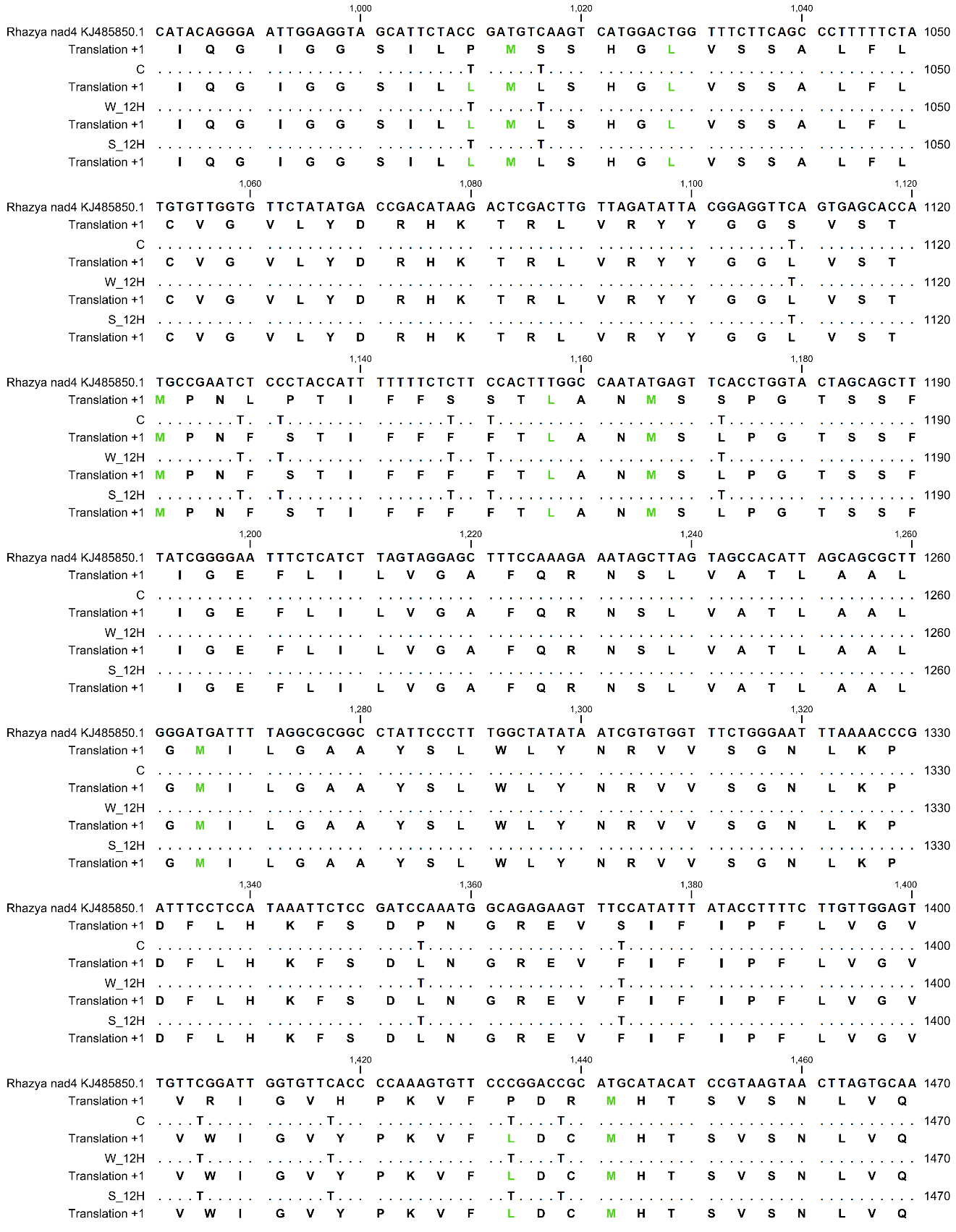

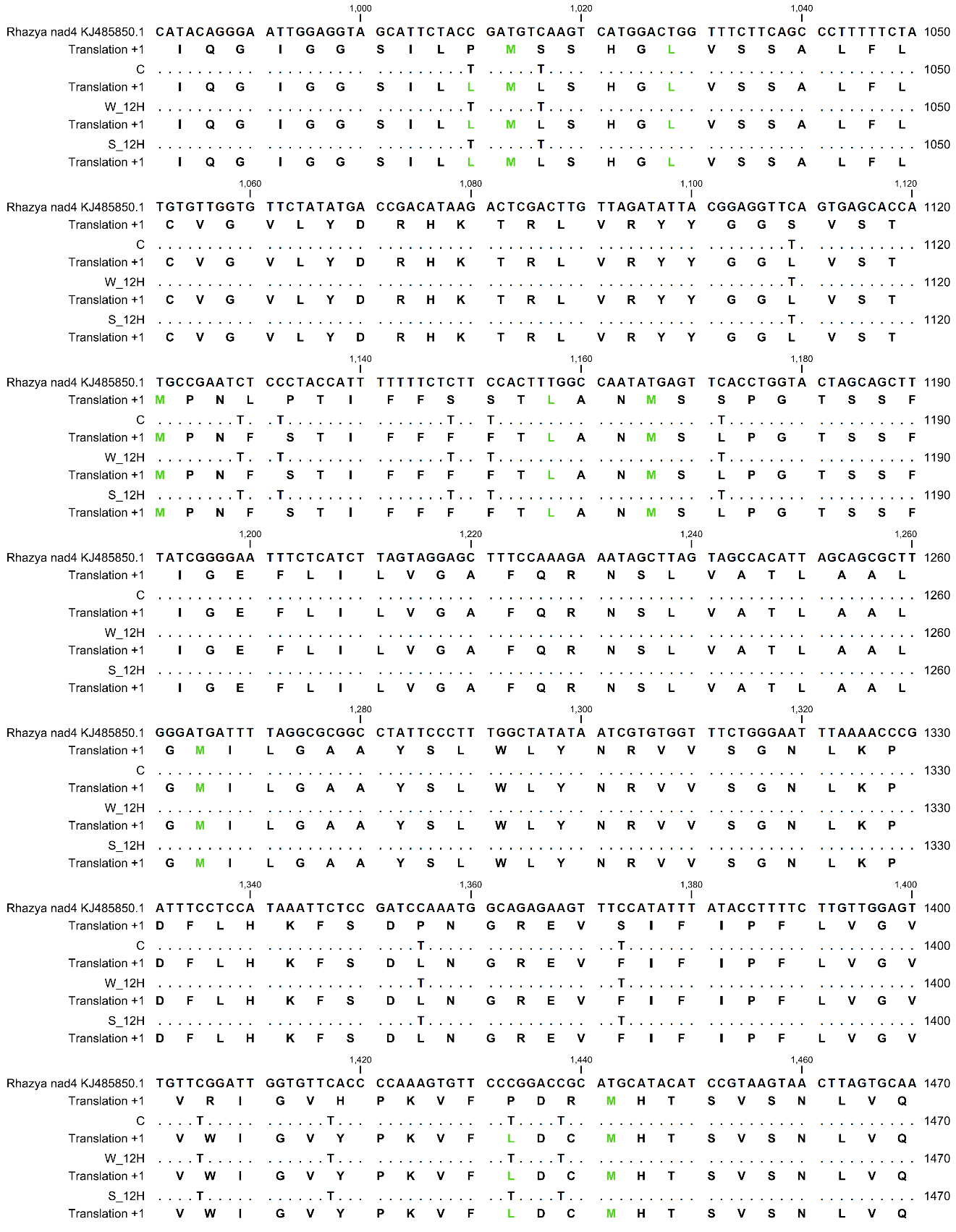

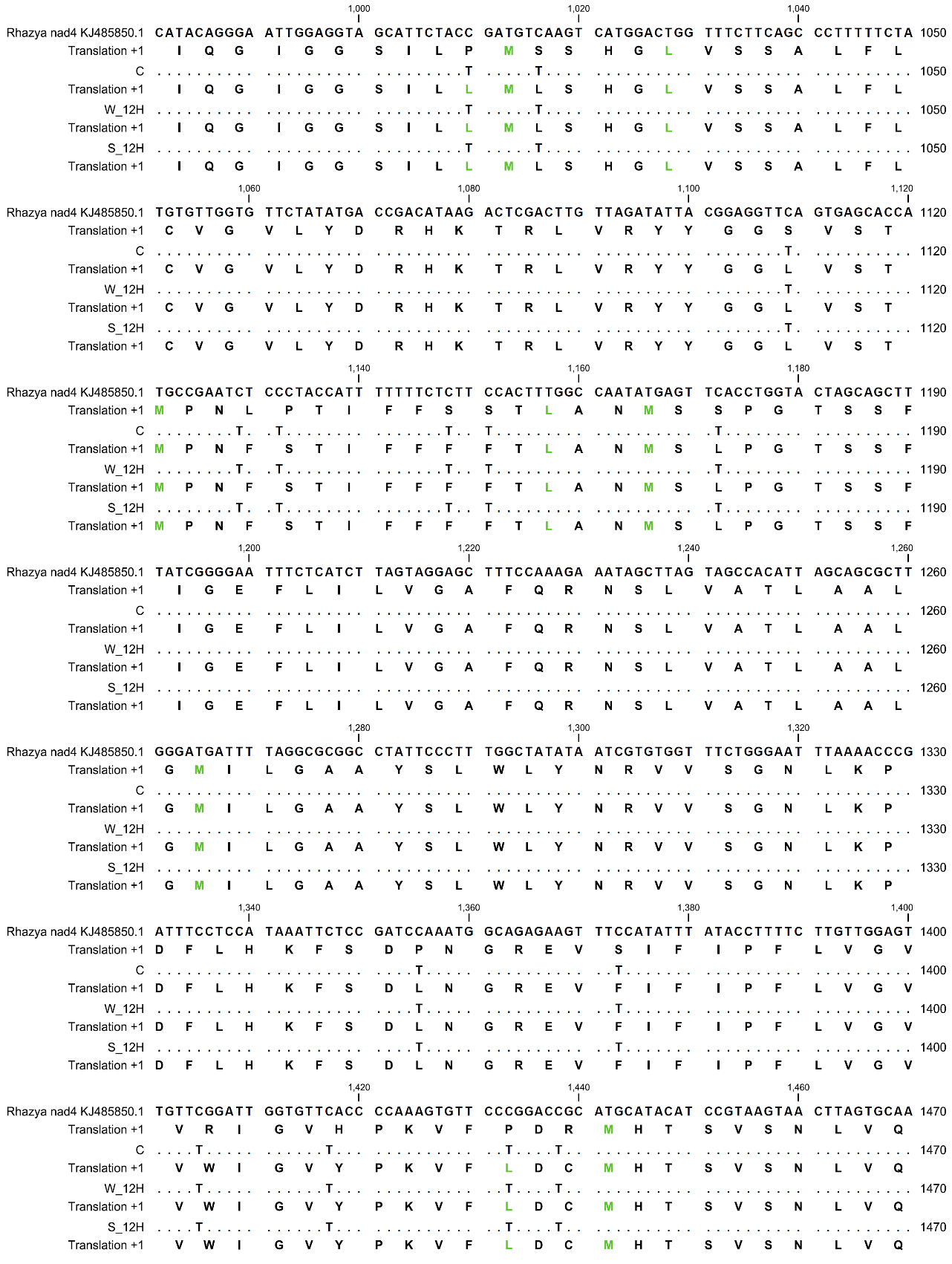

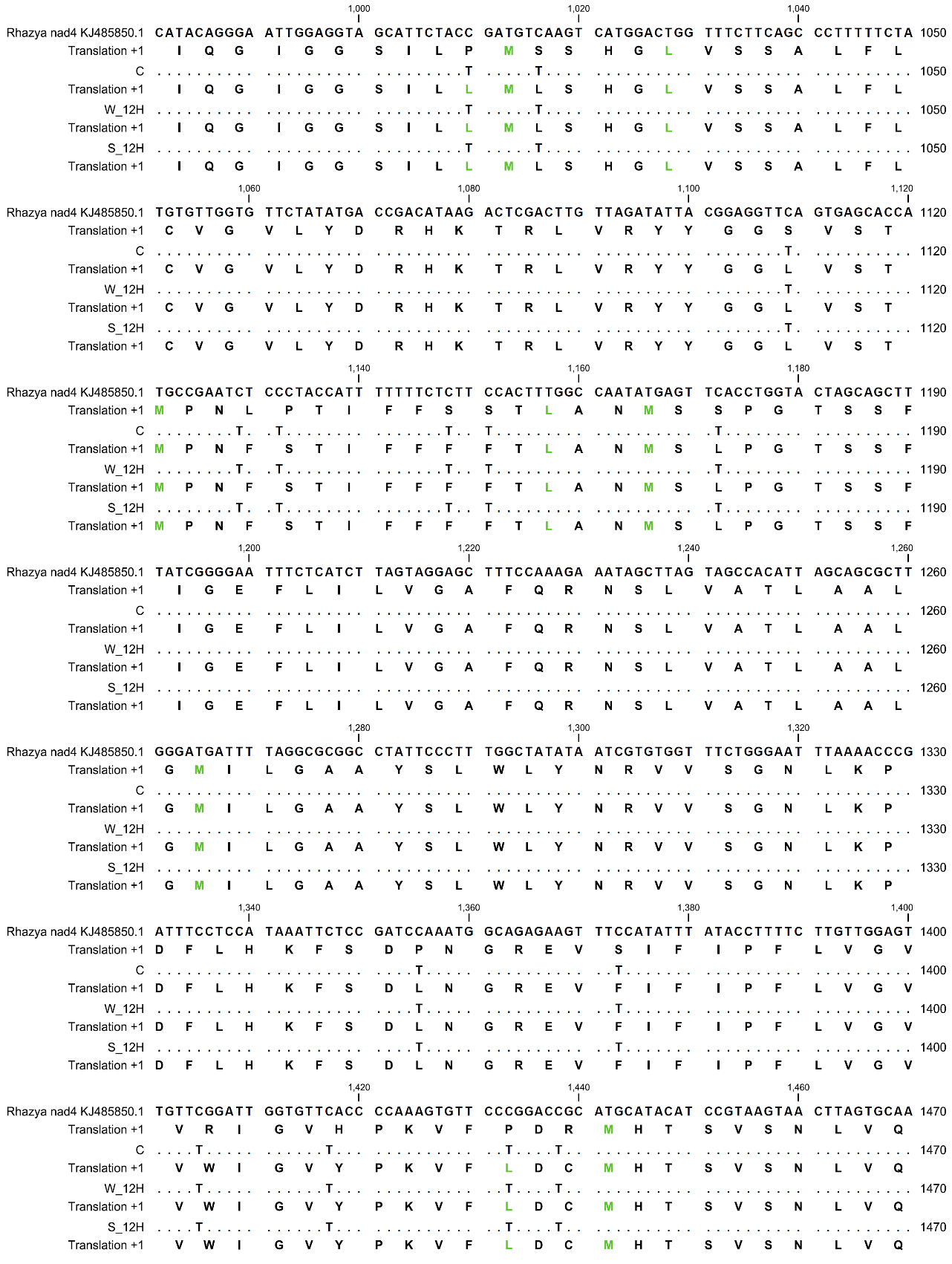


Figure S6. Continued

Supplement: S6 Fig — Five PPRs are known to edit six sites in this transcript, e.g., ORG1 edits two codons (no. 139 and 145), while PPRs MEF26, Agh11, MEF18 and MEF35 edit one codon each (no. 56, 126, 452 or 458, respectively). The six edit sites of codons no. 56, 126, 139, 145, 452 and 458 resulted in the conversion of arginine (CGG) to tryptophan (TGG), arginine (CGT) to cysteine (TGT), proline (CCT) to leucine (CTT), leucine (CTT) to phenylalanine (TTT), proline (CCA) to leucine (CTA) and serine (TCC) to phenylalanine (TTC), respectively. Red rectangles indicate the different edit sites of the known five PPRs, while blue rectangles indicate the other edit sites in this transcript. The letters in the figure indicate the abbreviations of different amino acids. (DOCX) [file pone.0177589.s006.docx]

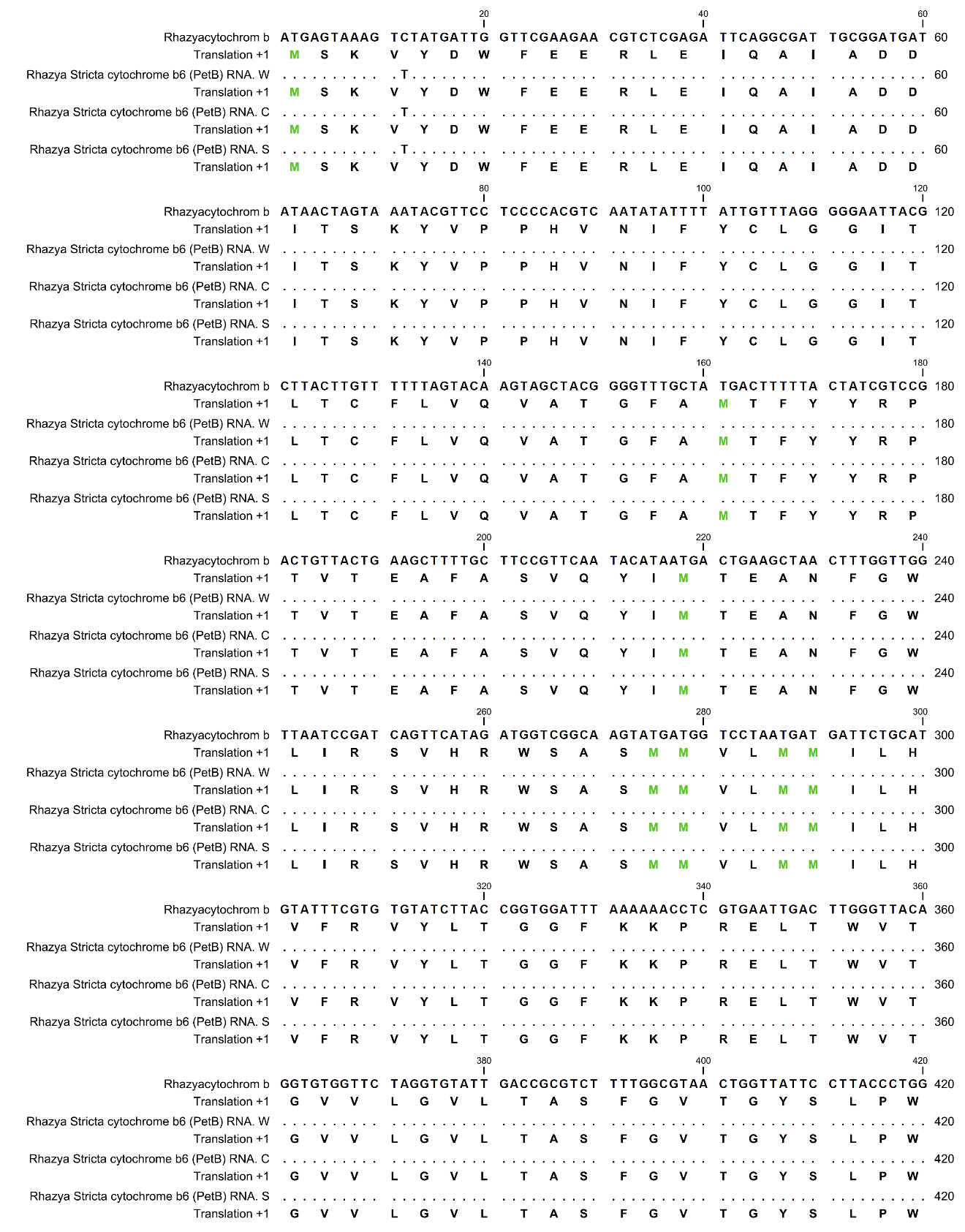
Figure S8.

Figure S8. Continued

Supplement: S8 Fig — Editing of the first site (GTC to GTT) resulted in no change in the amino acid valine (V), while the second edit (CCA to CTA) resulted in the conversion of proline (P) to leucine (L). No PPRs are known for editing either site. The letters in the figure indicate the abbreviations of different amino acids. (DOCX) [file pone.0177589.s008.docx]

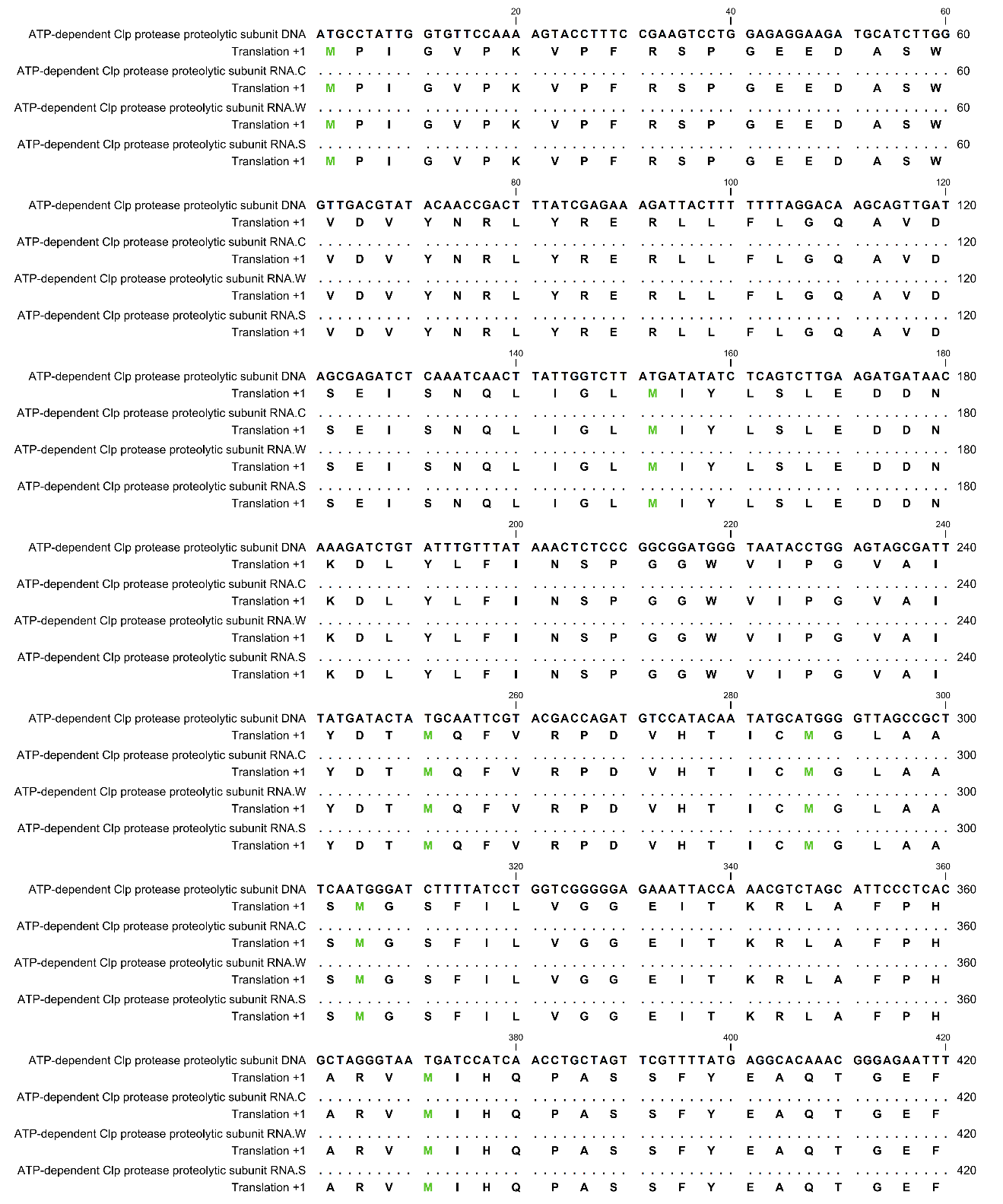
Figure S9.


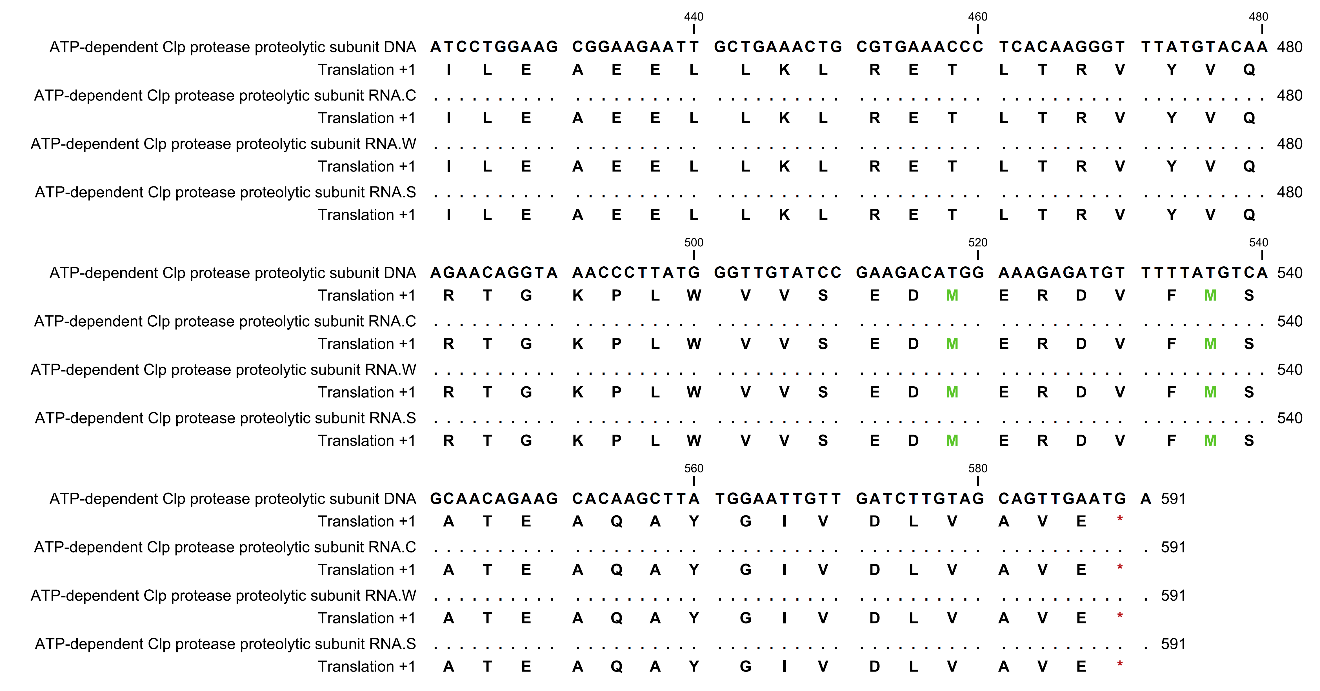
Figure S9. Continued

Supplement: S9 Fig — One PPR, CLB19, is known to edit one site in this transcript to convert histidine to tyrosine in codon number 187 (shown inside the red square). This codon (UAU) in R. stricta normally encodes a conserved tyrosine (Y), hence, requires no editing. The letters in the figure indicate the abbreviations of different amino acids. (DOCX) [file pone.0177589.s009.docx]
