# Supplementary material for: Transcriptomic analysis of salt stress responsive genes in Rhazya stricta
Source: PLoS One. 2017 May 16;12(5):e0177589. doi: 10.1371/journal.pone.0177589 (PMC5433744; doi:10.1371/journal.pone.0177589)
Supplement: S4 Fig — Information about the different genes is shown in S1 and S2 Tables [29]. (DOCX) [file pone.0177589.s004.docx]

Figure S4.

**Apical**

**Mature**
