# Supplementary material for: Transcriptomic analysis of salt stress responsive genes in Rhazya stricta
Source: PLoS One. 2017 May 16;12(5):e0177589. doi: 10.1371/journal.pone.0177589 (PMC5433744; doi:10.1371/journal.pone.0177589)
Supplement: S7 Fig — Editing resulted in the conversion of serine (S) codon (TCA) to leucine (L) codon (TTA). Two PPRs, OTP84 and ECB2, compete to edit this site in the transcript. The letters in the figure indicate the abbreviations of different amino acids. (DOCX) [file pone.0177589.s007.docx]

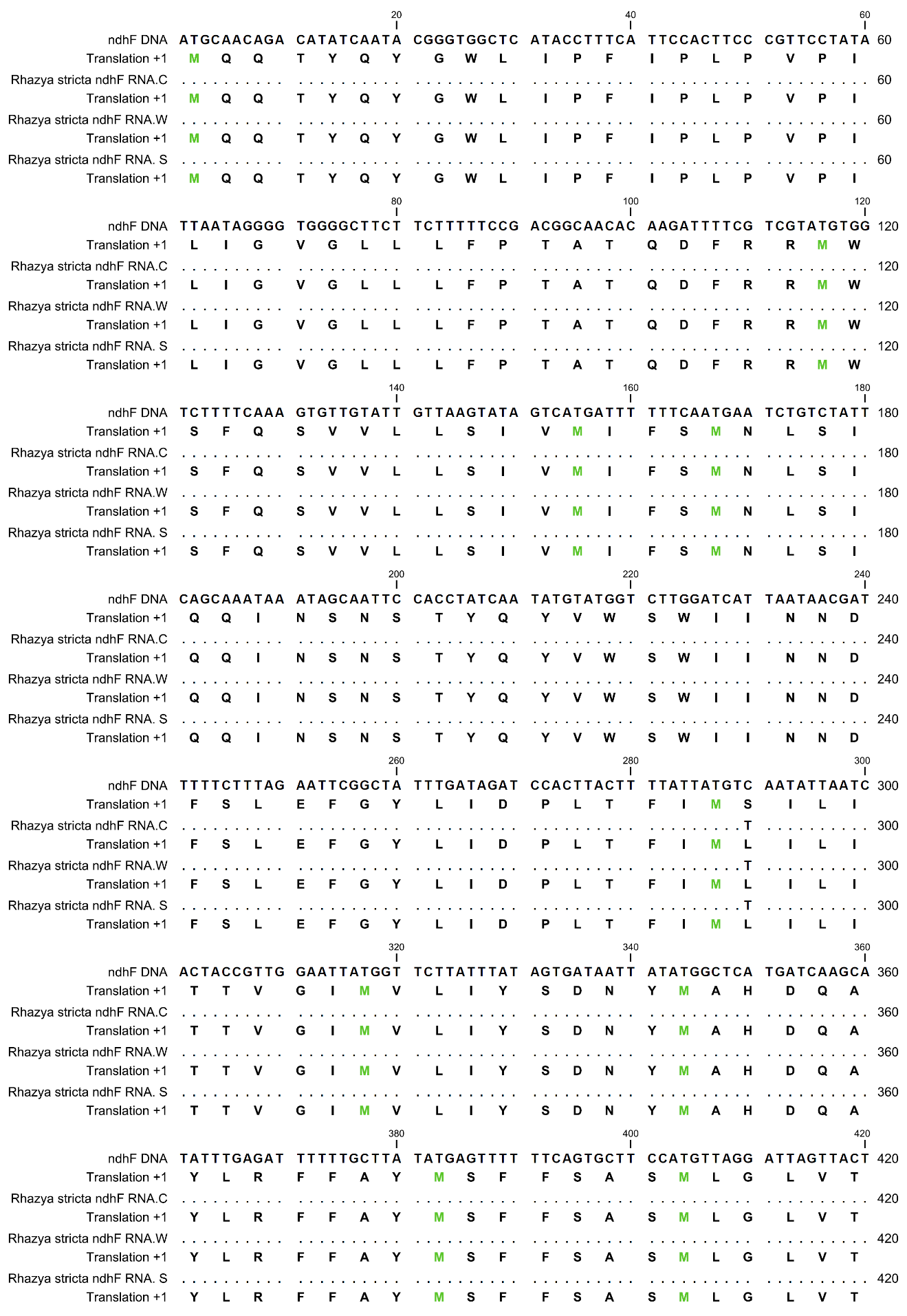
Figure S7. Figure S7. Continued
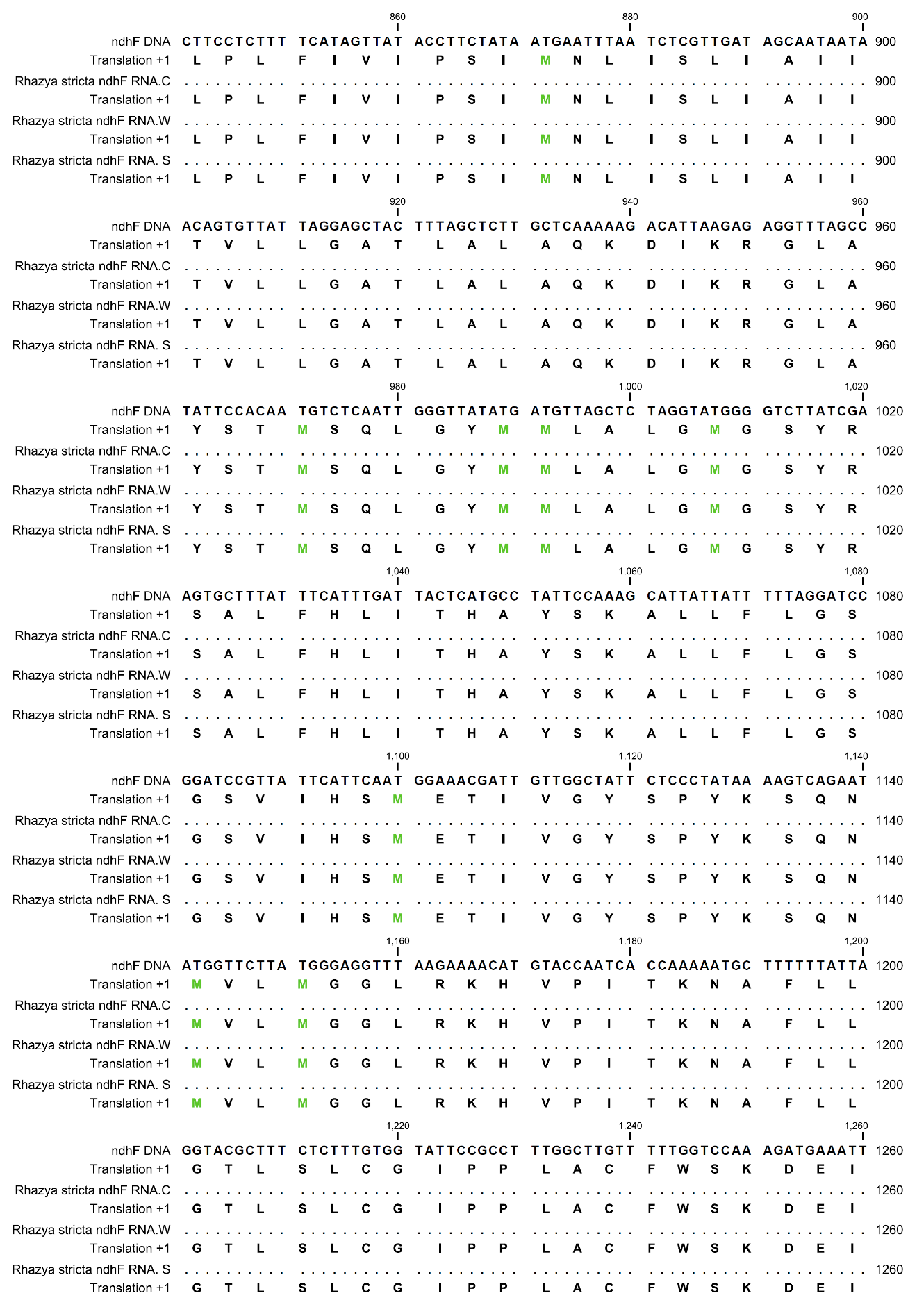
Figure S7. Continued
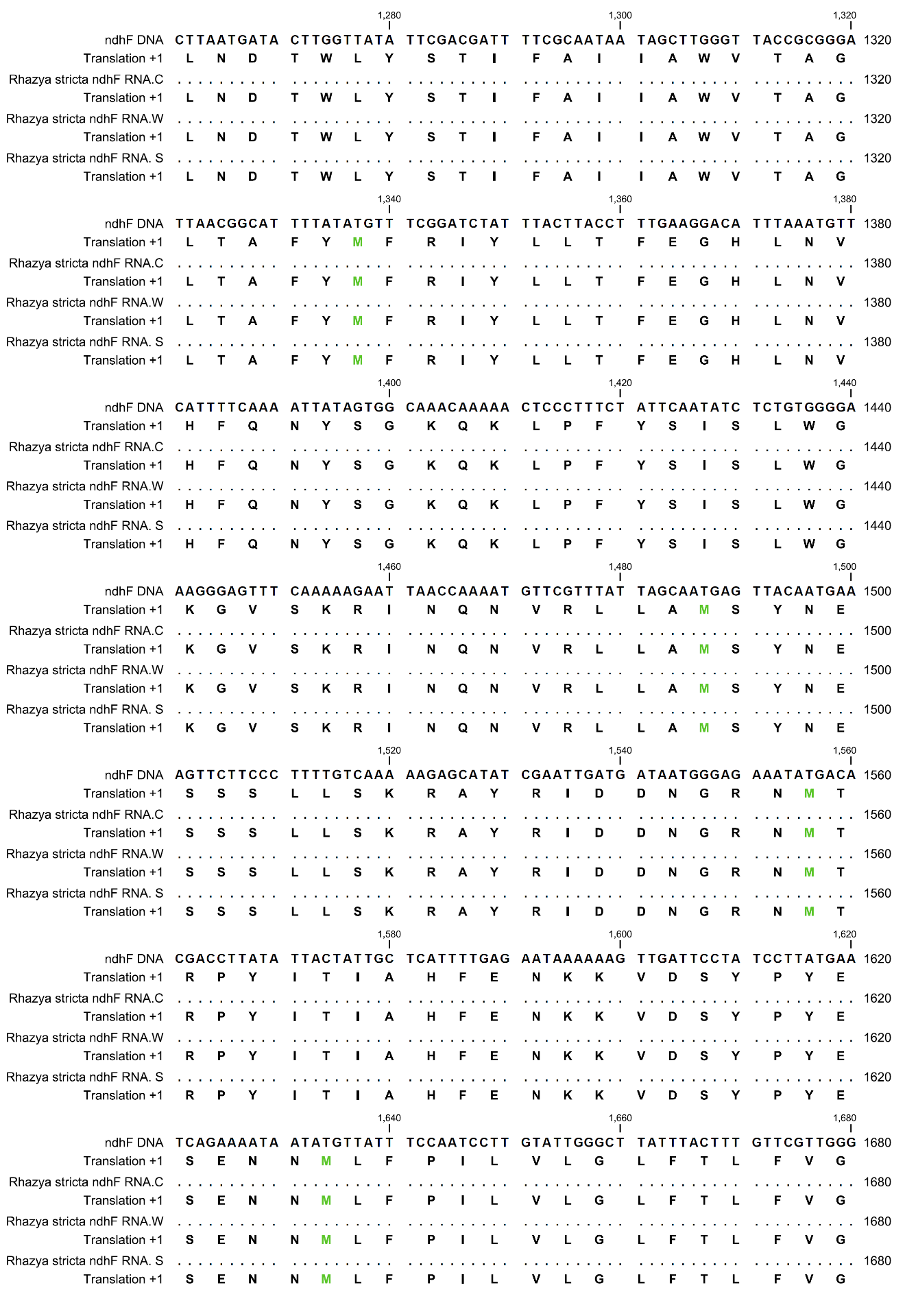
Figure S7. Continued
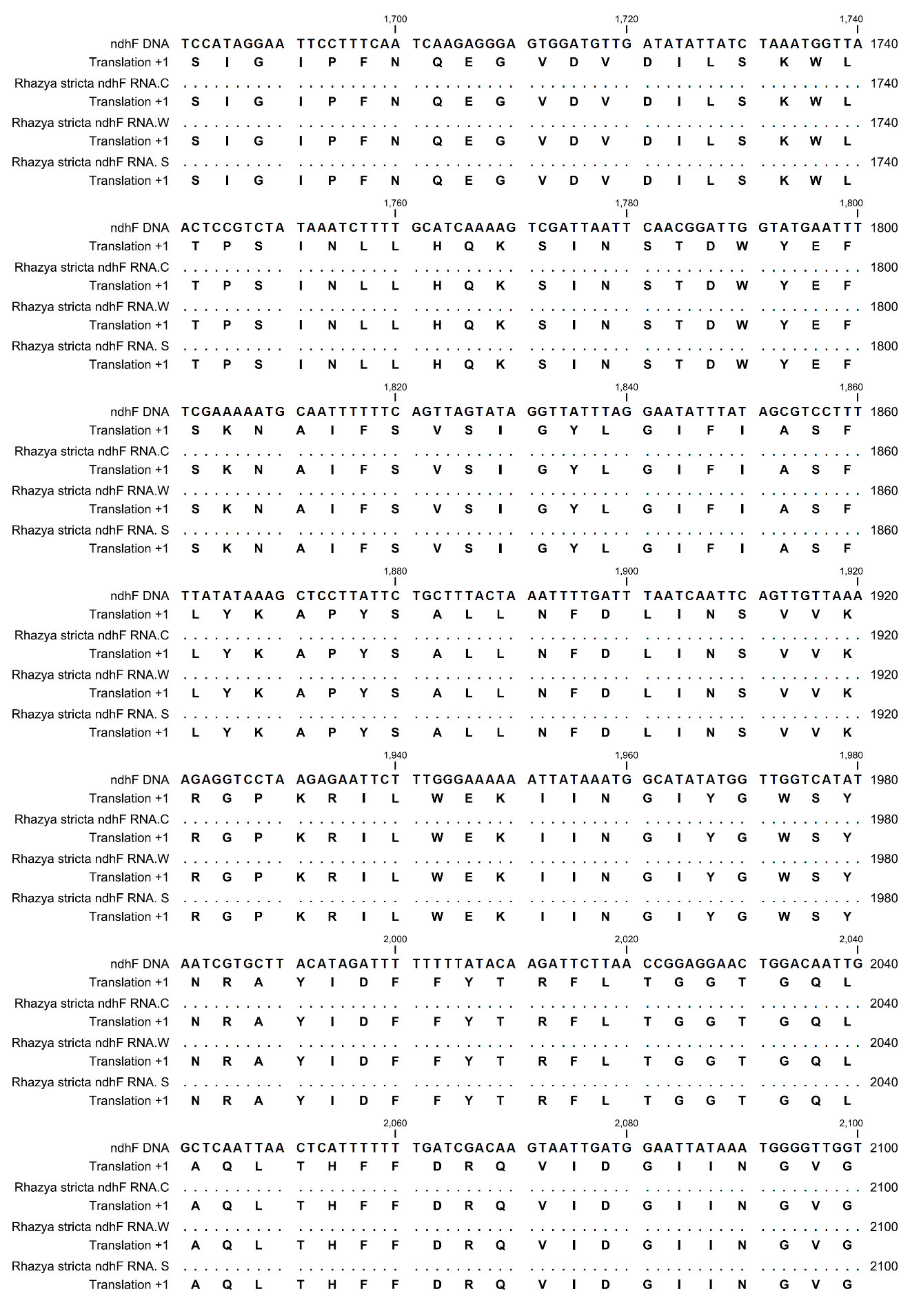
Figure S7. Continued

Figure S7. Continued
