# Supplementary material for: Transcriptomic analysis of salt stress responsive genes in Rhazya stricta
Source: PLoS One. 2017 May 16;12(5):e0177589. doi: 10.1371/journal.pone.0177589 (PMC5433744; doi:10.1371/journal.pone.0177589)
Supplement: S4 Table — Blue box = upregulation, orange box = downregulation. (DOCX) [file pone.0177589.s013.docx]

Table S4. Comparative differential expression of genes of *R.* *stricta* transcriptomes under salt (500 mM NaCl) treatments for 0 h, 2 h, 12 h and 24 h. Blue box = upregulation, orange box = downregulation. Green boxes indicate genes selected for further analysis. Transcripts in bold will be further analyzed.

| Transcript ID | Description | 0 h vs. | | |
| --- | --- | --- | --- | --- |
|  |  | 2 h | 12 h | 24 h |
| snap_masked-superscaffold2-processed-gene-47.15-mRNA-1 | abc transporter g family member 11-like |  |  |  |
| maker-superscaffold5-snap-gene-11.20-mRNA-1 | abc transporter g family member 1-like |  |  |  |
| snap_masked-superscaffold7-processed-gene-14.2-mRNA-1 | abc transporter g family member 20-like |  |  |  |
| maker-superscaffold2-snap-gene-64.36-mRNA-1 | actin-depolymerizing factor 1 |  |  |  |
| snap_masked-scaffold3_751-processed-gene-19.20-mRNA-1 | agamous-like mads-box protein agl31-like |  |  |  |
| maker-superscaffold13-snap-gene-81.21-mRNA-1 | agamous-like mads-box protein agl8 isoform 1 |  |  |  |
| maker-superscaffold3-snap-gene-36.29-mRNA-1 | alanine racemase |  |  |  |
| maker-scaffold3_748-snap-gene-2.21-mRNA-1 | annexin d3-like |  |  |  |
| maker-superscaffold9-snap-gene-30.28-mRNA-1 | ap2 domain-containing transcription factor family protein |  |  |  |
| maker-superscaffold10-snap-gene-68.45-mRNA-1 | ap2-like ethylene-responsive transcription factor |  |  |  |
| maker-superscaffold13-snap-gene-93.28-mRNA-1 | apoptosis-enhancing nuclease-like |  |  |  |
| maker-superscaffold11-snap-gene-6.16-mRNA-1 | basic helix-loop-helix dna-binding superfamily isoform 3 |  |  |  |
| maker-superscaffold17-snap-gene-23.23-mRNA-1 | beta-glucosidase 44-like |  |  |  |
| snap_masked-superscaffold21-processed-gene-64.1-mRNA-1 | blue copper |  |  |  |
| maker-superscaffold13-snap-gene-36.22-mRNA-1 | btb and taz domain protein 2 isoform 1 |  |  |  |
| snap_masked-superscaffold12-processed-gene-84.0-mRNA-1 | calcium-binding protein cml38-like |  |  |  |
| maker-superscaffold28-snap-gene-16.20-mRNA-1 | calcium-dependent protein kinase 34-like |  |  |  |
| maker-scaffold3_955-snap-gene-1.20-mRNA-1 | caleosin-related family protein |  |  |  |
| maker-superscaffold22-snap-gene-2.25-mRNA-1 | casp-like protein poptrdraft_798217-like |  |  |  |
| maker-superscaffold43-snap-gene-9.27-mRNA-1 | chaperone protein dnaj 6-like |  |  |  |
| snap_masked-superscaffold6-processed-gene-20.8-mRNA-1 | class iii peroxidase 70 |  |  |  |
| maker-superscaffold13-snap-gene-105.37-mRNA-1 | core-2 i-branching beta- -n-acetylglucosaminyltransferase family |  |  |  |
| maker-superscaffold7-snap-gene-8.22-mRNA-1 | cyclic nucleotide-gated channel 15 isoform 1 |  |  |  |
| snap_masked-superscaffold6-processed-gene-30.17-mRNA-1 | cyclin-dependent kinase f-4-like |  |  |  |
| maker-superscaffold20-snap-gene-6.19-mRNA-1 | cytochrome family subfamily polypeptide 6 |  |  |  |
| snap_masked-superscaffold6-processed-gene-108.3-mRNA-1 | cytochrome p450 716b2-like |  |  |  |
| snap_masked-superscaffold21-processed-gene-44.12-mRNA-1 | cytochrome p450 86a1 |  |  |  |
| snap_masked-superscaffold15-processed-gene-57.23-mRNA-1 | cytochrome p450 86a2-like |  |  |  |
| snap_masked-superscaffold32-processed-gene-12.8-mRNA-1 | cytochrome p450 86b1-like |  |  |  |
| maker-superscaffold6-snap-gene-99.28-mRNA-1 | cytochrome p450 98a2-like |  |  |  |
| maker-superscaffold9-snap-gene-3.23-mRNA-1 | defensin-like protein p322-like |  |  |  |
| maker-superscaffold21-snap-gene-19.35-mRNA-1 | duf21 domain-containing protein at5g52790-like |  |  |  |
| snap_masked-superscaffold9-processed-gene-39.14-mRNA-1 | er membrane protein complex subunit 2-like |  |  |  |
| maker-superscaffold17-snap-gene-5.26-mRNA-1 | expansin precursor |  |  |  |
| maker-superscaffold1-snap-gene-56.20-mRNA-1 | extra-large gtp-binding protein 3 |  |  |  |
| maker-superscaffold6-snap-gene-17.23-mRNA-1 | f-box and associated interaction domains-containing isoform 1 |  |  |  |
| maker-superscaffold28-snap-gene-35.24-mRNA-1 | gdsl esterase lipase at1g74460-like |  |  |  |
| maker-superscaffold13-snap-gene-45.23-mRNA-1 | gdsl esterase lipase at2g23540-like |  |  |  |
| maker-scaffold3_605-snap-gene-0.17-mRNA-1 | germin-like protein subfamily 1 member 13-like |  |  |  |
| maker-superscaffold8-snap-gene-46.29-mRNA-1 | glutamate receptor –like |  |  |  |
| maker-superscaffold13-snap-gene-95.30-mRNA-1 | glutathione s-transferase u9 |  |  |  |
| maker-superscaffold8-snap-gene-36.23-mRNA-1 | g-type lectin s-receptor-like serine threonine-protein kinase |  |  |  |
| maker-superscaffold6-snap-gene-2.24-mRNA-1 | homeobox-leucine zipper protein athb-7-like |  |  |  |
| snap_masked-superscaffold3-processed-gene-10.5-mRNA-1 | invertase inhibitor |  |  |  |
| snap_masked-superscaffold6-processed-gene-128.10-mRNA-1 | kda proline-rich partial |  |  |  |
| snap_masked-superscaffold28-processed-gene-55.18-mRNA-1 | lignin-forming anionic peroxidase |  |  |  |
| maker-superscaffold21-snap-gene-70.28-mRNA-1 | lob domain-containing protein 15 |  |  |  |
| snap_masked-superscaffold2-processed-gene-26.6-mRNA-1 | lob domain-containing protein 22 |  |  |  |
| maker-superscaffold17-snap-gene-60.27-mRNA-1 | low quality protein: isoflavone 2 -hydroxylase- partial |  |  |  |
| maker-superscaffold10-snap-gene-37.11-mRNA-1 | lysine decarboxylase family protein isoform 2 |  |  |  |
| maker-superscaffold17-snap-gene-46.17-mRNA-1 | mads-box transcription factor 27 |  |  |  |
| snap_masked-superscaffold13-processed-gene-17.14-mRNA-1 | mediator of rna polymerase ii transcription subunit 21-like |  |  |  |
| snap_masked-superscaffold8-processed-gene-52.3-mRNA-1 | methyl esterase |  |  |  |
| maker-scaffold3_955-snap-gene-2.43-mRNA-1 | mitochondrial import inner membrane translocase subunit tim14-1 |  |  |  |
| maker-superscaffold43-snap-gene-13.16-mRNA-1 | mlp-like protein 34-like |  |  |  |
| maker-superscaffold4-snap-gene-24.39-mRNA-1 | myb domain protein |  |  |  |
| maker-superscaffold21-snap-gene-43.19-mRNA-1 | nac domain containing protein 25 |  |  |  |
| maker-superscaffold14-snap-gene-44.24-mRNA-1 | nitrate transporter |  |  |  |
| maker-scaffold3_609-snap-gene-21.28-mRNA-1 | non-functional nadph-dependent codeinone reductase 2-like |  |  |  |
| maker-superscaffold13-snap-gene-77.33-mRNA-1 | patatin group a-3-like |  |  |  |
| snap_masked-superscaffold17-processed-gene-29.14-mRNA-1 | pathogenesis-related protein 5-like |  |  |  |
| maker-superscaffold10-snap-gene-16.39-mRNA-1 | pectin lyase-like superfamily protein isoform 1 |  |  |  |
| maker-superscaffold1-snap-gene-21.30-mRNA-1 | peroxidase 3-like |  |  |  |
| maker-superscaffold21-snap-gene-71.28-mRNA-1 | peroxidase 52-like isoform 1 |  |  |  |
| maker-superscaffold21-snap-gene-34.30-mRNA-1 | peroxidase 5-like |  |  |  |
| maker-superscaffold15-snap-gene-110.45-mRNA-1 | peroxidase 68 |  |  |  |
| maker-superscaffold6-snap-gene-79.21-mRNA-1 | phosphatidylinositol phosphatidylcholine transfer protein sfh12 |  |  |  |
| maker-scaffold3_609-snap-gene-0.33-mRNA-1 | potassium transporter 5 family protein |  |  |  |
| maker-superscaffold18-snap-gene-24.29-mRNA-1 | PREDICTED: citrate-binding protein-like |  |  |  |
| maker-superscaffold40-snap-gene-3.17-mRNA-1 | PREDICTED: laccase-3-like |  |  |  |
| maker-superscaffold1-snap-gene-74.27-mRNA-1 | PREDICTED: laccase-4-like |  |  |  |
| maker-superscaffold8-snap-gene-66.33-mRNA-1 | premnaspirodiene oxygenase-like |  |  |  |
| maker-superscaffold21-snap-gene-6.38-mRNA-1 | probable f-box protein at4g22030-like |  |  |  |
| snap_masked-superscaffold1-processed-gene-23.12-mRNA-1 | probable indole-3-acetic acid-amido synthetase |  |  |  |
| maker-scaffold3_605-snap-gene-1.21-mRNA-1 | probable mannitol dehydrogenase-like |  |  |  |
| snap_masked-superscaffold1-processed-gene-42.13-mRNA-1 | probable pectate lyase 8-like |  |  |  |
| maker-superscaffold12-snap-gene-85.35-mRNA-1 | probable wrky transcription factor 45 |  |  |  |
| snap_masked-superscaffold21-processed-gene-27.2-mRNA-1 | protein early flowering 4 |  |  |  |
| maker-superscaffold8-snap-gene-56.29-mRNA-1 | protein kinase pinoid 2-like |  |  |  |
| snap_masked-superscaffold29-processed-gene-1.14-mRNA-1 | protein translocase subunit 1 |  |  |  |
| maker-scaffold3_609-snap-gene-39.31-mRNA-1 | protein transparent testa 12-like |  |  |  |
| maker-superscaffold30-snap-gene-3.30-mRNA-1 | regulator of chromosome condensation repeat-containing protein |  |  |  |
| maker-superscaffold7-snap-gene-39.46-mRNA-1 | regulatory protein npr5 |  |  |  |
| maker-superscaffold15-snap-gene-100.40-mRNA-1 | respiratory burst oxidase homolog protein b |  |  |  |
| snap_masked-superscaffold1-processed-gene-56.13-mRNA-1 | ring u-box superfamily protein |  |  |  |
| maker-superscaffold12-snap-gene-48.27-mRNA-1 | sensory transduction histidine isoform 1 |  |  |  |
| maker-superscaffold3-snap-gene-11.18-mRNA-1 | stachyose synthase-like |  |  |  |
| maker-superscaffold11-snap-gene-5.7-mRNA-1 | sugar phosphate exchanger |  |  |  |
| maker-superscaffold3-snap-gene-49.22-mRNA-1 | sugar transporter erd6-like 16 isoform x4 |  |  |  |
| maker-superscaffold29-snap-gene-25.21-mRNA-1 | transcription factor bhlh92 |  |  |  |
| maker-superscaffold6-snap-gene-41.33-mRNA-1 | udp-glucosyl transferase 85a2 |  |  |  |
| snap_masked-superscaffold2-processed-gene-40.5-mRNA-1 | udp-glycosyltransferase 74e2-like |  |  |  |
| maker-superscaffold2-snap-gene-48.31-mRNA-1 | udp-glycosyltransferase 85a1-like |  |  |  |
| maker-superscaffold15-snap-gene-109.32-mRNA-1 | yellow stripe like 7 |  |  |  |
| snap_masked-superscaffold7-processed-gene-4.9-mRNA-1 | zinc finger protein zat5 |  |  |  |
